# Multifaceted photocatalysis enables cobalt catalyzed enantioselective C–H activation and APEX reaction for C–N axially chiral molecules

**DOI:** 10.1039/d5sc05287d

**Published:** 2025-09-11

**Authors:** Mainak Koner, Nityananda Ballav, Anirudh J. Varma, Suman Ghosh, Tuhin Mondal, Rositha Kuniyil, Mahiuddin Baidya

**Affiliations:** a Department of Chemistry, Indian Institute of Technology Madras Chennai 600036 India mbaidya@iitm.ac.in; b Department of Chemistry, Indian Institute of Technology Palakkad Palakkad 678623 Kerala India

## Abstract

Molecules with the C–N stereogenic axes have widespread applications, yet their asymmetric synthesis under mild and environmentally friendly conditions is becoming increasingly challenging. Herein, we report the merger of inexpensive photoredox catalysis with a cobalt-catalyzed asymmetric C–H activation/APEX strategy, enabling cascade annulation of (hetero)arenes and alkenes with α-aryl-allenyl acetates to deliver C–N axially chiral benzophenanthridinone and polycyclic quinolinone frameworks with very high yields and enantioselectivities at room temperature. This methodology leverages the multifaceted role of photocatalysis – facilitating the redox event of the cobalt catalyst and the formation of key intermediates that drive the APEX process. It also harnesses the α-aryl-allenyl acetate coupling partners as the formal arene precursors, which have been elusive thus far. The intricacy of the reaction mechanism and the rationale behind the stereoselective outcomes were also unraveled through controlled experiments and DFT studies.

## Abstract 2

A dual photoredox/cobalt-catalyzed C–H activation/APEX strategy couples amides with allenyl acetates, enabling efficient access to C–N axially chiral benzophenanthridinone and quinolinone scaffolds in high yield and enantioselectivity.

## Graphical abstract

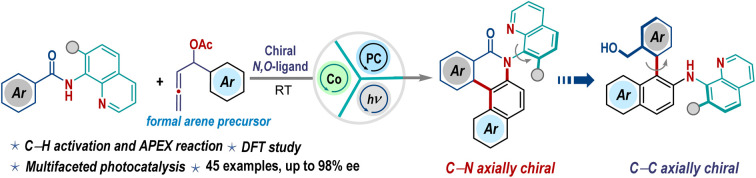

## Introduction

**Scheme 1:**
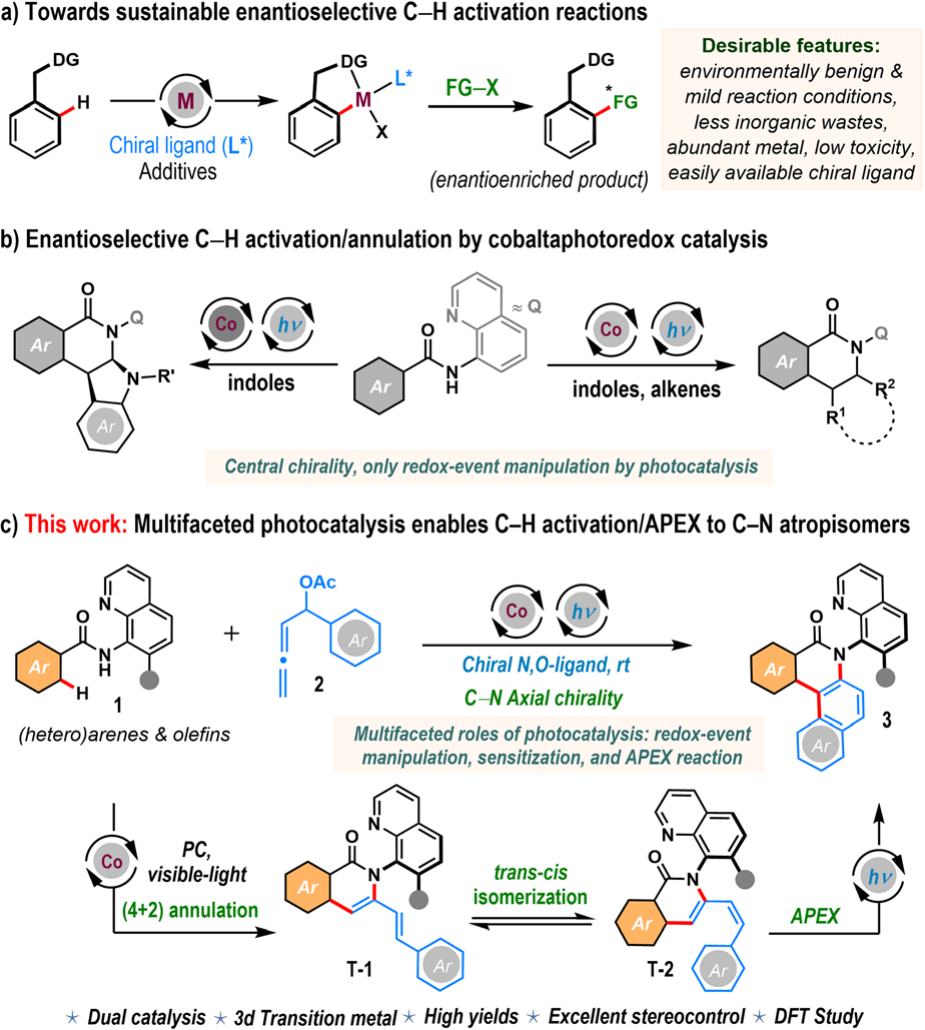
Asymmetric C–H activation/annulation through the merger of visible-light photoredox catalysis.

In the vast landscape of organic synthesis, the transition metal-catalyzed C–H activation strategy stands as a revolutionary technology, challenging traditional methods with its efficiency and selectivity.^1^ Unlike conventional approaches that rely on prefunctionalized starting materials, the C–H bond activation methodology directly manipulates inert C–H bonds within organic molecules, offering broader chemical space and streamlined pathways to complex structures.^2^ However, achieving these transformations with enhanced sustainability and in an enantioselective fashion is becoming increasingly challenging (Scheme 1a). One of the primary hurdles lies in the selection of metal catalysts and chiral ligands. Current innovations heavily rely on inexpensive and less abundant noble metal catalysts, while synthesizing chiral ligands typically requires multistep and tedious procedures. Furthermore, traditional methods for modulating the crucial redox events in the catalytic cycle often necessitate the use of stoichiometric amounts of external metallic oxidants, resulting in significant inorganic waste and undermining sustainability efforts.^1^ A scenario that would dramatically improve the sustainable portfolio of the C–H activation methodology would be capitalizing on earth-abundant first-row transition metal catalysts and integrating visible-light photoredox catalysis with the C–H activation strategy.^3,4^ In this context, cobalt catalysis is highly desirable as cobalt encompasses a relatively nontoxic profile with high abundance in the Earth's crust.^3*f*,*g*^ Further, visible light provides a clean and efficient energy source and could facilitate photoredox catalysis so that different oxidation states of metal can be manipulated during the course of the reaction under mild conditions. Meanwhile, recent breakthroughs, pioneered by Shi and Niu, have underscored the exceptional versatility of bidentate chiral salicyloxazoline (Salox) ligands as powerful enablers of enantioselective C–H activation, especially in cobalt-catalyzed processes.^5^ Despite these promises favoring sustainable catalysis, the enantioselective C–H bond activation reaction through the merger of Co(ii)-catalysis with photoredox catalysis to fabricate chiral frameworks is underdeveloped. Recent contributions from the Shi, Sundararaju, and Ackermann groups have demonstrated early examples of this synergistic cobalta-photoredox strategy, particularly in enantioselective indole dearomatization reactions, which primarily afford central chirality (Scheme 1b).^6–8^ However, such a synergistic cobalta-photoredox catalytic methodology to construct axially chiral molecules is scarce in the literature.^8*b*^ Further, the role of the photocatalyst in most of these transformations is largely confined to redox mediation. This narrow functional scope represents a critical limitation in the field. A key frontier lies in reimagining the role of photocatalysis – not merely as a redox shuttle, but as a multifunctional platform that can orchestrate complex reaction pathways, thereby expanding the synthetic utility of cobaltaphotoredox catalysis in the construction of stereochemically intricate molecules.

Molecules bearing a chiral axis, known as atropisomers, derive their chirality from restricted rotation about a single bond and are prevalent in natural products, bioactive molecules, privileged ligands, and functional materials.^9^ Among these, C–N atropisomers are particularly fundamental, yet their asymmetric synthesis remains challenging due to lower rotational barriers and unstable configurations compared to their C–C axially chiral biaryl congeners.^9*f*,*h*^ We propose a cobaltaphotoredox-catalyzed asymmetric C–H activation/annulation reaction of 8-aminoquinoline-embedded aromatic amides 1 and α-aryl-allenyl acetates 2 as a succinct route to access chiral C–N atropisomers 3 bearing the high-value benzo[*a*]phenanthridinone core (Scheme 1c). This approach is underpinned by several fundamental considerations. While allenes^10^ are typically challenging coupling partners for C–H activation reactions due to their two orthogonal carbon–carbon double bonds, the choice of α-aryl-allenyl acetates (2) in this context is advantageous as versatile synthons capable of introducing dienyl frameworks, crucial for forming intermediate T-1. Photocatalysis is poised to play multiple roles in this endeavor. Aiding with a suitable chiral ligand, it will facilitate the cobalt catalyst's redox event (Co(i)/Co(iii)), promoting asymmetric intermolecular annulative coupling to induce chirality in intermediate T-1. Additionally, it will enable the *trans* to *cis* isomerization of the diene motif, yielding intermediate T-2. This transformation will pave the way for a photoredox-driven annulative π-extension (APEX) reaction,^11^ opening an avenue to the novel realm of C–N axially chiral benzo[*a*]phenanthridinones 3. In a nutshell, α-aryl-allenyl acetates serve as the formal arene precursors, a frontier yet to be fully explored.

Herein, we report the development of this methodology that successfully combines visible-light photoredox catalysis with cobalt-catalyzed asymmetric C–H activation/APEX reactions of (hetero)arenes and alkenes with α-aryl-allenyl acetates, enabling the synthesis of C–N axially chiral benzo[*a*]phenanthridinones and polycyclic quinolinones at room temperature.^12^ The protocol is operationally simple, tolerates a range of functional groups, including heterocyclic motifs, features *de novo* construction of the central heterocyclic core, and remains effective in the presence of pharmaceutical scaffolds. In all cases, products were obtained in high yields and with excellent enantioselectivity. Significantly, the methodology can be tuned for regio- and atroposelective dienylation reactions with high asymmetric induction. Additionally, DFT studies along with control experiments were conducted to unravel the intricate reaction mechanism and the rationale behind the stereoselective outcomes. It is worth noting that, in sharp contrast to previous studies where photocatalysis was limited to modulating the redox state of metal catalysts, our work reveals a multifaceted role for photocatalysis – simultaneously facilitating redox events, enabling sensitization-driven *trans-cis* isomerization, and driving the APEX reaction – thereby establishing a distinctly broadened catalytic framework.

## Results and discussion

We commenced our investigation by exposing a mixture of amide 1a and α-phenyl-allenyl acetate 2a to different cobalt complexes generated *in situ* from Co(acac)_2_ and chiral ligands L1–L4 in trifluoroethanol (TFE) solvent. The reaction mixture was irradiated with 427 nm Kessil LED light, using Eosin Y as a cost-effective organic photocatalyst and NaOPiv·H_2_O base at room temperature under an oxygen atmosphere. Satisfyingly, the envisioned cascade annulation proceeded with ligand (S)-L1, delivering the desired C–N axially chiral benzo[*a*]phenanthridinone 3a in 45% yield and 94% ee (entry 1). Consideration of chiral ligand (S)-L2 with a benzyl substituent improved the yield to 56%, albeit with reduced enantioselectivity (70% ee, entry 2). Ligand (S)-L3, featuring an isopropyl group, also resulted in diminished yield and enantioselectivity (entry 3). Further exploration identified chiral ligand (S)-L4, bearing a methoxy group on the phenol unit, as highly effective, delivering product 3a in 78% yield and 94% ee (entry 4). Examination of different protic solvents such as hexafluoroisopropanol (HFIP) or *t*BuOH, and aprotic solvents, for example, 1,2-dichloroethane (DCE) and CH_3_CN, gave detrimental outcomes (entries 5–8). In the absence of Eosin Y, both yield and ee dropped significantly, substantiating its significance in this cascade (entry 9). Screening of other cobalt salts revealed enhanced reactivity with Co(OAc)_2_·4H_2_O, offering 3a in 81% yield with 94% enantioselectivity (entry 10), whereas the reaction was unsuccessful with CoBr_2_ (entry 11). When the reaction was conducted in the absence of light, it prematurely stopped at the annulative dienylation stage, furnishing 5a instead of 3a (elaborated in the later section, Scheme 3), and it is also in line with our initial hypothesis (entry 12). The reaction efficacy also drastically reduced upon alteration of the light source to 390 nm or 456 nm (entries 13–14).

**Scheme 2:**
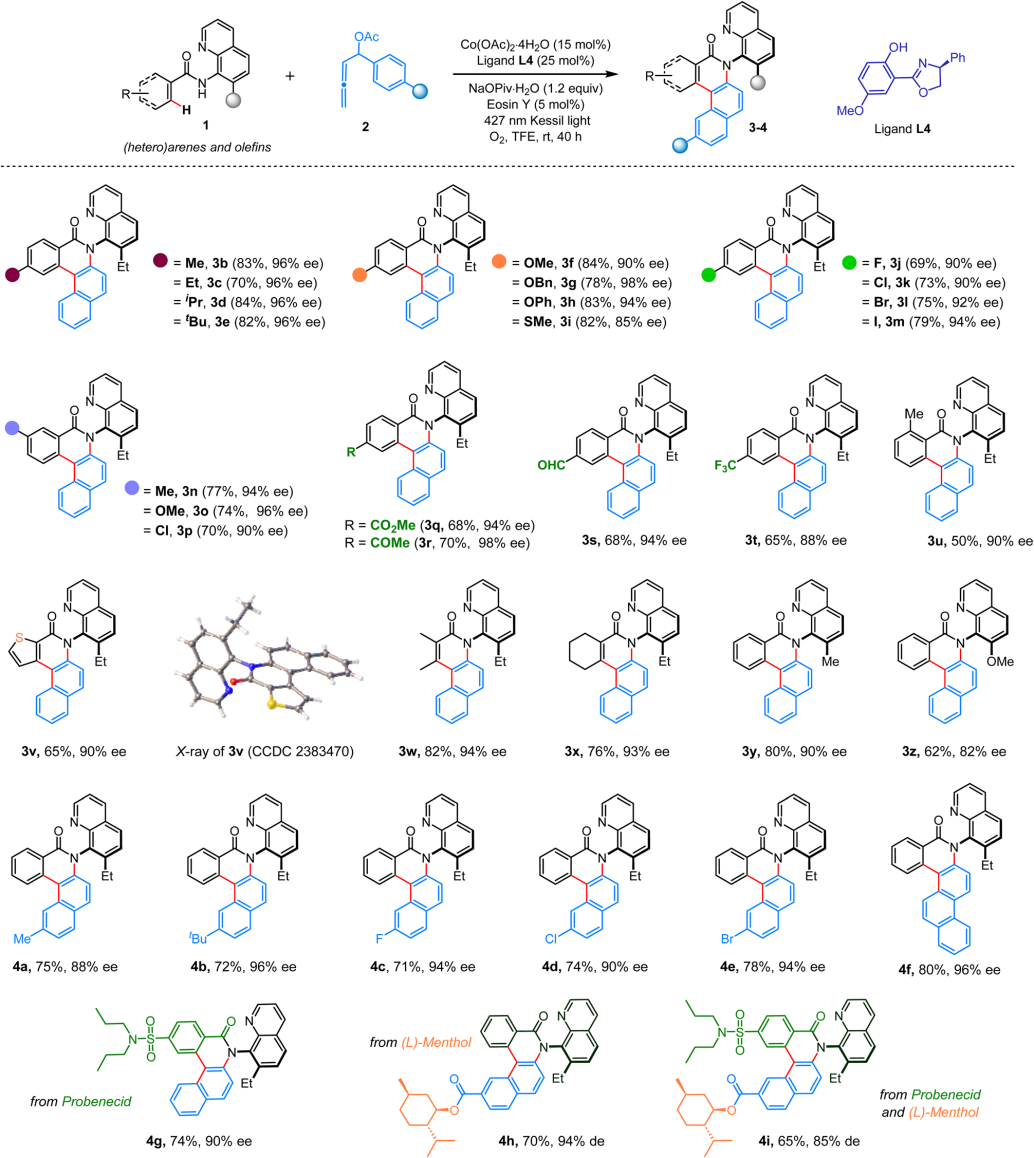
Exploration of substrate scope. ^*a*^Reaction conditions: 1 (0.2 mmol), 2 (0.3 mmol), TFE (0.1 M) for 40 h. Isolated yields were provided.

With the optimized reaction conditions (Table 1, entry 10), we then explored the substrate scope (Scheme 2). Initially, variation in aromatic amides (1) was considered, which proved to be quite general. Aromatic amides bearing electron-donating alkyl (3b–3e), alkoxy (3f), benzyloxy (3g), and aryloxy (3h) moieties and halogen functionalities such as fluoro (3j), chloro (3k), bromo (3l), and iodo (3m) groups at the *para*-position readily participated in the production of C–N axially chiral benzo[*a*]phenanthridinone molecules in very high yields and enantioselectivities. Similarly, *meta*-substituted benzamides furnished the desired tetracyclic C–N atropisomers 3n–3p in very high yields and enantioselectivities. Gratifyingly, various useful functional groups, including ester (3q), ketone (3r), aldehyde (3s), thiomethyl (3i), and trifluoromethyl (3t), were well tolerated. This annulation was also practical with a bulky *ortho*-substituted amide, offering 3u in 50% yield with 90% ee. An aromatic amide with a heterocyclic thiophene moiety did not hamper the APEX reaction, delivering 3v in 90% ee. The compound 3v was crystallized, and the single crystal X-ray analysis unambiguously confirmed the product structure and its absolute R configuration. The absolute configurations of other compounds were analogously assigned. Notably, alkenyl amides are also good substrates for this annulative coupling. Amides obtained from tiglic acid and 1-cyclohexene-1-carboxylic acid offered benzo[*f*]quinolinone embedded C–N atropisomers 3w and 3x, with 94% ee and 93% ee, respectively (Scheme 2). Variation in the quinoline ring was also considered. For example, a 7-methyl-substituted 8-aminoquinoline directing group delivered the desired product 3y in 80% yield with 90% ee. However, replacing the methyl group with a methoxy substituent resulted in a lower yield and ee for the product 3z. Next, the variation in the allene unit (2) was investigated (Scheme 2). Under the standard reaction conditions, various α-aryl-allenyl acetates having methyl, *tert*-butyl, fluoro, chloro, and bromo groups at the aryl ring uniformly generated C–N axially chiral benzo[*a*]phenanthridinone molecules 4a–4e in high yields and high enantioselectivity. Employing a naphthyl-substituted allene, the synthetically challenging polycyclic C–N atropisomer 4f was prepared in 80% yield with 96% ee.

**Table 1:** Optimization of reaction conditions^a^

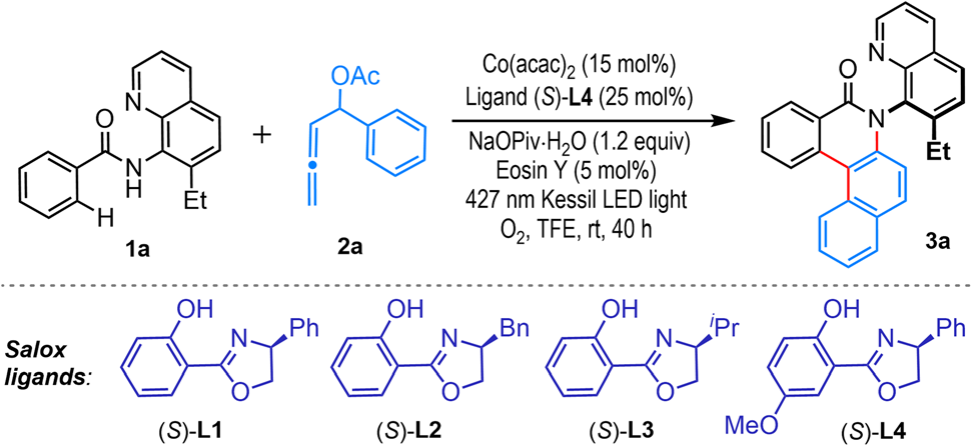			
Entry	Deviation from the standard conditions	Yield of 3a (%)^b^	ee of 3a (%)^c^
1	Ligand L1	45	94
2	Ligand L2	56	70
3	Ligand L3	40	65
4	None	78	94
5	HFIP instead of TFE	20	NP
6	*t*BuOH instead of TFE	68	91
7	DCE instead of TFE	60	90
8	CH_3_CN instead of TFE	75	88
9	Without eosin Y	69	80
10	With Co(OAc)_2_·4H_2_O	81	94
11	With CoBr_2_	—	—
12	Without light	—d	—
13	With 390 nm Kessil LED light	35	94
14	With 456 nm Kessil LED light	50^e^	NP

a Reaction conditions: 1a (0.2 mmol), 2a (0.3 mmol), base (0.24 mmol), and TFE (2.0 mL), 40 h, under an O_2_ (balloon) atmosphere.

b Isolated yields were provided.

c Determined by chiral HPLC.

d Annulative dienylation product 5a was formed in 55% yield (see the discussion in the later section).

e Product 5a was formed along with 3a. NP: not performed.

To underscore the synthetic utility, we performed this APEX strategy with substrates consisting of biologically relevant scaffolds (Scheme 2). Amides derived from probenecid, a commercial drug to treat chronic gouty arthritis, generated C–N atropisomer 4g in 74% yield with 90% ee. An allene bearing a natural product, (L)-menthol, as a core delivered product 4h as a single observable diastereomer. To further expand scaffold diversity, we planned the challenging APEX reaction with coupling partners featuring different bioactive scaffolds. Accordingly, a probenecid-derived amide was reacted with an (L)-menthol embedded allene under standard reaction conditions, resulting in a functionally enriched C–N atropisomer 4i, isolated as a single diastereomer (Scheme 2).

**Scheme 3:**
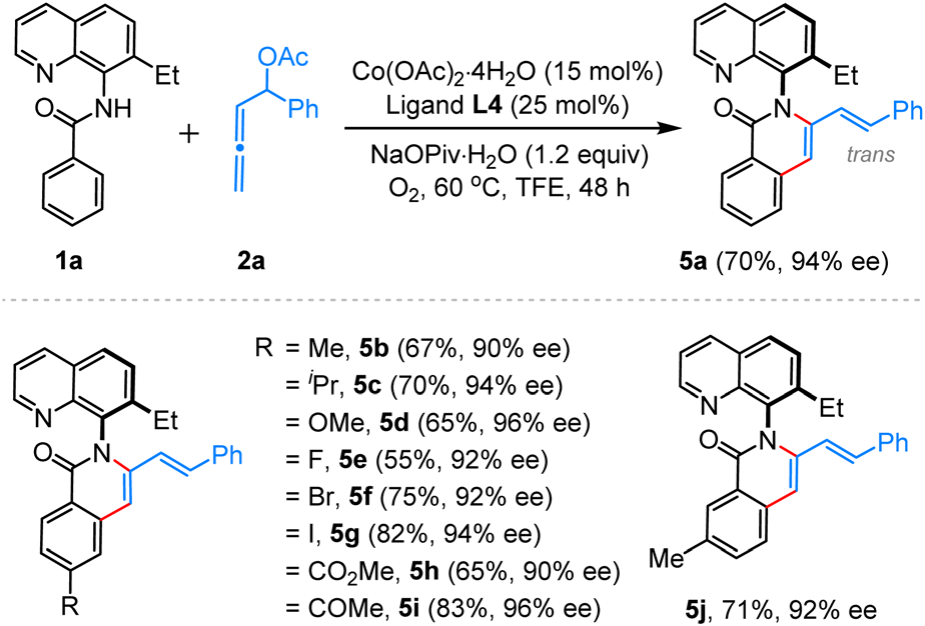
Synthesis of C–N atropisomers with 3-styrenylisoquinolinone core. ^*a*^Reaction conditions: 1a (0.2 mmol), 2 (0.3 mmol), TFE (0.1 M) for 48 h. Isolated yields were provided.

As indicated in the preceding section (Table 1, entry 12), our reaction is halted at the annulative dienenylation stage in the absence of light. Motivated by this result and noting the scarcity of literature reports on asymmetric dienenylation strategies,^13^ we further optimized the reaction conditions to exclusively produce C–N atropisomers of this type. Remarkably, when the reaction between aromatic amide 1a and 2a was carried out at 60 °C under an oxygen atmosphere, without a photocatalyst and LED light, the desired product 5a was obtained with a 70% yield and 94% ee (Scheme 3). This protocol proved to be general for a range of aromatic amides, facilitating the creation of a small library of functionally enriched C–N atropisomers with a 3-styrenylisoquinolinone core (5b–5j). Notably, all C–N atropisomers were produced with very high ee and exclusive E-selectivity (Scheme 3).

**Scheme 4:**
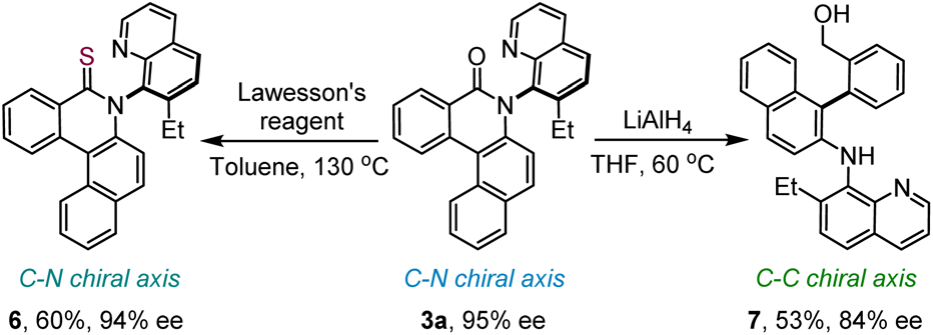
Post-synthetic manipulations.

Moreover, the axially chiral product 3a was transformed into axially chiral thiolactam 6 in good yield upon treatment with Lawesson's reagent (Scheme 4, left). Further, 3a was reacted with LiAlH_4_ to afford 7 in 53% yield with 84% ee. Intriguingly, during this post-functionalization, the chiral axis shifts from C–N to C–C, resulting in a functionally enriched biaryl framework (Scheme 4, right).

**Scheme 5:**
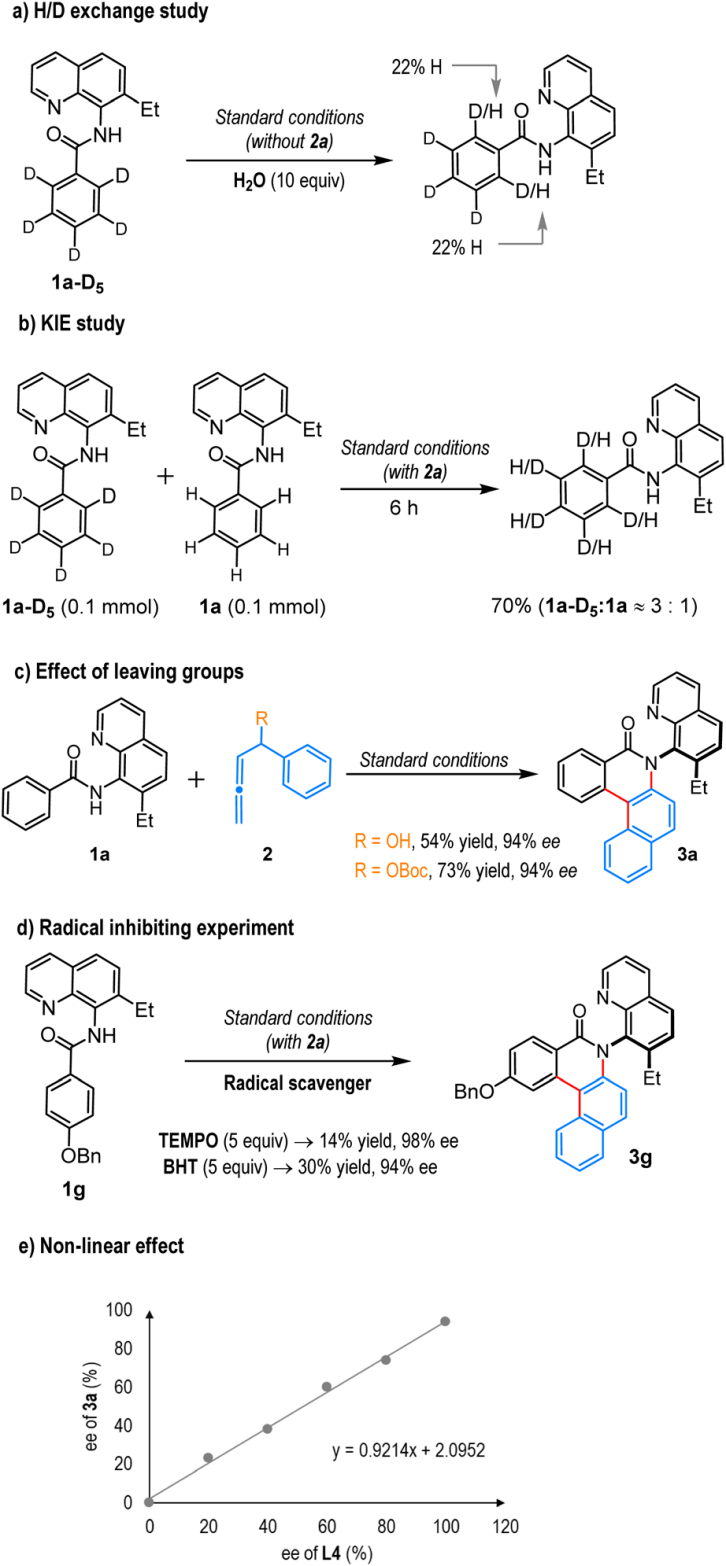
Control experiments.

To understand the nature of the C–H cobaltation step, we performed a deuterium labeling experiment. When amide 1a-D_5_ was exposed to the standard reaction conditions in the presence of H2O, the deuterium-hydrogen exchange in 1a-D_5_ was significant, indicating the C–H cobaltation step is reversible (Scheme 5a). Further, the parallel KIE study revealed a ≈3 : 1 ratio of kH/kD suggesting the C–H activation step is most likely not the rate-limiting step (Scheme 5b). To leverage the effect of the leaving group in the allene unit, an annulation reaction was performed with hydroxyl and *tert*-butoxycarbonyl (Boc) group substituted allenes, where the desired product 3a was isolated in lower yields, although ee was unchanged (Scheme 5c). The presence of radical scavengers also impedes the progress of this annulation reaction, suggesting involvement of radical intermediates (Scheme 5d). We also found that the enantiomeric excess of product 3a shows a linear correlation with the enantiopurity of the chiral ligand (L4), validating that a single chiral ligand coordinates with a cobalt atom to form the active cobalt catalyst (Scheme 5e).^14^

**Scheme 6:**
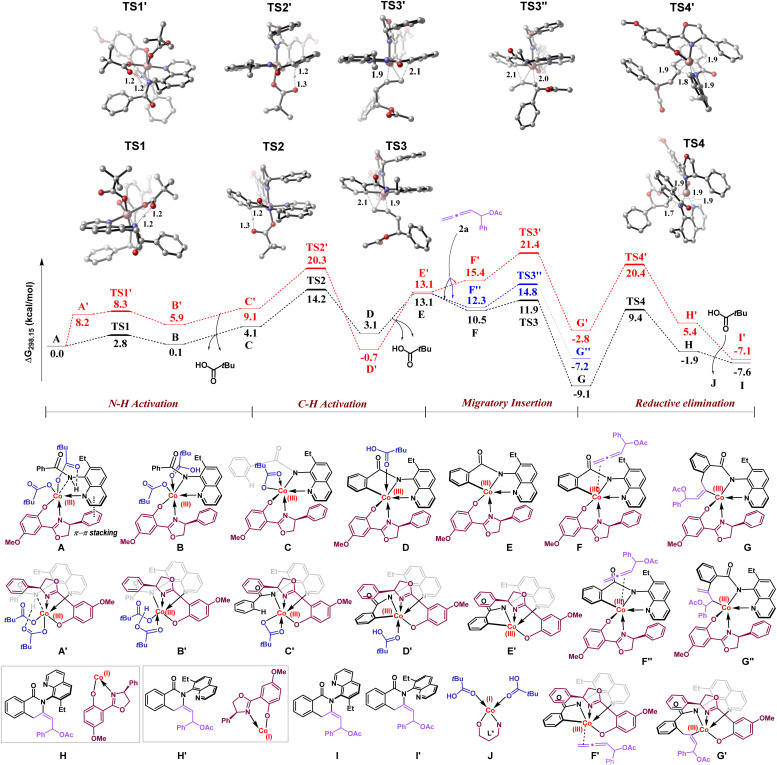
DFT calculations.

To understand the rationale behind the regio- and enantioselectivity of this catalytic cascade annulation, DFT calculations were performed at the M06-D3/6-311 ++ G(d,p), SDD(Co) + SMD(TFE)//M06-D3/6-31G(d), SDD(Co) level of theory (Scheme 6).

**Fig. 1:**
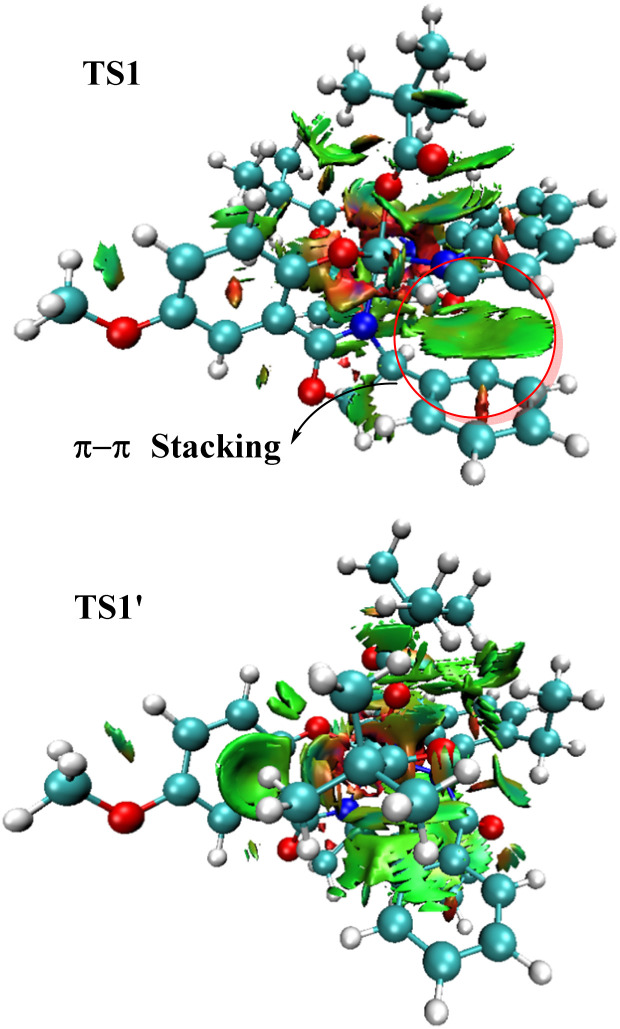
NCI plots for N–H activation transition states TS1 and TS1′.

The mechanism begins with the catalytically active cobalt(iii) species A, which undergoes pivalate-assisted N–H bond activation to form the intermediate B, as depicted in the black pathway. During this step, the amide hydrogen of the substrate is abstracted by the pivalate ion *via* the transition state TS1, having an energy barrier of 2.8 kcal mol^−1^. Subsequent removal of pivalic acid gives intermediate C, from which the C–H bond activation occurs. Here, the proximal *ortho* C–H bond is cleaved by the metal-bound pivalate ion through the transition state TS2 (14.2 kcal mol^−1^) and generates the intermediate D. Then, ligand exchange happens to give allene coordinated cobalt(iii) species F and F′′, which are well-set for the migratory insertion process. Both 1,2-insertion (*via*TS3, 11.9 kcal mol^−1^) and 2,3-insertion (*via*TS3′′, 14.8 kcal mol^−1^) of allene 2a into the metal–carbon bond were examined, with the 1,2-insertion pathway favored by 2.9 kcal mol^−1^. This regioselective migratory insertion affords the stable intermediate G. Subsequently, the rate-determining reductive elimination proceeds from G through the transition state TS4, with a barrier of 18.5 kcal mol^−1^, leading to the pre-product complex H. This intermediate eventually gives rise to the R-atropisomer of the final product 3a. A comparison of pathways leading to the R-atropisomer (black line) and the S-atropisomer (red line), where the major difference lies in the orientation of the chiral ligand, revealed that all key steps such as N–H activation (TS1′, 8.3 kcal mol^−1^), C–H activation (TS2′, 20.3 kcal mol^−1^), migratory insertion (TS3′, 21.4 kcal mol^−1^), and reductive elimination (TS4′, 20.4 kcal mol^−1^) have significantly higher barriers for the pathway leading to the S-enantiomer. These findings clearly indicate a kinetic preference for the formation of the R-isomer, which aligns well with our experimental observations. Further, to rationalize the enhanced stability of transition state TS1 compared to TS1′, the non-covalent interactions (NCIs) were analyzed using a Non-Covalent Interaction (NCI) plot. In the NCI graphical depiction (Fig. 1), strong attractive interactions are indicated by blue areas, while dispersion or weak noncovalent interactions are denoted by green areas, and repulsive interactions are represented by red areas. The NCI plot for TS1 reveals a distinct π–π stacking interaction, which contributes significantly to its greater stability relative to TS1′ (Fig. 1).

## Conclusions

In conclusion, we have demonstrated a dual photoredox/cobalt-catalyzed C–H activation and APEX reaction that efficiently couples 8-aminoquinoline embedded aromatic amides with allenyl acetates at room temperature. The cascade reaction offers challenging C–N axially chiral polycyclic benzophenanthridinones in high yields and excellent enantioselectivities. It employs an inexpensive organic photocatalyst (Eosin Y) and the photocatalysis plays multiple roles: facilitating the redox event of the cobalt catalyst, promoting the essential olefin isomerization, and driving the pivotal APEX cascade. It also strategically employs α-aryl-allenyl acetate (2) as a formal arene precursor, which has previously been elusive. This approach is sustainable, enables *de novo* construction of an arene ring, features broad substrate generality, and remains effective in the presence of biorelevant scaffolds. Importantly, the method can be fine-tuned for regio- and atroposelective dienylation with high asymmetric induction. Also, the C–N axially chiral product can be converted into a C–C axially chiral biaryl through reduction. In-depth DFT calculations, along with control experiments, provide a comprehensive understanding of the reaction's intricate pathway and stereoselective outcomes, where the reductive-elimination is the rate-limiting step. Notably, this methodology represents a rare instance of enantioselective cobaltaphotoredox catalyzed annulative π-extension at room temperature for producing valuable axially chiral molecules, laying the foundation for future advancements.

## Author contributions

The manuscript was written through contributions of all authors. All authors have given approval to the final version of the manuscript. M. B. and M. K. have conceptualized the idea. M. K., N. B., S. G. and T. M. carried out the experiments and mechanistic investigations, and analyzed experimental data. R. K. and A. J. V. conducted the computational studies. All the authors discussed the results and co-wrote the manuscript.

## Conflicts of interest

There are no conflicts to declare.

## Supplementary Material

SC-016-D5SC05287D-s001

SC-016-D5SC05287D-s002

SC-016-D5SC05287D-s003

## Data Availability

CCDC 2383470 contains the supplementary crystallographic data for this paper.^15^ The data supporting this article have been included as part of the SI. See DOI: https://doi.org/10.1039/d5sc05287d.
