# Supplementary material for: Multifaceted photocatalysis enables cobalt catalyzed enantioselective C–H activation and APEX reaction for C–N axially chiral molecules
Source: Chem Sci. 2025 Sep 11;16(41):19296–303. doi: 10.1039/d5sc05287d (PMC12447758; doi:10.1039/d5sc05287d)
Supplement: SC-016-D5SC05287D-s001 [file SC-016-D5SC05287D-s001.pdf]

Supplementary Information (SI) for Chemical Science.
This journal is © The Royal Society of Chemistry 2025

Electronic Supporting Information

PART A

Multifaceted Photocatalysis Enables Cobalt Catalyzed Enantioselective C–H Activation and APEX Reaction for C–N Axially Chiral Molecules

Mainak Koner,[a] Nityananda Ballav,[a†] Anirudh J Varma,[b†] Suman Ghosh,[a] Tuhin Mondal,[a] Rositha Kuniyil,*[b] and Mahiuddin Baidya*[a]

[a]Department of Chemistry, Indian Institute of Technology Madras

Chennai 600036, Tamil Nadu, India

E-mail: mbaidya@iitm.ac.in

[b]Department of Chemistry, Indian Institute of Technology Palakkad

Palakkad 678623, Kerala, India

E-mail: rosithak@iitpkd.ac.in

[†]These authors contributed equally to the work.

-Table of Contents-

General Information	S3
1. General procedure (GP-A) for the synthesis of products 3 and 4	S4
2. General procedure (GP-B) for the synthesis of products 5	S5
3. Plausible Mechanistic cycle	S6
4. Procedure for the synthesis of compound 6	S7
5. Procedure for the synthesis of compound 7	S8
5. Mechanistic Studies	S9 - S13
7. Crystallographic Experimental Data	S14 – S15
8. Characterization of Products	S16 – S62
9. NMR Spectra	S63 – S114

General Information

All non-aqueous reactions were carried out under an atmosphere of nitrogen in flame-dried glassware and were stirred using a magnetic stir plate. All reactions were carried out using commercial-grade solvent unless otherwise noted. CH_3CN, DCE, and CH_2Cl_2 were dried over calcium hydride. Dry THF was prepared by distilling over sodium ketyl. All reactions were monitored by thin layer chromatography (TLC) on WhatmanPartisil® K6F TLC plates (silica gel 60 Å, 0.25 mm thickness) and visualized using a UV lamp (366 or 254 nm) or by use of one of the following visualization reagents: $KMnO_4$: 0.75g potassium permanganate, 5g K_2CO_3 / 100 mL water. Products were isolated by column chromatography (Merck silica gel 100-200µm). Yields refer to chromatographically and spectroscopically homogenous materials unless noted otherwise. ^1H, ^{13}C and ^{19}F NMR spectra were recorded on Bruker 400 or Bruker 500 MHz spectrometers. Chemical shift values (δ) are reported in ppm and calibrated to the residual solvent peak $CDCl_3$ δ = 7.26ppm for ^1H, δ = 77.16 for ^{13}C; or calibrated to tetramethylsilane (δ = 0.00 ppm). All NMR spectra were recorded at ambient temperature (290 K) unless otherwise noted. ^1H NMR spectra are reported as follows: chemical shift (multiplicity, coupling constant, integration). The following abbreviations are used to indicate multiplicities: s, singlet; d, doublet; t, triplet; q, quartet; m, multiplet; dd, doublet of doublet; dt, doublet of triplet; dq, doublet of quartet; td, triplet of doublet; tt, triplet of triplet; dq, doublet of quartet; br, broad; ddd, doublet of doublet of doublet.

Mass spectra were recorded by electrospray ionization (ESI) method on a Q-TOF Micro with lock spray source. The crystal data were collected and integrated using a BrukerAxs kappa apex2 CCD diffractometer, with graphite monochromated Mo-Kα radiation.

High pressure liquid chromatography (HPLC) analyses were performed on a Shimadzu instrument using a chiral stationary phase column (Daicel Co. CHIRALPAK). The chiral HPLC methods were calibrated with the corresponding racemic mixtures.

The substituted benzamides **1** were synthesized following literature procedures a. X. J. Si, D. Yang, M. C. Sun, D. Wei, M. P. Song and J. L. Niu, *Nat. Synth., 2022,* **1**, 709–718. b. T. von Münchow, S. Dana, Y. Xu, B. Yuan and L. Ackermann, *Science,* 2023, **379**, 1036–1042. c. Y. Lin, T. von Münchow and L. Ackermann, *ACS catal.,* 2023**,** **13**, 9713-9723. All α-aryl allenyl acetates **2** were prepared following the reported procedure (A. Singh, R. K. Shukla and C. M. R. Volla, *Chem. Sci.,* 2022, **13**, 2043-2049). Chiral ligands were prepared following the

reported procedure (Q. J. Yao, J.-H. Chen, H. Song, F.-R. Huang and B. F. Shi, *Angew. Chem. Int. Ed.* 2022, **61**, e202202892).

General procedure (GP-A) for the synthesis of products 3 and 4

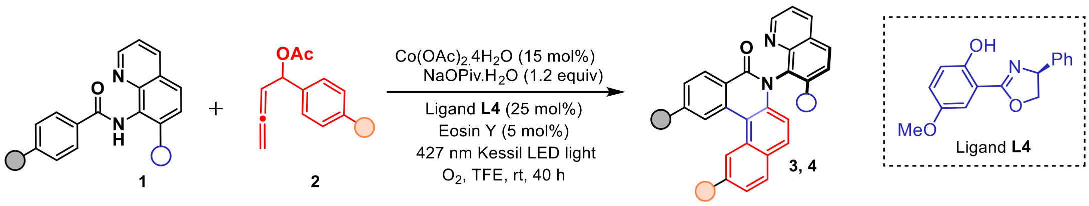

A 15 mL dry reaction tube equipped with a magnetic stir bar was charged with benzamide **1** (0.2 mmol), *α*-aryl-allenyl acetate **2** (0.3 mmol), $Co(OAc)_2·4H_2O$ (15 mol%), **L4** (25 mol%), Eosin Y (5 mol%), and $NaOPiv·H_2O$ (1.2 equiv). Anhydrous TFE solvent (2.0 mL) was added via syringe. The reaction system was sealed with a stopper and equipped with an O_2 balloon to maintain a constant oxygen supply. The mixture was then irradiated with 427 nm Kessil light and stirred for 40 h. Upon completion, the reaction mixture was diluted with CH_2Cl_2 (20 mL), filtered through a pad of Celite, and concentrated under reduced pressure. The resulting crude product was purified by column chromatography using a petroleum ether/ethyl acetate (PE/EA) gradient to obtain the pure chiral product.

For the racemic samples, reactions were performed with **racemic L4** ligand.

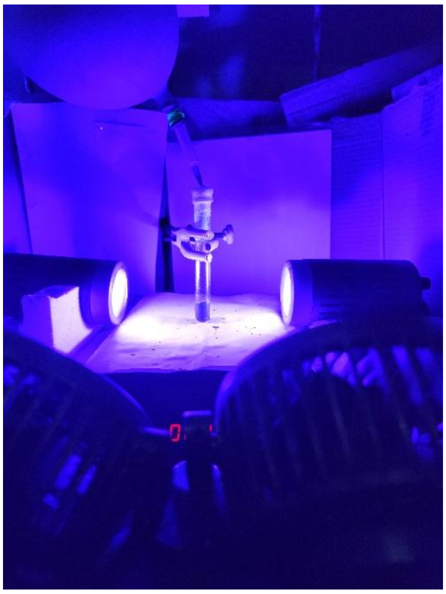

Fig. 1: Picture of the Reaction Setup (Photochemical)

General procedure (GP-B) for the synthesis of product 5

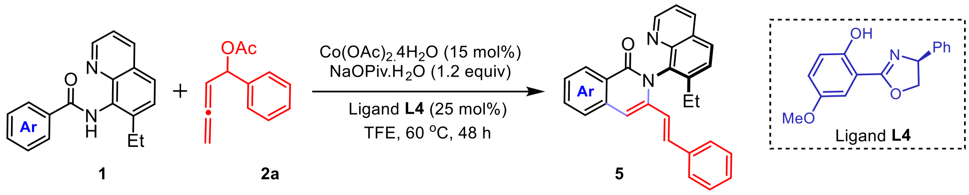

A 15 mL dry reaction tube equipped with a magnetic stir bar was charged with benzamide **1** (0.2 mmol), *α*-aryl-allenyl acetate **2a** (0.3 mmol), $Co(OAc)_2 \cdot 4H_2O$ (15 mol%), **L4** (25 mol%), and NaOPiv·H_2O (1.2 equiv). Anhydrous TFE solvent (2.0 mL) was added via syringe. Then the reaction system was closed with a stopper, purged with oxygen, and then stirred at 60 °C for 48 h. After completion, the reaction mixture was diluted with CH_2Cl_2 (20 mL), filtered through a pad of celite, and concentrated under reduced pressure. The crude product was purified by column chromatography using petroleum ether/ethyl acetate (PE/EA) as eluent to furnish pure chiral product.

For the racemic samples, reactions were performed with **racemic L4** ligand.

Plausible mechanistic cycle

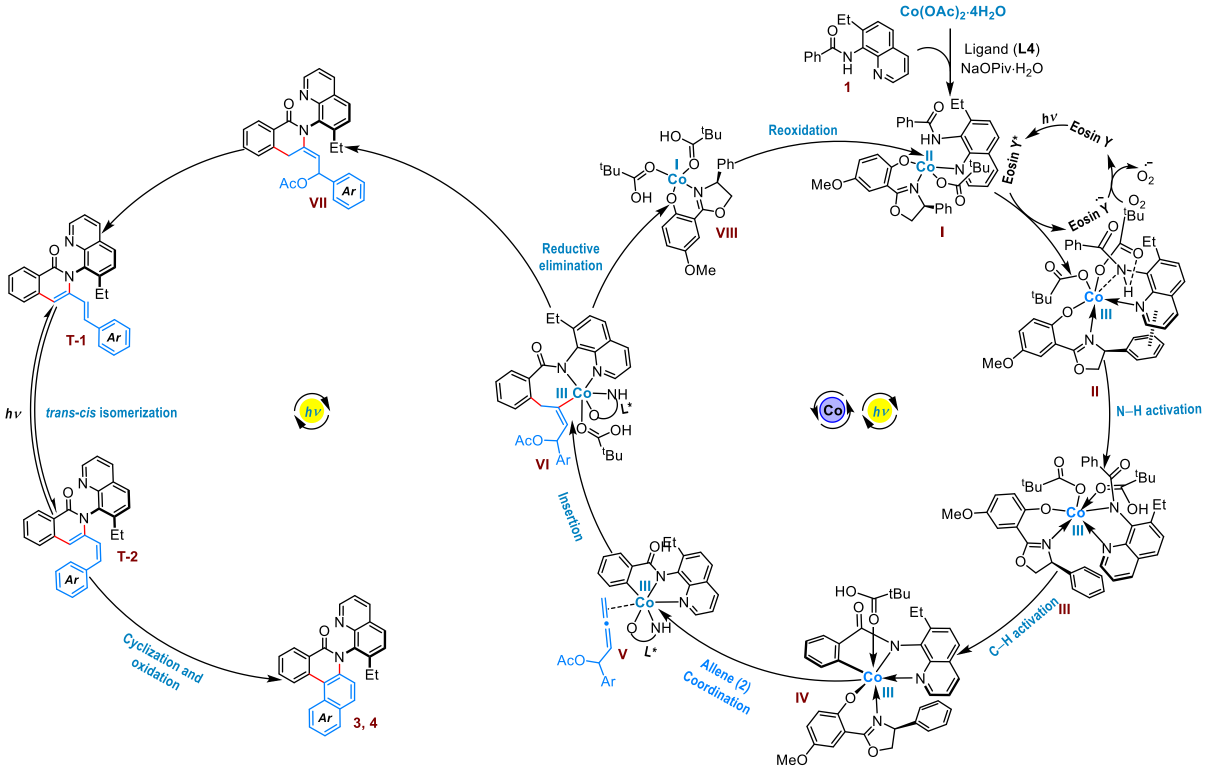

A plausible mechanism is depicted above. Initially, Co(II)-complex **I** is formed through ligand exchange with benzamide **1**, chiral ligand **L4**, and sodium pivalate. The complex then undergoes oxidation by the excited photocatalyst (Eosin Y*) to give Co(III)-species **II**, which undergoes pivalate-assisted N–H bond activation to form the intermediate **III.** Following this, carboxylate-assisted C−H activation occurs, resulting in the formation of cobaltacycle **IV**. This intermediate subsequently coordinates with the external π-bond of allene **2**, generating intermediate **V** through regioselective insertion. The next step involves reductive coupling, leading to the formation of the annulated intermediate **VII** and Co(I)-species **VIII**. The Co(I)-species is then reoxidized to Co(II)-species, allowing the catalytic cycle to continue. The annulated intermediate **VII** transforms into styrenylisoquinolinone **T-1** through the elimination of AcOH. Further, light-mediated *trans-cis* isomerization gives intermediate **T-2**, from which electrocyclization and concomitant oxidation furnish the desired C−N axially chiral product **3, 4**.

Procedure for the synthesis of compound 6

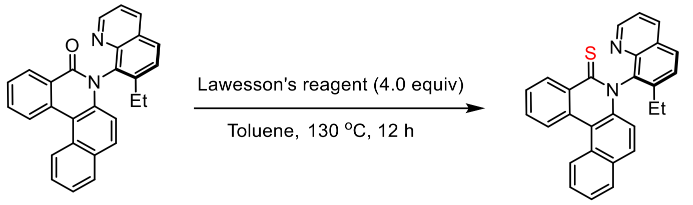

3a, 95% ee

6, 60%, 94% ee

To a 15 mL dry Schlenk tube with a magnetic stir bar, chiral benzo[*a*]phenanthridin-5(6*H*)-one derivative **3a** (0.1 mmol) and Lawesson's reagent (0.4 mmol) were taken. Tube was capped with a septum, evacuated under vacuum, and carefully backfilled with nitrogen. Anhydrous toluene (0.8 mL) was then added via syringe under nitrogen and the mixture was stirred at 130 °C for 12 h. After completion, the reaction mixture was diluted with CH_2Cl_2 (20 mL), filtered through a pad of celite, and concentrated under reduced pressure. The crude product was purified by column chromatography using petroleum ether/ethyl acetate (PE/EA) as eluent to furnish pure product **6**.

For the racemic sample, the above reaction was performed with racemic benzo[*a*]phenanthridin-5(6*H*)-one derivative **3a**.

Procedure for the synthesis of compound 7

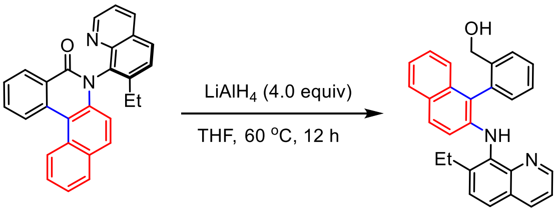

3a, 95% ee

7, 53%, 84% ee

To a 15 mL dry Schlenk tube with a magnetic stir bar, chiral benzo[*a*]phenanthridin-5(6*H*)-one derivative **3a** (0.1 mmol) was taken. The tube was capped with septum, evacuated under vacuum and backfilled with nitrogen. Freshly prepared anhydrous THF (1.0 mL) was then added to the tube and placed at 0 °C. $LiAlH_4$ (4.0 equiv) was then added slowly to the reaction mixture under the flow of nitrogen. Then the reaction system was closed with a stopper and stirred at 60 °C for 12 h. After completion, the reaction was carefully quenched with water and treated with ethyl acetate. The organic layer was separated, dried over sodium sulphate, and concentrated in vacuum. The crude product was purified by column chromatography using petroleum ether/ethyl acetate (PE/EA) as eluent to furnish pure product **7**.

For the racemic sample, the above reaction was performed with racemic benzo[*a*]phenanthridin-5(6*H*)-one derivative **3a**.

Mechanistic Studies:

1. H/D Exchange experiment:

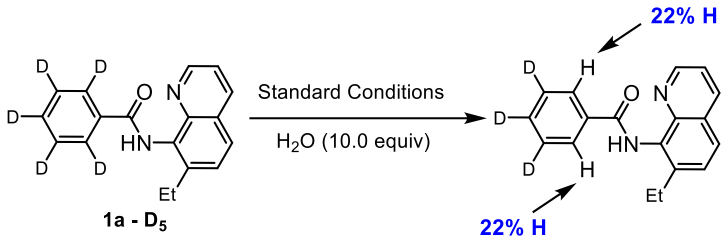

A 15 mL dry reaction tube equipped with a magnetic stir bar was charged with benzamide **1a–D5** (0.1 mmol), $Co(OAc)_2·4H_2O$ (15 mol%), **L4** (25 mol%), Eosin Y (5 mol%), $NaOPiv·H_2O$ (1.2 equiv), and H_2O (10.0 equiv). Anhydrous TFE solvent (1.0 mL) was added via syringe. The reaction system was sealed with a stopper and equipped with an O_2 balloon to maintain a constant oxygen supply. The mixture was then irradiated with 427 nm Kessil light and stirred for 10 h. After completion, the reaction mixture was diluted with CH_2Cl_2 (20 mL) and filtered through a pad of celite, and then concentrated under vacuum. The crude product was purified by column chromatography using PE/EA (3:1) as eluent. ^{1}H-NMR analysis showed 22% Hydrogen incorporation at ortho position.

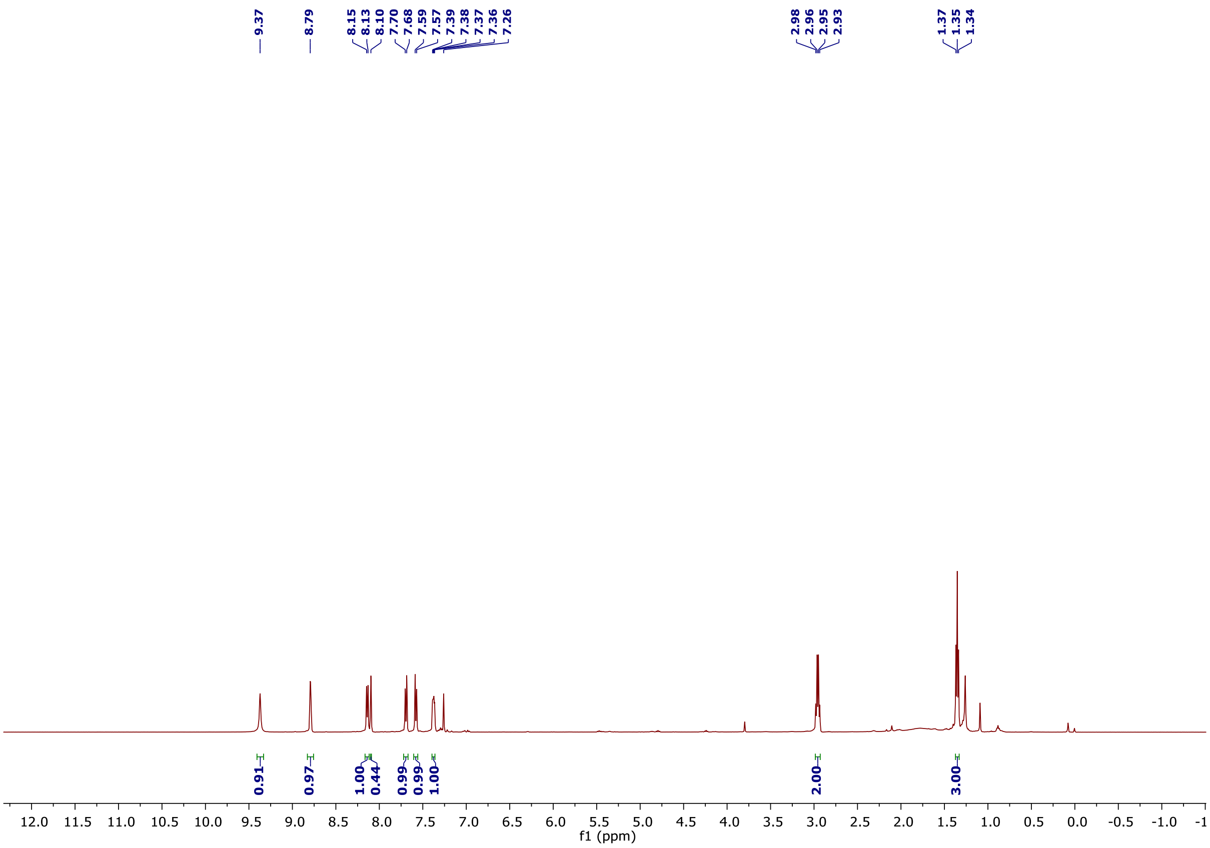

2. KIE Study:

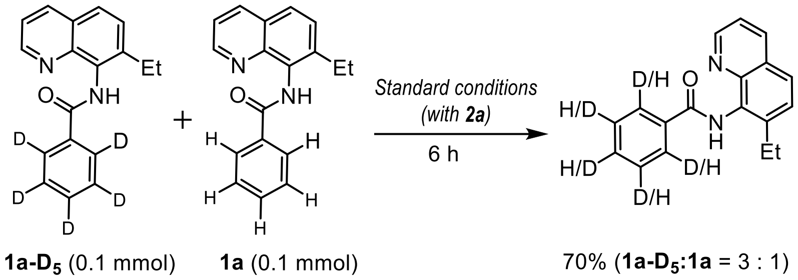

A 15 mL dry reaction tube equipped with a magnetic stir bar was charged with benzamide **1a-D**$_5$ (0.1 mmol), benzamide **1a** (0.1 mmol), *α*-aryl-allenyl acetate **2a** (0.3 mmol), $Co(OAc)_2 \cdot 4H_2O$ (15 mol%), **L4** (25 mol%), Eosin Y (5 mol%), and $NaOPiv \cdot H_2O$ (1.2 equiv). Anhydrous TFE solvent (2.0 mL) was added via syringe. The reaction tube was sealed with a stopper and equipped with an O_2 balloon to maintain a constant oxygen supply. The mixture was then irradiated with 427 nm Kessil light and stirred for 6 h. After completion, the reaction mixture was diluted with CH_2Cl_2 (20 mL) and filtered through a pad of celite, and then concentrated under vacuum. The crude product was purified by column chromatography using PE/EA (3:1) as eluent. ^{1}H NMR analysis of the unreacted starting materials gave a ratio of ≈3:1 of **1a-D**$_5$ and **1a**.

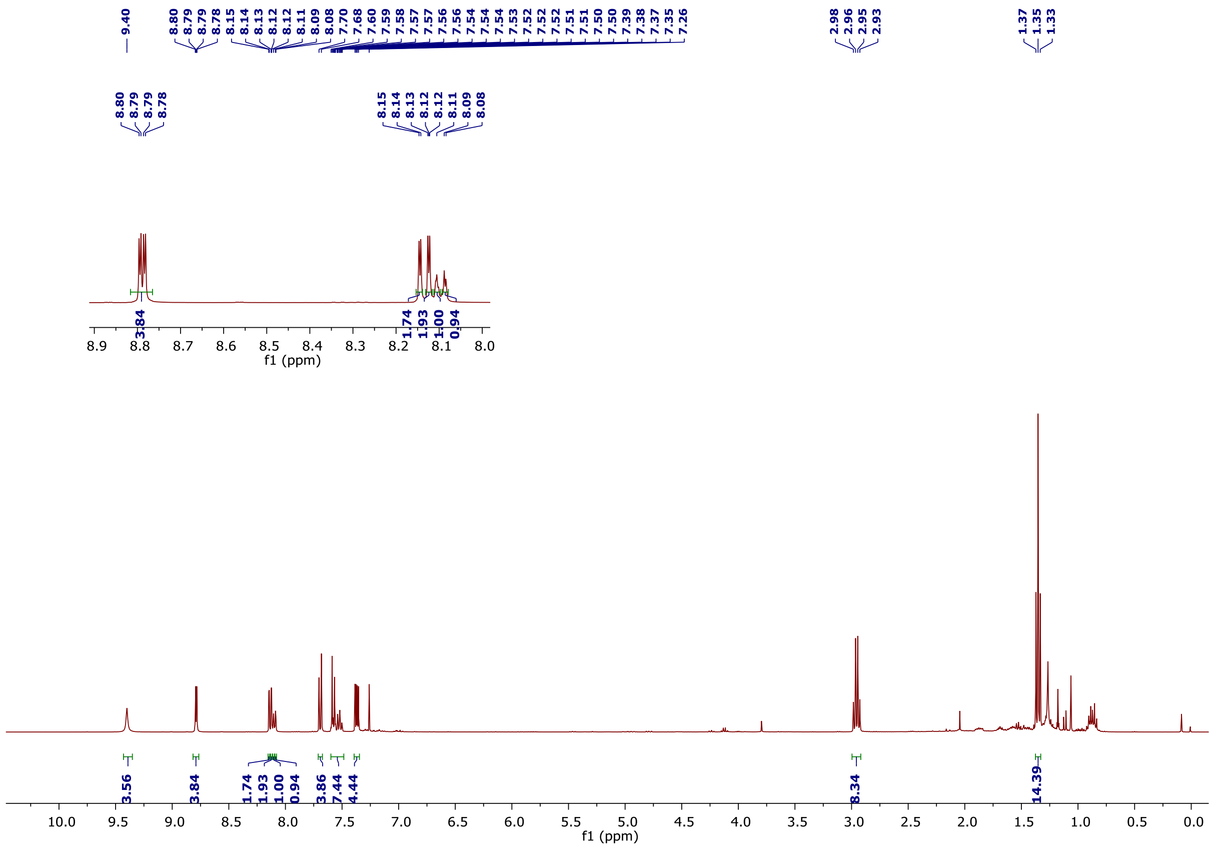

3. Effect of Leaving Groups:

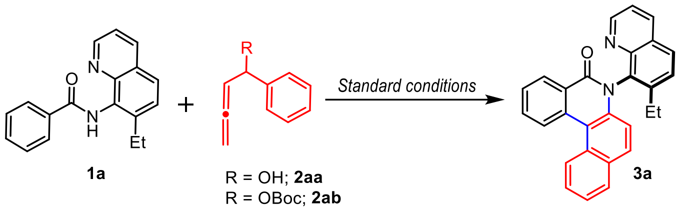

Entry	Coupling partner (1.5 equiv)	Yield of **3a**	ee of **3a**
1	**2aa**	54%	94% ee
2	**2ab**	73%	94% ee

Entry 1: A 15 mL dry reaction tube equipped with a magnetic stir bar was charged with benzamide **1a** (0.1 mmol), **2aa** (0.15 mmol), $Co(OAc)_2 \cdot 4H_2O$ (15 mol%), **L4** (25 mol%), Eosin Y (5 mol%), and $NaOPiv \cdot H_2O$ (1.2 equiv). Anhydrous TFE solvent (1.0 mL) was added via syringe. The reaction system was sealed with a stopper and equipped with an O_2 balloon to maintain a constant oxygen supply. The mixture was then irradiated with 427 nm Kessil light and stirred for 40 h. Upon completion, the reaction mixture was diluted with CH_2Cl_2 (20 mL), filtered through a pad of Celite, and concentrated under reduced pressure. The resulting crude product was purified by column chromatography using a petroleum ether/ethyl acetate (PE/EA) gradient to obtain the pure chiral product **3a**.

Entry 2: A 15 mL dry reaction tube equipped with a magnetic stir bar was charged with benzamide **1a** (0.1 mmol), **2ab** (0.15 mmol), $Co(OAc)_2 \cdot 4H_2O$ (15 mol%), **L4** (25 mol%), Eosin Y (5 mol%), and $NaOPiv \cdot H_2O$ (1.2 equiv). Anhydrous TFE solvent (1.0 mL) was added via syringe. The reaction system was sealed with a stopper and equipped with an O_2 balloon to maintain a constant oxygen supply. The mixture was then irradiated with 427 nm Kessil light and stirred for 40 h. Upon completion, the reaction mixture was diluted with CH_2Cl_2 (20 mL), filtered through a pad of Celite, and concentrated under reduced pressure. The resulting crude product was purified by column chromatography using a petroleum ether/ethyl acetate (PE/EA) gradient to obtain the pure chiral product **3a**.

4. Radical inhibiting experiments:

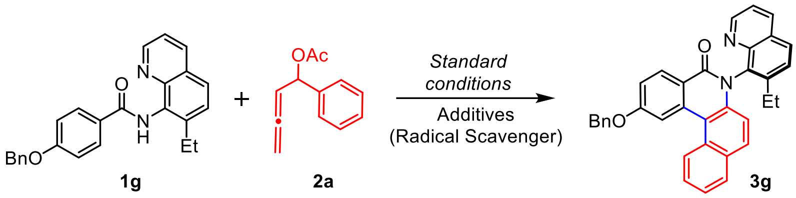

Entry	Additives (5.0 equiv)	Yield of **3g**	ee of **3g**
1	TEMPO	14%	98% ee
2	BHT	30%	94% ee

A 15 mL dry reaction tube equipped with a magnetic stir bar was charged with benzamide **1g** (0.1 mmol), α-aryl-allenyl acetate **2a** (0.15 mmol), $Co(OAc)_2{\cdot}4H_2O$ (15 mol%), **L4** (25 mol%), Eosin Y (5 mol%), additive TEMPO or BHT (5.0 equiv) and NaOPiv·H_2O (1.2 equiv). Anhydrous TFE solvent (1.0 mL) was added via syringe. The reaction system was sealed with a stopper and equipped with an O_2 balloon to maintain a constant oxygen supply. The mixture was then irradiated with 427 nm Kessil light and stirred for 40 h. Upon completion, the reaction mixture was diluted with CH_2Cl_2 (10 mL), filtered through a pad of Celite, and concentrated under reduced pressure. The resulting crude product was purified by column chromatography using a petroleum ether/ethyl acetate (PE/EA) gradient to obtain the pure chiral product.

5. Nonlinear effect study: We investigated the relation between the enantiomeric excess of product **3a** with the enantiopurity of the chiral ligand **L4**. To perform this study, we prepared (*S*)-**L4**, and mixed with (*R*)-**L4** to afford 100%, 80%, 60%, 40%, 20% and 0% ee of the ligand and executed reactions following the general procedure (GP-A). We obtained a linear correlation between enantiomeric excess (ee) of product **3a** and the enantiopurity of chiral ligand **L4**.

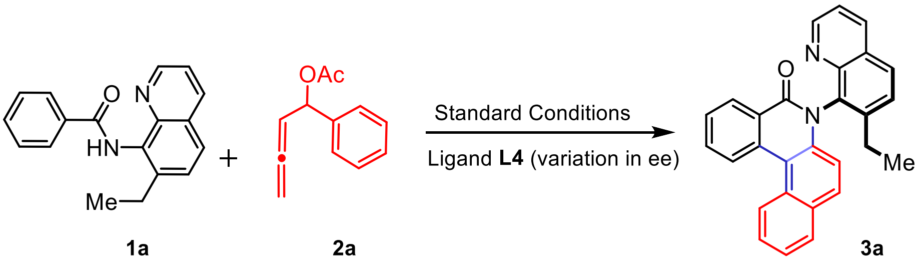

Ligand ee (%)	0	20	40	60	80	100
Product ee (%)	0	23	38	60	74	94

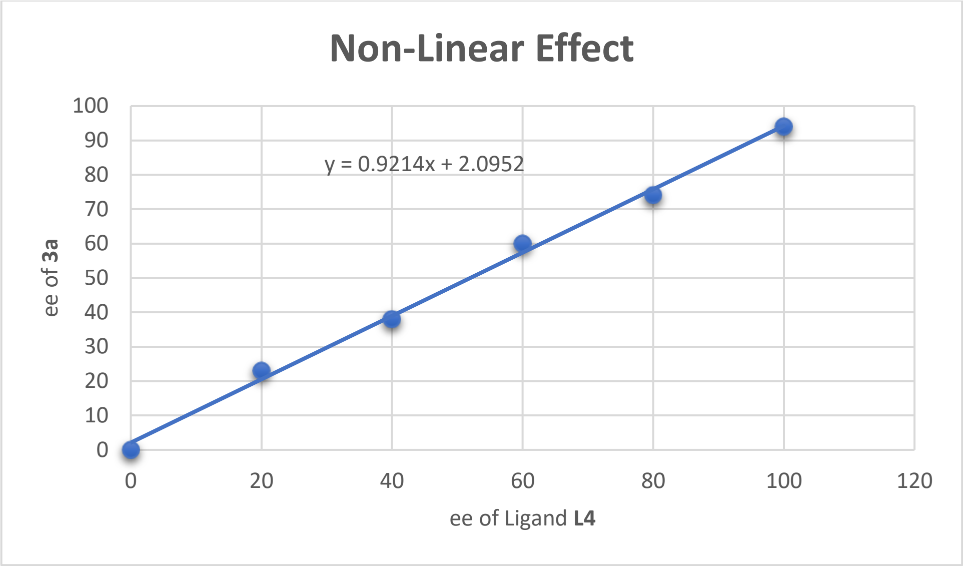

Study for the non-linear effect

Crystallographic experimental data

Crystallization: Crystals of compound **3v** was obtained through slow evaporation technique at room temperature from the solution in an isopropanol/hexane mixture. Crystal structure of compound **3v** (CCDC number: 2383470, Ellipsoid Probability 50%).

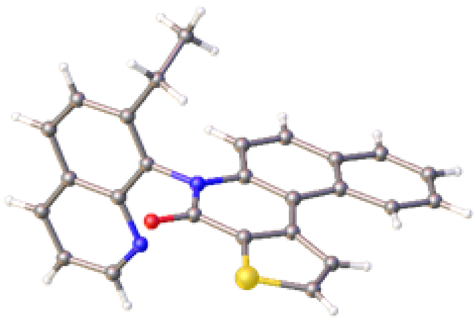

Table 1. Crystal data and structure refinement for **3v**.

**Identification code**	**3v**
**Empirical formula**	C26 H18 N2 O S
**Formula weight**	406.48
**Temperature**	297(2) K
**Wavelength**	0.71073 A
**Crystal system, space group**	Orthorhombic, P 21 21 21
**Unit cell dimensions**	a = 9.2320(7) A alpha = 90 deg.
	b = 9.3771(6) A beta = 90 deg.
	c = 23.9503(17) A gamma = 90 deg.
**Volume**	2073.4(3) A^3
**Z, Calculated density**	4, 1.302 Mg/m^3
**Absorption coefficient**	0.176 mm^-1
**F(000)**	848
**Crystal size**	0.328 x 0.167 x 0.115 mm
**Theta range for data collection**	3.211 to 27.116 deg.
**Limiting indices**	-11<=h<=11, -12<=k<=12, -30<=l<=30
**Reflections collected / unique**	49411 / 4555 [R(int) = 0.0490]
**Completeness to theta** = 25.242	99.7 %
**Absorption correction**	Semi-empirical from equivalents

**Max. and min. transmission**	0.7455 and 0.5165
**Refinement method**	Full-matrix least-squares on F^2
**Data / restraints / parameters**	4555 / 0 / 272
**Goodness-of-fit on F^2**	1.097
**Final R indices [I>2sigma(I)]**	R1 = 0.0396, wR2 = 0.1087
**R indices (all data)**	R1 = 0.0422, wR2 = 0.1110
**Absolute structure paramete**r	0.00(2)
**Extinction coefficient**	n/a
**Largest diff. peak and hole**	0.181 and -0.292 e.A^-3

Characterization of Products

(*R*)-6-(7-ethylquinolin-8-yl)benzo[*a*]phenanthridin-5(6*H*)-one (3a):

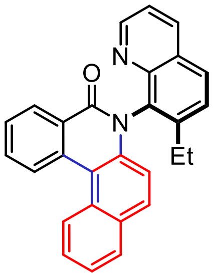

Synthesized according to general procedure GP-A as a brownish solid, eluent (PE:EtOAc = 3:1, v/v), Yield: 81% (64.8 mg), 94% ee, m.p.: 90°C - 92°C.

$[\alpha]_D^{20}$ = -107.066 (c = 0.2, $CHCl_3$)

^{1}H NMR (400 MHz, $CDCl_3$) δ 8.93 (d, *J* = 8.7 Hz, 1H), 8.85 (d, *J* = 8.3 Hz, 1H), 8.72 (s, 1H), 8.68 (d, *J* = 7.9 Hz, 1H), 8.25 (d, *J* = 8.1 Hz, 1H), 8.02 (d, *J* = 8.5 Hz, 1H), 7.88 (t, *J* = 7.8 Hz, 1H), 7.83 (d, *J* = 8.0 Hz, 1H), 7.72 (d, *J* = 8.5 Hz, 1H), 7.66 (t, *J* = 7.4 Hz, 2H), 7.58 (d, *J* = 9.0 Hz, 1H), 7.49 (t, *J* = 7.4 Hz, 1H), 7.37 (dd, *J* = 8.2, 3.9 Hz, 1H), 6.62 (d, *J* = 9.0 Hz, 1H), 2.67 – 2.53 (m, 2H), 1.16 (t, *J* = 7.5 Hz, 3H) ppm.

^{13}C NMR (126 MHz, $CDCl_3$) δ 161.8, 151.4, 145.0, 144.1, 137.9, 136.1, 135.0, 133.4, 132.1, 130.8, 130.4, 130.0, 129.3, 129.1, 128.8, 128.2, 128.0, 127.45, 127.40, 127.31, 127.28, 126.3, 124.7, 121.3, 116.7, 113.9, 25.0, 13.9 ppm.

HRMS (ESI) m/z: $[M+H^+]^+$ Calculated for $[C_{28}H_{21}N_2O^+]$: 401.1648; found: 401.1659;

HPLC Condition The enantiomeric excess was determined by Daicel Chiralpak IB, Hexanes/IPA = 70/30, 1.0 mL/min, λ = 254 nm, t (minor) = 15.6 min, t (major) = 11.3 min.

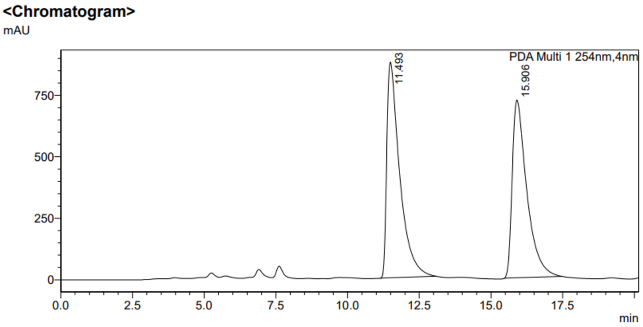

<Peak Table>

PDA Ch1 254nm

Peak#	Ret. Time	Area	Height	Area%
1	11.493	27452695	876583	51.733
2	15.906	25613838	721982	48.267
Total		53066533	1598565	100.000

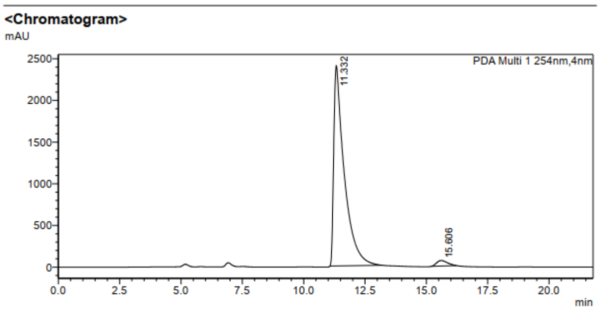

<Peak Table>

PDA Ch1 254nm

Peak#	Ret. Time	Area	Height	Area%
1	11.332	71619811	2402234	97.277
2	15.606	2004482	65671	2.723
Total		73624293	2467905	100.000

(*R*)-6-(7-ethylquinolin-8-yl)-2-methylbenzo[*a*]phenanthridin-5(6*H*)-one (3b):

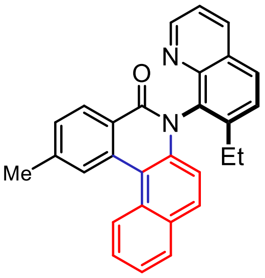

Synthesized according to general procedure GP-A as brownish sticky liquid, eluent (PE:EtOAc = 3:1 v/v), Yield: 83% (68.7 mg), 96% ee.

$[\alpha]_D^{20}$ = -122.062 (c = 0.1, $CHCl_3$)

^{1}H NMR (400 MHz, $CDCl_3$) δ 8.94 (d, *J* = 8.7 Hz, 1H), 8.70 (d, *J* = 4.0 Hz, 1H), 8.65 (s, 1H), 8.58 (d, *J* = 8.1 Hz, 1H), 8.23 (d, *J* = 8.3 Hz, 1H), 8.00 (d, *J* = 8.5 Hz, 1H), 7.83 (d, *J* = 8.0 Hz, 1H), 7.72– 7.65 (m, 2H), 7.57 (d, *J* = 9.1 Hz, 1H), 7.49 (d, *J* = 6.3 Hz, 2H), 7.35 (dd, *J* = 8.3, 4.1 Hz, 1H), 6.61 (d, *J* = 9.1 Hz, 1H), 2.66 – 2.53 (m, 5H), 1.16 (t, *J* = 7.6 Hz, 3H) ppm.

^{13}C NMR (101 MHz, $CDCl_3$) δ 161.8, 151.4, 144.8, 144.1, 142.5, 137.8, 136.2, 135.1, 133.3, 130.7, 130.3, 129.8, 129.3, 129.1, 128.8, 128.7, 128.1, 128.0, 127.4, 127.2, 126.3, 125.0, 124.6, 121.3, 116.7, 113.9, 24.9, 22.5, 14.0 ppm.

HRMS (ESI) m/z: $[M+H^+]^+$ Calculated for $[C_{29}H_{23}N_2O^+]$: 415.1805; found: 415.1815;

HPLC Condition The enantiomeric excess was determined by Daicel Chiralpak IB, Hexanes/IPA = 70/30, 1.0 mL/min, λ = 254 nm, t (minor) = 19.4 min, t (major) = 11.4 min.

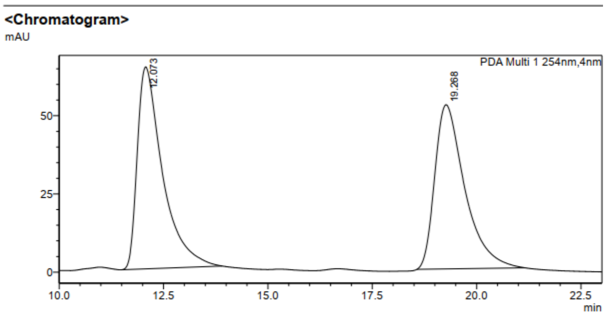

<Peak Table>

PDA Ch1 254nm

Peak#	Ret. Time	Area	Height	Area%
1	12.073	2715452	64553	50.874
2	19.268	2622162	52482	49.126
Total		5337614	117036	100.000

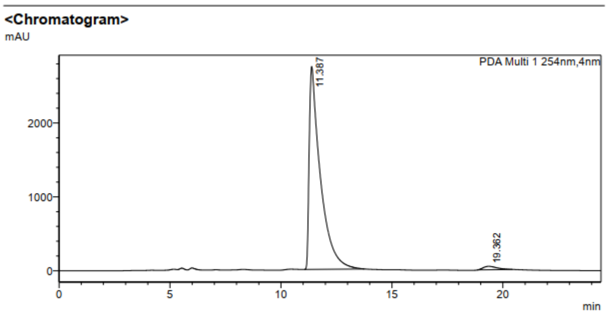

<Peak Table>

PDA Ch1 254nm

Peak#	Ret. Time	Area	Height	Area%
1	11.387	96137819	2744346	97.922
2	19.362	2039645	47084	2.078
Total		98177463	2791430	100.000

(*R*)-2-ethyl-6-(7-ethylquinolin-8-yl)benzo[*a*]phenanthridin-5(6*H*)-one (3c):

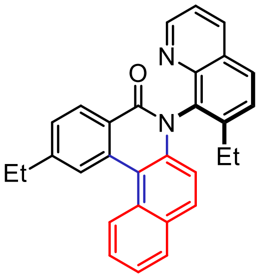

Synthesized according to general procedure GP-A as yellowish sticky liquid, eluent (PE:EtOAc = 3:1 v/v), Yield: 70% (59.9 mg), 96% ee.

$[\alpha]_D^{20}$ = -427.023 (c = 0.1, $CHCl_3$)

^{1}H NMR (400 MHz, $CDCl_3$) δ 8.90 (d, *J* = 8.8 Hz, 1H), 8.76 (s, 1H), 8.65 (s, 1H), 8.60 (d, *J* = 8.2 Hz, 1H), 8.25 (d, *J* = 8.2 Hz, 1H), 8.01 (d, *J* = 8.5 Hz, 1H), 7.83 (d, *J* = 8.1 Hz, 1H), 7.73 – 7.63 (m, 2H), 7.58 – 7.48 (m, 2H), 7.39 – 7.36 (m, 1H), 7.17 (d, *J* = 8.1 Hz, 1H), 6.61 (d, *J* = 9.0 Hz, 1H), 2.95 (q, *J* = 7.8 Hz, 2H), 2.70 – 2.56 (m, 2H), 1.43 (t, *J* = 7.7 Hz, 3H), 1.15 (t, *J* = 7.8 Hz, 3H) ppm.

^{13}C NMR (101 MHz, $CDCl_3$) δ 161.9, 151.3, 148.7, 144.7, 144.3, 137.8, 136.5, 135.1, 133.2, 130.8, 130.2, 129.8, 129.3, 129.1, 128.8, 128.1, 127.8, 127.6, 127.2, 126.4, 126.3, 125.2, 124.6, 121.3, 116.7, 114.1, 29.7, 24.9, 15.7, 13.9 ppm.

HRMS (ESI) m/z: $[M+H^+]^+$ Calculated for $[C_{30}H_{25}N_2O^+]$: 429.1961; found: 429.1964;

HPLC Condition The enantiomeric excess was determined by Daicel Chiralpak IB, Hexanes/IPA = 70/30, 1.0 mL/min, λ = 254 nm, t (minor) = 16.2 min, t (major) = 10.6 min.

<Chromatogram>

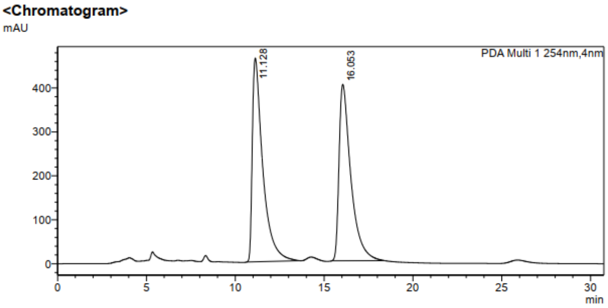

<Peak Table>

PDA Ch1 254nm

Peak#	Ret. Time	Area	Height	Area%
1	11.128	18996237	463217	50.579
2	16.053	18561335	401517	49.421
Total		37557572	864735	100.000

<Chromatogram>

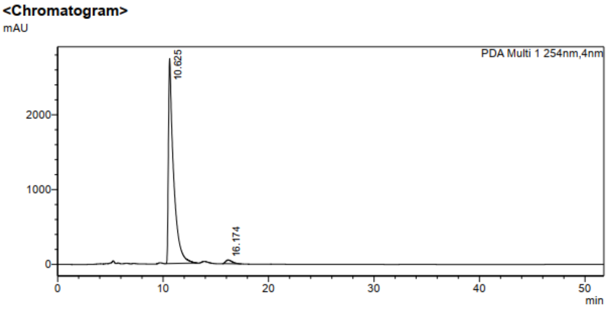

<Peak Table>

PDA Ch1 254nm

Peak#	Ret. Time	Area	Height	Area%
1	10.625	92096354	2744212	97.741
2	16.174	2128929	49057	2.259
Total		94225284	2793270	100.000

(*R*)-6-(7-ethylquinolin-8-yl)-2-isopropylbenzo[*a*]phenanthridin-5(6*H*)-one (3d):

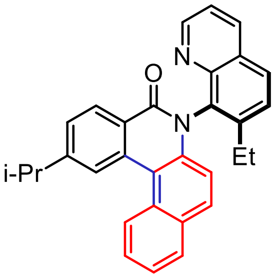

Synthesized according to general procedure GP-A as yellowish sticky liquid, eluent (PE:EtOAc = 3:1 v/v), Yield: 84% (74.3 mg), 96% ee.

$[\alpha]_D^{20}$ = -252.154 (c = 0.1, $CHCl_3$)

^{1}H NMR (400 MHz, $CDCl_3$) δ 8.91 (d, *J* = 8.8 Hz, 1H), 8.72 (s, 1H), 8.68 (s, 1H), 8.59 (d, *J* = 8.2 Hz, 1H), 8.25 (d, *J* = 8.2 Hz, 1H), 8.01 (d, *J* = 8.6 Hz, 1H), 7.83 (d, *J* = 8.0 Hz, 1H), 7.72 (d, *J* = 8.4 Hz, 1H), 7.66 (t, *J* = 7.8 Hz, 1H), 7.55 (t, *J* = 9.7 Hz, 2H), 7.49 (t, *J* = 7.3 Hz, 1H), 7.40 – 7.35 (m, 1H), 6.60 (d, *J* = 9.0 Hz, 1H), 3.25 – 3.18 (m, 1H), 2.66 – 2.52 (m, 2H), 1.44 (d, *J* = 6.9 Hz, 6H), 1.15 (t, *J* = 7.7 Hz, 3H) ppm.

^{13}C NMR (101 MHz, $CDCl_3$) δ 161.8, 153.2, 151.4, 144.9, 144.2, 137.9, 136.2, 135.1, 133.4, 130.8, 130.4, 129.8, 129.4, 129.1, 128.8, 128.1, 128.0, 127.1, 126.3, 126.2, 125.3, 125.1, 124.6, 121.3, 116.8, 114.2, 34.9, 24.9, 24.09, 24.11, 13.9 ppm.

HRMS (ESI) m/z: $[M+H^+]^+$ Calculated for $[C_{31}H_{27}N_2O^+]$: 443.2118; found: 443.2122;

HPLC Condition The enantiomeric excess was determined by Daicel Chiralpak IB, Hexanes/IPA = 70/30, 1.0 mL/min, λ = 254 nm, t (minor) = 13.9 min, t (major) = 9.0

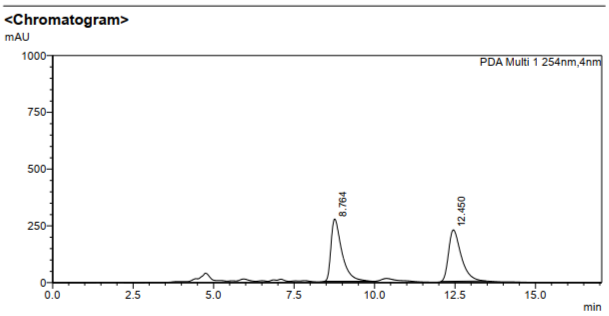

<Peak Table>

PDA Ch1 254nm

Peak#	Ret. Time	Area	Height	Area%
1	8.764	6448908	273834	51.204
2	12.450	6145649	226660	48.796
Total		12594557	500495	100.000

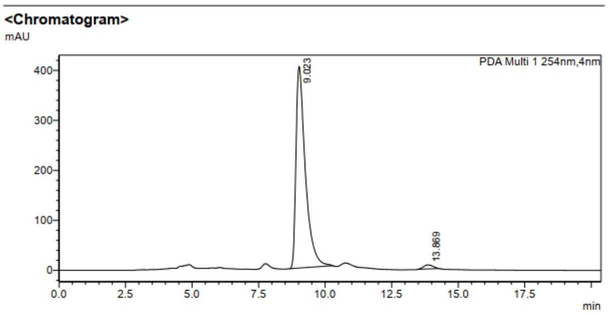

<Peak Table>

PDA Ch1 254nm

Peak#	Ret. Time	Area	Height	Area%
1	9.023	9874749	403030	98.029
2	13.869	198512	8011	1.971
Total		10073261	411041	100.000

(R)-2-(tert-butyl)-6-(7-ethylquinolin-8-yl)benzo[a]phenanthridin-5(6H)-one (3e):

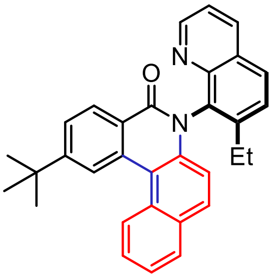

Synthesized according to general procedure GP-A as yellowish sticky liquid, eluent (PE:EtOAc = 3:1 v/v), Yield: 82% (74.8 mg), 96% ee.

$[\alpha]_D^{20}$ = -111.253 (c = 0.1, $CHCl_3$)

^{1}H NMR (400 MHz, $CDCl_3$) δ 8.92 (d, J = 8.7 Hz, 1H), 8.85 (s, 1H), 8.70 (d, J = 4.1 Hz, 1H), 8.60 (d, J = 8.4 Hz, 1H), 8.22 (d, J = 8.2 Hz, 1H), 8.00 (d, J = 8.5 Hz, 1H), 7.84 (d, J = 8.0 Hz, 1H), 7.71 (d, J = 8.5 Hz, 2H), 7.69-7.65 (m, 1H), 7.57 (d, J = 9.0 Hz, 1H), 7.49 (t, J = 7.4 Hz, 1H), 7.35 (dd, J = 8.3, 4.2 Hz, 1H), 6.61 (d, J = 9.1 Hz, 1H), 2.67 – 2.53 (m, 2H), 1.53 (s, 9H), 1.16 (t, J = 7.6 Hz, 3H) ppm.

^{13}C NMR (101 MHz, $CDCl_3$) δ 161.8, 155.3, 151.4, 144.9, 144.2, 137.9, 136.1, 134.7, 133.4, 130.8, 130.4, 129.7, 129.1, 129.0, 128.9, 128.1, 128.0, 127.1, 126.2, 125.2, 124.9, 124.6, 124.1, 121.3, 116.8, 114.4, 35.7, 31.5, 24.9, 14.0 ppm.

HRMS (ESI) m/z: $[M+H^+]^+$ Calculated for $[C_{32}H_{29}N_2O^+]$: 457.2274; found: 457.2280;

HPLC Condition The enantiomeric excess was determined by Daicel Chiralpak IB, Hexanes/IPA = 70/30, 1.0 mL/min, λ = 254 nm, t (minor) = 11.3 min, t (major) = 8.8 min.

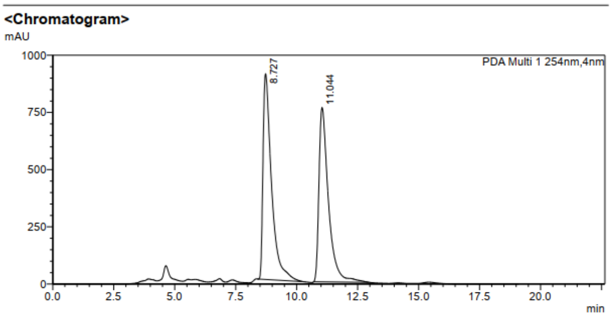

<Peak Table>

PDA Ch1 254nm

Peak#	Ret. Time	Area	Height	Area%
1	8.727	21909076	898775	51.486
2	11.044	20644778	761407	48.514
Total		42553854	1660182	100.000

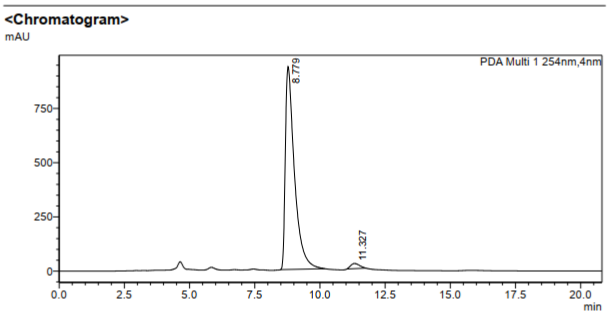

<Peak Table>

PDA Ch1 254nm

Peak#	Ret. Time	Area	Height	Area%
1	8.779	22780033	935442	97.578
2	11.327	565429	23933	2.422
Total		23345462	959375	100.000

(*R*)-6-(7-ethylquinolin-8-yl)-2-methoxybenzo[*a*]phenanthridin-5(6*H*)-one (3f):

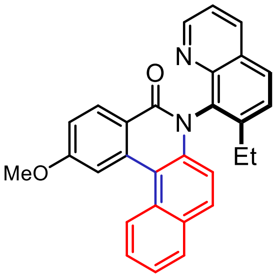

Synthesized according to general procedure GP-A as yellowish solid, eluent (PE:EtOAc = 2:1 v/v), Yield: 84% (72.3 mg), 90% ee, m.p. 95˚C-97˚C

$[\alpha]_D^{20}$ = -232.955 (c = 0.1, $CHCl_3$)

^{1}H NMR (400 MHz, $CDCl_3$) δ 8.97 (d, *J* = 8.8 Hz, 1H), 8.71 (s, 1H), 8.62 (d, *J* = 8.8 Hz, 1H), 8.30 (s, 1H), 8.23 (d, *J* = 8.1 Hz, 1H), 8.00 (d, *J* = 8.5 Hz, 1H), 7.83 (d, *J* = 8.1 Hz, 1H), 7.71 (d, *J* = 8.5 Hz, 1H), 7.65 (t, *J* = 7.9 Hz, 1H), 7.57 (d, *J* = 8.9 Hz, 1H), 7.48 (t, *J* = 7.5 Hz, 1H), δ 7.37 – 7.34 (m, 1H), 7.23 (d, *J* = 8.8 Hz, 1H), 6.60 (d, *J* = 8.8 Hz, 1H), 4.04 (s, 3H), 2.67 – 2.55 (m, 2H), 1.16 (t, *J* = 7.7 Hz, 3H) ppm.

^{13}C NMR (101 MHz, $CDCl_3$) δ 162.8, 161.6, 151.3, 144.8, 144.2, 138.3, 136.8, 136.3, 133.3, 131.4, 130.7, 130.5, 130.2, 129.1, 128.9, 128.1, 128.0, 127.3, 125.9, 124.6, 121.3, 120.9, 116.8, 114.8, 113.7, 111.0, 55.8, 24.9, 14.0 ppm.

HRMS (ESI) m/z: $[M+H^+]^+$ Calculated for $[C_{29}H_{23}N_2O_2^+]$: 431.1754; found: 431.1769;

HPLC Condition The enantiomeric excess was determined by Daicel Chiralpak IB, Hexanes/IPA = 70/30, 1.0 mL/min, λ = 254 nm, t (minor) = 25.4 min, t (major) = 17.0 min.

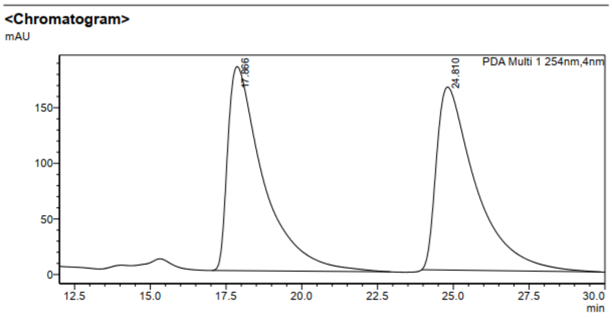

<Peak Table>

PDA Ch1 254nm

Peak#	Ret. Time	Area	Height	Area%
1	17.866	15002552	183503	50.130
2	24.810	14924716	164589	49.870
Total		29927267	348092	100.000

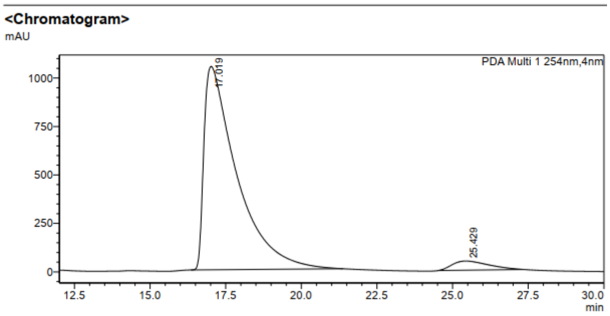

<Peak Table>

PDA Ch1 254nm

Peak#	Ret. Time	Area	Height	Area%
1	17.019	79218972	1048825	95.204
2	25.429	3990780	47539	4.796
Total		83209752	1096364	100.000

(*R*)-2-(benzyloxy)-6-(7-ethylquinolin-8-yl)benzo[*a*]phenanthridin-5(6*H*)-one (3g):

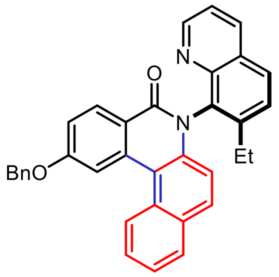

Synthesized according to general procedure GP-A as brownish sticky liquid, eluent (PE:EtOAc = 2:1 v/v), Yield: 78% (78.9 mg), 98% ee.

$[\alpha]_D^{20}$ = -110.450 (c = 0.1, $CHCl_3$)

^{1}H NMR (500 MHz, $CDCl_3$) δ 8.71 (s, 1H), 8.63 (t, *J* = 10.6 Hz, 2H), 8.28 (s, 1H), 8.22 (d, *J* = 8.0 Hz, 1H), 7.99 (d, *J* = 8.2 Hz, 1H), 7.80 (d, *J* = 7.6 Hz, 1H), 7.70 (d, *J* = 8.4 Hz, 1H), 7.55 (d, *J* = 8.2 Hz, 2H), 7.52 – 7.45 (m, 3H), 7.42 – 7.33 (m, 5H), 6.59 (d, *J* = 8.9 Hz, 1H), 5.33 (s, 2H), 2.66 – 2.54 (m, 2H), 1.15 (t, *J* = 7.2 Hz, 3H) ppm.

^{13}C NMR (126 MHz, $CDCl_3$) δ 161.8, 161.6, 151.4, 144.9, 144.2, 138.3, 136.8, 136.6, 136.2, 133.4, 131.4, 130.7, 130.4, 130.2, 129.1, 129.0, 128.8, 128.3, 128.1, 128.0, 127.5, 127.4, 125.8, 124.6, 121.3, 121.1, 116.8, 116.1, 113.6, 111.6, 70.3, 24.9, 13.9 ppm.

HRMS (ESI) m/z: $[M+H^+]^+$ Calculated for $[C_{35}H_{27}N_2O_2^+]$: 507.2067; found: 507.2069;

HPLC Condition The enantiomeric excess was determined by Daicel Chiralpak IB, Hexanes/IPA = 70/30, 1.0 mL/min, λ = 254 nm, t (minor) = 24.4 min, t (major) = 14.6 min.

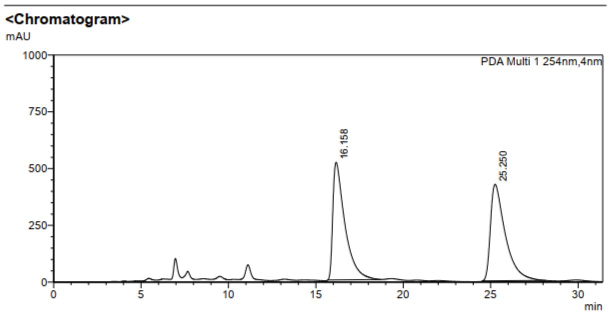

<Peak Table>

PDA Ch1 254nm

Peak#	Ret. Time	Area	Height	Area%
1	16.158	24948527	518585	49.788
2	25.250	25161335	424938	50.212
Total		50109861	943522	100.000

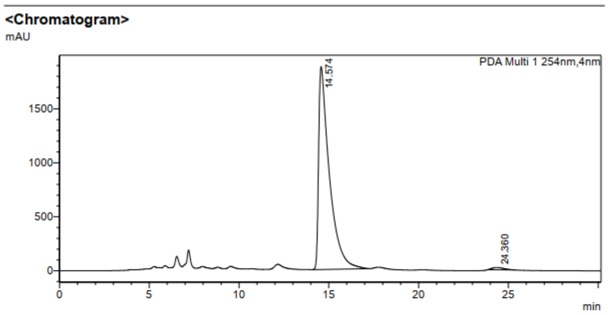

<Peak Table>

PDA Ch1 254nm

Peak#	Ret. Time	Area	Height	Area%
1	14.574	78048953	1878959	98.730
2	24.360	1003585	21160	1.270
Total		79052539	1900119	100.000

(*R*)-6-(7-ethylquinolin-8-yl)-2-phenoxybenzo[*a*]phenanthridin-5(6*H*)-one (3h):

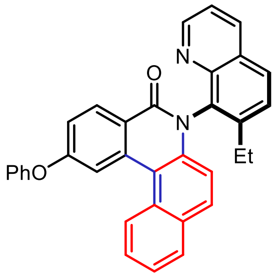

Synthesized according to general procedure GP-A as yellowish solid, eluent (PE:EtOAc = 3:1 v/v), Yield: 83% (81.7 mg), 94% ee, m.p. 82 ℃ - 84 ℃.

$[\alpha]_D^{20}$ = -113.260 (c = 0.1, $CHCl_3$)

^{1}H NMR (500 MHz, $CDCl_3$) δ 8.73 (dd, *J* = 4.2, 1.7 Hz, 1H), 8.70 (d, *J* = 8.7 Hz, 1H), 8.64 (d, *J* = 8.8 Hz, 1H), 8.33 (d, *J* = 2.2 Hz, 1H), 8.23 (dd, *J* = 8.3, 1.6 Hz, 1H), 8.01 (d, *J* = 8.5 Hz, 1H), 7.79 (d, *J* = 8.0 Hz, 1H), 7.71 (d, *J* = 8.6 Hz, 1H), 7.56 (d, *J* = 9.1 Hz, 1H), 7.53 – 7.41 (m, 5H), 7.36 (dd, *J* = 8.3, 4.2 Hz, 1H), 7.28 (dd, *J* = 8.8, 2.3 Hz, 1H), 7.24 – 7.22 (m, 2H), 6.59 (d, *J* = 9.1 Hz, 1H), 2.69 – 2.61 (m, 1H), 2.60 – 2.53 (m, 1H), 1.16 (t, *J* = 7.6 Hz, 3H) ppm.

^{13}C NMR (126 MHz, $CDCl_3$) δ 161.5, 156.1, 151.4, 144.8, 144.2, 138.4, 136.9, 136.3, 133.2, 132.0, 131.6, 130.6, 130.4, 130.3, 129.1, 128.8, 128.1, 128.0, 127.3, 125.7, 124.7 (2xC), 122.3, 121.3, 120.3, 120.1, 117.7, 116.7, 115.2, 113.4, 24.9, 13.9 ppm.

HRMS (ESI) m/z: $[M+H^+]^+$ Calculated for $[C_{34}H_{25}N_2O_2^+]$: 493.1911; found: 493.1923;

HPLC Condition The enantiomeric excess was determined by Daicel Chiralpak IB, Hexanes/IPA = 70/30, 1.0 mL/min, λ = 254 nm, t (minor) = 20.8 min, t (major) = 11.8 min.

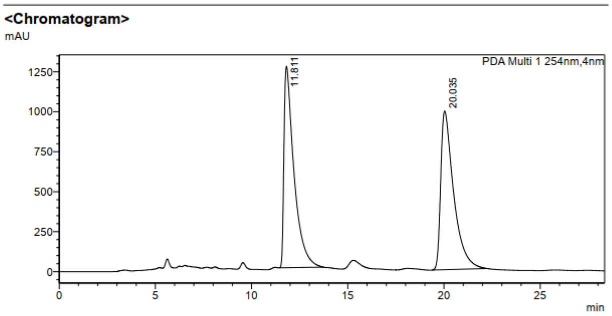

<Peak Table>
PDA Ch1 254nm

Peak#	Ret. Time	Area	Height	Area%
1	11.811	45577519	1259548	49.610
2	20.035	46294862	992531	50.390
Total		91872382	2252079	100.000

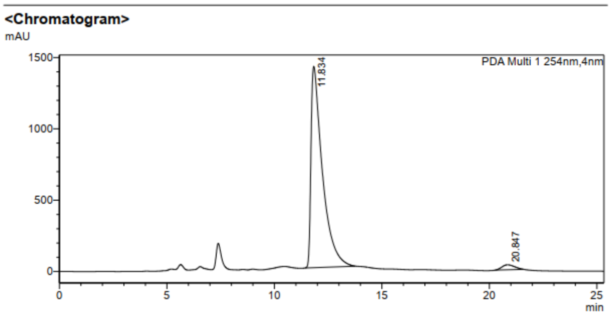

<Peak Table>
PDA Ch1 254nm

Peak#	Ret. Time	Area	Height	Area%
1	11.834	52014883	1410349	97.322
2	20.847	1431563	34959	2.678
Total		53446446	1445307	100.000

(*R*)-6-(7-ethylquinolin-8-yl)-2-(methylthio)benzo[*a*]phenanthridin-5(6*H*)-one (3i):

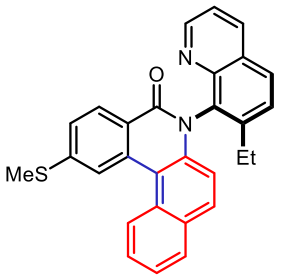

Synthesized according to general procedure GP-A as brownish solid, eluent (PE:EtOAc = 2:1 v/v), Yield: 82% (73.2 mg), 85% ee, m.p. 88˚C - 90˚C

$[\alpha]_D^{20}$ = -86.442 (c = 0.1, $CHCl_3$)

^{1}H NMR (500 MHz, $CDCl_3$) δ 8.88 (d, *J* = 8.5 Hz, 1H), 8.73 (s, 1H), 8.63 (s, 1H), 8.57 (d, *J* = 8.3 Hz, 1H), 8.23 (d, *J* = 8.1 Hz, 1H), 8.00 (d, *J* = 8.5 Hz, 1H), 7.83 (d, *J* = 7.8 Hz, 1H), 7.71 (d, *J* = 8.5 Hz, 1H), 7.65 (t, *J* = 7.1 Hz, 1H), 7.58 (d, *J* = 8.9 Hz, 1H), 7.51 – 7.47 (m, 2H), 7.37 – 7.35 (m, 1H), 6.60 (d, *J* = 9.0 Hz, 1H), 2.67 (s, 3H), 2.65 – 2.55 (m, 2H), 1.16 (t, *J* = 7.4 Hz, 3H) ppm.

^{13}C NMR (126 MHz, $CDCl_3$) δ 161.6, 151.4, 144.8, 144.5, 144.2, 138.3, 136.3, 135.4, 133.2, 130.7, 130.3 (2xC), 129.6, 129.1, 128.9, 128.1, 128.0, 127.4, 125.9, 124.8, 124.7, 124.1, 123.4, 121.3, 116.7, 113.4, 24.9, 15.4, 13.9 ppm.

HRMS (ESI) m/z: $[M+H^+]^+$ Calculated for $[C_{29}H_{23}N_2OS^+]$: 447.1526; found: 447.1531;

HPLC Condition The enantiomeric excess was determined by Daicel Chiralpak IB, Hexanes/IPA = 70/30, 1.0 mL/min, λ = 254 nm, t (minor) = 28.6 min, t (major) = 14.8 min.

<Chromatogram>

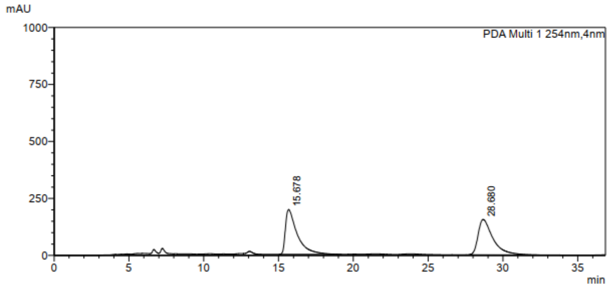

<Peak Table>

PDA Ch1 254nm

Peak#	Ret. Time	Area	Height	Area%
1	15.678	10807341	196310	50.269
2	28.680	10691846	155199	49.731
Total		21499188	351509	100.000

<Chromatogram>

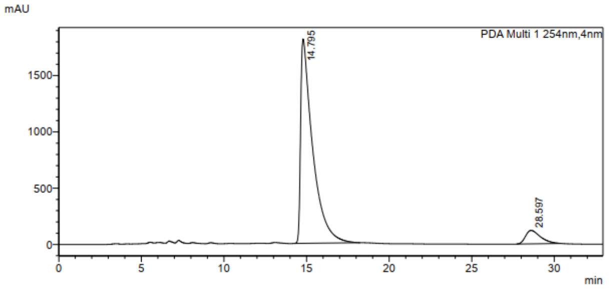

<Peak Table>

PDA Ch1 254nm

Peak#	Ret. Time	Area	Height	Area%
1	14.795	91787052	1815096	92.408
2	28.597	7540858	119735	7.592
Total		99327910	1934831	100.000

(*R*)-6-(7-ethylquinolin-8-yl)-2-fluorobenzo[*a*]phenanthridin-5(6*H*)-one (3j):

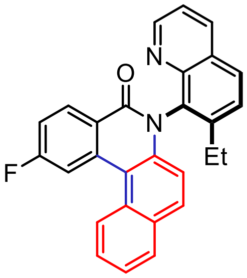

Synthesized according to general procedure GP-A as yellowish solid, eluent (PE:EtOAc = 3:1 v/v), Yield: 69% (57.7 mg), 90% ee, m.p. 109 ℃ - 111 ℃

$[\alpha]_D^{20}$ = -163.674 (c = 0.1, $CHCl_3$)

^{1}H NMR (500 MHz, $CDCl_3$) δ 8.87 (d, *J* = 8.7 Hz, 1H), 8.70 – 8.67 (m, 2H), 8.53 – 8.51 (m, 1H), 8.23 (d, *J* = 7.8 Hz, 1H), 8.01 (d, *J* = 8.4 Hz, 1H), 7.84 (d, *J* = 7.5 Hz, 1H), 7.73 – 7.68 (m, 2H), 7.60 (d, *J* = 7.8 Hz, 1H), 7.51 (t, *J* = 6.9 Hz, 1H), 7.37 – 7.34 (m, 2H), 6.60 (d, *J* = 9.0 Hz, 1H), 2.66 – 2.53 (m, 2H), 1.16 (t, *J* = 7.4 Hz, 3H) ppm.

^{13}C NMR (101 MHz, $CDCl_3$) δ 165.5 (d, *J* = 250.7 Hz), 161.2, 151.5, 144.8, 144.1, 138.6, 137.29 (d, *J* = 10.0 Hz), 136.1, 133.1, 132.42 (d, *J* = 10.0 Hz), 130.8, 130.7, 130.3, 129.2, 129.0, 128.1, 128.0, 127.8, 125.6, 124.9, 123.8, 121.4, 116.7, 115.5 (d, *J* = 23.0 Hz), 113.14 (d, *J* = 24.5 Hz),113.08 (d, *J* = 3.0 Hz), 24.9, 14.0 ppm.

^{19}F NMR (471 MHz, $CDCl_3$) δ -106.3.

HRMS (ESI) m/z: $[M+H^+]^+$ Calculated for $[C_{28}H_{20}FN_2O^+]$: 419.1554; found: 419.1559;

HPLC Condition The enantiomeric excess was determined by Daicel Chiralpak IB, Hexanes/IPA = 70/30, 1.0 mL/min, λ = 254 nm, t (minor) = 25.8 min, t (major) = 11.4 min.

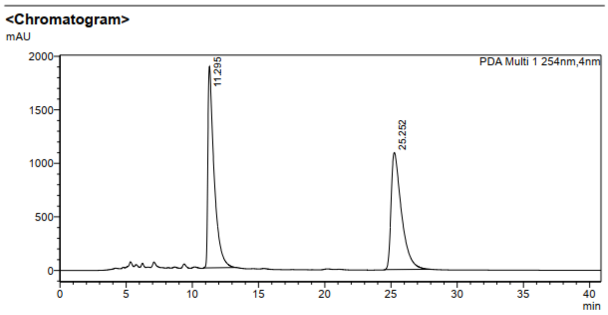

<Peak Table>
PDA Ch1 254nm

Peak#	Ret. Time	Area	Height	Area%
1	11.295	61680296	1880541	50.913
2	25.252	59469268	1092678	49.087
Total		121149564	2973219	100.000

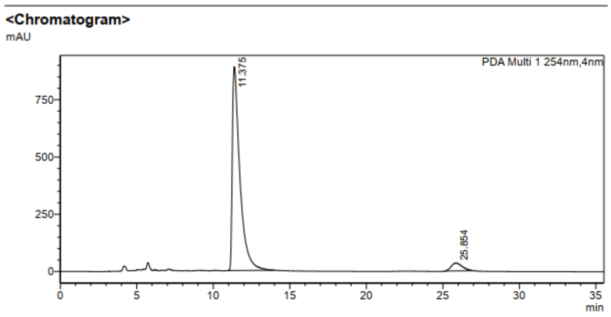

<Peak Table>
PDA Ch1 254nm

Peak#	Ret. Time	Area	Height	Area%
1	11.375	30597750	889237	94.681
2	25.854	1718777	34100	5.319
Total		32316528	923338	100.000

(*R*)-2-chloro-6-(7-ethylquinolin-8-yl)benzo[*a*]phenanthridin-5(6*H*)-one (3k):

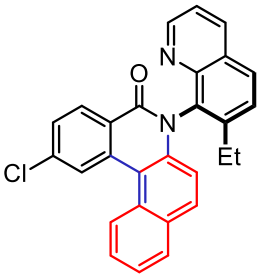

Synthesized according to general procedure GP-A as yellowish sticky liquid, eluent (PE:EtOAc = 3:1 v/v), Yield: 73% (63.4 mg), 90% ee.

$[\alpha]_D^{20}$ = -72.642 (c = 0.1, $CHCl_3$)

^{1}H NMR (400 MHz, $CDCl_3$) δ 8.85 – 8.82 (m, 2H), 8.72 (d, *J* = 4.3 Hz, 1H), 8.61 (d, *J* = 8.7 Hz, 1H), 8.24 (dd, *J* = 8.3, 1.6 Hz, 1H), 8.01 (d, *J* = 8.5 Hz, 1H), 7.83 (d, *J* = 8.0 Hz, 1H), 7.73 – 7.68 (m, 2H), 7.60 (d, *J* = 8.8 Hz, 2H), 7.52 – 7.49 (m, 1H), 7.39 – 7.35 (m, 1H), 6.61 (d, *J* = 9.1 Hz, 1H), 2.66 – 2.53 (m, 2H), 1.16 (t, *J* = 7.6 Hz, 3H) ppm.

^{13}C NMR (101 MHz, $CDCl_3$) δ 161.3, 151.5, 144.7, 144.1, 138.9, 138.5, 136.4, 136.3, 133.0, 131.0, 130.8, 130.7, 130.1, 129.3, 129.0, 128.2, 128.0, 127.9, 127.6, 126.9, 125.7, 125.6, 125.0, 121.4, 116.6, 112.9, 24.9, 14.0 ppm.

HRMS (ESI) m/z: $[M+H^+]^+$ Calculated for $[C_{28}H_{20}ClN_2O^+]$: 435.1259; found: 435.1257;

HPLC Condition The enantiomeric excess was determined by Daicel Chiralpak IB, Hexanes/IPA = 70/30, 1.0 mL/min, λ = 254 nm, t (minor) = 30.3 min, t (major) = 10.8 min.

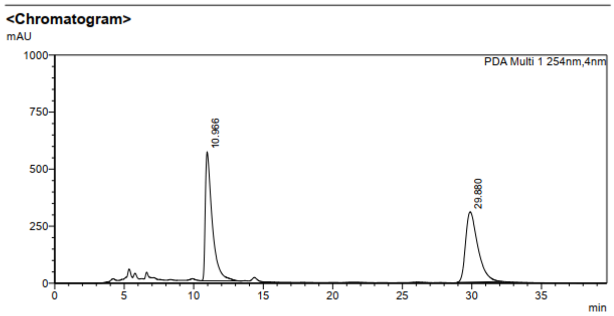

<Peak Table>

PDA Ch1 254nm

Peak#	Ret. Time	Area	Height	Area%
1	10.966	19733636	564275	51.049
2	29.880	18922584	308229	48.951
Total		38656221	872503	100.000

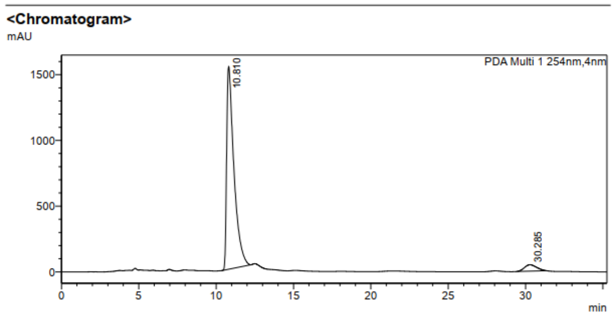

<Peak Table>

PDA Ch1 254nm

Peak#	Ret. Time	Area	Height	Area%
1	10.810	50475288	1541438	95.020
2	30.285	2645360	48447	4.980
Total		53120648	1589886	100.000

(*R*)-2-bromo-6-(7-ethylquinolin-8-yl)benzo[*a*]phenanthridin-5(6*H*)-one (3l):

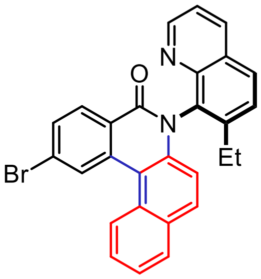

Synthesized according to general procedure GP-A as yellowish solid, eluent (PE:EtOAc = 3:1 v/v), Yield: 75% (71.7 mg), 92% ee, m.p. 102˚C-104˚C

$[\alpha]_D^{20}$ = -210.529 (c = 0.1, $CHCl_3$).

^{1}H NMR (400 MHz, $CDCl_3$) δ 9.00 (s, 1H), 8.85 (d, *J* = 8.8 Hz, 1H), 8.70 (s, 1H), 8.53 (dd, *J* = 8.5, 2.4 Hz, 1H), 8.23 (d, *J* = 8.3 Hz, 1H), 8.01 (d, *J* = 8.3 Hz, 1H), 7.84 (d, *J* = 8.2 Hz, 1H), 7.77 – 7.70 (m, 3H), 7.60 (d, *J* = 9.1 Hz, 1H), 7.51 (t, *J* = 6.8 Hz, 1H), 7.37 (dd, *J* = 8.1, 3.7 Hz, 1H), 6.60 (dd, *J* = 9.1, 2.4 Hz, 1H), 2.66 – 2.54 (m, 2H), 1.16 (t, *J* = 7.4 Hz, 3H) ppm.

^{13}C NMR (101 MHz, $CDCl_3$) δ 161.4, 151.4, 144.0, 143.9, 138.4, 136.5, 136.2, 133.0, 131.1, 130.8, 130.7, 130.5, 130.1, 129.9, 129.3, 129.0, 128.1, 128.0, 127.9, 127.6, 125.9, 125.7, 125.0, 121.4, 116.6, 112.7, 24.9, 14.0 ppm.

HRMS (ESI) m/z: $[M+H^+]^+$ Calculated for $[C_{28}H_{20}BrN_2O^+]$: 479.0754; found: 479.0759;

HPLC Condition The enantiomeric excess was determined by Daicel Chiralpak IB, Hexanes/IPA = 70/30, 1.0 mL/min, λ = 254 nm, t (minor) = 32.3 min, t (major) = 10.5 min.

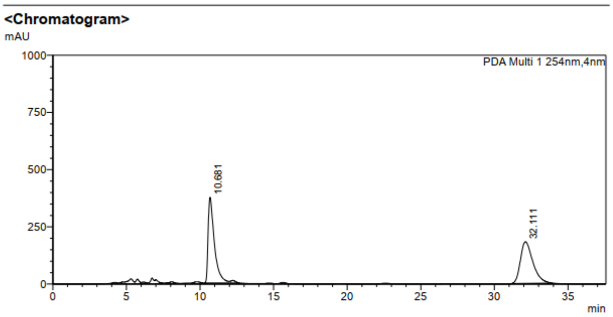

<Peak Table>

PDA Ch1 254nm

Peak#	Ret. Time	Area	Height	Area%
1	10.681	11334323	374049	51.501
2	32.111	10673691	182327	48.499
Total		22008014	556376	100.000

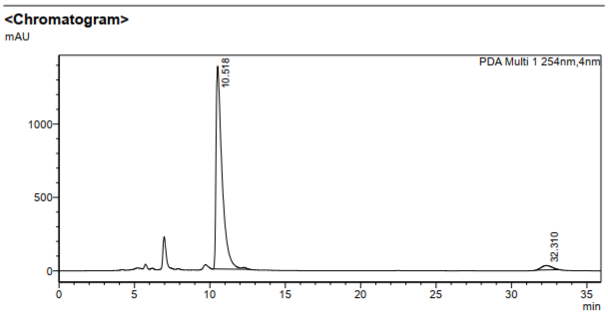

<Peak Table>

PDA Ch1 254nm

Peak#	Ret. Time	Area	Height	Area%
1	10.518	38594867	1378404	96.367
2	32.310	1455205	29740	3.633
Total		40050072	1408144	100.000

(*R*)-6-(7-ethylquinolin-8-yl)-2-iodobenzo[*a*]phenanthridin-5(6*H*)-one (3m):

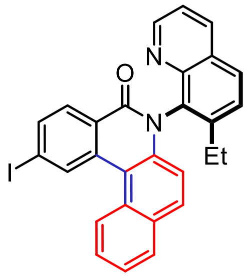

Synthesized according to general procedure GP-A as brownish solid, eluent (PE:EtOAc = 3:1 v/v), Yield: 79% (83.1 mg), 94% ee, m.p. 128˚C-130˚C

$[\alpha]_D^{20}$ = -100.078 (c = 0.1, $CHCl_3$)

^{1}H NMR (400 MHz, $CDCl_3$) δ 9.21 (s, 1H), 8.83 (d, *J* = 8.7 Hz, 1H), 8.69 (dd, *J* = 4.3, 1.7 Hz, 1H), 8.37 (d, *J* = 8.4 Hz, 1H), 8.21 (dd, *J* = 8.3, 1.7 Hz, 1H), 8.00 (d, *J* = 8.5 Hz, 1H), 7.96 (dd, *J* = 8.3, 1.6 Hz, 1H), 7.83 (d, *J* = 7.9 Hz, 1H), 7.73 – 7.69 (m, 2H), 7.60 (d, *J* = 9.1 Hz, 1H), 7.50 (t, *J* = 7.4 Hz, 1H), 7.34 (dd, *J* = 8.3, 4.2 Hz, 1H), 6.61 (d, *J* = 9.1 Hz, 1H), 2.68-2.53 (m, 2H), 1.17 (t, *J* = 7.6 Hz, 3H) ppm.

^{13}C NMR (101 MHz, $CDCl_3$) δ 161.5, 151.4, 144.7, 144.0, 138.3, 136.4, 136.2 (2xC), 136.12, 136.09, 133.0, 130.7 (2xC), 130.0, 129.3, 128.9, 128.1, 128.0, 127.9, 126.4, 125.6, 125.0, 121.4, 116.6, 112.5, 100.1, 24.9, 14.0 ppm.

HRMS (ESI) m/z: $[M+H^+]^+$ Calculated for $[C_{28}H_{20}IN_2O^+]$: 527.0615; found: 527.0620;

HPLC Condition The enantiomeric excess was determined by Daicel Chiralpak IB, Hexanes/IPA = 70/30, 1.0 mL/min, λ = 254 nm, t (minor) = 30.4 min, t (major) = 11.4 min.

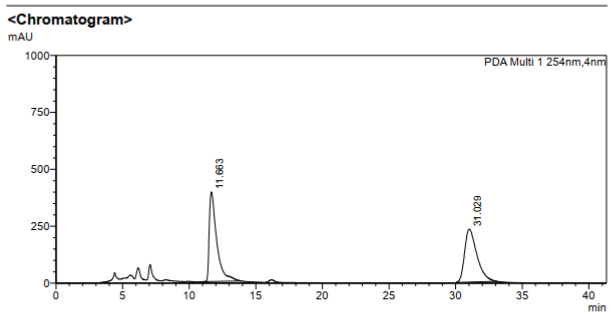

<Peak Table>

PDA Ch1 254nm

Peak#	Ret. Time	Area	Height	Area%
1	11.663	15460194	393118	50.881
2	31.029	14924719	232631	49.119
Total		30384913	625749	100.000

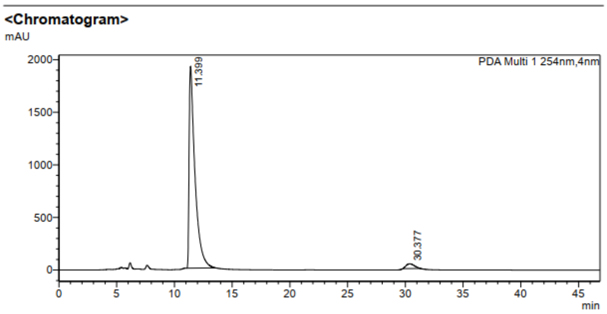

<Peak Table>

PDA Ch1 254nm

Peak#	Ret. Time	Area	Height	Area%
1	11.399	67514576	1917639	96.768
2	30.377	2254902	45169	3.232
Total		69769478	1962807	100.000

(*R*)-6-(7-ethylquinolin-8-yl)-3-methylbenzo[*a*]phenanthridin-5(6*H*)-one (3n):

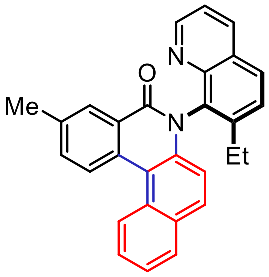

Synthesized according to general procedure GP-A as brownish sticky liquid, eluent (PE:EtOAc = 3:1 v/v), Yield: 77% (63.8 mg), 94% ee.

$[\alpha]_D^{20}$ = -172.116 (c = 0.1, $CHCl_3$)

^{1}H NMR (400 MHz, $CDCl_3$) δ 8.92 (d, *J* = 8.7 Hz, 1H), 8.75 (d, *J* = 8.4 Hz, 1H), 8.69 (dd, *J* = 4.3, 1.6 Hz, 1H), 8.49 (s, 1H), 8.22 (dd, *J* = 8.3, 1.6 Hz, 1H), 8.00 (d, *J* = 8.6 Hz, 1H), 7.82 (d, *J* = 8.0 Hz, 1H), 7.72 – 7.68 (m, 2H), 7.67 – 7.62 (m, 1H), 7.55 (d, *J* = 9.1 Hz, 1H), 7.47 (t, *J* = 7.4 Hz, 1H), 7.34 (dd, *J* = 8.3, 4.2 Hz, 1H), 6.61 (d, *J* = 9.1 Hz, 1H), 2.67 – 2.53 (m, 5H), 1.16 (t, *J* = 7.6 Hz, 3H) ppm.

^{13}C NMR (101 MHz, $CDCl_3$) δ 161.9, 151.4, 144.9, 144.1, 137.5, 137.3, 136.1, 133.5, 133.4, 132.6, 130.8, 130.3, 129.5, 129.1, 129.0, 128.8, 128.1, 128.0, 127.4, 127.1 (2xC), 126.3, 124.6, 121.3, 116.7, 114.1, 24.9, 21.4, 14.0 ppm.

HRMS (ESI) m/z: $[M+H^+]^+$ Calculated for $[C_{29}H_{23}N_2O^+]$: 415.1805; found: 415.1816;

HPLC Condition The enantiomeric excess was determined by Daicel Chiralpak IB, Hexanes/IPA = 70/30, 1.0 mL/min, λ = 254 nm, t (minor) = 22.3 min, t (major) = 9.8 min.

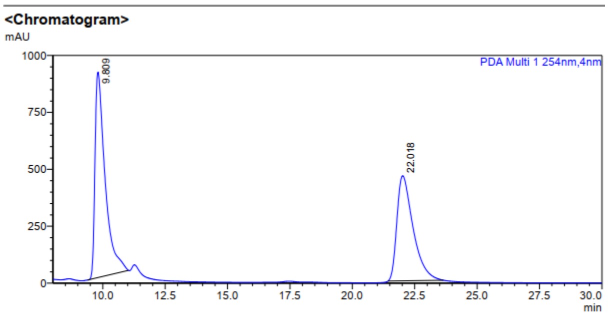

<Peak Table>

PDA Ch1 254nm

Peak#	Ret. Time	Area	Height	Area%
1	9.809	25539715	901892	55.133
2	22.018	20783701	461549	44.867
Total		46323415	1363441	100.000

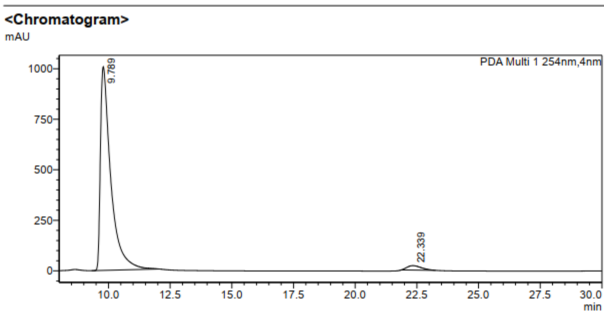

<Peak Table>

PDA Ch1 254nm

Peak#	Ret. Time	Area	Height	Area%
1	9.789	29449332	1007405	97.107
2	22.339	877211	21809	2.893
Total		30326543	1029214	100.000

(*R*)-6-(7-ethylquinolin-8-yl)-3-methoxybenzo[*a*]phenanthridin-5(6*H*)-one (3o):

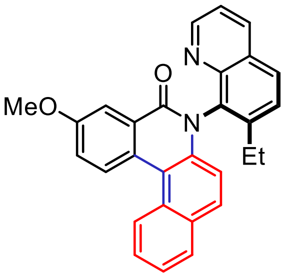

Synthesized according to general procedure GP-A as yellowish solid, eluent (PE:EtOAc = 3:1 v/v), Yield: 74% (63.7 mg), 96% ee, m.p. 133 ℃ - 135 ℃

$[\alpha]_D^{20}$ = -182.090 (c = 0.1, $CHCl_3$)

^{1}H NMR (400 MHz, $CDCl_3$) δ 8.86 (d, *J* = 8.7 Hz, 1H), 8.79 – 8.73 (m, 2H), 8.24 (d, *J* = 8.2 Hz, 1H), 8.13 (d, *J* = 2.8 Hz, 1H), 8.01 (d, *J* = 8.5 Hz, 1H), 7.82 (d, *J* = 8.0 Hz, 1H), 7.72 (d, *J* = 8.6 Hz, 1H), 7.63 (t, *J* = 7.7 Hz, 1H), 7.54 (d, *J* = 9.1 Hz, 1H), 7.49 – 7.45 (m, 2H), 7.37 (dd, *J* = 8.2, 4.2 Hz, 1H), 6.62 (d, *J* = 9.1 Hz, 1H), 3.99 (s, 3H), 2.66 – 2.52 (m, 2H), 1.16 (t, *J* = 7.5 Hz, 3H) ppm.

^{13}C NMR (101 MHz, $CDCl_3$) δ 161.6, 158.9, 151.4, 144.7, 144.1, 136.4, 136.3, 133.4, 130.8, 130.0, 129.13, 129.11, 129.0, 128.8, 128.74, 128.67, 128.1, 128.0, 127.1, 126.3, 124.7, 121.8, 121.3, 116.6, 114.2, 109.6, 55.8, 24.9, 13.9 ppm.

HRMS (ESI) m/z: $[M+H^+]^+$ Calculated for $[C_{29}H_{23}N_2O_2^+]$: 431.1754; found: 431.1760;

HPLC Condition The enantiomeric excess was determined by Daicel Chiralpak IB, Hexanes/IPA = 70/30, 1.0 ml/min, λ = 254 nm, t (minor) = 31.4 min, t (major) = 10.3 min.

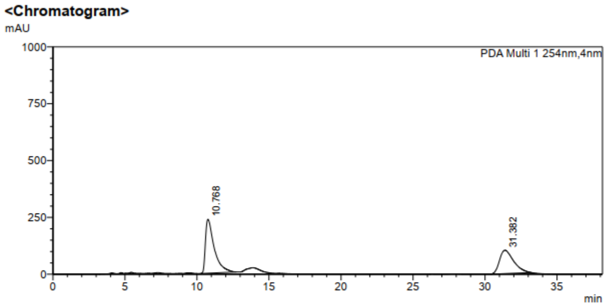

<Peak Table>

PDA Ch1 254nm

Peak#	Ret. Time	Area	Height	Area%
1	10.768	10062975	237334	58.943
2	31.382	7009337	102884	41.057
Total		17072312	340218	100.000

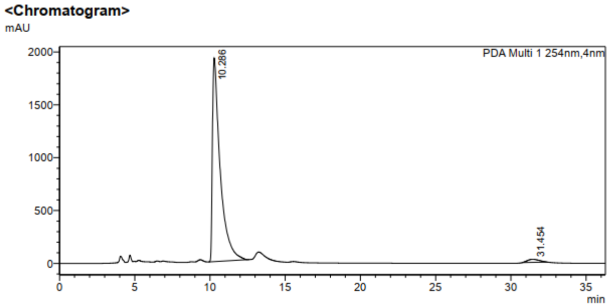

<Peak Table>

PDA Ch1 254nm

Peak#	Ret. Time	Area	Height	Area%
1	10.286	69093798	1926170	97.817
2	31.454	1541926	28459	2.183
Total		70635724	1954629	100.000

(*R*)-3-chloro-6-(7-ethylquinolin-8-yl)benzo[*a*]phenanthridin-5(6*H*)-one (3p):

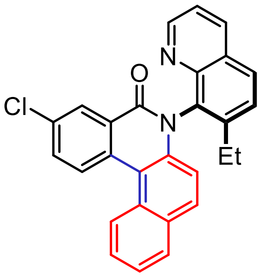

Synthesized according to general procedure GP-A as yellowish solid, eluent (PE:EtOAc = 3:1 v/v), Yield: 70% (60.8 mg), 90% ee, m.p. 148 ℃ - 150 ℃

$[\alpha]_D^{20}$ = -130.480 (c = 0.2, $CHCl_3$)

^{1}H NMR (400 MHz, $CDCl_3$) 8.78 – 8.77 (m, 2H), 8.75 (s, 1H), 8.66 (d, *J* = 2.4 Hz, 1H), 8.26 (d, *J* = 8.1 Hz, 1H), 8.02 (d, *J* = 8.6 Hz, 1H), 7.83 – 7.78 (m, 2H), 7.73 (d, *J* = 8.6 Hz, 1H), 7.62 (dd, *J* = 18.2, 8.4 Hz, 2H), 7.48 (t, *J* = 7.4 Hz, 1H), 7.41 – 7.37 (m, 1H), 6.61 (d, *J* = 9.1 Hz, 1H), 2.66 – 2.52 (m, 2H), 1.17 (t, *J* = 7.6 Hz, 3H) ppm.

^{13}C NMR (101 MHz, $CDCl_3$) δ 160.9, 151.3, 144.4, 144.3, 137.7, 136.6, 133.43, 133.38, 132.8, 132.4, 130.8, 130.4, 130.0, 129.4, 129.0, 128.9, 128.6, 128.5, 128.21, 128.15, 127.6, 125.9, 125.0, 121.5, 116.5, 113.4, 24.9, 13.9 ppm.

HRMS (ESI) m/z: $[M+H^+]^+$ Calculated for $[C_{28}H_{20}ClN_2O^+]$: 435.1259; found: 435.1264;

HPLC Condition The enantiomeric excess was determined by Daicel Chiralpak IB, Hexanes/IPA = 70/30, 1.0 mL/min, λ = 254 nm, t (minor) = 22.9 min, t (major) = 8.1 min.

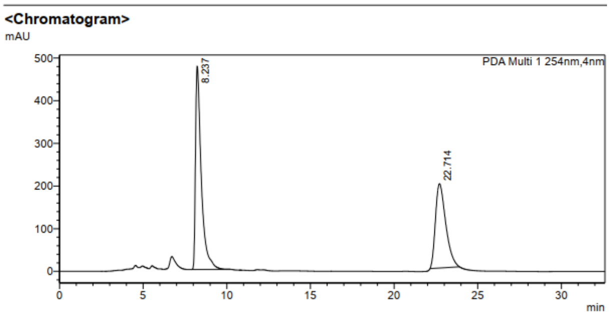

<Peak Table>

PDA Ch1 254nm

Peak#	Ret. Time	Area	Height	Area%
1	8.237	11458990	475913	57.529
2	22.714	8459696	197669	42.471
Total		19918686	673583	100.000

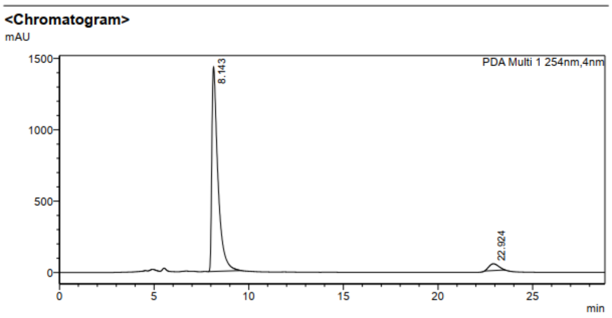

<Peak Table>

PDA Ch1 254nm

Peak#	Ret. Time	Area	Height	Area%
1	8.143	32172410	1432393	95.097
2	22.924	1658607	47021	4.903
Total		33831017	1479414	100.000

(*R*)-methyl 6-(7-ethylquinolin-8-yl)-5-oxo-5,6-dihydrobenzo[*a*]phenanthridine-2-carboxylate (3q):

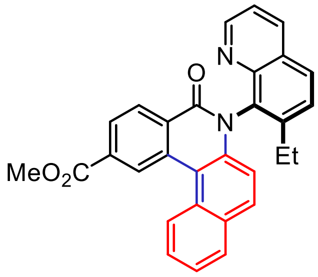

Synthesized according to general procedure GP-A as brownish sticky liquid, eluent (PE:EtOAc = 3:1 v/v), Yield: 68% (62.3 mg), 94% ee.

$[\alpha]_D^{20}$ = -133.665 (c = 0.1, $CHCl_3$)

^{1}H NMR (400 MHz, $CDCl_3$) δ 9.57 (s, 1H), 8.92 (d, *J* = 8.7 Hz, 1H), 8.74 (d, *J* = 8.3 Hz, 1H), 8.69 (d, *J* = 3.1 Hz, 1H), 8.24 (ddd, *J* = 12.0, 8.2, 1.5 Hz, 2H), 8.01 (d, *J* = 8.5 Hz, 1H), 7.85 (d, *J* = 8.0 Hz, 1H), 7.75 – 7.71 (m, 2H), 7.62 (d, *J* = 9.1 Hz, 1H), 7.52 (t, *J* = 7.5 Hz, 1H), 7.35 (dd, *J* = 8.3, 4.2 Hz, 1H), 6.63 (d, *J* = 9.0 Hz, 1H), 4.05 (s, 3H), 2.67 – 2.54 (m, 2H), 1.17 (t, *J* = 7.6 Hz, 3H) ppm.

^{13}C NMR (101 MHz, $CDCl_3$) δ 166.9, 161.2, 151.4, 144.7, 144.0, 138.1, 136.2, 134.9, 133.2, 133.0, 130.8, 130.7, 130.2, 130.0, 129.6, 129.29, 129.27, 128.9, 128.1, 128.0, 127.9, 127.3, 125.9, 125.0, 121.4, 116.6, 113.6, 52.8, 24.9, 13.9 ppm.

HRMS (ESI) m/z: $[M+H^+]^+$ Calculated for $[C_{30}H_{23}N_2O_3^+]$: 459.1703; found: 459.1703;

HPLC Condition The enantiomeric excess was determined by Daicel Chiralpak IB, Hexanes/IPA = 70/30, 1.0 mL/min, λ = 254 nm, t (minor) = 31.7 min, t (major) = 13.9 min.

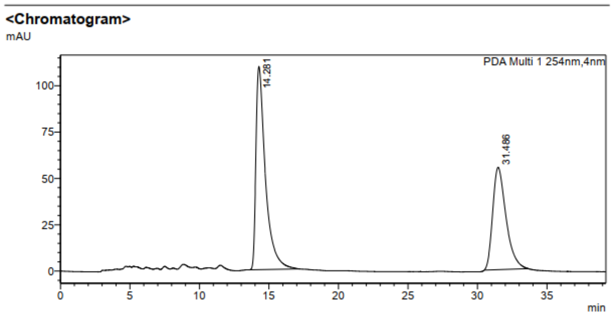

<Peak Table>

PDA Ch1 254nm

Peak#	Ret. Time	Area	Height	Area%
1	14.281	5207967	109561	58.542
2	31.486	3688226	55256	41.458
Total		8896193	164816	100.000

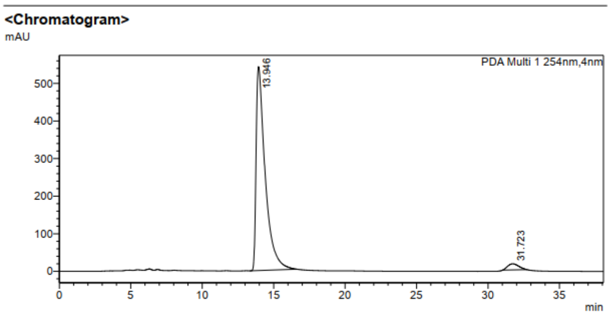

<Peak Table>

PDA Ch1 254nm

Peak#	Ret. Time	Area	Height	Area%
1	13.946	24124771	542224	96.534
2	31.723	866082	16114	3.466
Total		24990853	558338	100.000

(*R*)-2-acetyl-6-(7-ethylquinolin-8-yl)benzo[*a*]phenanthridin-5(6*H*)-one (3r):

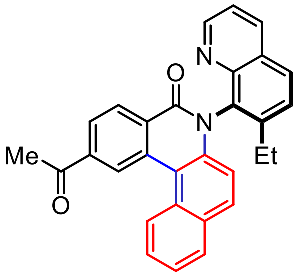

Synthesized according to general procedure GP-A as yellowish solid, eluent (PE:EtOAc = 3:1 v/v), Yield: 70% (61.9 mg), 98% ee, m.p. 114 ℃ - 116 ℃

$[\alpha]_D^{20}$ = -180.632 (c = 0.1, $CHCl_3$)

^{1}H NMR (400 MHz, $CDCl_3$) δ 9.44 (s, 1H), 8.88 (d, *J* = 8.7 Hz, 1H), 8.75 (d, *J* = 8.3 Hz, 1H), 8.71 (s, 1H), 8.25 (d, *J* = 8.0 Hz, 1H), 8.16 (d, *J* = 8.1 Hz, 1H), 8.03 (d, *J* = 8.4 Hz, 1H), 7.86 (d, *J* = 7.8 Hz, 1H), 7.73 (d, *J* = 7.2 Hz, 2H), 7.63 (d, *J* = 9.1 Hz, 1H), 7.52 (t, *J* = 7.2 Hz, 1H), 7.37 (dd, *J* = 8.8, 4.2 Hz, 1H), 6.63 (d, *J* = 9.0 Hz, 1H), 2.80 (s, 3H), 2.66 – 2.52 (m, 2H), 1.16 (t, *J* = 7.6 Hz, 3H) ppm.

^{13}C NMR (101 MHz, $CDCl_3$) δ 198.2, 161.2, 151.5, 144.6, 144.1, 139.5, 138.1, 136.3, 135.0, 132.9, 130.8, 130.7, 130.1, 130.0, 129.8, 129.3, 129.0, 128.1, 128.0, 127.94, 127.91, 126.2, 125.8, 125.1, 121.4, 116.6, 113.7, 27.3, 24.9, 14.0 ppm.

HRMS (ESI) m/z: $[M+H^+]^+$ Calculated for $[C_{30}H_{23}N_2O_2^+]$: 443.1754; found: 443.1758;

HPLC Condition The enantiomeric excess was determined by Daicel Chiralpak IB, Hexanes/IPA = 70/30, 1.0 mL/min, λ = 254 nm, t (minor) = 51.6 min, t (major) = 30.1 min.

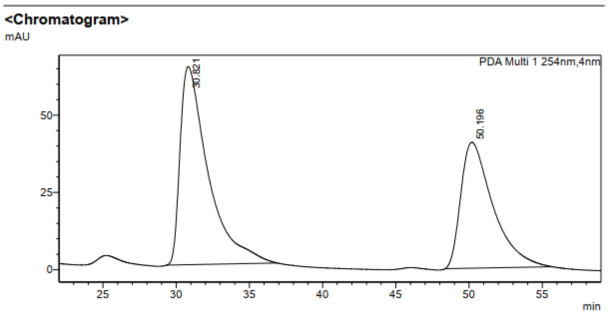

<Peak Table>

PDA Ch1 254nm

Peak#	Ret. Time	Area	Height	Area%
1	30.821	8549237	64058	57.831
2	50.196	6233863	40746	42.169
Total		14783099	104804	100.000

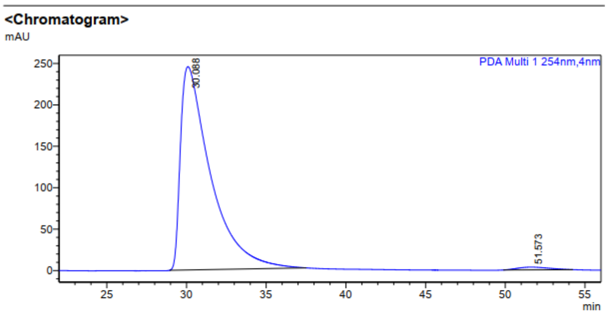

<Peak Table>

PDA Ch1 254nm

Peak#	Ret. Time	Area	Height	Area%
1	30.088	32234035	245419	98.633
2	51.573	446674	3286	1.367
Total		32680709	248704	100.000

(*R*)-6-(7-ethylquinolin-8-yl)-5-oxo-5,6-dihydrobenzo[*a*]phenanthridine-2-carbaldehyde (3s):

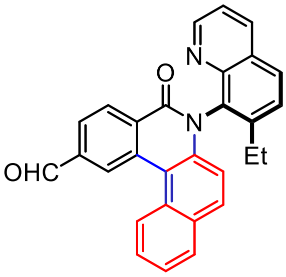

Synthesized according to general procedure GP-A as yellowish solid, eluent (PE:EtOAc = 3:1 v/v), Yield: 68% (58.3 mg), 94% ee, m.p. 131 °C - 133 °C

$[\alpha]_D^{20}$ = -250.934 (c = 0.1, $CHCl_3$)

^{1}H NMR (400 MHz, $CDCl_3$) δ 10.32 (s, 1H), 9.34 (s, 1H), 8.89 (d, *J* = 8.6 Hz, 1H), 8.82 (d, *J* = 8.0 Hz, 1H), 8.70 (s, 1H), 8.25 (d, *J* = 7.9 Hz, 1H), 8.11 (d, *J* = 8.2 Hz, 1H), 8.03 (d, *J* = 8.5 Hz, 1H), 7.86 (d, *J* = 8.1 Hz, 1H), 7.75 – 7.71 (m, 2H), 7.64 (d, *J* = 8.6 Hz, 1H), 7.53 (t, *J* = 7.5 Hz, 1H), 7.39 – 7.36 (m, 1H), 6.64 (d, *J* = 8.9 Hz, 1H), 2.67 – 2.56 (m, 2H), 1.17 (t, *J* = 7.5 Hz, 3H).

^{13}C NMR (101 MHz, $CDCl_3$) δ 192.4, 161.1, 151.4, 144.4, 144.1, 138.5, 138.2, 136.4, 135.5, 132.8, 131.0, 130.9, 130.8, 130.3, 130.2, 130.1, 129.4, 129.0, 128.15, 128.06 (2×C), 126.5, 125.7, 125.2, 121.5, 116.6, 113.4, 24.9, 14.0.

HRMS (ESI) m/z: $[M+H^+]^+$ Calculated for $[C_{29}H_{21}N_2O_2^+]$: 429.1598; found: 429.1603;

HPLC Condition The enantiomeric excess was determined by Daicel Chiralpak IB, Hexanes/IPA = 70/30, 1.0 mL/min, λ = 254 nm, t (minor) = 47.6 min, t (major) = 31.4 min.

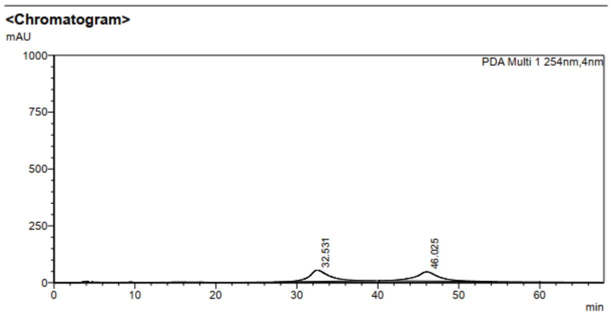

<Peak Table>

PDA Ch1 254nm

Peak#	Ret. Time	Area	Height	Area%
1	32.531	8934547	49487	51.402
2	46.025	8447139	40542	48.598
Total		17381686	90029	100.000

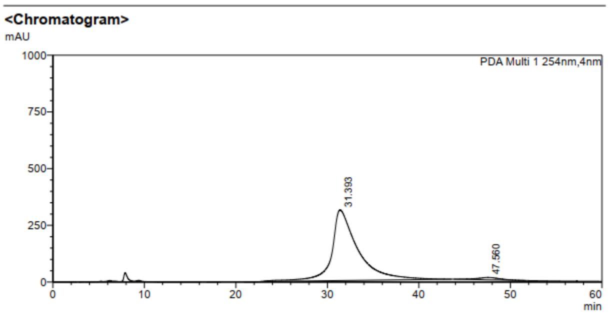

<Peak Table>

PDA Ch1 254nm

Peak#	Ret. Time	Area	Height	Area%
1	31.393	62493261	311316	97.186
2	47.560	1809454	10361	2.814
Total		64302715	321677	100.000

(*R*)-6-(7-ethylquinolin-8-yl)-2-(trifluoromethyl)benzo[*a*]phenanthridin-5(6*H*)-one (3t):

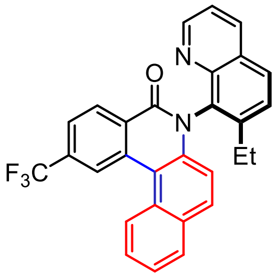

Synthesized according to general procedure GP-A as yellowish sticky liquid, eluent (PE:EtOAc = 3:1 v/v), Yield: 65% (60.8 mg), 88% ee

$[\alpha]_D^{20}$ = -110.461 (c = 0.1, $CHCl_3$)

^{1}H NMR (500 MHz, $CDCl_3$) δ 9.14 (s, 1H), 8.81 (dd, J = 17.1, 8.5 Hz, 2H), 8.68 (dd, J = 4.2, 1.7 Hz, 1H), 8.23 (dd, J = 8.3, 1.7 Hz, 1H), 8.02 (d, J = 8.5 Hz, 1H), 7.88 – 7.85 (m, 2H), 7.75 – 7.72 (m, 2H), 7.63 (d, J = 9.1 Hz, 1H), 7.53 (t, J = 7.3 Hz, 1H), 7.36 (dd, J = 8.3, 4.2 Hz, 1H), 6.64 (d, J = 9.1 Hz, 1H), 2.67 – 2.56 (m, 2H), 1.18 (t, J = 7.6 Hz, 3H) ppm.

^{13}C NMR (126 MHz, $CDCl_3$) δ 161.0, 151.5, 144.8, 144.0, 138.6, 136.1, 135.2,133.7 (q, J = 32.4 Hz), 133.0, 131.0, 130.9, 130.3, 130.1, 129.5, 129.4, 129.1, 128.2, 128.1, 128.0, 125.5, 125.2, 124.6 (q, J = 4.0 Hz), 124.3 (q, J = 272.9 Hz),123.3 (q, J = 3.3 Hz), 121.4, 116.7, 113.1, 25.0, 14.0 ppm.

^{19}F NMR (471 MHz, $CDCl_3$) δ -62.6 ppm.

HRMS (ESI) m/z: $[M+H^+]^+$ Calculated for $[C_{29}H_{20}F_3N_2O^+]$: 469.1522; found: 469.1527;

HPLC Condition The enantiomeric excess was determined by Daicel Chiralpak IB, Hexanes/IPA = 70/30, 1.0 mL/min, λ = 254 nm, t (minor) = 38.1 min, t (major) = 8.3 min.

<Chromatogram>

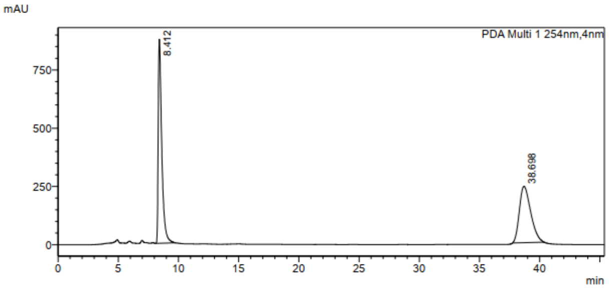

<Peak Table>

PDA Ch1 254nm

Peak#	Ret. Time	Area	Height	Area%
1	8.412	17934148	876487	53.029
2	38.698	15885528	242487	46.971
Total		33819676	1118974	100.000

<Chromatogram>

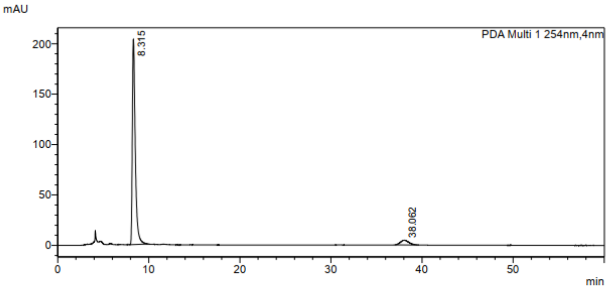

<Peak Table>

PDA Ch1 254nm

Peak#	Ret. Time	Area	Height	Area%
1	8.315	4572833	203661	93.887
2	38.062	297745	4606	6.113
Total		4870578	208267	100.000

(*R*)- 6-(7-ethylquinolin-8-yl)-4-methylbenzo[*a*]phenanthridin-5(6*H*)-one (3u):

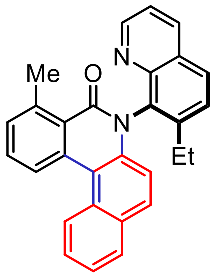

Synthesized according to general procedure GP-A as yellowish sticky liquid, eluent (PE:EtOAc = 3:1 v/v), Yield: 50% (41.5 mg), 90% ee

$[\alpha]_D^{20}$ = -190.324 (c = 0.1, $CHCl_3$)

^{1}H NMR (400 MHz, $CDCl_3$) δ 8.87 – 8.84 (m, 1H), 8.70 (dd, *J* = 4.2, 1.7 Hz, 1H), 8.64 (d, *J* = 8.3 Hz, 1H), 8.21 (dd, *J* = 8.3, 1.8 Hz, 1H), 7.98 (d, *J* = 8.5 Hz, 1H), 7.80 (dd, *J* = 8.2, 1.4 Hz, 1H), 7.70 (d, *J* = 8.3 Hz, 2H), 7.63 – 7.59 (m, 1H), 7.53 (d, *J* = 9.0 Hz, 1H), 7.47 – 7.41 (m, 2H), 7.34 (dd, *J* = 8.3, 4.2 Hz, 1H), 6.52 (d, *J* = 9.0 Hz, 1H), 2.96 (s, 3H), 2.68 – 2.54 (m, 2H), 1.16 (t, *J* = 7.6 Hz, 3H) ppm.

^{13}C NMR (101 MHz, $CDCl_3$) δ 162.4, 151.3, 145.1, 144.0, 142.6, 137.7, 136.5, 136.1, 133.8, 131.0, 130.8, 130.7, 130.4, 129.8, 128.9, 128.7, 128.2, 128.1, 127.0, 126.4, 126.0, 125.8, 124.5, 121.2, 116.4, 114.1, 25.0, 24.5, 14.0 ppm.

HRMS (ESI) m/z: $[M+H^+]^+$ Calculated for $[C_{29}H_{23}N_2O^+]$: 415.1805; found: 415.1786;

HPLC Condition The enantiomeric excess was determined by Daicel Chiralpak IB, Hexanes/IPA = 70/30, 1.0 mL/min, λ = 254 nm, t (minor) = 7.1 min, t (major) = 5.9 min.

<Chromatogram>

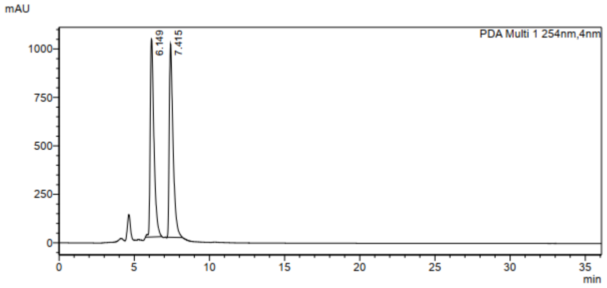

<Peak Table>

PDA Ch1 254nm

Peak#	Ret. Time	Area	Height	Area%
1	6.149	17738581	1022823	50.301
2	7.415	17526291	996335	49.699
Total		35264872	2019158	100.000

<Chromatogram>

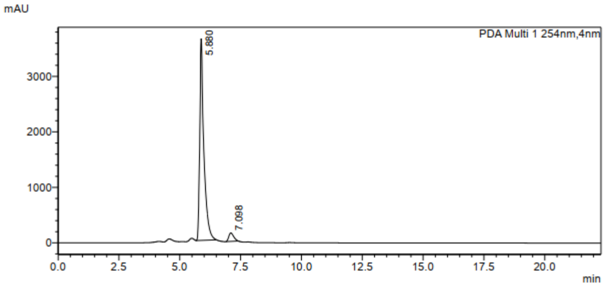

<Peak Table>

PDA Ch1 254nm

Peak#	Ret. Time	Area	Height	Area%
1	5.880	42677834	3636967	95.268
2	7.098	2119732	151093	4.732
Total		44797567	3788059	100.000

(*R*)-5-(7-ethylquinolin-8-yl)benzo[*f*]thieno[2,3-c]quinolin-4(5*H*)-one (3v):

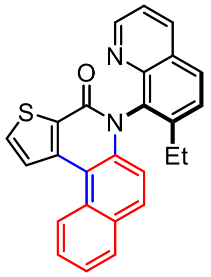

Synthesized according to general procedure GP-A as yellowish solid, eluent (PE:EtOAc = 3:1 v/v), Yield: 65% (52.8 mg), 90% ee, m.p. 195 ℃ - 198 ℃

$[\alpha]_D^{20}$ = -126.048 (c = 0.1, $CHCl_3$)

^{1}H NMR (400 MHz, $CDCl_3$) δ 8.96 (d, *J* = 8.7 Hz, 1H), 8.71 (dd, *J* = 4.2, 1.7 Hz, 1H), 8.52 (d, *J* = 5.5 Hz, 1H), 8.23 (dd, *J* = 8.3, 1.7 Hz, 1H), 8.03 – 7.98 (m, 2H), 7.84 (d, *J* = 8.0 Hz, 1H), 7.73 – 7.66 (m, 2H), 7.61 (d, *J* = 9.3 Hz, 1H), 7.52 (t, *J* = 7.5 Hz, 1H), 7.35 (dd, *J* = 8.4, 4.3 Hz, 1H), 6.72 (d, *J* = 9.2 Hz, 1H), 2.65 – 2.55 (m, 2H), 1.17 (t, *J* = 7.6 Hz, 3H) ppm.

^{13}C NMR (101 MHz, $CDCl_3$) δ 158.1, 151.5, 144.9, 144.2, 142.1, 138.9, 136.1, 133.5, 133.0, 132.9, 130.3, 130.2, 129.8, 129.3, 129.0, 128.1, 128.0, 127.7, 126.7, 125.0, 124.9, 121.4, 117.3, 113.4, 24.9, 13.9 ppm.

HRMS (ESI) m/z: $[M+H^+]^+$ Calculated for $[C_{26}H_{19}SN_2O^+]$: 407.1213; found: 407.1218;

HPLC Condition The enantiomeric excess was determined by Daicel Chiralpak IH, Hexanes/IPA = 95/5, 1.0 mL/min, λ = 254 nm, t (minor) = 36.3 min, t (major) = 40.2 min.

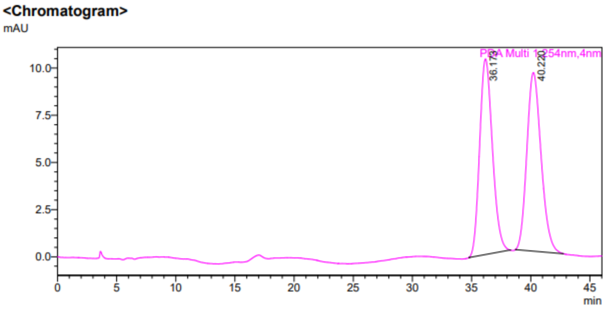

<Peak Table>

PDA Ch1 254nm

Peak#	Ret. Time	Area	Height	Height%	Area%
1	36.173	779024	10366	52.284	49.551
2	40.220	793131	9460	47.716	50.449
Total		1572155	19826	100.000	100.000

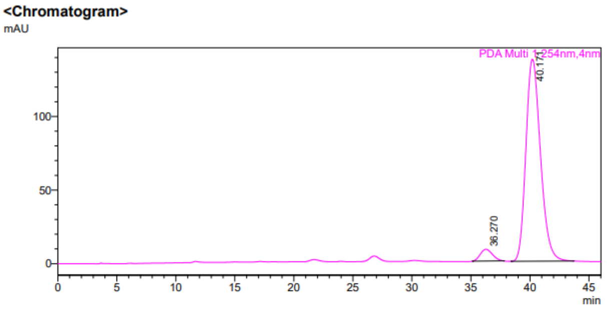

<Peak Table>

PDA Ch1 254nm

Peak#	Ret. Time	Area	Height	Height%	Area%
1	36.270	575790	7996	5.507	4.659
2	40.171	11782000	137207	94.493	95.341
Total		12357790	145202	100.000	100.000

(*R*)-4-(7-ethylquinolin-8-yl)-1,2-dimethylbenzo[*f*]quinolin-3(4*H*)-one (3w):

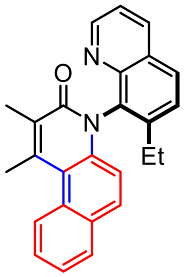

Synthesized according to general procedure GP-A as yellowish liquid, eluent (PE:EtOAc = 3:1 v/v), Yield: 82% (62.0 mg), 94% ee

$[\alpha]_D^{20}$ = -283.773 (c = 0.1, $CHCl_3$)

^{1}H NMR (400 MHz, $CDCl_3$) δ 8.74 (s, 1H), 8.58 (d, *J* = 8.7 Hz, 1H), 8.21 (d, *J* = 8.1 Hz, 1H), 7.98 (d, *J* = 8.5 Hz, 1H), 7.77 (d, *J* = 7.9 Hz, 1H), 7.69 (d, *J* = 8.5 Hz, 1H), 7.59 (t, *J* = 8.1 Hz, 1H), 7.53 (d, *J* = 9.1 Hz, 1H), 7.45 (t, *J* = 7.6 Hz, 1H), 7.37 – 7.33 (m, 1H), 6.57 (d, *J* = 9.1 Hz, 1H), 2.96 (s, 3H), 2.64 – 2.44 (m, 5H), 1.15 (t, *J* = 7.3 Hz, 3H) ppm.

^{13}C NMR (101 MHz, $CDCl_3$) δ 161.7, 151.5, 144.6, 144.4, 143.6, 138.6, 136.1, 133.7, 130.6, 130.5, 130.3, 129.1, 128.94, 128.86, 128.0, 127.9, 126.6, 126.3, 124.5, 121.2, 116.9, 116.5, 24.8, 22.9, 14.4, 13.8 ppm.

HRMS (ESI) m/z: $[M+H^+]^+$ Calculated for $[C_{26}H_{23}N_2O^+]$: 379.1805; found: 379.1816;

HPLC Condition The enantiomeric excess was determined by Daicel Chiralpak IB, Hexanes/IPA = 70/30, 1.0 mL/min, λ = 254 nm, t (minor) = 21.7 min, t (major) = 13.9 min.

<Chromatogram>

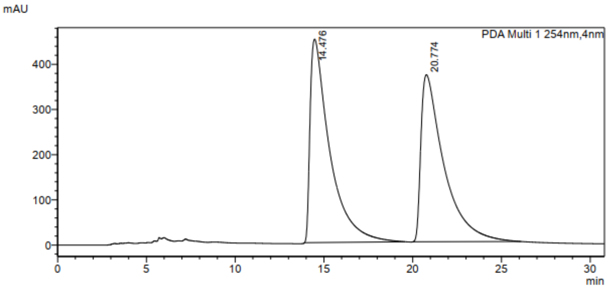

<Peak Table>

PDA Ch1 254nm

Peak#	Ret. Time	Area	Height	Area%
1	14.476	34652689	450254	51.015
2	20.774	33273682	369803	48.985
Total		67926371	820058	100.000

<Chromatogram>

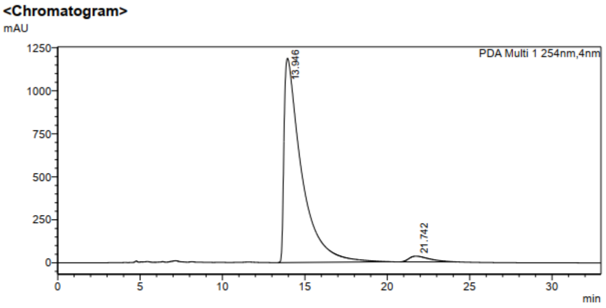

<Peak Table>

PDA Ch1 254nm

Peak#	Ret. Time	Area	Height	Area%
1	13.946	87612105	1187256	96.851
2	21.742	2848353	33156	3.149
Total		90460459	1220412	100.000

(*R*)-6-(7-ethylquinolin-8-yl)-2,3,4,6-tetrahydrobenzo[*a*]phenanthridin-5(1*H*)-one (3x):

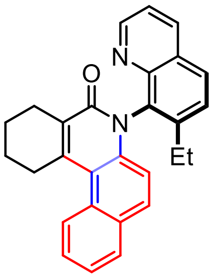

Synthesized according to general procedure GP-A as brownish solid, eluent (PE:EtOAc = 3:1 v/v), Yield: 76% (61.4 mg), 93% ee, m.p. 119 ℃ - 121 ℃

$[\alpha]_D^{20}$ = -162.888 (c = 0.1, $CHCl_3$)

^{1}H NMR (400 MHz, $CDCl_3$) δ 8.74 – 8.69 (m, 1H), 8.21 (d, *J* = 7.8 Hz, 1H), 7.98 (d, *J* = 8.5 Hz, 1H), 7.77 (d, *J* = 7.4 Hz, 1H), 7.69 (d, *J* = 8.3 Hz, 1H), 7.59 – 7.52 (m, 2H), 7.46 – 7.42 (m, 1H), 7.397 – 7.33 (m, 1H), 6.61 (d, *J* = 9.0 Hz, 1H), 3.49 (s, 2H), 2.86 (s, 2H), 2.62 – 2.47 (m, 2H), 1.99 – 1.89 (m, 4H), 1.14 (t, *J* = 7.2 Hz, 3H) ppm.

^{13}C NMR (101 MHz, $CDCl_3$) δ 161.5, 151.5, 146.3, 144.7, 143.6, 138.6, 136.1, 133.7, 130.8, 130.6, 130.3, 129.3, 129.1, 128.9, 128.0, 127.9, 126.8, 126.3, 124.4, 121.2, 116.7, 116.6, 34.1, 25.4, 24.8, 23.5, 22.0, 13.8 ppm.

HRMS (ESI) m/z: $[M+H^+]^+$ Calculated for $[C_{28}H_{25}N_2O^+]$: 405.1961; found: 405.1971;

HPLC Condition The enantiomeric excess was determined by Daicel Chiralpak IB, Hexanes/IPA = 70/30, 1.0 mL/min, λ = 254 nm, t (minor) = 27.9 min, t (major) = 15.2 min.

<Chromatogram>

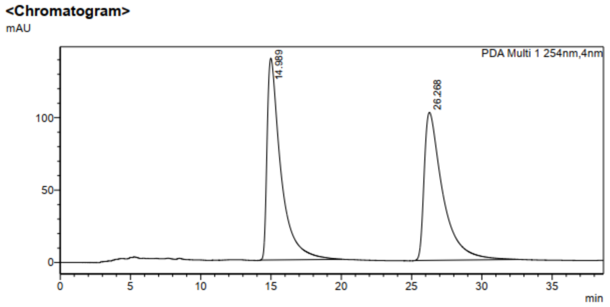

<Peak Table>

PDA Ch1 254nm

Peak#	Ret. Time	Area	Height	Area%
1	14.989	9507710	139349	50.340
2	26.268	9379125	102140	49.660
Total		18886835	241490	100.000

<Chromatogram>

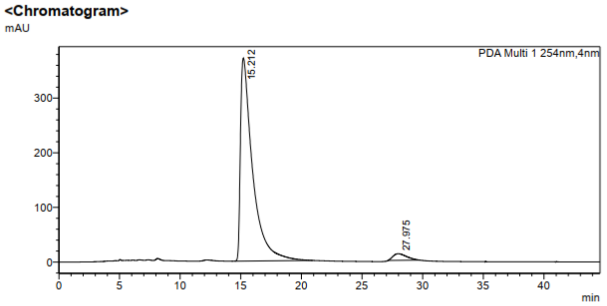

<Peak Table>

PDA Ch1 254nm

Peak#	Ret. Time	Area	Height	Area%
1	15.212	25883330	371479	96.458
2	27.975	950436	12144	3.542
Total		26833766	383623	100.000

(*R*) - 6-(7-methylquinolin-8-yl)benzo[*a*]phenanthridin-5(6*H*)-one (3y):

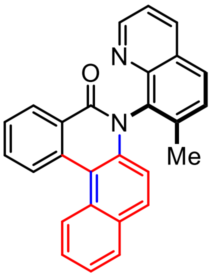

Synthesized according to general procedure GP-A as brownish sticky liquid, eluent (PE:EtOAc = 3:1 v/v), Yield: 80% (61.8 mg), 90% ee.

$[\alpha]_D^{20}$ = -97.466 (c = 0.2, $CHCl_3$)

^{1}H NMR (400 MHz, $CDCl_3$) δ 8.94 (d, *J* = 8.7 Hz, 1H), 8.85 (d, *J* = 8.3 Hz, 1H), 8.71 – 8.68 (m, 2H), 8.23 (d, *J* = 8.2 Hz, 1H), 7.96 (d, *J* = 8.4 Hz, 1H), 7.90 – 7.83 (m, 2H), 7.67 – 7.64 (m, 3H), 7.59 (d, *J* = 9.3 Hz, 1H), 7.51 – 7.47 (m, 1H), 7.37 – 7.34 (m, 1H), 6.62 (d, *J* = 9.0 Hz, 1H), 2.28 (s, 3H) ppm.

^{13}C NMR (101 MHz, $CDCl_3$) δ 161.5, 151.5, 144.9, 138.8, 137.3, 136.2, 135.0, 134.0, 132.1, 130.8, 130.4, 130.2, 129.8, 129.2, 128.81, 128.82, 128.1, 127.4, 127.34, 127.30, 127.2, 126.3, 124.7, 121.3, 116.3, 114.0, 18.5 ppm.

HRMS (ESI) m/z: $[M+H^+]^+$ Calculated for $[C_{27}H_{19}N_2O^+]$: 387.1492; found: 387.1494;

HPLC Condition The enantiomeric excess was determined by Daicel Chiralpak IB, Hexanes/IPA = 70/30, 1.0 mL/min, λ = 254 nm, t (minor) = 23.6 min, t (major) = 14.5 min.

<Chromatogram>

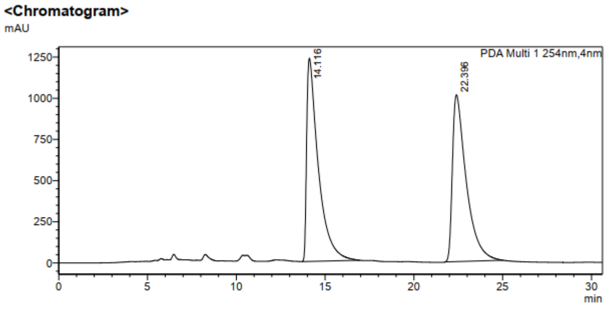

<Peak Table>

PDA Ch1 254nm

Peak#	Ret. Time	Area	Height	Area%
1	14.116	56230058	1233510	50.639
2	22.396	54810373	1011991	49.361
Total		111040430	2245501	100.000

<Chromatogram>

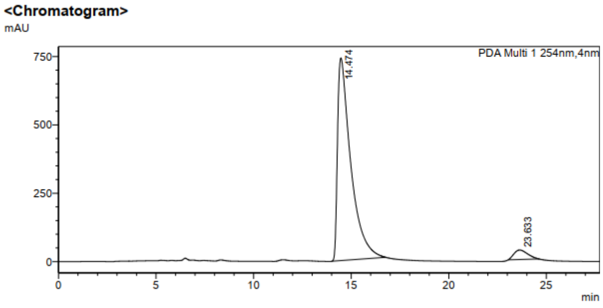

<Peak Table>

PDA Ch1 254nm

Peak#	Ret. Time	Area	Height	Area%
1	14.474	34970714	740979	95.328
2	23.633	1714006	34882	4.672
Total		36684720	775861	100.000

(*R*)-6-(7-methoxyquinolin-8-yl)benzo[*a*]phenanthridin-5(6*H*)-one (3z):

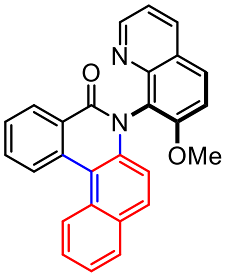

Synthesized according to general procedure GP-A as yellowish sticky liquid, eluent (PE:EtOAc = 2:1 v/v), Yield: 62% (49.9 mg), 82% ee.

$[\alpha]_D^{20}$ = -60.848 (c = 0.1, $CHCl_3$)

^{1}H NMR (400 MHz, $CDCl_3$) δ 8.88 (d, *J* = 8.7 Hz, 1H), 8.82 – 8.78 (m, 2H), 8.68 (d, *J* = 7.9 Hz, 1H), 8.23 (d, *J* = 7.9 Hz, 1H), 8.06 (d, *J* = 9.0 Hz, 1H), 7.84 (dd, *J* = 13.5, 7.5 Hz, 2H), 7.65 – 7.56 (m, 4H), 7.46 (t, *J* = 7.5 Hz, 1H), 7.32 – 7.30 (m, 1H), 6.72 (d, *J* = 8.9 Hz, 1H), 3.86 (s, 3H) ppm .

^{13}C NMR (101 MHz, $CDCl_3$) δ 161.8, 156.7, 152.1, 145.4, 137.7, 136.8, 135.1, 132.0, 130.8, 130.5, 130.4, 130.0, 129.2, 128.7, 127.4, 127.3, 127.2, 127.1, 126.2, 124.6, 124.4, 120.9, 119.9, 116.6, 114.6, 114.2, 56.8 ppm.

HRMS (ESI) m/z: $[M+H^+]^+$ Calculated for $[C_{27}H_{19}N_2O_2^+]$: 403.1441; found: 403.1446;

HPLC Condition The enantiomeric excess was determined by Daicel Chiralpak IB, Hexanes/IPA = 70/30, 1.0 mL/min, λ = 254 nm, t (minor) = 36.6 min, t (major) = 25.7 min.

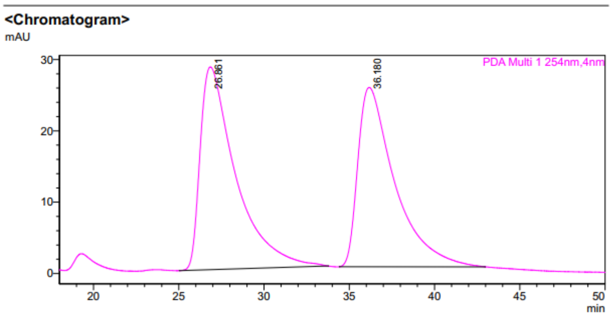

<Peak Table>
PDA Ch1 254nm

Peak#	Ret. Time	Area	Height	Area%
1	26.861	4120207	28425	52.103
2	36.180	3787595	25140	47.897
Total		7907802	53565	100.000

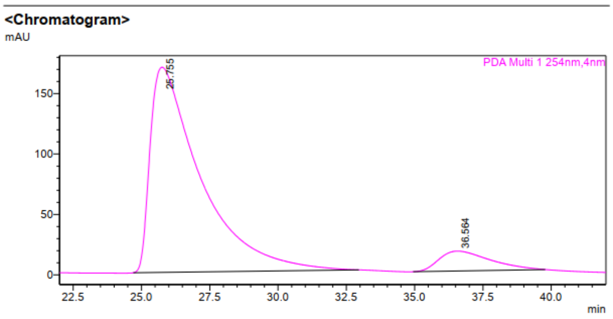

<Peak Table>
PDA Ch1 254nm

Peak#	Ret. Time	Area	Height	Area%
1	25.755	21853554	169755	91.086
2	36.564	2138700	16469	8.914
Total		23992254	186224	100.000

(*R*)-6-(7-ethylquinolin-8-yl)-11-methylbenzo[*a*]phenanthridin-5(6*H*)-one (4a):

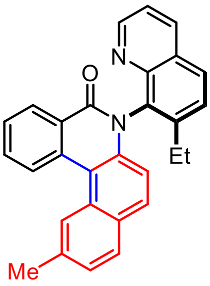

Synthesized according to general procedure GP-A as yellowish sticky liquid, eluent (PE:EtOAc = 3:1 v/v), Yield: 75% (62.1 mg), 88% ee.

$[\alpha]_D^{20}$ = -179.723 (c = 0.1, $CHCl_3$)

^{1}H NMR (400 MHz, $CDCl_3$) δ 8.86 (d, *J* = 8.4 Hz, 1H), 8.72 – 8.66 (m, 3H), 8.23 (d, *J* = 8.1 Hz, 1H), 8.00 (d, *J* = 8.4 Hz, 1H), 7.88 (t, *J* = 7.7 Hz, 1H), 7.72 (t, *J* = 7.6 Hz, 2H), 7.65 (t, *J* = 7.6 Hz, 1H), 7.53 (d, *J* = 8.9 Hz, 1H), 7.37 – 7.21 (m, 2H), 6.54 (d, *J* = 8.9 Hz, 1H), 2.63 – 2.54 (m, 5H), 1.15 (t, *J* = 7.6 Hz, 3H) ppm.

^{13}C NMR (101 MHz, $CDCl_3$) δ 161.9, 151.4, 144.1, 138.0, 137.2 (2×C), 136.2, 135.2, 133.4, 132.0, 130.6, 129.8, 129.3, 129.1, 129.0, 128.7, 128.1, 128.0, 127.3, 127.2, 127.1, 126.8, 125.6, 121.3, 115.8, 113.5, 24.9, 22.5, 13.9 ppm.

HRMS (ESI) m/z: $[M+H^+]^+$ Calculated for $[C_{29}H_{23}N_2O^+]$: 415.1805; found: 415.1810;

HPLC Condition The enantiomeric excess was determined by Daicel Chiralpak IB, Hexanes/IPA = 70/30, 1.0 mL/min, λ = 254 nm, t (minor) = 14.1 min, t (major) = 10.5 min.

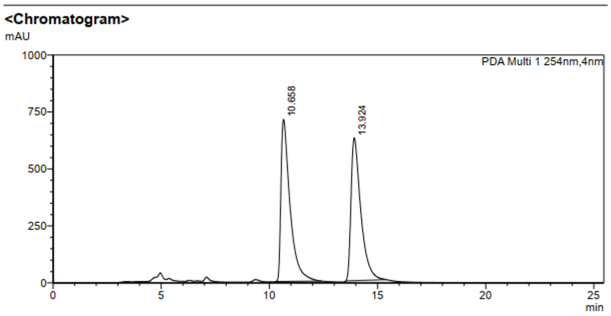

<Peak Table>

PDA Ch1 254nm

Peak#	Ret. Time	Area	Height	Area%
1	10.658	20759024	710815	51.327
2	13.924	19685633	625968	48.673
Total		40444657	1336783	100.000

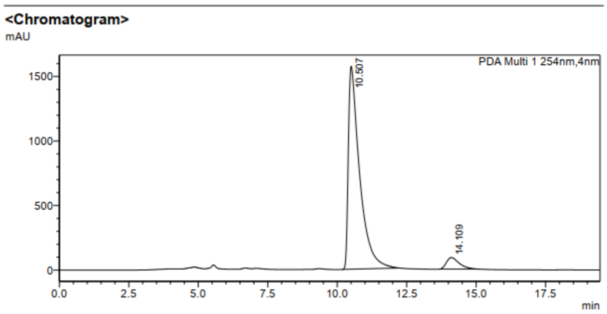

<Peak Table>

PDA Ch1 254nm

Peak#	Ret. Time	Area	Height	Area%
1	10.507	44630396	1570558	93.906
2	14.109	2896485	89944	6.094
Total		47526880	1660503	100.000

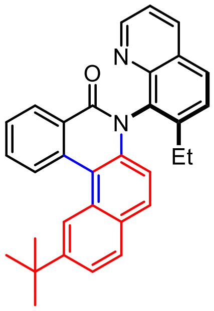

(*R*)-11-(tert-butyl)-6-(7-ethylquinolin-8-yl)benzo[*a*]phenanthridin-5(6*H*)-one (4b):

Synthesized according to general procedure GP-A as yellowish sticky liquid, eluent (PE:EtOAc = 4:1 v/v), Yield: 72% (65.7 mg), 96% ee.

$[\alpha]_D^{20}$ = -227.349 (c = 0.1, $CHCl_3$)

^{1}H NMR (400 MHz, $CDCl_3$) δ 8.91 (s, 1H), 8.84 (d, *J* = 8.3 Hz, 1H), 8.70 – 8.67 (m, 2H), 8.22 (dd, *J* = 8.3, 1.7 Hz, 1H), 8.00 (d, *J* = 8.6 Hz, 1H), 7.90 – 7.87 (m, 1H), 7.77 (d, *J* = 8.5 Hz, 1H), 7.71 (d, *J* = 8.6 Hz, 1H), 7.67 – 7.64 (m, 1H), 7.58 (dd, *J* = 8.5, 1.8 Hz, 1H), 7.53 (d, *J* = 9.0 Hz, 1H), 7.35 (dd, *J* = 8.3, 4.2 Hz, 1H), 6.55 (d, *J* = 9.0 Hz, 1H), 2.66 – 2.54 (m, 2H), 1.50 (s, 9H), 1.16 (t, *J* = 7.6 Hz, 3H) ppm.

^{13}C NMR (101 MHz, $CDCl_3$) 161.9, 151.4, 150.1, 144.9, 144.1, 137.9, 136.1, 135.3, 133.5, 131.9, 130.2, 129.5, 129.3, 129.1, 128.9, 128.4, 128.1, 128.0, 127.3, 127.23, 127.17, 123.4, 122.0, 121.3, 116.0, 114.1, 35.5, 31.6, 24.9, 13.9 ppm.

HRMS (ESI) m/z: $[M+H^+]^+$ Calculated for $[C_{32}H_{29}N_2O^+]$: 457.2274; found: 457.2273;

HPLC Condition The enantiomeric excess was determined by Daicel Chiralpak IB, Hexanes/IPA = 70/30, 1.0 mL/min, λ = 254 nm, t (minor) = 11.6 min, t (major) = 8.5 min.

<Chromatogram>

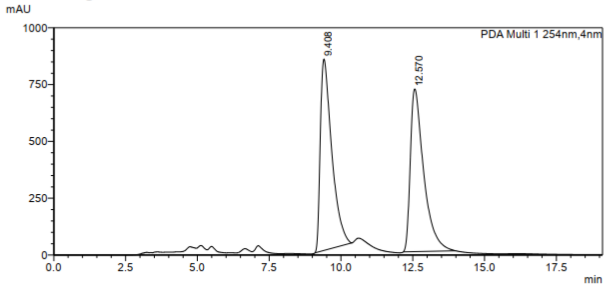

<Peak Table>

PDA Ch1 254nm

Peak#	Ret. Time	Area	Height	Area%
1	9.408	23466647	842341	50.277
2	12.570	23207936	715078	49.723
Total		46674584	1557419	100.000

<Chromatogram>

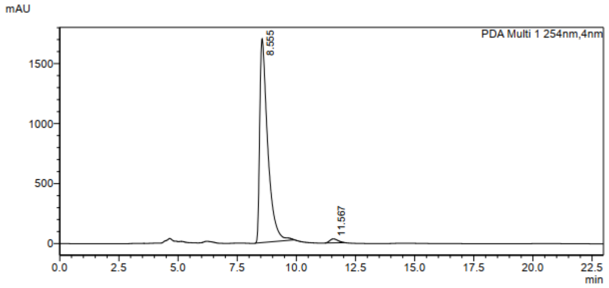

<Peak Table>

PDA Ch1 254nm

Peak#	Ret. Time	Area	Height	Area%
1	8.555	39978139	1697722	98.040
2	11.567	799077	33181	1.960
Total		40777216	1730903	100.000

(*R*)-11-fluoro-6-(quinolin-8-yl)benzo[*a*]phenanthridin-5(6*H*)-one (4c):

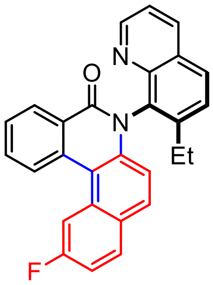

Synthesized according to general procedure GP-A as brownish sticky liquid, eluent (PE:EtOAc = 5:1 v/v), Yield: 71% (59.4 mg), 94% ee.

$[\alpha]_D^{20}$ = -136.458 (c = 0.1, $CHCl_3$)

1H NMR (400 MHz, $CDCl_3$) δ 8.80 (d, *J* = 8.4 Hz, 1H), 8.74 (s, 1H), 8.70 (d, *J* = 7.9 Hz, 1H), 8.60 - 8.57 (d, *J* = 12.5 Hz, 1H), 8.26 (d, *J* = 8.4 Hz, 1H), 8.04 (d, *J* = 8.6 Hz, 1H), 7.92 (t, *J* = 8.0 Hz, 1H), 7.85 – 7.81 (m, 1H), 7.74 (d, *J* = 8.8 Hz, 1H), 7.71 – 7.67 (m, 1H), 7.58 (d, *J* = 9.1 Hz, 1H), 7.41 – 7.38 (m, 1H), 7.30 – 7.26 (m, 1H), 6.60 (d, *J* = 9.1 Hz, 1H), 2.69 – 2.57 (m, 2H), 1.18 (t, *J* = 7.2 Hz, 3H) ppm.

^{13}C NMR (126 MHz, $CDCl_3$) δ 162.3 (d, *J* = 245.7 Hz), 161.8, 151.4, 144.7, 144.2, 138.7, 136.3, 134.8, 133.2, 132.3, 131.4 (d, *J* = 9.4 Hz), 131.1 (d, *J* = 9.5 Hz), 129.8, 129.4, 129.2, 128.2, 128.1, 127.7, 127.5, 127.2, 126.7, 121.4, 116.1, 114.5 (d, *J* = 24.8 Hz), 113.5 (d, *J* = 4.7 Hz), 110.7 (d, *J* = 24.0 Hz), 25.0, 13.9 ppm.

^{19}F NMR (471 MHz, $CDCl_3$) δ -112.4.

HRMS (ESI) m/z: $[M+H^+]^+$ Calculated for $[C_{28}H_{20}FN_2O^+]$: 419.1554; found: 419.1559;

HPLC Condition The enantiomeric excess was determined by Daicel Chiralpak IB, Hexanes/IPA = 70/30, 1.0 mL/min, λ = 254 nm, t (minor) = 16.1 min, t (major) = 12.8 min.

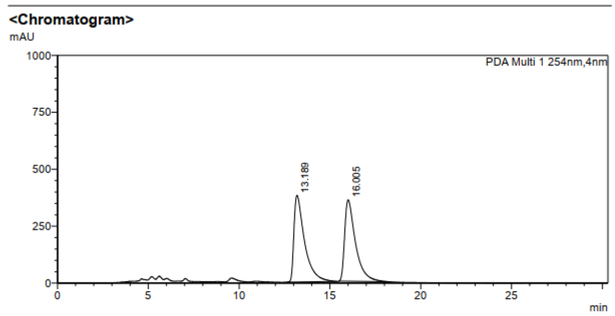

<Peak Table>

PDA Ch1 254nm

Peak#	Ret. Time	Area	Height	Area%
1	13.189	15532305	380809	51.075
2	16.005	14878593	357563	48.925
Total		30410898	738371	100.000

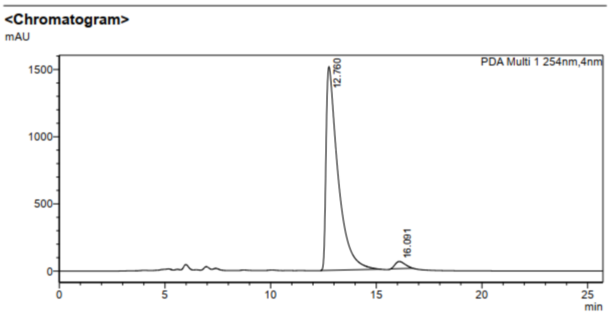

<Peak Table>

PDA Ch1 254nm

Peak#	Ret. Time	Area	Height	Area%
1	12.760	59713752	1515618	97.173
2	16.091	1737015	53067	2.827
Total		61450767	1568686	100.000

(*R*)-11-chloro-6-(quinolin-8-yl)benzo[*a*]phenanthridin-5(6*H*)-one (4d):

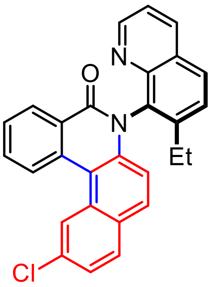

Synthesized according to general procedure GP-A as yellowish sticky liquid, eluent (PE:EtOAc = 4:1 v/v), Yield: 74% (64.2 mg), 90% ee

$[\alpha]_D^{20}$ = -148.873 (c = 0.1, $CHCl_3$)

^{1}H NMR (400 MHz, $CDCl_3$)δ 8.90 (s, 1H), 8.76 (d, *J* = 8.3 Hz, 1H), 8.71 – 8.61 (m, 2H), 8.23 (dd, *J* = 8.4, 1.7 Hz, 1H), 8.01 (d, *J* = 8.5 Hz, 1H), 7.94 – 7.90 (m, 1H), 7.77 – 7.66 (m, 3H), 7.54 (d, *J* = 9.1 Hz, 1H), 7.43 (dd, *J* = 8.5, 1.9 Hz, 1H), 7.36 (dd, *J* = 8.3, 4.2 Hz, 1H), 6.61 (d, *J* = 9.1 Hz, 1H), 2.64 – 2.53 (m, 2H), 1.16 (t, *J* = 7.6 Hz, 3H) ppm.

^{13}C NMR (101 MHz, $CDCl_3$) δ 161.8, 151.4, 144.8, 144.1, 138.5, 136.2, 134.6, 133.7, 133.1, 132.5, 131.1, 130.3, 129.6, 129.4, 129.2, 129.0, 128.2, 128.0, 127.6, 127.3, 126.9, 125.5, 125.4, 121.4, 117.0, 113.2, 24.9, 14.0 ppm.

HRMS (ESI) m/z: $[M+H^+]^+$ Calculated for $[C_{28}H_{20}ClN_2O^+]$: 435.1259; found: 435.1268;

HPLC Condition The enantiomeric excess was determined by Daicel Chiralpak IB, Hexanes/IPA = 70/30, 1.0 mL/min, λ = 254 nm, t (minor) = 18.6 min, t (major) = 12.8 min.

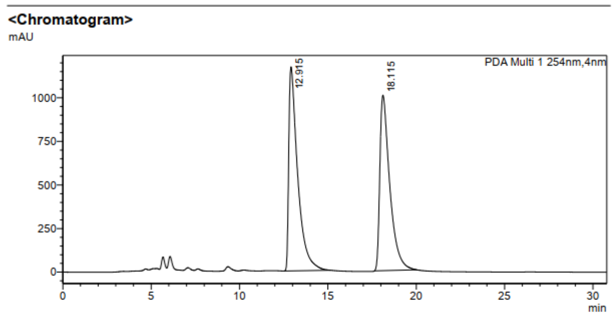

<Peak Table>

PDA Ch1 254nm

Peak#	Ret. Time	Area	Height	Area%
1	12.915	41190139	1169338	51.171
2	18.115	39304822	1005568	48.829
Total		80494961	2174905	100.000

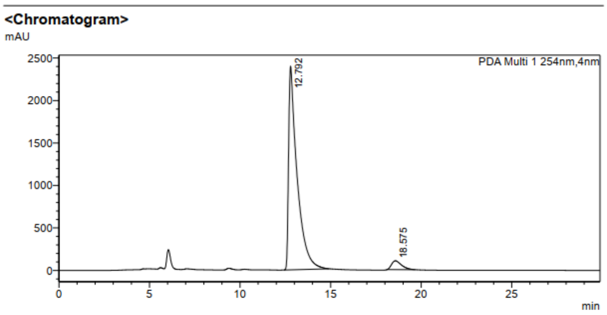

<Peak Table>

PDA Ch1 254nm

Peak#	Ret. Time	Area	Height	Area%
1	12.792	78088257	2392524	95.204
2	18.575	3933394	103343	4.796
Total		82021651	2495866	100.000

(*R*)-11-bromo-6-(quinolin-8-yl)benzo[*a*]phenanthridin-5(6*H*)-one (4e):

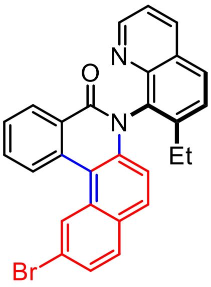

Synthesized according to general procedure GP-A as yellowish solid, eluent (PE:EtOAc = 3:1 v/v), Yield: 78% (74.6 mg), 94% ee, m.p. 128 ℃ - 130 ℃.

$[\alpha]_D^{20}$ = -178.111 (c = 0.1, $CHCl_3$)

^{1}H NMR (400 MHz, $CDCl_3$) δ 9.07 (s, 1H), 8.75 (d, *J* = 8.3 Hz, 1H), 8.69 – 8.67 (m, 2H), 8.22 (dd, *J* = 8.2, 1.7 Hz, 1H), 8.01 (d, *J* = 8.5 Hz, 1H), 7.92 (t, *J* = 7.5 Hz, 1H), 7.72 – 7.66 (m, 3H), 7.56 - 7.52 (m, 2H), 7.35 (dd, *J* = 8.3, 4.2 Hz, 1H), 6.63 (d, *J* = 9.0 Hz, 1H), 2.66 – 2.53 (m, 2H), 1.16 (t, *J* = 7.6 Hz, 3H).

^{13}C NMR (101 MHz, $CDCl_3$) δ 161.7, 151.5, 144.8, 144.1, 138.4, 136.2, 134.5, 133.1, 132.50, 131.5, 130.3, 129.7, 129.4, 129.24, 129.16, 128.5, 128.1, 128.02, 127.99, 127.6, 127.2, 126.9, 122.0, 121.4, 117.2, 113.1, 24.9, 14.0.

HRMS (ESI) m/z: $[M+H^+]^+$ Calculated for $[C_{28}H_{20}BrN_2O^+]$: 479.0759; found: 481.0745;

HPLC Condition The enantiomeric excess was determined by Daicel Chiralpak IB, Hexanes/IPA = 70/30, 1.0 mL/min, λ = 254 nm, t (minor) = 19.1 min, t (major) = 14.0 min.

<Chromatogram>

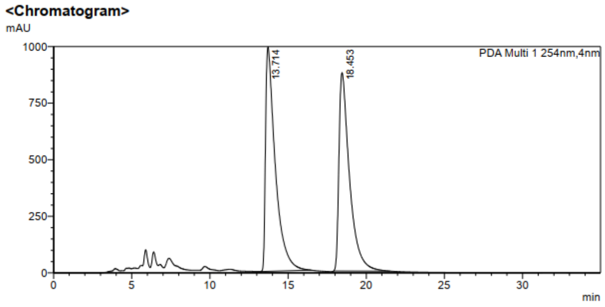

<Peak Table>

PDA Ch1 254nm

Peak#	Ret. Time	Area	Height	Area%
1	13.714	43082317	990201	51.134
2	18.453	41170932	876697	48.866
Total		84253250	1866899	100.000

<Chromatogram>

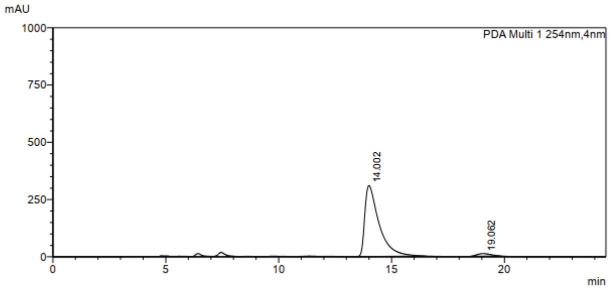

<Peak Table>

PDA Ch1 254nm

Peak#	Ret. Time	Area	Height	Area%
1	14.002	13613045	309620	96.689
2	19.062	466138	11226	3.311
Total		14079182	320847	100.000

(*R*)-1-(7-ethylquinolin-8-yl)naphtho[2,1-*a*]phenanthridin-2(1*H*)-one (4f):

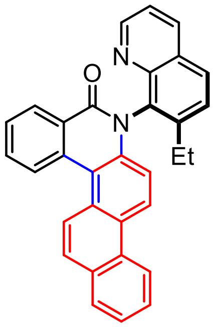

Synthesized according to general procedure GP-A as brownish liquid, eluent (PE:EtOAc = 4:1 v/v), Yield: 80% (72.0 mg), 96% ee.

$[\alpha]_D^{20}$ = -312.972 (c = 0.1, $CHCl_3$)

^{1}H NMR (400 MHz, $CDCl_3$) δ 8.84 – 8.69 (m, 4H), 8.53 (d, *J* = 7.9 Hz, 1H), 8.46 (d, *J* = 8.9 Hz, 1H), 8.25 (d, *J* = 8.1 Hz, 1H), 8.03 (d, *J* = 8.5 Hz, 1H), 7.95 – 7.87 (m, 3H), 7.74 (d, *J* = 8.5 Hz, 1H), 7.70 – 7.66 (m, 1H), 7.64 – 7.57 (m, 2H), 7.37 – 7.35 (m, 1H), 6.76 (d, *J* = 9.0 Hz, 1H), 2.70 – 2.56 (m, 2H), 1.17 (t, *J* = 7.4 Hz, 3H).

^{13}C NMR (101 MHz, $CDCl_3$) δ 161.8, 151.4, 144.7, 144.3, 138.1, 136.4, 134.8, 133.1, 131.9, 131.0, 130.5, 129.3, 129.2, 129.0, 128.4, 128.2, 128.1, 128.0, 127.7, 127.6, 127.5, 127.1, 127.0, 126.4, 125.1, 124.3, 122.6, 121.3, 116.1, 115.6, 24.9, 14.0.

HRMS (ESI) m/z: $[M+H^+]^+$ Calculated for $[C_{32}H_{23}N_2O^+]$: 451.1805; found: 451.1808;

HPLC Condition The enantiomeric excess was determined by Daicel Chiralpak IB, Hexanes/IPA = 70/30, 1.0 mL/min, λ = 254 nm, t (minor) = 20.7 min, t (major) = 17.0 min.

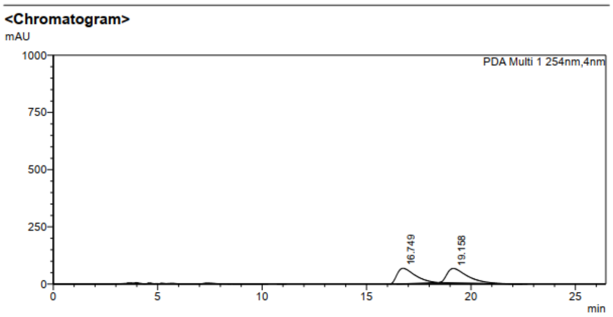

<Peak Table>

PDA Ch1 254nm

Peak#	Ret. Time	Area	Height	Area%
1	16.749	4095318	67967	49.112
2	19.158	4243410	62490	50.888
Total		8338728	130457	100.000

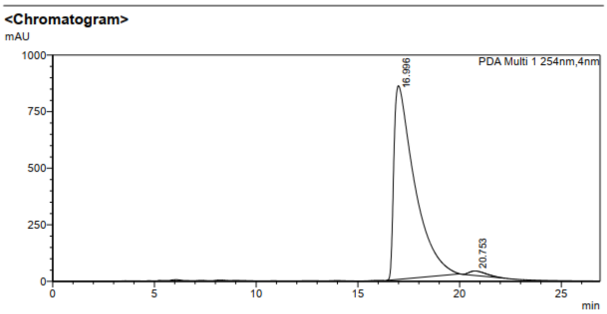

<Peak Table>

PDA Ch1 254nm

Peak#	Ret. Time	Area	Height	Area%
1	16.996	60770132	855438	98.130
2	20.753	1158184	19672	1.870
Total		61928316	875110	100.000

(*R*)-6-(7-ethylquinolin-8-yl)-5-oxo-*N*,*N*-dipropyl-5,6-dihydrobenzo[*a*]phenanthridine-2-sulfonamide (4g):

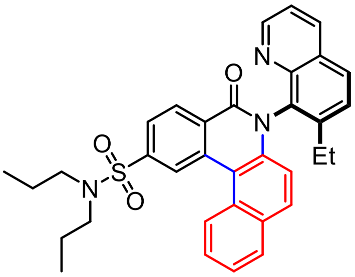

Synthesized according to general procedure GP-A as light-yellow sticky liquid, eluent (PE:EtOAc = 4:1 v/v), Yield: 74% (83.4 mg), 90% ee.

$[\alpha]_D^{20}$ = -150.093 (c = 0.1, $CHCl_3$)

^{1}H NMR (400 MHz, $CDCl_3$) δ 9.31 (s, 1H), 8.85 (d, *J* = 8.7 Hz, 1H), 8.78 (d, *J* = 8.2 Hz, 1H), 8.70 (s, 1H), 8.25 (d, *J* = 8.2 Hz, 1H), 8.02 (dd, *J* = 13.2, 8.4 Hz, 2H), 7.86 (d, *J* = 8.0 Hz, 1H), 7.72 (t, *J* = 8.9 Hz, 2H), 7.65 (d, *J* = 8.9 Hz, 1H), 7.53 (t, *J* = 7.4 Hz, 1H), 7.38 (dd, *J* = 8.5, 4.0 Hz, 1H), 6.64 (d, *J* = 8.9 Hz, 1H), 3.24 (t, *J* = 7.7 Hz, 4H), 2.64 – 2.55 (m, 2H), 1.71 – 1.61 (m, 4H), 1.17 (t, *J* = 7.6 Hz, 3H), 0.94 (t, *J* = 7.3 Hz, 6H).

^{13}C NMR (101 MHz, $CDCl_3$) δ 160.9, 151.4, 144.5, 144.1, 143.5, 138.5, 136.4, 135.3, 132.7, 131.2, 130.8, 130.5, 130.0, 129.4, 129.3, 129.1, 128.18, 128.16, 128.1, 126.4, 125.4, 125.2, 124.5, 121.5, 116.5, 113.1, 50.5, 24.9, 22.4, 14.0, 11.4.

HRMS (ESI) m/z: $[M+H^+]^+$ Calculated for $[C_{34}H_{34}SN_3O_3^+]$: 564.2315; found: 564.2321;

HPLC Condition The enantiomeric excess was determined by Daicel Chiralpak IB, Hexanes/IPA = 70/30, 1.0 mL/min, λ = 254 nm, t (minor) = 15.0 min, t (major) = 35.8 min.

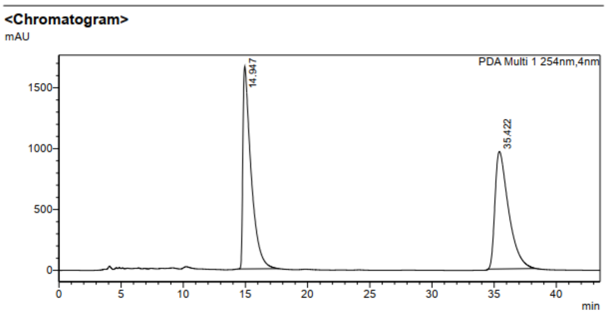

<Peak Table>
PDA Ch1 254nm

Peak#	Ret. Time	Area	Height	Area%
1	14.947	76271967	1661425	50.894
2	35.422	73591317	965122	49.106
Total		149863284	2626547	100.000

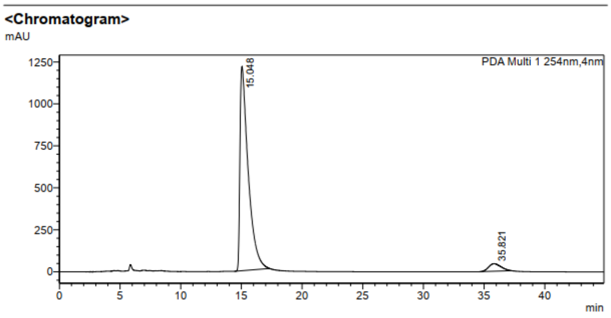

<Peak Table>
PDA Ch1 254nm

Peak#	Ret. Time	Area	Height	Area%
1	15.048	57273680	1215602	94.755
2	35.821	3170073	44868	5.245
Total		60443753	1260470	100.000

(*R*)-(1*R*,2*S*,5*R*)-2-isopropyl-5-methylcyclohexyl 6-(7-ethylquinolin-8-yl)-5-oxo-5,6-dihydrobenzo[*a*]phenanthridine-11-carboxylate (4h):

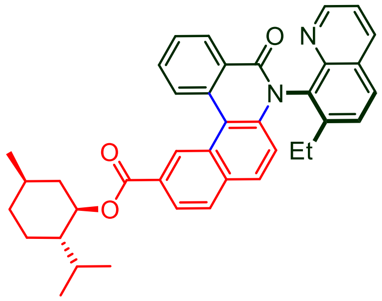

Synthesized according to general procedure GP-A as brownish sticky liquid, eluent (PE:EtOAc = 4:1 v/v), Yield: 70% (81.6 mg), 94% de.

$[\alpha]_D^{20}$ = 437.313 (c = 0.5, $CHCl_3$)

^{1}H NMR (400 MHz, $CDCl_3$) δ 9.71 (s, 1H), 8.82 (d, *J* = 8.2 Hz, 1H), 8.70 (d, *J* = 9.0 Hz, 2H), 8.25 – 8.13 (m, 1H), 8.09 (d, *J* = 8.0 Hz, 1H), 8.02 (d, *J* = 8.0 Hz, 1H), 7.94 – 7.86 (m, 2H), 7.73 – 7.69 (m, 2H), 7.62 (d, *J* = 8.1 Hz, 1H), 7.36 (s, 1H), 6.73 (d, *J* = 8.4 Hz, 1H), 5.06 – 5.01 (m, 1H), 2.66 – 2.57 (m, 2H), 2.25 (d, *J* = 11.8 Hz, 1H), 2.16 – 2.12 (m, 1H), 1.81 – 1.76 (m, 2H), 1.69 – 1.63 (m, 2H), 1.19 – 1.15 (m, 4H), 1.02 – 0.96 (m, 8H), 0.90 – 0.89 (m, 3H).

^{13}C NMR (101 MHz, $CDCl_3$) δ 166.5, 161.8, 151.4, 144.7, 144.1, 138.1, 136.2, 134.5, 133.0, 132.9, 132.4, 129.5 (2xC), 129.31, 129.27, 129.2, 129.1, 128.8, 128.1, 128.0, 127.8, 127.5, 127.4, 124.4, 121.4, 118.9, 114.9, 75.4, 47.5, 41.1, 34.4, 31.6, 26.9, 24.9, 23.9, 22.2, 21.0, 16.8, 14.0.

HRMS (ESI) m/z: $[M+H^+]^+$ Calculated for $[C_{39}H_{39}N_2O_3^+]$: 583.2955; found: 583.2954;

HPLC Condition The enantiomeric excess was determined by Daicel Chiralpak IB, Hexanes/IPA = 70/30, 1.0 mL/min, λ = 254 nm, t (minor) = 16.7 min, t (major) = 10.8 min.

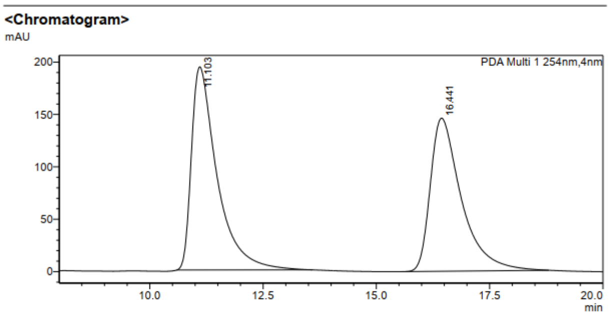

<Peak Table>
PDA Ch1 254nm

Peak#	Ret. Time	Area	Height	Area%
1	11.103	7651218	193922	52.299
2	16.441	6978602	146132	47.701
Total		14629820	340054	100.000

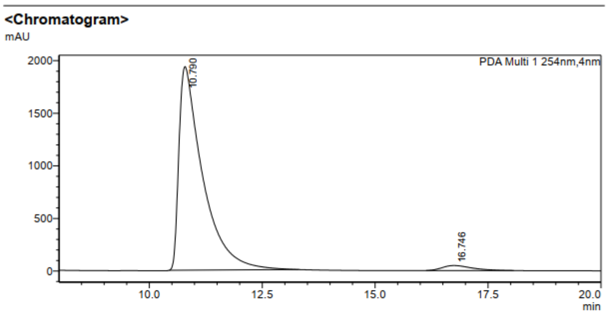

<Peak Table>
PDA Ch1 254nm

Peak#	Ret. Time	Area	Height	Area%
1	10.790	70816020	1934859	96.983
2	16.746	2202682	47779	3.017
Total		73018702	1982638	100.000

(*R*)-(1*R*,2*S*,5*R*)-2-isopropyl-5-methylcyclohexyl 2-(*N*,*N*-dipropylsulfamoyl)-6-(7-ethylquinolin-8-yl)-5-oxo-5,6-dihydrobenzo[*a*]phenanthridine-11-carboxylate (4i):

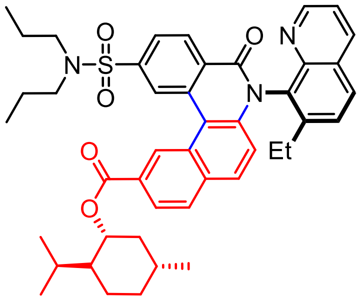

Synthesized according to general procedure GP-A as colourless oil, eluent (PE:EtOAc = 4:1 v/v), Yield: 65% (96.9 mg), 85% de.

$[\alpha]_D^{20}$ = -589.440 (c = 0.1, $CHCl_3$)

^{1}H NMR (400 MHz, $CDCl_3$) δ 9.61 (s, 1H), 9.26 (s, 1H), 8.79 (d, *J* = 8.2 Hz, 1H), 8.68 (s, 1H), 8.25 (d, *J* = 7.8 Hz, 1H), 8.12 – 8.03 (m, 3H), 7.90 (d, *J* = 8.3 Hz, 1H), 7.70 (dd, *J* = 23.8, 8.6 Hz, 2H), 7.40 – 7.36 (m, 1H), 6.73 (d, *J* = 9.1 Hz, 1H), 5.03 (t, *J* = 11.0 Hz, 1H), 3.40 – 3.32 (m, 4H), 2.64 – 2.57 (m, 2H), 2.19 (d, *J* = 12.2 Hz, 1H), 2.04 – 1.98 (m, 1H), 1.77 – 1.65 (m, 9H), 1.35 – 1.26 (m, 2H), 1.17 (t, *J* = 7.9 Hz, 3H), 1.01 – 0.92 (m, 12H), 0.81 (d, *J* = 6.8 Hz, 3H).

^{13}C NMR (101 MHz, $CDCl_3$) δ 166.0, 160.9, 151.5, 144.5, 144.4, 144.1, 138.8, 136.3, 135.0, 132.8, 132.6, 130.6, 130.5, 130.2, 129.5, 129.4, 129.3, 129.1, 128.3, 128.2, 128.1, 126.3, 125.3, 124.8, 121.5, 118.7, 114.1, 75.6, 50.4, 47.0, 41.0, 34.4, 31.7, 26.6, 25.0, 23.8, 22.3, 22.2, 20.9, 16.6, 14.0, 11.3.

HRMS (ESI) m/z: $[M+H^+]^+$ Calculated for $[C_{45}H_{52}SN_3O_5^+]$: 746.3622; found: 746.3624;

HPLC Condition The enantiomeric excess was determined by Daicel Chiralpak IB, Hexanes/IPA = 70/30, 1.0 mL/min, λ = 254 nm, t (minor) = 33.1 min, t (major) = 12.1 min.

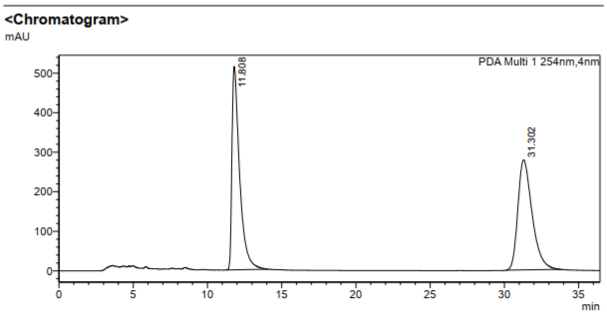

<Peak Table>
PDA Ch1 254nm

Peak#	Ret. Time	Area	Height	Area%
1	11.808	18416178	513943	49.795
2	31.302	18568048	278251	50.205
Total		36984226	792193	100.000

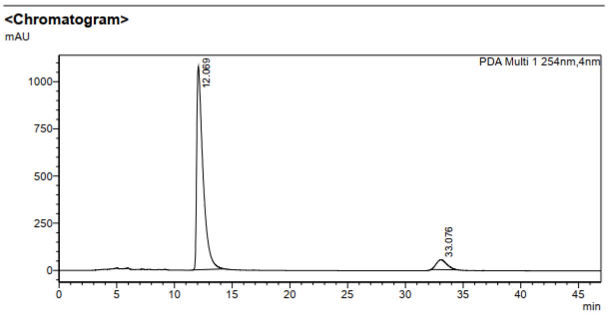

<Peak Table>
PDA Ch1 254nm

Peak#	Ret. Time	Area	Height	Area%
1	12.069	41013677	1077036	92.487
2	33.076	3331558	52059	7.513
Total		44345235	1129095	100.000

(*R*)-(*E*)-2-(7-ethylquinolin-8-yl)-3-styrylisoquinolin-1(2*H*)-one (5a):

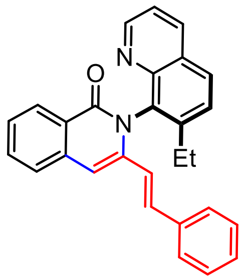

Synthesized according to general procedure GP-B as yellowish solid, eluent (PE:EtOAc = 2:1 v/v), Yield: 70% (56.3 mg), 94% ee, m.p. 100 ℃ - 103 ℃.

$[\alpha]_D^{20}$ = 10.0 (c = 0.1, $CHCl_3$)

^{1}H NMR (400 MHz, $CDCl_3$) δ 8.84 (d, *J* = 3.2 Hz, 1H), 8.43 (d, *J* = 8.0 Hz, 1H), 8.20 (d, *J* = 8.1 Hz, 1H), 7.93 (d, *J* = 8.5 Hz, 1H), 7.71 – 7.60 (m, 4H), 7.48 (t, *J* = 7.2 Hz, 1H), 7.38 – 7.35 (m, 1H), 7.19 – 7.16 (m, 3H), 7.06 – 7.01 (m, 3H), 6.12 (d, *J* = 15.9 Hz, 1H), 2.69 – 2.61 (m, 2H), 1.23 (t, *J* = 7.6 Hz, 3H).

^{13}C NMR (101 MHz, $CDCl_3$) δ 162.9, 151.5, 144.7, 143.6, 141.8, 137.6, 136.3, 136.1, 133.8, 132.8, 132.7, 129.1, 128.7, 128.6, 128.4, 127.69, 127.67, 126.8, 126.6, 126.4, 125.7, 122.2, 121.3, 104.1, 24.9, 13.8

HRMS (ESI) m/z: $[M+H^+]^+$ Calculated for $[C_{28}H_{23}N_2O^+]$: 403.1805; found: 403.1807;

HPLC Condition The enantiomeric excess was determined by Daicel Chiralpak IB, Hexanes/IPA = 70/30, 1.0 mL/min, λ = 254 nm, t (minor) = 14.0 min, t (major) = 11.1 min.

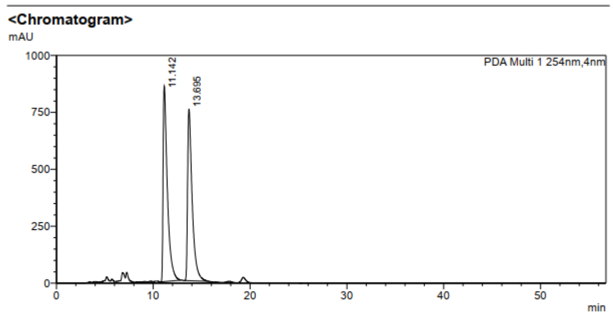

<Peak Table>

PDA Ch1 254nm

Peak#	Ret. Time	Area	Height	Area%
1	11.142	26375628	858307	51.562
2	13.695	24777253	753413	48.438
Total		51152882	1611720	100.000

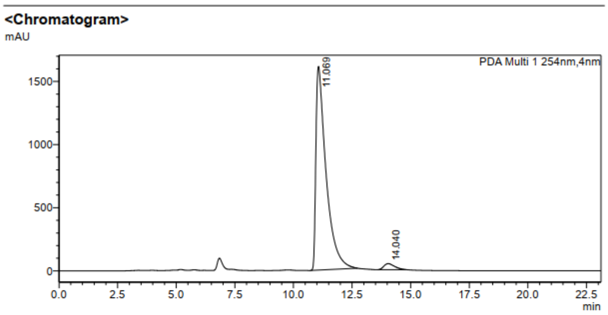

<Peak Table>

PDA Ch1 254nm

Peak#	Ret. Time	Area	Height	Area%
1	11.069	48745972	1611168	96.993
2	14.040	1511399	47721	3.007
Total		50257371	1658889	100.000

(*R*)-(*E*)-2-(7-ethylquinolin-8-yl)-6-methyl-3-styrylisoquinolin-1(2*H*)-one (5b):

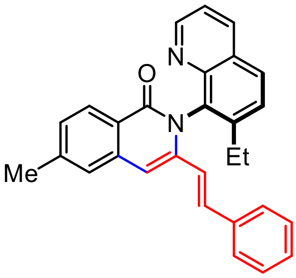

Synthesized according to general procedure GP-B as yellowish sticky liquid, eluent (PE:EtOAc = 2:1 v/v), Yield: 67% (55.8 mg), 90% ee.

$[\alpha]_D^{20}$ = 50.021 (c = 0.1, $CHCl_3$)

^{1}H NMR (400 MHz, $CDCl_3$) δ 8.86 (s, 1H), 8.33 (d, *J* = 8.1 Hz, 1H), 8.20 (d, *J* = 8.2 Hz, 1H), 7.93 (d, *J* = 8.4 Hz, 1H), 7.62 (d, *J* = 8.4 Hz, 1H), 7.44 (s, 1H), 7.38 – 7.36 (m, 1H), 7.31 (d, *J* = 8.3 Hz, 1H), 7.18 – 7.17 (m, 3H), 7.09 – 6.99 (m, 4H), 6.12 (d, *J* = 15.8 Hz, 1H), 2.69 – 2.52 (m, 2H), 2.52 (s, 3H), 1.23 (t, *J* = 7.4 Hz, 3H).

^{13}C NMR (101 MHz, $CDCl_3$) δ 162.9, 151.4, 144.7, 143.6, 143.2, 141.7, 137.7, 136.3, 136.2, 133.8, 132.6, 129.0, 128.8, 128.7, 128.4, 128.3, 127.7, 127.6, 126.8, 126.1, 123.5, 122.3, 121.2, 104.0, 24.8, 22.0, 13.8

HRMS (ESI) m/z: $[M+H^+]^+$ Calculated for $[C_{29}H_{25}N_2O^+]$: 417.1961; found: 417.1967;

HPLC Condition The enantiomeric excess was determined by Daicel Chiralpak ID, Hexanes/IPA = 60/40, 1.0 mL/min, λ = 254 nm, t (minor) = 12.7 min, t (major) = 20.7 min

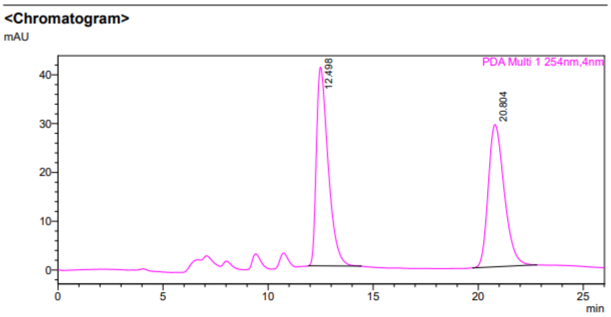

<Peak Table>

PDA Ch1 254nm

Peak#	Ret. Time	Area	Height	Height%	Area%
1	12.498	1585057	40711	58.274	50.540
2	20.804	1551167	29150	41.726	49.460
Total		3136223	69861	100.000	100.000

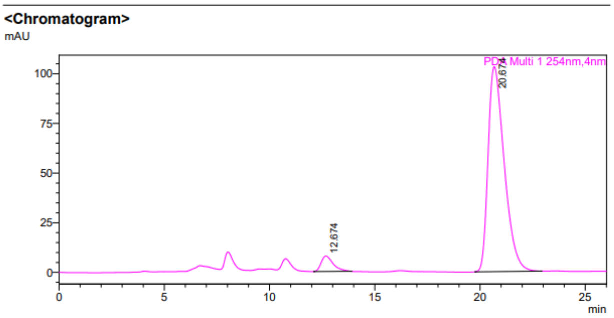

<Peak Table>

PDA Ch1 254nm

Peak#	Ret. Time	Area	Height	Height%	Area%
1	12.674	307209	7759	6.993	5.280
2	20.674	5511281	103205	93.007	94.720
Total		5818490	110965	100.000	100.000

(*R*)-(*E*)-2-(7-ethylquinolin-8-yl)-6-isopropyl-3-styrylisoquinolin-1(2*H*)-one (5c):

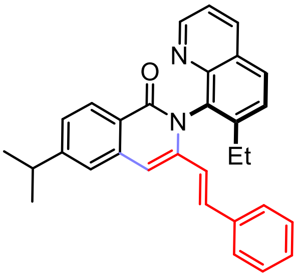

Synthesized according to general procedure GP-B as brownish sticky liquid, eluent (PE:EtOAc = 2:1 v/v), Yield: 70% (62.2 mg), 94% ee.

$[\alpha]_D^{20}$ = 8.412 (c = 0.05, $CHCl_3$)

^{1}H NMR (400 MHz, $CDCl_3$) δ 8.84 (dd, *J* = 4.2, 1.5 Hz, 1H), 8.38 (d, *J* = 8.2 Hz, 1H), 8.18 (dd, *J* = 8.3, 1.8 Hz, 1H), 7.93 (d, *J* = 8.5 Hz, 1H), 7.62 (d, *J* = 8.5 Hz, 1H), 7.49 (s, 1H), 7.40 – 7.34 (m, 2H), 7.20 – 7.17 (m, 3H), 7.07 (d, *J* = 15.9 Hz, 1H), 7.04 – 7.01 (m, 3H), 6.14 (d, *J* = 15.9 Hz, 1H), 3.13 – 3.06 (m, 1H), 2.73 – 2.61 (m, 2H), 1.37 (d, *J* = 6.9 Hz, 6H), 1.24 (t, *J* = 7.6 Hz, 3H).

^{13}C NMR (101 MHz, $CDCl_3$) δ 162.8, 153.9, 151.4, 144.8, 143.6, 141.7, 137.8, 136.4, 136.0, 133.9, 132.5, 128.9, 128.7 (2×C), 128.3, 127.6 (2×C), 126.8, 125.9, 123.9, 123.4, 122.4, 121.2, 104.1, 34.6, 24.9, 23.92, 23.90, 13.8.

HRMS (ESI) m/z: $[M+H^+]^+$ Calculated for $[C_{31}H_{29}N_2O^+]$: 445.2274; found: 445.2280;

HPLC Condition The enantiomeric excess was determined by Daicel Chiralpak IA, Hexanes/IPA = 90/10, 1.0 mL/min, λ = 254 nm, t (minor) = 22.0 min, t (major) = 29.9 min

<Chromatogram>

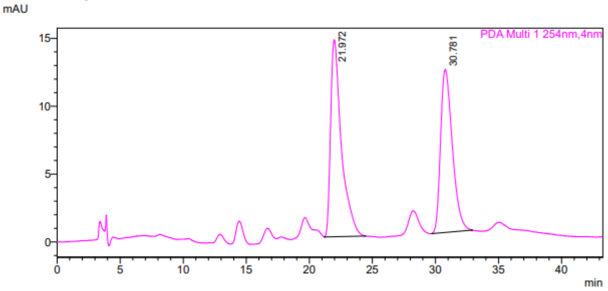

<Peak Table>

PDA Ch1 254nm

Peak#	Ret. Time	Area	Height	Height%	Area%
1	21.972	865093	14520	54.709	52.584
2	30.781	780082	12020	45.291	47.416
Total		1645175	26540	100.000	100.000

<Chromatogram>

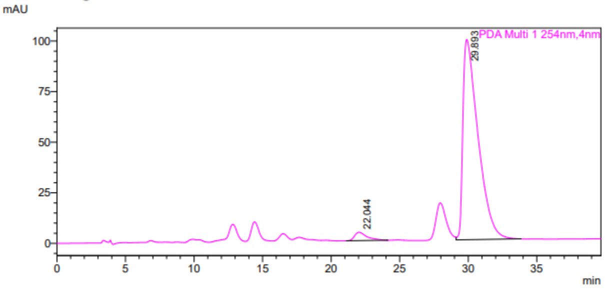

<Peak Table>

PDA Ch1 254nm

Peak#	Ret. Time	Area	Height	Height%	Area%
1	22.044	260291	4133	4.008	3.308
2	29.893	7607848	98971	95.992	96.692
Total		7868139	103103	100.000	100.000

(*R*)-(*E*)-2-(7-ethylquinolin-8-yl)-6-methoxy-3-styrylisoquinolin-1(2*H*)-one (5d):

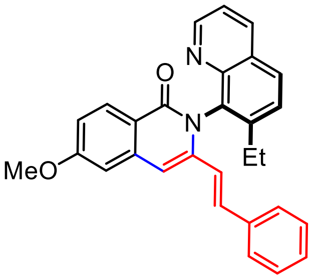

Synthesized according to general procedure GP-B as brownish sticky liquid, eluent (PE:EtOAc = 3:2 v/v), Yield: 65% (59.6 mg), 96% ee.

$[\alpha]_D^{20}$ = 53.625 (c = 0.1, $CHCl_3$)

^{1}H NMR (400 MHz, $CDCl_3$) δ 8.87 – 8.86 (m, 1H), 8.36 (d, *J* = 8.8 Hz, 1H), 8.19 (d, *J* = 8.0 Hz, 1H), 7.93 (d, *J* = 8.5 Hz, 1H), 7.62 (d, *J* = 8.5 Hz, 1H), 7.37 (dd, *J* = 7.6, 4.2 Hz, 1H), 7.17 – 6.98 (m, 9H), 6.12 (d, *J* = 15.9 Hz, 1H), 3.94 (s, 3H), 2.71 – 2.61 (m, 2H), 1.23 (t, *J* = 7.6 Hz, 3H).

^{13}C NMR (101 MHz, $CDCl_3$) δ 163.1, 162.6, 151.4, 144.7, 143.7, 142.3, 139.5, 136.23, 136.20, 133.7, 132.7, 130.6, 129.0, 128.7, 128.4, 127.7, 127.6, 126.8, 122.2, 121.2, 119.6, 116.1, 107.1, 103.9, 55.6, 24.8, 13.8.

HRMS (ESI) m/z: $[M+H^+]^+$ Calculated for $[C_{31}H_{29}N_2O^+]$: 433.1911; found: 433.1914;

HPLC Condition The enantiomeric excess was determined by Daicel Chiralpak ID, Hexanes/IPA = 60/40, 1.0 mL/min, λ = 254 nm, t (minor) = 12.7 min, t (major) = 20.7 min

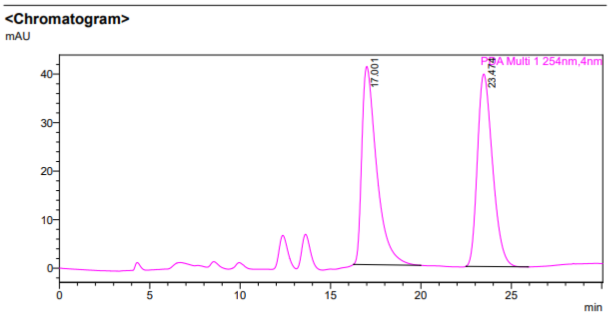

<Peak Table>

PDA Ch1 254nm

Peak#	Ret. Time	Area	Height	Height%	Area%
1	17.001	2333879	40844	50.728	50.230
2	23.474	2312509	39672	49.272	49.770
Total		4646388	80516	100.000	100.000

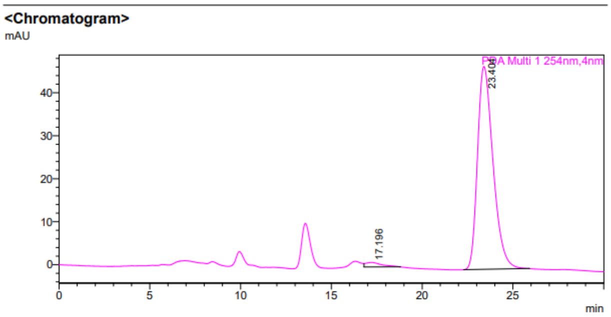

<Peak Table>

PDA Ch1 254nm

Peak#	Ret. Time	Area	Height	Area%	Height%
1	17.196	65191	1048	2.267	2.172
2	23.404	2809958	47208	97.733	97.828
Total		2875150	48256	100.000	100.000

(*R*)-(*E*)-2-(7-ethylquinolin-8-yl)-6-fluoro-3-styrylisoquinolin-1(2*H*)-one (5e):

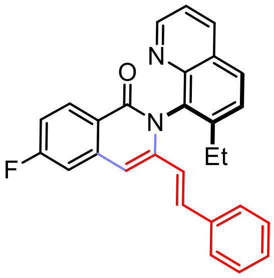

Synthesized according to general procedure GP-B as yellowish oil, eluent (PE:EtOAc = 2:1 v/v), Yield: 55% (46.2 mg), 92% ee.

$[\alpha]_D^{20}$ = 24.405 (c = 0.1, $CHCl_3$)

^{1}H NMR (400 MHz, $CDCl_3$) δ 8.86 (dd, *J* = 4.3, 1.7 Hz, 1H), 8.45 (dd, *J* = 8.8, 5.7 Hz, 1H), 8.20 (dd, *J* = 8.2, 1.7 Hz, 1H), 7.95 (d, *J* = 8.5 Hz, 1H), 7.64 (d, *J* = 8.6 Hz, 1H), 7.38 (dd, *J* = 8.2, 4.2 Hz, 1H), 7.29 – 7.27 (m, 1H), 7.20 – 7.15 (m, 4H), 7.10 (d, *J* = 15.9 Hz, 1H), 7.03 (dd, *J* = 6.7, 2.9 Hz, 2H), 6.98 (s, 1H), 6.12 (d, *J* = 15.9 Hz, 1H), 2.72 – 2.60 (m, 2H), 1.25 (t, *J* = 7.6 Hz, 3H).

^{13}C NMR (101 MHz, $CDCl_3$) δ 165.7 (d, *J* = 252.2 Hz), 162.2, 151.5, 144.7, 143.6, 143.2, 139.8 (d, *J* = 10.5 Hz), 136.14, 136.09, 133.5, 133.4, 131.9 (d, *J* = 10.0 Hz), 129.2, 128.7, 128.6, 127.7 (2×C), 126.9, 122.3 (d, *J* = 1.7 Hz), 121.9, 121.3, 115.2 (d, *J* = 23.5 Hz), 110.9 (d, *J* = 21.8 Hz), 103.3 (d, *J* = 3.4 Hz), 24.9, 13.8.

^{19}F NMR (471 MHz, $CDCl_3$) δ -106.6.

HRMS (ESI) m/z: $[M+H^+]^+$ Calculated for $[C_{28}H_{22}FN_2O^+]$: 421.1711; found: 421.1739;

HPLC Condition The enantiomeric excess was determined by Daicel Chiralpak IB, Hexanes/IPA = 85/15, 1.0 mL/min, λ = 254 nm, t (minor) = 36.8 min, t (major) = 18.1 min

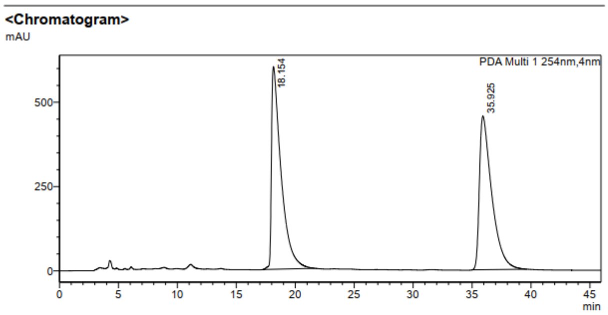

<Peak Table>

PDA Ch1 254nm

Peak#	Ret. Time	Area	Height	Area%
1	18.154	33995443	601072	50.558
2	35.925	33245478	455568	49.442
Total		67240921	1056640	100.000

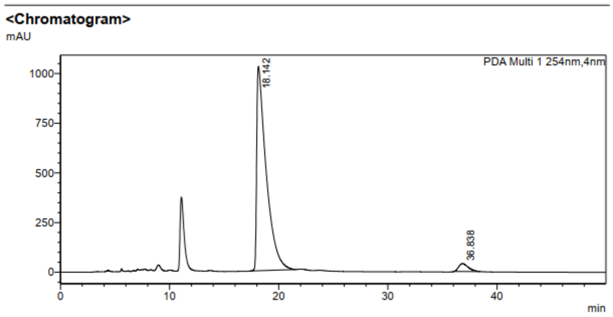

<Peak Table>

PDA Ch1 254nm

Peak#	Ret. Time	Area	Height	Area%
1	18.142	62253435	1027481	96.244
2	36.838	2429379	39793	3.756
Total		64682814	1067274	100.000

(*R*)-(*E*)-6-bromo-2-(7-ethylquinolin-8-yl)-3-styrylisoquinolin-1(2*H*)-one (5f):

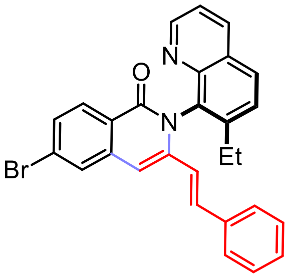

Synthesized according to general procedure GP-B as brownish oil, eluent (PE:EtOAc = 2:1 v/v), Yield: 75% (72.2 mg), 92% ee.

$[\alpha]_D^{20}$ = -7.609 (c = 0.05, $CHCl_3$)

^{1}H NMR (400 MHz, $CDCl_3$) δ 8.84 (dd, *J* = 4.3, 1.7 Hz, 1H), 8.28 (d, *J* = 8.5 Hz, 1H), 8.19 (dd, *J* = 8.2, 1.7 Hz, 1H), 7.94 (d, *J* = 8.5 Hz, 1H), 7.80 (d, *J* = 1.9 Hz, 1H), 7.62 (d, *J* = 8.5 Hz, 1H), 7.56 (dd, *J* = 8.6, 1.9 Hz, 1H), 7.37 (dd, *J* = 8.2, 4.2 Hz, 1H), 7.19 – 7.17 (m, 3H), 7.08 (d, *J* = 15.9 Hz, 1H), 7.02 (dd, *J* = 6.6, 3.0 Hz, 2H), 6.93 (s, 1H), 6.11 (d, *J* = 15.9 Hz, 1H), 2.71 – 2.59 (m, 2H), 1.24 (t, *J* = 7.6 Hz, 3H).

^{13}C NMR (101 MHz, $CDCl_3$) δ 162.4, 151.5, 144.6, 143.5, 143.2, 139.0, 136.1, 136.0, 133.52, 133.47, 130.4, 129.7, 129.2, 128.72, 128.65, 128.6, 127.8, 127.6 (2×C), 126.9, 124.2, 121.8, 121.3, 102.7, 24.9, 13.8.

HRMS (ESI) m/z: $[M+H^+]^+$ Calculated for $[C_{28}H_{22}BrN_2O^+]$: 481.0910; found: 481.0906;

HPLC Condition The enantiomeric excess was determined by Daicel Chiralpak IB, Hexanes/IPA = 60/40, 1.0 mL/min, λ = 254 nm, t (minor) = 21.6 min, t (major) = 9.6 min

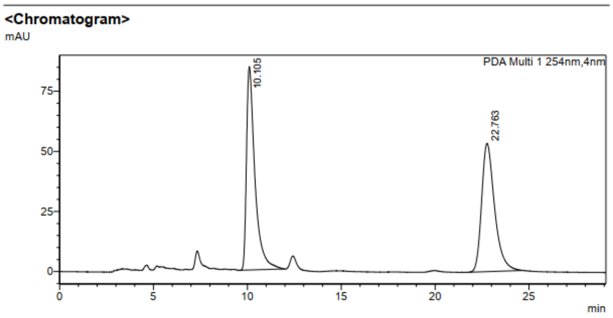

<Peak Table>

PDA Ch1 254nm

Peak#	Ret. Time	Area	Height	Area%
1	10.105	2637613	84588	50.662
2	22.763	2568690	53377	49.338
Total		5206302	137965	100.000

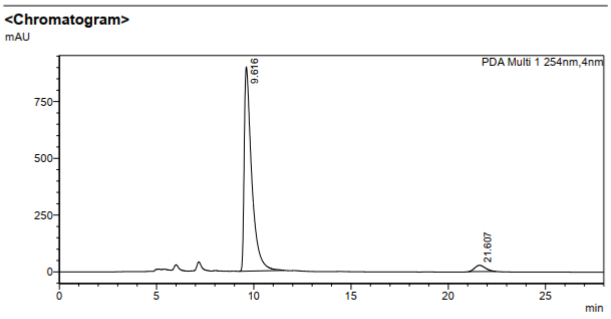

<Peak Table>

PDA Ch1 254nm

Peak#	Ret. Time	Area	Height	Area%
1	9.616	24113848	899341	95.847
2	21.607	1044786	27483	4.153
Total		25158634	926824	100.000

(*R*)-(*E*)-2-(7-ethylquinolin-8-yl)-6-iodo-3-styrylisoquinolin-1(2*H*)-one (5g):

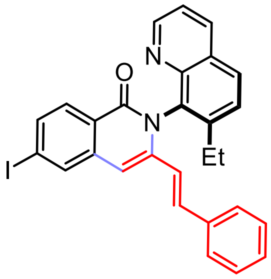

Synthesized according to general procedure GP-B as brownish sticky liquid, eluent (PE:EtOAc = 2:1 v/v), Yield: 82% (68.7 mg), 94% ee.

$[\alpha]_D^{20}$ = 17.605 (c = 0.1, $CHCl_3$)

^{1}H NMR (400 MHz, $CDCl_3$) δ 8.84 (dd, *J* = 4.3, 1.7 Hz, 1H), 8.19 (dd, *J* = 8.2, 1.7 Hz, 1H), 8.12 (d, *J* = 8.5 Hz, 1H), 8.04 (d, *J* = 1.7 Hz, 1H), 7.94 (d, *J* = 8.5 Hz, 1H), 7.76 (dd, *J* = 8.4, 1.7 Hz, 1H), 7.62 (d, *J* = 8.6 Hz, 1H), 7.37 (dd, *J* = 8.2, 4.2 Hz, 1H), 7.19 – 7.17 (m, 3H), 7.07 (d, *J* = 15.9 Hz, 1H), 7.01 (dd, *J* = 6.6, 3.0 Hz, 2H), 6.90 (s, 1H), 6.10 (d, *J* = 15.9 Hz, 1H), 2.70 – 2.58 (m, 2H), 1.23 (t, *J* = 7.6 Hz, 3H).

^{13}C NMR (101 MHz, $CDCl_3$) δ 162.6, 151.5, 144.6, 143.5, 143.1, 139.1, 136.14, 136.06, 135.4, 135.0, 133.47, 133.48, 130.1, 129.2, 128.7, 128.6, 127.67, 127.65, 126.9, 124.7, 121.9, 121.3, 102.5, 100.5, 24.9, 13.8.

HRMS (ESI) m/z: $[M+H^+]^+$ Calculated for $[C_{28}H_{22}IN_2O^+]$: 529.0771; found: 529.0789;

HPLC Condition The enantiomeric excess was determined by Daicel Chiralpak IB, Hexanes/IPA = 70/30, 1.0 mL/min, λ = 254 nm, t (minor) = 24.7 min, t (major) = 10.5 min

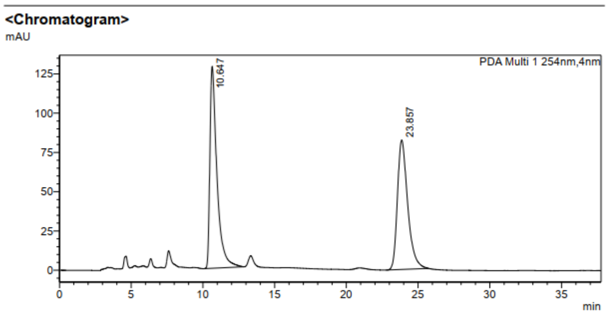

<Peak Table>

PDA Ch1 254nm

Peak#	Ret. Time	Area	Height	Area%
1	10.647	4389771	128296	51.614
2	23.857	4115258	82378	48.386
Total		8505029	210674	100.000

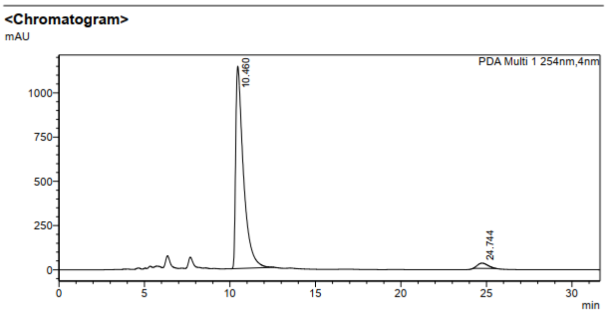

<Peak Table>

PDA Ch1 254nm

Peak#	Ret. Time	Area	Height	Area%
1	10.460	36153718	1142795	96.657
2	24.744	1250415	29669	3.343
Total		37404132	1172464	100.000

(*R*)-methyl (*E*)-2-(7-ethylquinolin-8-yl)-1-oxo-3-styryl-1,2-dihydroisoquinoline-6-carboxylate (5h):

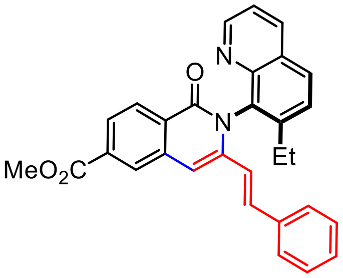

Synthesized according to general procedure GP-B as yellowish sticky liquid, eluent (PE:EtOAc = 2:1 v/v), Yield: 65% (59.9 mg), 90% ee.

$[\alpha]_D^{20}$ = 18.424 (c = 0.1, $CHCl_3$)

^{1}H NMR (500 MHz, $CDCl_3$) δ 8.83 (dd, *J* = 4.2, 1.7 Hz, 1H), 8.48 (d, *J* = 8.3 Hz, 1H), 8.37 (d, *J* = 1.6 Hz, 1H), 8.20 (dd, *J* = 8.3, 1.8 Hz, 1H), 8.06 (dd, *J* = 8.4, 1.7 Hz, 1H), 7.94 (d, *J* = 8.5 Hz, 1H), 7.63 (d, *J* = 8.5 Hz, 1H), 7.37 (dd, *J* = 8.3, 4.2 Hz, 1H), 7.19 – 7.17 (m, 3H), 7.12 – 7.09 (m, 2H), 7.04 – 7.02 (m, 2H), 6.12 (d, *J* = 16.0 Hz, 1H), 4.01 (s, 3H), 2.70 – 2.60 (m, 2H), 1.24 (t, *J* = 7.6 Hz, 3H).

^{13}C NMR (126 MHz, $CDCl_3$) δ 166.7, 162.4, 151.5, 144.6, 143.5, 142.8, 137.4, 136.1, 133.8, 133.5, 133.4, 129.2, 129.0, 128.7, 128.6, 128.4, 128.3, 127.7 (2×C), 126.9, 126.4, 121.9, 121.3 (2xC), 103.8, 52.6, 24.9, 13.8.

HRMS (ESI) m/z: $[M+H^+]^+$ Calculated for $[C_{30}H_{25}N_2O_3^+]$: 461.1860; found: 461.1864;

HPLC Condition The enantiomeric excess was determined by Daicel Chiralpak IH, Hexanes/IPA = 95/5, 1.0 mL/min, λ = 254 nm, t (minor) = 37.9 min, t (major) = 29.3 min

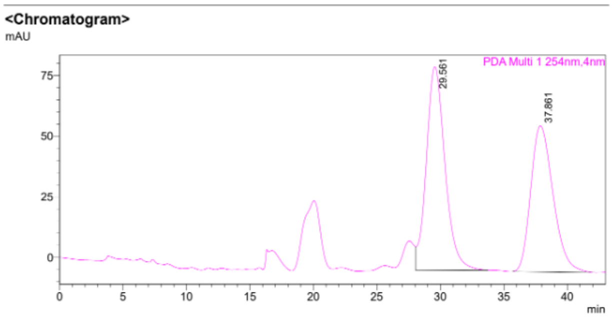

<Peak Table>

PDA Ch1 254nm

Peak#	Ret. Time	Area	Height	Height%	Area%
1	29.561	8584349	83856	58.214	53.835
2	37.861	7361243	60191	41.786	46.165
Total		15945592	144047	100.000	100.000

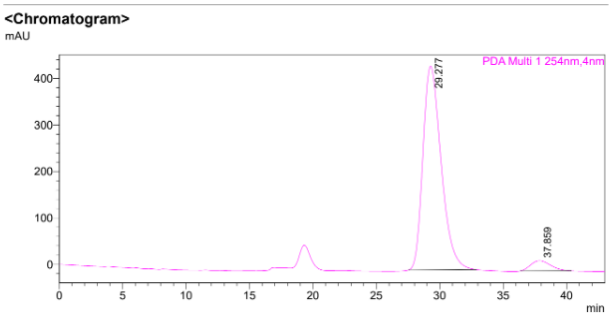

<Peak Table>

PDA Ch1 254nm

Peak#	Ret. Time	Area	Height	Height%	Area%
1	29.277	43778658	437724	95.227	94.822
2	37.859	2390879	21941	4.773	5.178
Total		46169537	459665	100.000	100.000

(*R*)-(*E*)-6-acetyl-2-(7-ethylquinolin-8-yl)-3-styrylisoquinolin-1(2*H*)-one (5i):

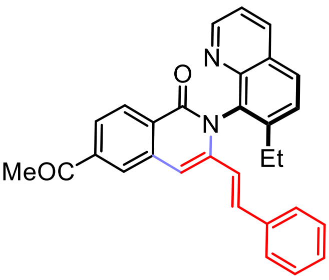

Synthesized according to general procedure GP-B as yellowish sticky liquid, eluent (PE:EtOAc = 2:1 v/v), Yield: 83% (73.8 mg), 96% ee.

$[\alpha]_D^{20}$ = 8.000 (c = 0.05, $CHCl_3$).

^{1}H NMR (400 MHz, $CDCl_3$) δ 8.82 (dd, *J* = 4.2, 1.7 Hz, 1H), 8.50 (d, *J* = 8.3 Hz, 1H), 8.22 (s, 1H), 8.18 (dd, *J* = 8.2, 1.7 Hz, 1H), 7.99 – 7.93 (m, 2H), 7.63 (d, *J* = 8.5 Hz, 1H), 7.35 (dd, *J* = 8.2, 4.2 Hz, 1H), 7.18 – 7.17 (m, 3H), 7.11 (t, *J* = 8.0 Hz, 2H), 7.02 (dd, *J* = 6.7, 2.9 Hz, 2H), 6.13 (d, *J* = 15.9 Hz, 1H), 2.71 – 2.62 (m, 5H), 1.24 (t, *J* = 7.6 Hz, 3H).

^{13}C NMR (101 MHz, $CDCl_3$) δ 198.0, 162.3, 151.4, 144.5, 143.4, 142.8, 140.1, 137.5, 136.1, 136.0, 133.42, 133.43, 129.2, 129.1, 128.7, 128.6, 128.1, 127.6 (2×C), 127.0, 126.8, 125.1, 121.7, 121.3, 103.9, 27.1, 24.8, 13.8.

HRMS (ESI) m/z: $[M+H^+]^+$ Calculated for $[C_{30}H_{25}N_2O_2^+]$: 445.1911; found: 445.1910;

HPLC Condition The enantiomeric excess was determined by Daicel Chiralpak IB, Hexanes/IPA = 70/30, 1.0 mL/min, λ = 254 nm, t (minor) = 30.7 min, t (major) = 25.5 min

<Chromatogram>

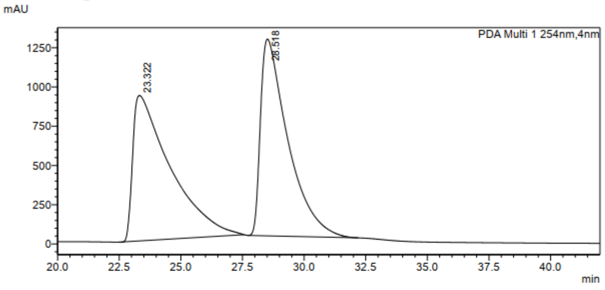

<Peak Table>

PDA Ch1 254nm

Peak#	Ret. Time	Area	Height	Area%
1	23.322	98276409	927011	50.364
2	28.518	96854200	1253545	49.636
Total		195130610	2180557	100.000

<Chromatogram>

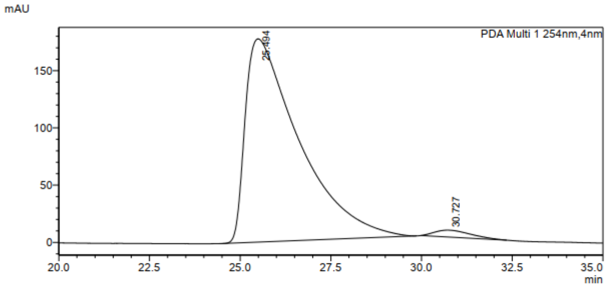

<Peak Table>

PDA Ch1 254nm

Peak#	Ret. Time	Area	Height	Area%
1	25.494	18361033	177569	97.785
2	30.727	415928	5953	2.215
Total		18776961	183522	100.000

(*R*)-(*E*)-2-(7-ethylquinolin-8-yl)-7-methyl-3-styrylisoquinolin-1(2*H*)-one (5j):

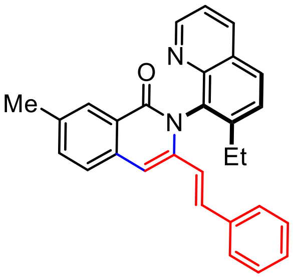

Synthesized according to general procedure GP-B as brownish sticky liquid, eluent (PE:EtOAc = 2:1 v/v), Yield: 71% (59.1 mg), 92% ee.

$[\alpha]_D^{20}$ = -30.431 (c = 0.1, $CHCl_3$)

^{1}H NMR (500 MHz, $CDCl_3$) δ 8.89 (dd, *J* = 4.3, 1.7 Hz, 1H), 8.27 (s, 1H), 8.20 (dd, *J* = 8.2, 1.7 Hz, 1H), 7.93 (d, *J* = 8.5 Hz, 1H), 7.63 (d, *J* = 8.6 Hz, 1H), 7.57 – 7.51 (m, 2H), 7.38 (dd, *J* = 8.3, 4.2 Hz, 1H), 7.19 – 7.15 (m, 3H), 7.07 – 7.01 (m, 4H), 6.12 (d, *J* = 15.9 Hz, 1H), 2.71 – 2.60 (m, 2H), 2.50 (s, 3H), 1.24 (t, *J* = 7.6 Hz, 3H).

^{13}C NMR (126 MHz, $CDCl_3$) δ 162.9, 151.4, 144.7, 143.7, 140.8, 136.7, 136.4, 136.3, 135.2, 134.2, 132.3 130.5, 129.0, 128.7 (2×C), 128.3, 128.2, 127.73, 127.70, 126.8 (2×C), 126.3, 125.7, 122.3, 121.2, 104.1, 24.9, 21.6, 13.8

HRMS (ESI) m/z: $[M+H^+]^+$ Calculated for $[C_{29}H_{25}N_2O^+]$: 417.1961; found: 417.1967;

HPLC Condition The enantiomeric excess was determined by Daicel Chiralpak IB, Hexanes/IPA = 80/20, 1.0 mL/min, λ = 230 nm, t (minor) = 23.2 min, t (major) = 11.1 min.

<Chromatogram>

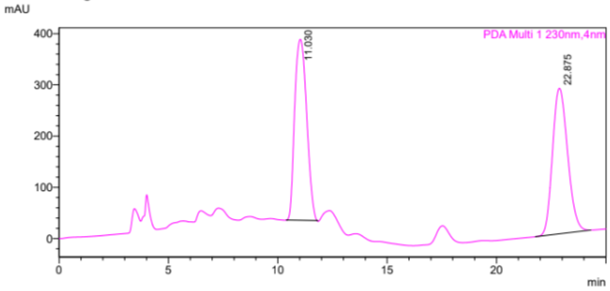

<Peak Table>

PDA Ch1 230nm

Peak#	Ret. Time	Area	Height	Height%	Area%
1	11.030	13479015	353292	55.420	49.627
2	22.875	13681574	284188	44.580	50.373
Total		27160589	637480	100.000	100.000

<Chromatogram>

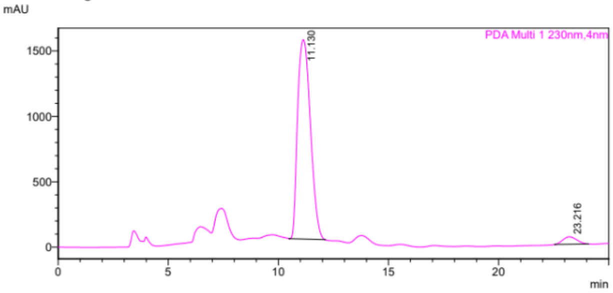

<Peak Table>

PDA Ch1 230nm

Peak#	Ret. Time	Area	Height	Height%	Area%
1	11.130	61806626	1523468	96.422	96.093
2	23.216	2512960	56536	3.578	3.907
Total		64319586	1580004	100.000	100.000

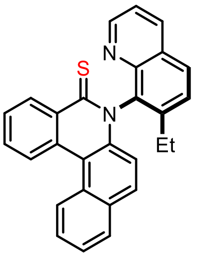

(*R*)-6-(7-ethylquinolin-8-yl)benzo[*a*]phenanthridine-5(6*H*)-thione (6a):

Synthesized according to procedure for compound **6** as light-yellow oil, eluent (PE:EtOAc = 4:1 v/v), Yield: 60% (25 mg), 94% ee

$[\alpha]_D^{20}$ = -35.177 (c = 0.1, $CHCl_3$)

^{1}H NMR (400 MHz, $CDCl_3$) δ 9.37 (dd, *J* = 8.3, 1.5 Hz, 1H), 8.91 (d, *J* = 8.7 Hz, 1H), 8.81 (d, *J* = 8.3 Hz, 1H), 8.71 (s, 1H), 8.26 (d, *J* = 8.1 Hz, 1H), 8.04 (d, *J* = 8.5 Hz, 1H), 7.90 – 7.83 (m, 2H), 7.74 (d, *J* = 8.5 Hz, 1H), 7.70 – 7.64 (m, 2H), 7.58 – 7.52 (m, 2H), 7.36 (dd, *J* = 8.3, 3.7 Hz, 1H), 6.67 (d, *J* = 9.1 Hz, 1H), 2.62 – 2.55 (m, 1H), 2.48 – 2.42 (m, 1H), 1.17 (t, *J* = 7.6 Hz, 3H).

^{13}C NMR (101 MHz, $CDCl_3$) δ 187.0, 151.2, 143.6, 143.1, 137.9, 137.6, 136.6, 134.0, 133.8, 132.1, 131.5, 130.3, 130.0, 129.9, 129.0, 128.7, 128.3, 128.0 (2×C), 127.50, 127.47, 127.1, 125.7, 121.4, 117.6, 117.3, 24.6, 13.1.

HRMS (ESI) m/z: $[M+H^+]^+$ Calculated for $[C_{28}H_{21}N_2S^+]$: 417.1420; found: 417.1410;

HPLC Condition The enantiomeric excess was determined by Daicel Chiralpak IB, Hexanes/IPA = 70/30, 1.0 mL/min, λ = 254 nm, t (minor) = 10.2 min, t (major) = 14.8 min.

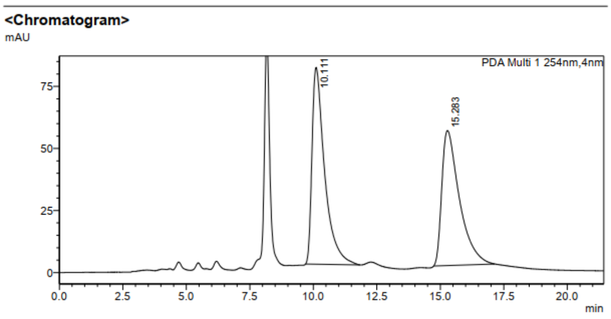

<Peak Table>

PDA Ch1 254nm

Peak#	Ret. Time	Area	Height	Area%
1	10.111	2749758	79272	51.147
2	15.283	2626448	54386	48.853
Total		5376206	133658	100.000

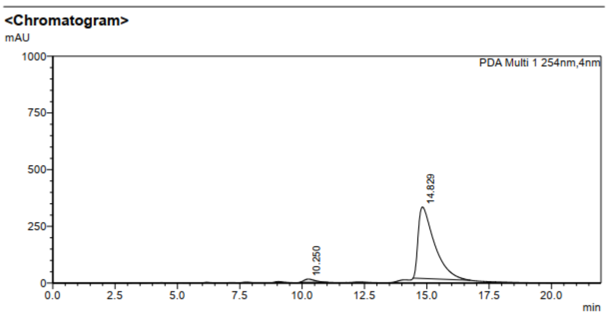

<Peak Table>

PDA Ch1 254nm

Peak#	Ret. Time	Area	Height	Area%
1	10.250	435690	14081	2.943
2	14.829	14369687	314988	97.057
Total		14805377	329069	100.000

(2-(2-((7-ethylquinolin-8-yl)amino)naphthalen-1-yl)phenyl)methanol (7a):

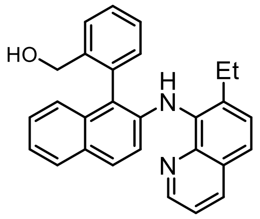

Synthesized according to procedure for compound **7** as light-yellow oil, eluent (PE:EtOAc = 4:1 v/v), Yield: 53% (21.4 mg), 84% ee

$[\alpha]_D^{20}$ = 36.812 (c = 0.1, $CHCl_3$)

^{1}H NMR (400 MHz, $CDCl_3$) δ 8.70 – 8.68 (m, 1H), 8.14 (dt, *J* = 8.2, 1.9 Hz, 1H), 7.82 – 7.79 (m, 1H), 7.72 – 7.65 (m, 2H), 7.60 – 7.53 (m, 4H), 7.47 – 7.44 (m, 1H), 7.34 (ddd, *J* = 8.2, 4.3, 1.8 Hz, 1H), 7.28 – 7.27 (m, 1H), 7.25 – 7.20 (m, 1H), 7.14 (d, *J* = 8.3 Hz, 1H), 6.62 (dd, *J* = 8.9, 1.8 Hz, 1H), 6.07 (bs, 1H), 4.51 – 4.37 (m, 2H), 2.90 – 2.72 (m, 2H), 1.21 (td, *J* = 7.5, 1.8 Hz, 3H).

^{13}C NMR (101 MHz, $CDCl_3$) δ 149.8, 144.7, 142.3, 142.2, 136.8, 136.1, 135.1, 134.2, 131.8, 131.4, 129.1, 129.0, 128.6, 128.5, 128.2, 128.1, 128.0, 126.6, 125.0, 124.3, 123.1, 122.5, 120.9, 119.6, 115.0, 63.0, 25.6, 14.4.

HRMS (ESI) m/z: $[M+H^+]^+$ Calculated for $[C_{28}H_{25}N_2O+]$: 405.1961; found: 405.1951;

HPLC Condition The enantiomeric excess was determined by Daicel Chiralpak IA, Hexanes/IPA = 90/10, 1.0 mL/min, λ = 254 nm, t (minor) = 9.8 min, t (major) = 8.7 min.

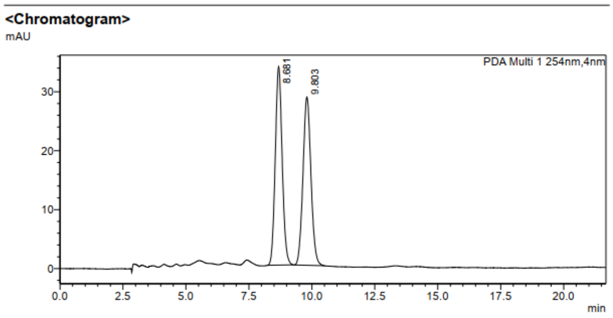

<Peak Table>
PDA Ch1 254nm

Peak#	Ret. Time	Area	Height	Area%
1	8.681	641919	33670	50.486
2	9.803	629572	28550	49.514
Total		1271491	62220	100.000

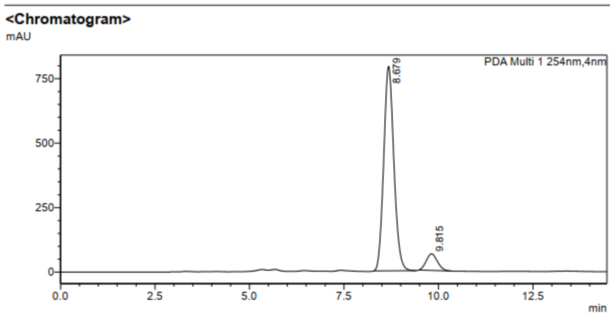

<Peak Table>
PDA Ch1 254nm

Peak#	Ret. Time	Area	Height	Area%
1	8.679	14843605	791732	91.808
2	9.815	1324418	64318	8.192
Total		16168023	856049	100.000

NMR SPECTRA

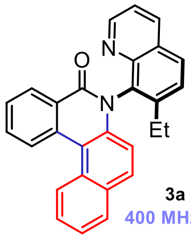

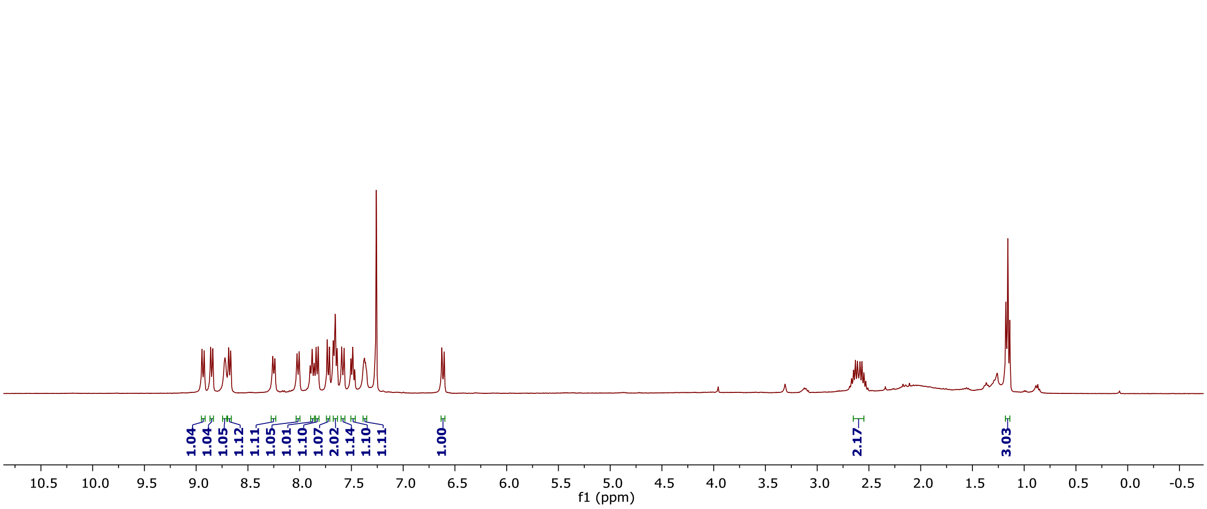

3a

400 MHz, $CDCl_3$

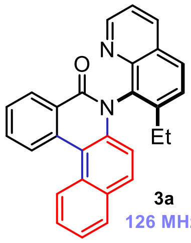

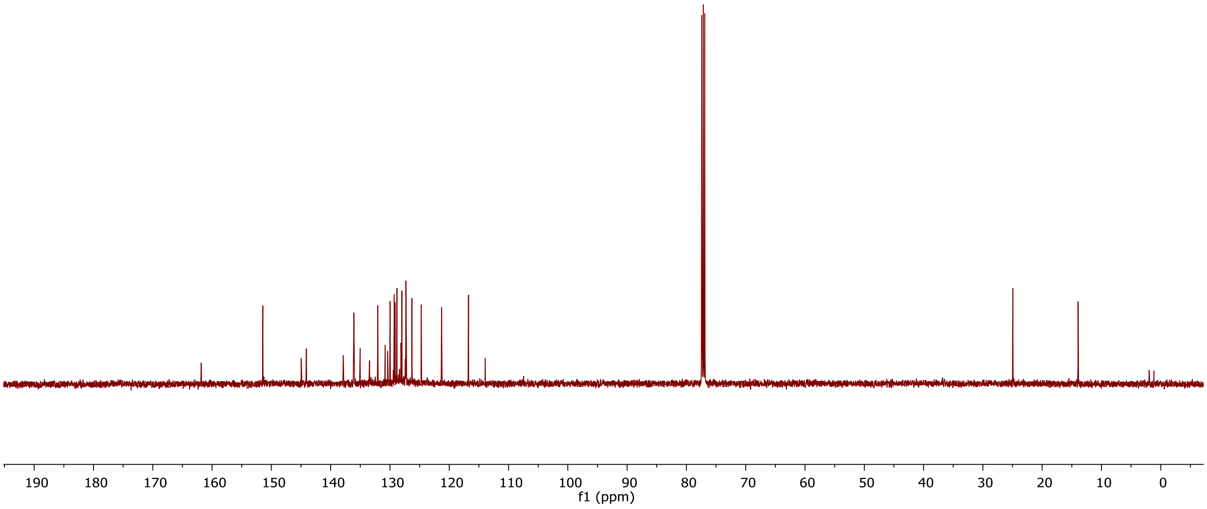

3a

126 MHz, $CDCl_3$

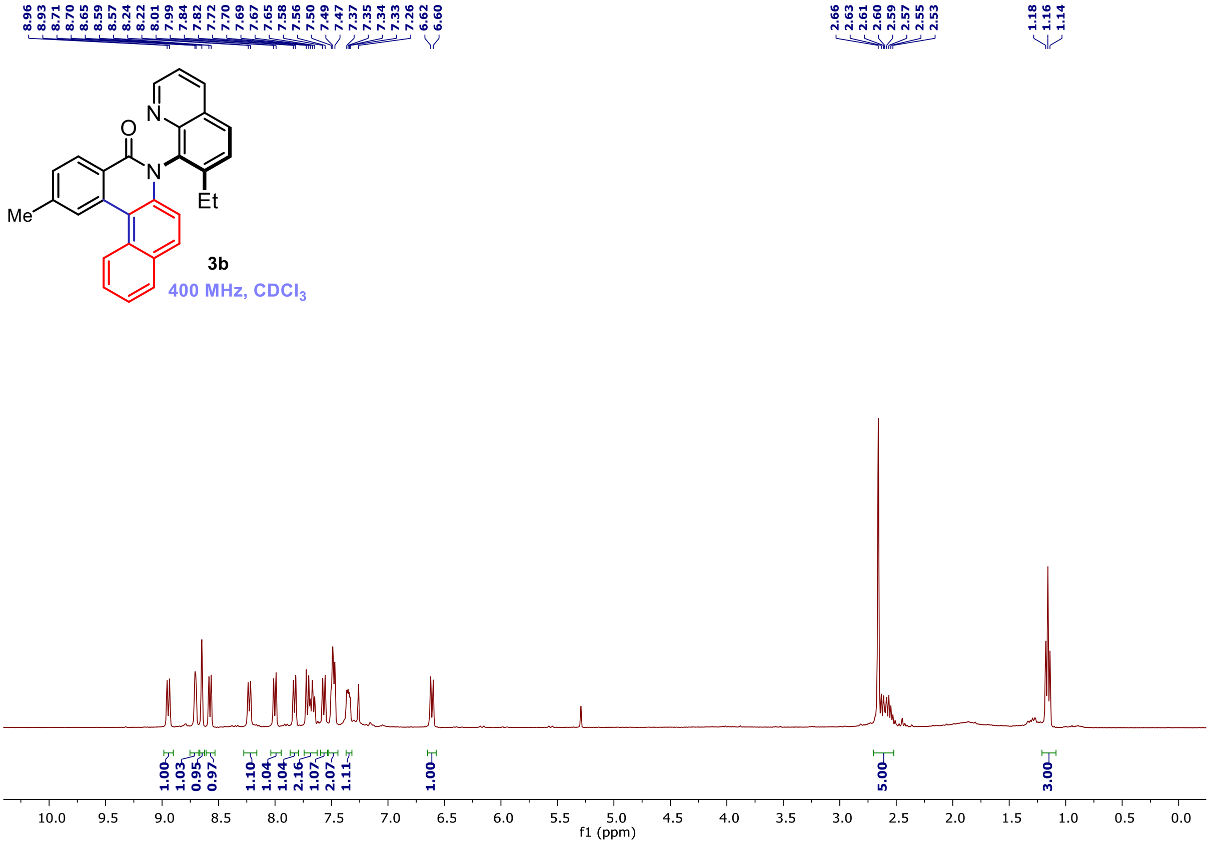
8.96 8.93 8.71 8.70 8.65 8.59 8.57 8.24 8.22 8.01 7.99 7.84 7.82 7.72 7.70 7.69 7.67 7.65 7.58 7.56 7.50 7.49 7.47 7.37 7.35 7.34 7.33 7.26 6.62 6.60
2.66 2.63 2.61 2.60 2.59 2.57 2.55 2.53
1.18 1.16 1.14
Me
N
O
Et
3b
400 MHz, CDCl3
1.00 1.03 0.95 0.97 1.10 1.04 1.04 2.16 1.07 2.07 1.11 1.00 5.00 3.00
f1 (ppm)

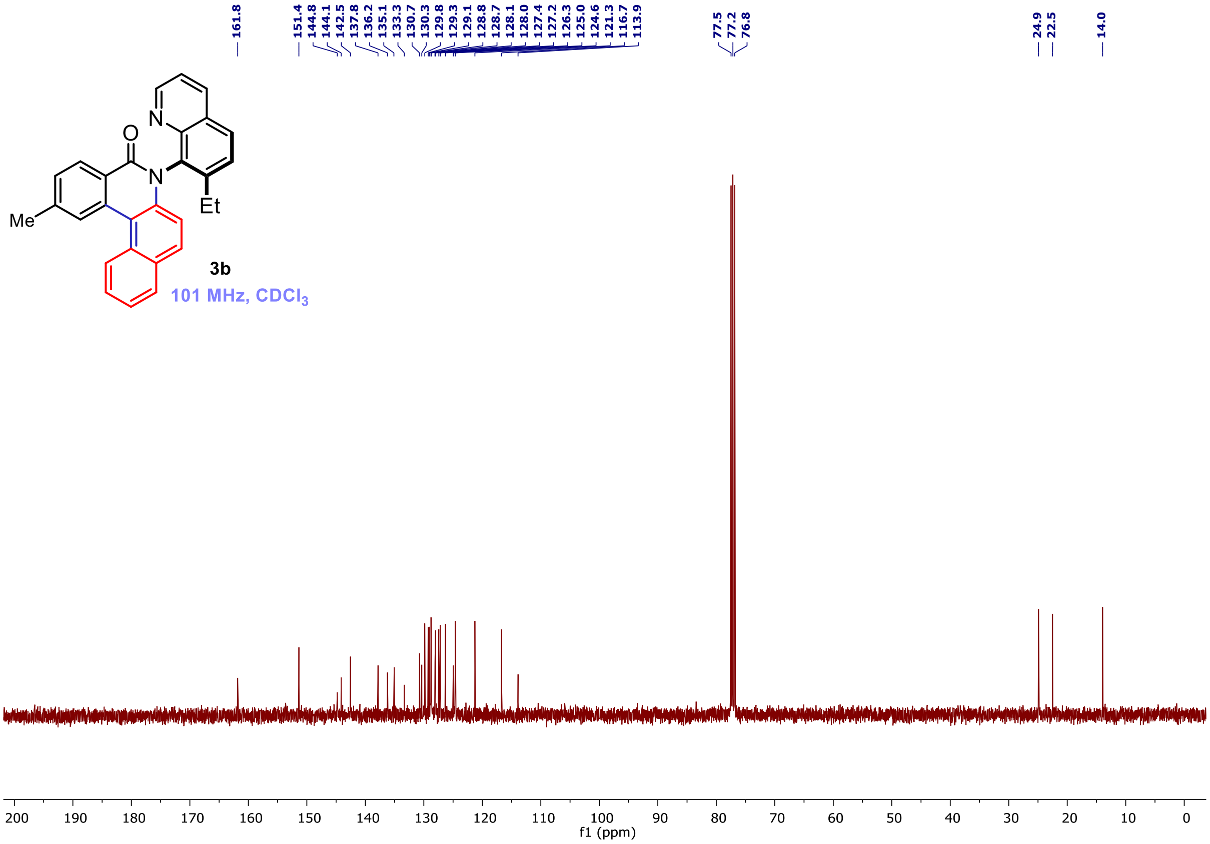
161.8
151.4 144.8 144.1 142.5 137.8 136.2 135.1 133.3 130.7 130.3 129.8 129.3 129.1 128.8 128.7 128.1 128.0 127.4 127.2 126.3 125.0 124.6 121.3 116.7 113.9
77.5 77.2 76.8
24.9 22.5 14.0
Me
N
O
Et
3b
101 MHz, CDCl3
f1 (ppm)

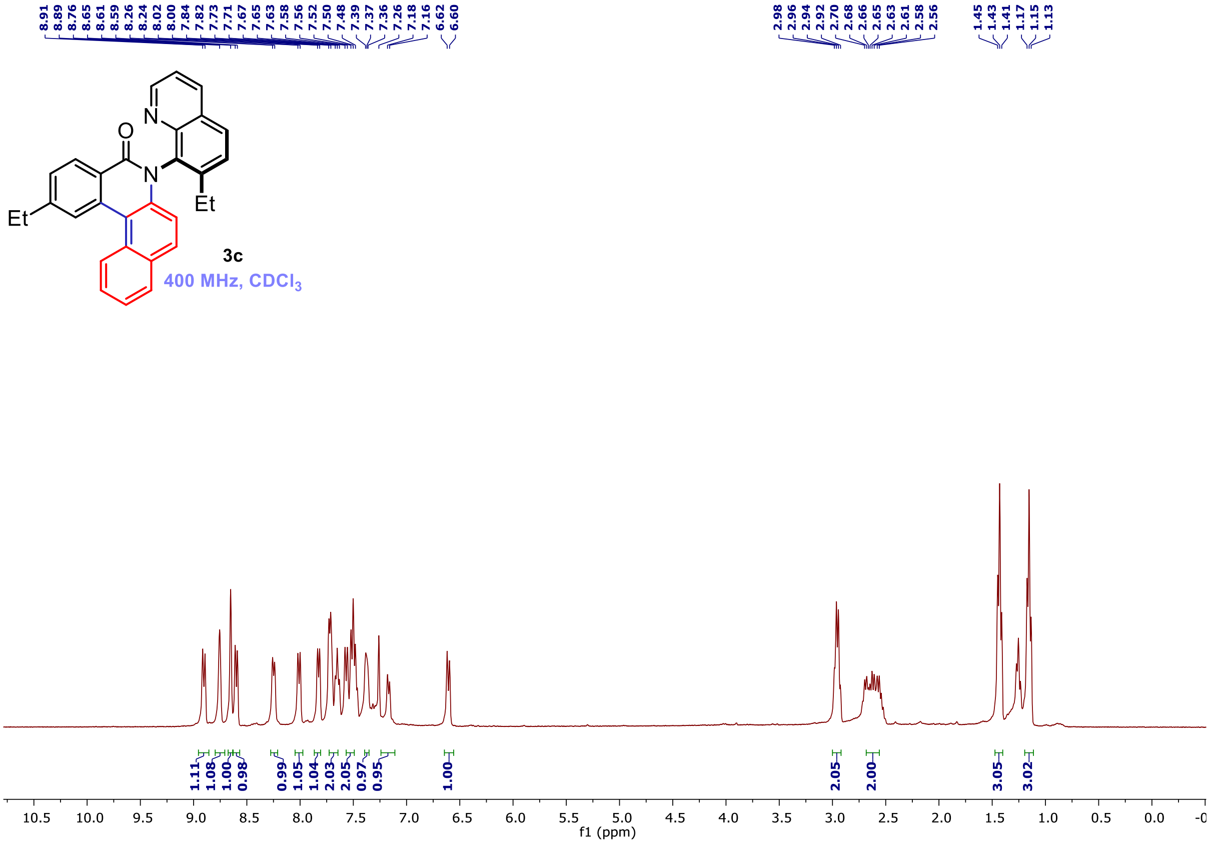
8.91 8.89 8.76 8.65 8.61 8.59 8.26 8.24 8.02 8.00 7.84 7.82 7.73 7.71 7.67 7.65 7.63 7.58 7.56 7.52 7.50 7.48 7.39 7.37 7.36 7.26 7.18 7.16 6.62 6.60
2.98 2.96 2.94 2.92 2.70 2.68 2.66 2.65 2.63 2.61 2.58 2.56
1.45 1.43 1.41 1.17 1.15 1.13
O
N
N
Et
Et
3c
400 MHz, CDCl3
1.11 1.08 1.00 0.98 0.99 1.05 1.04 2.03 2.05 0.97 0.95 1.00 2.05 2.00 3.05 3.02
f1 (ppm)

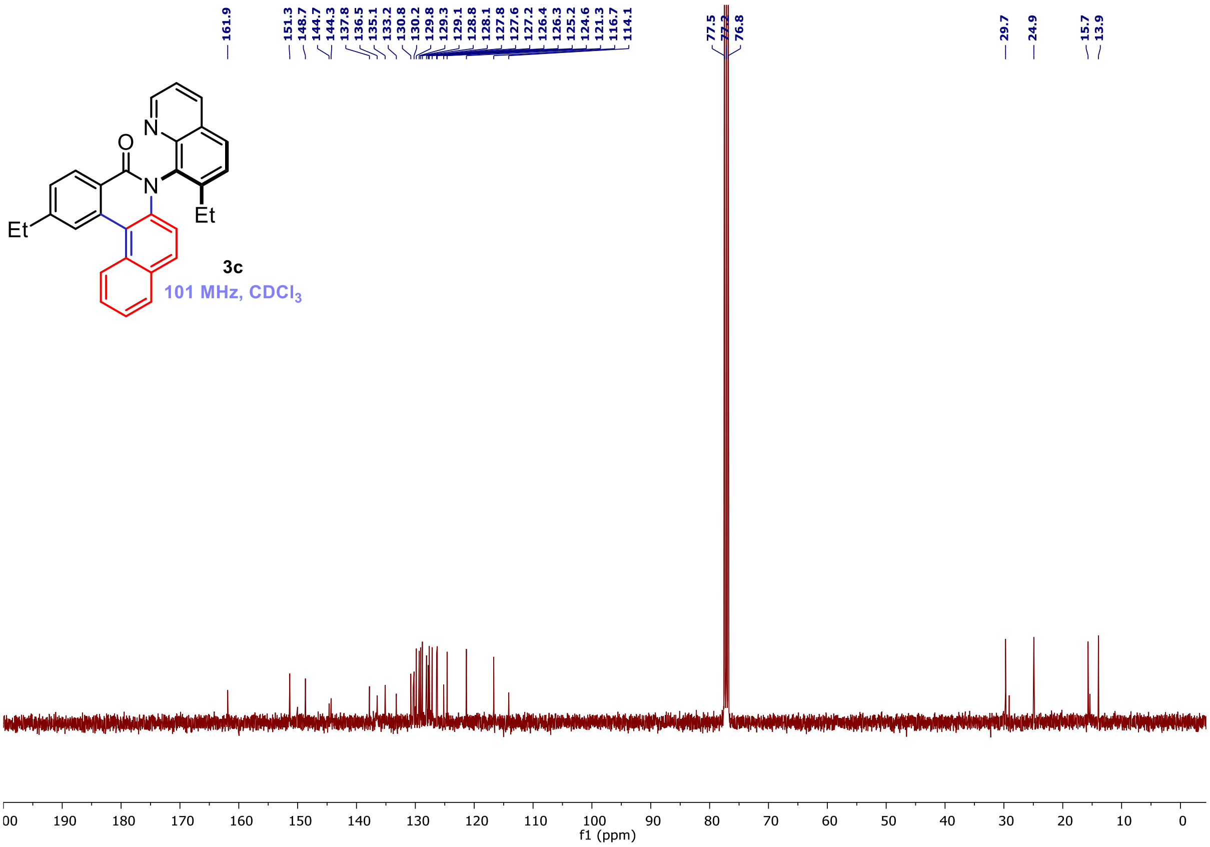
161.9
151.3 148.7 144.7 144.3 137.8 136.5 135.1 133.2 130.8 130.2 129.8 129.3 129.1 128.8 128.1 127.8 127.6 127.2 126.4 126.3 125.2 124.6 121.3 116.7 114.1
77.5 77.2 76.8
29.7 24.9 15.7 13.9
O
N
N
Et
Et
3c
101 MHz, CDCl3
f1 (ppm)

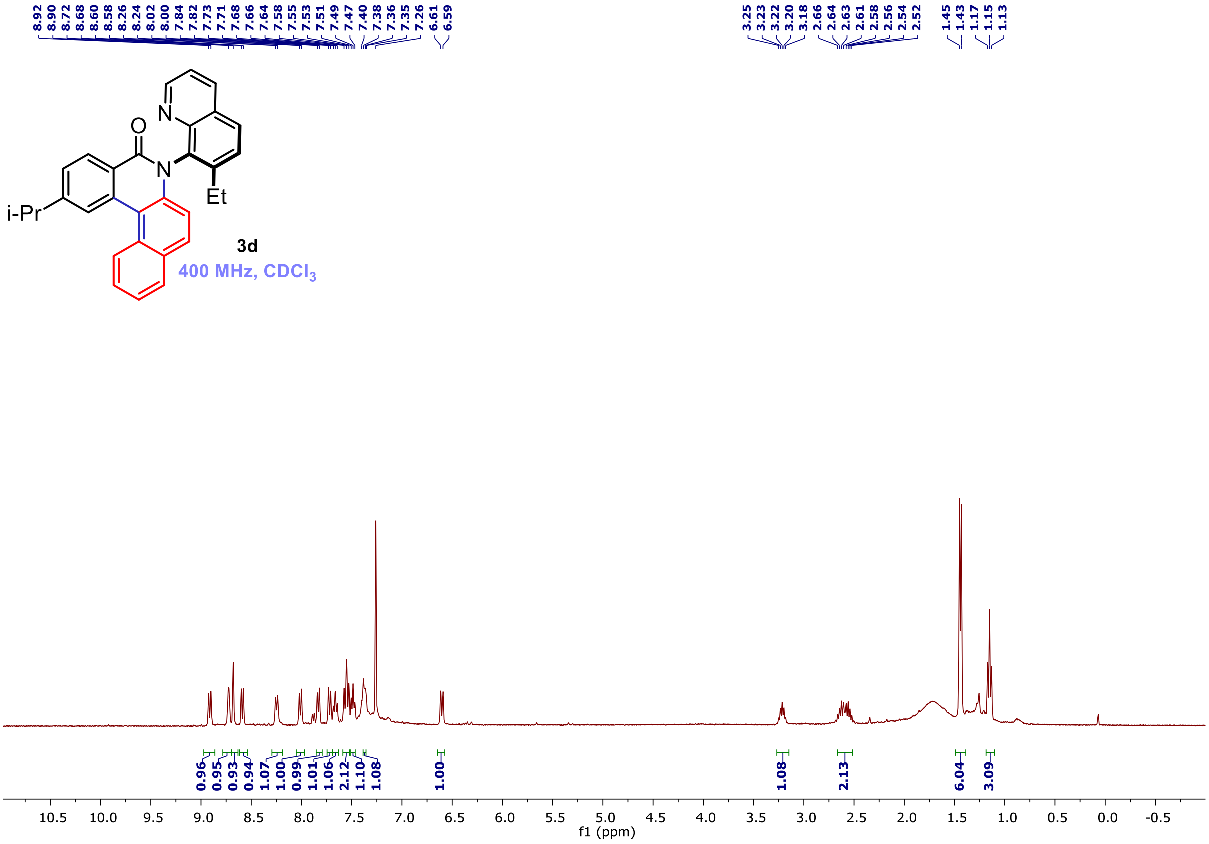
8.92 8.90 8.72 8.68 8.60 8.58 8.26 8.24 8.02 8.00 7.84 7.82 7.73 7.71 7.68 7.66 7.64 7.58 7.55 7.53 7.51 7.49 7.47 7.40 7.38 7.36 7.35 7.26 6.61 6.59
3.25 3.23 3.22 3.20 3.18 2.66 2.64 2.63 2.61 2.58 2.56 2.54 2.52 1.45 1.43 1.17 1.15 1.13
O
N
N
Et
i-Pr
3d
400 MHz, CDCl3
0.96 0.95 0.93 0.94 1.07 1.00 0.99 1.01 1.06 2.12 1.10 1.08 1.00 1.08 2.13 6.04 3.09
10.5 10.0 9.5 9.0 8.5 8.0 7.5 7.0 6.5 6.0 5.5 5.0 4.5 4.0 3.5 3.0 2.5 2.0 1.5 1.0 0.5 0.0 -0.5
f1 (ppm)

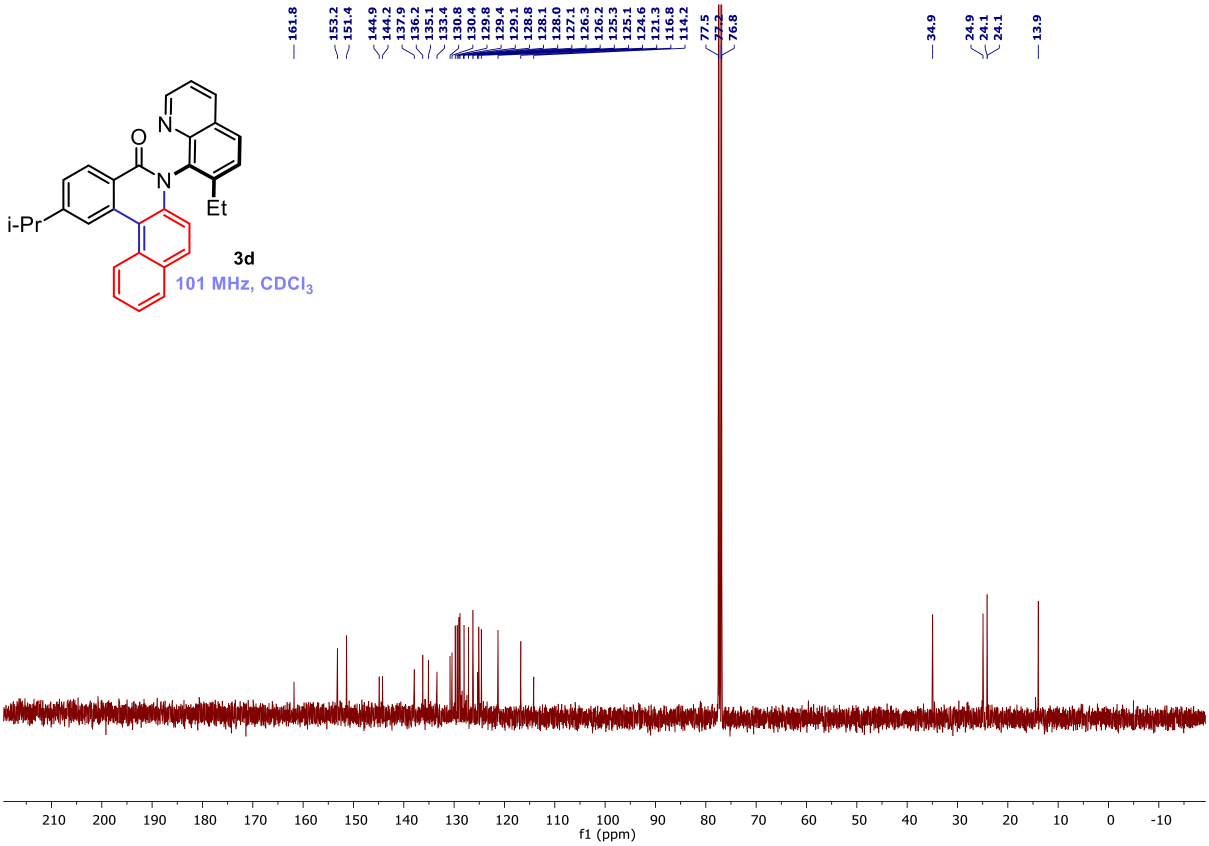
161.8 153.2 151.4 144.9 144.2 137.9 136.2 135.1 133.4 130.8 130.4 129.8 129.4 129.1 128.8 128.1 128.0 127.1 126.3 126.2 125.3 125.1 124.6 121.3 116.8 114.2 77.5 77.2 76.8
34.9 24.9 24.1 24.1 13.9
O
N
N
Et
i-Pr
3d
101 MHz, CDCl3
210 200 190 180 170 160 150 140 130 120 110 100 90 80 70 60 50 40 30 20 10 0 -10
f1 (ppm)

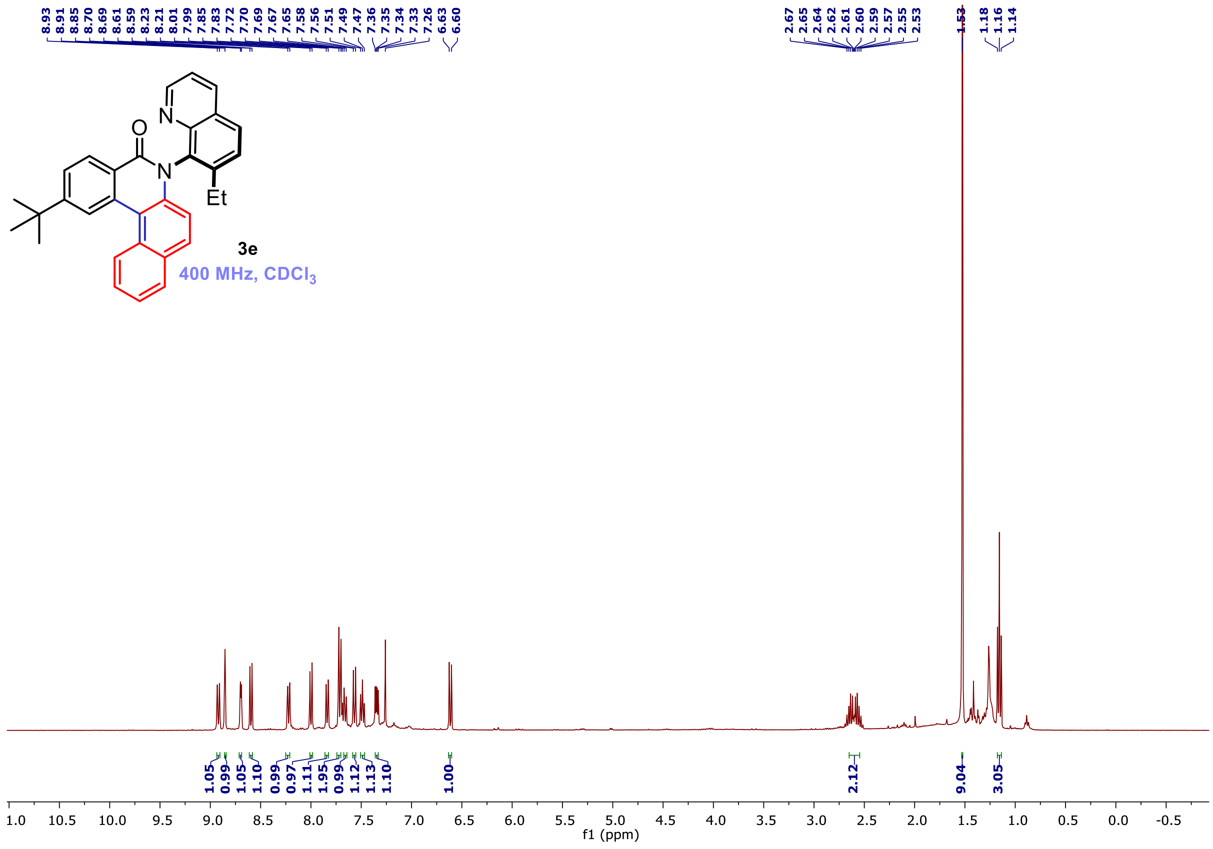
8.93 8.91 8.85 8.70 8.69 8.61 8.59 8.23 8.21 8.01 7.99 7.85 7.83 7.72 7.70 7.69 7.67 7.65 7.58 7.56 7.51 7.49 7.47 7.36 7.35 7.34 7.33 7.26 6.63 6.60
2.67 2.65 2.64 2.62 2.61 2.60 2.59 2.57 2.55 2.53
1.53 1.18 1.16 1.14
O
N
N
Et
3e
400 MHz, CDCl3
1.05 0.99 1.05 1.10 0.99 0.97 1.11 1.95 0.99 1.12 1.13 1.10 1.00 2.12 9.04 3.05
f1 (ppm)

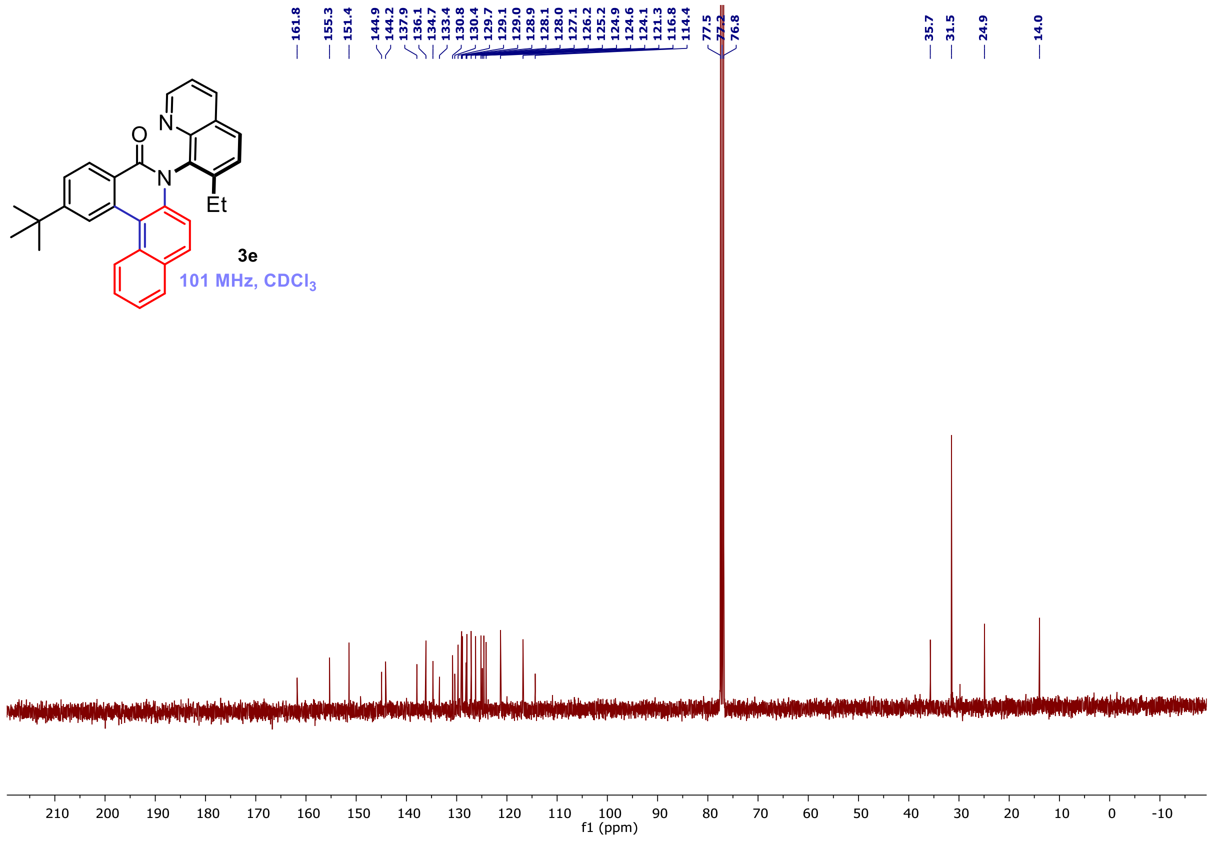
161.8 155.3 151.4 144.9 144.2 137.9 136.1 134.7 133.4 130.8 130.4 129.7 129.1 129.0 128.9 128.1 128.0 127.1 126.2 125.2 124.9 124.6 124.1 121.3 116.8 114.4 77.5 77.2 76.8
35.7 31.5 24.9 14.0
O
N
N
Et
3e
101 MHz, CDCl3
f1 (ppm)

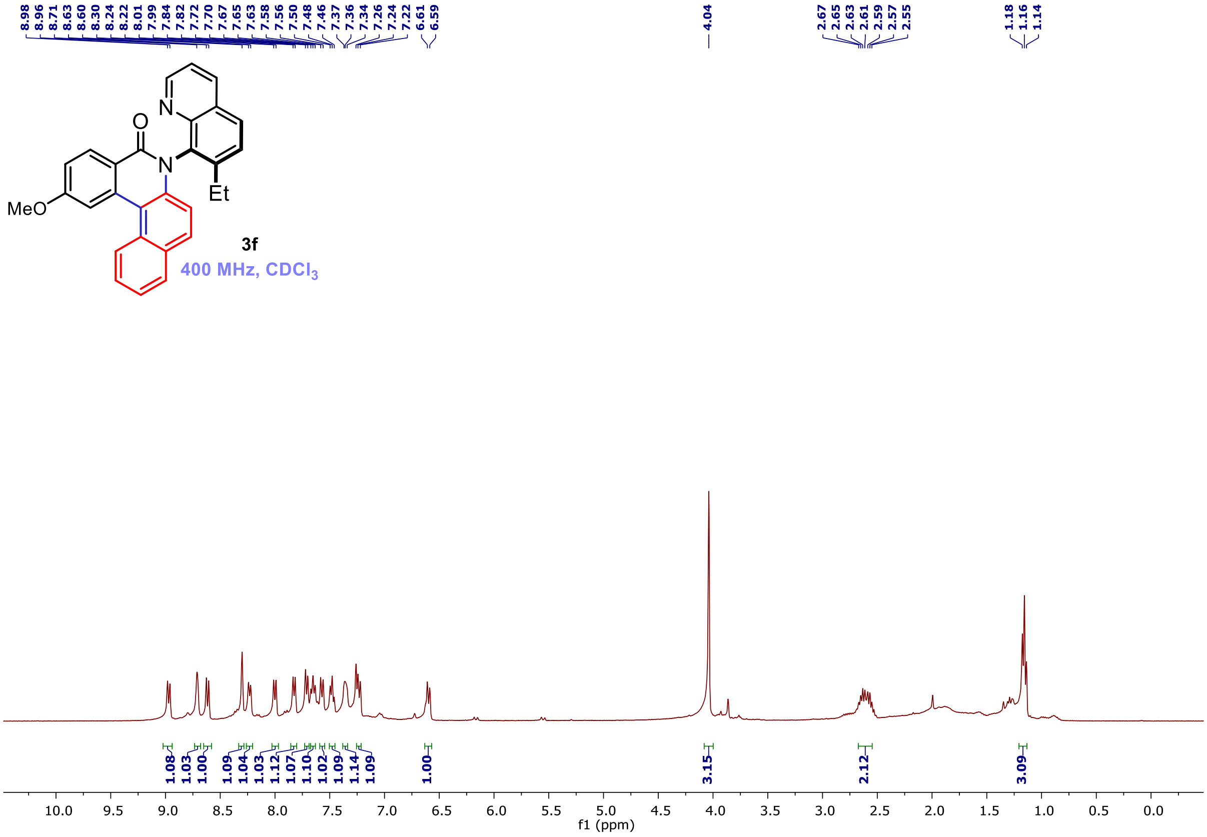

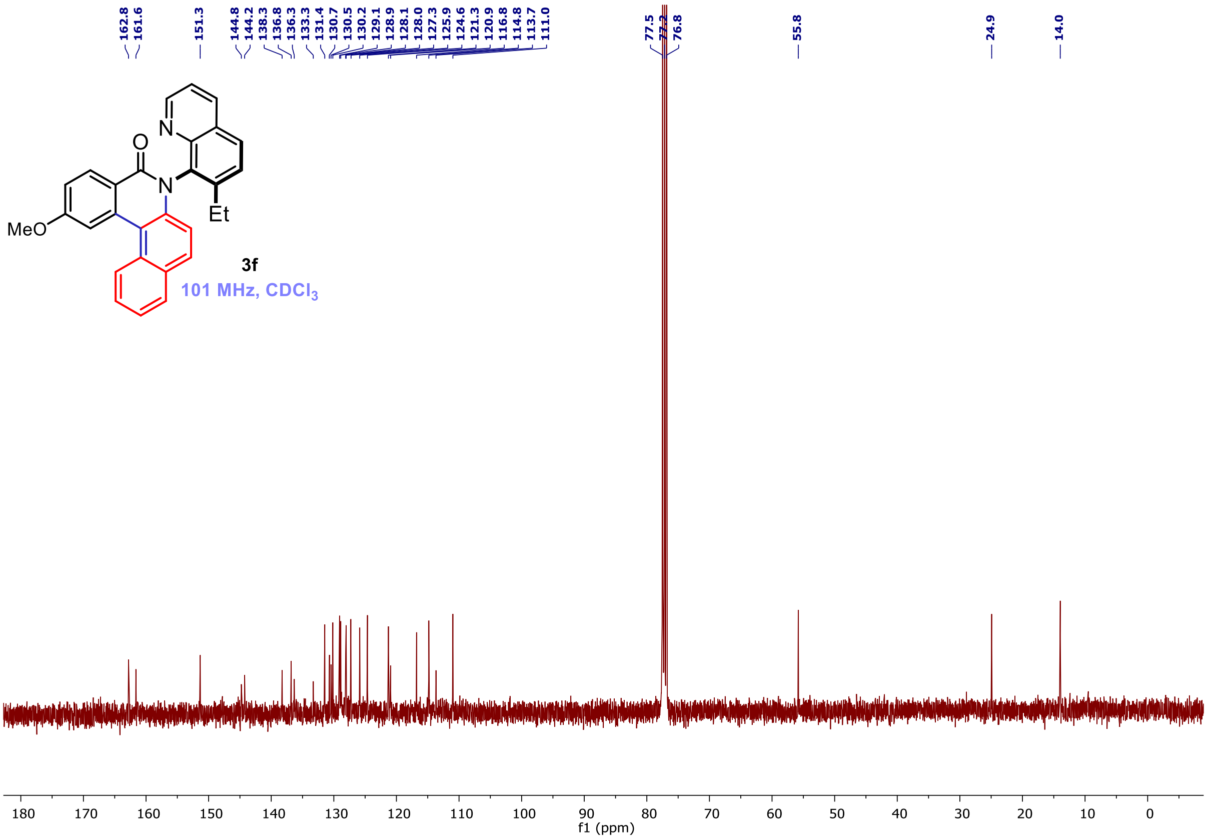

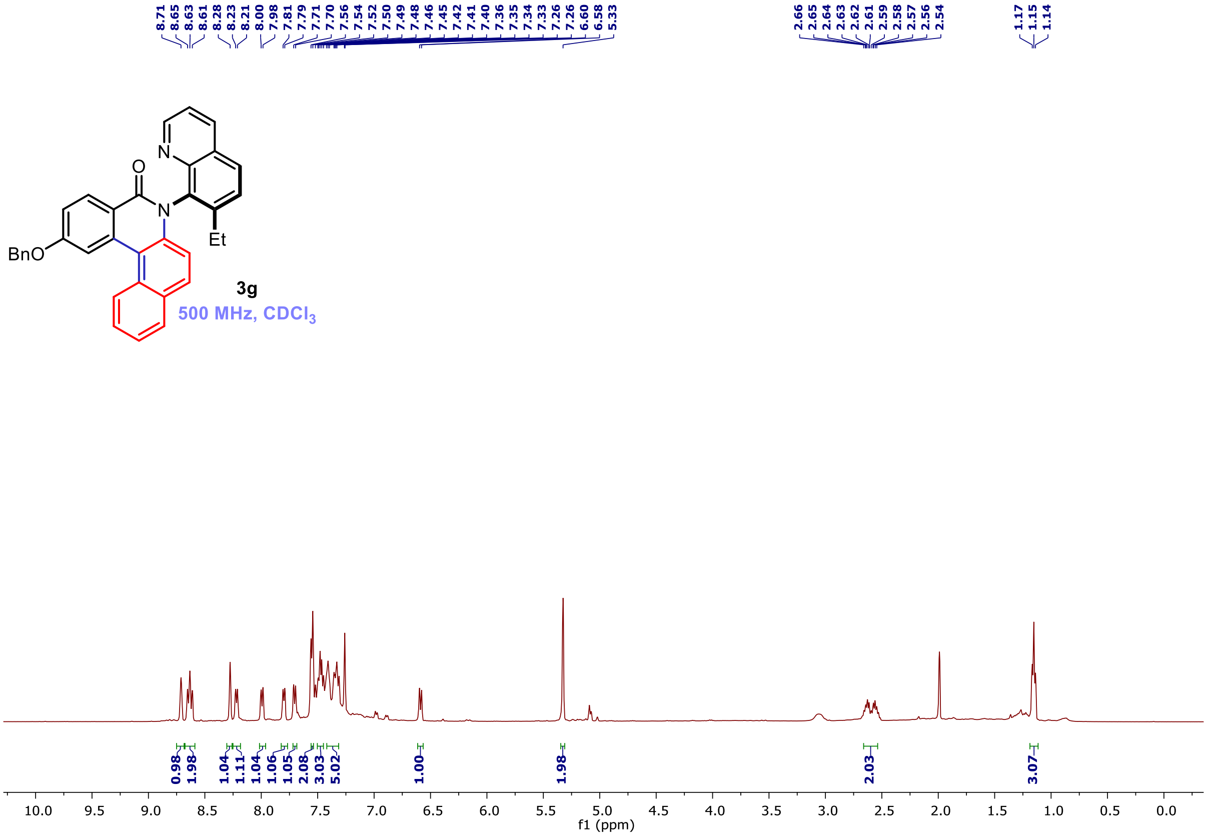

3g
500 MHz, $CDCl_3$

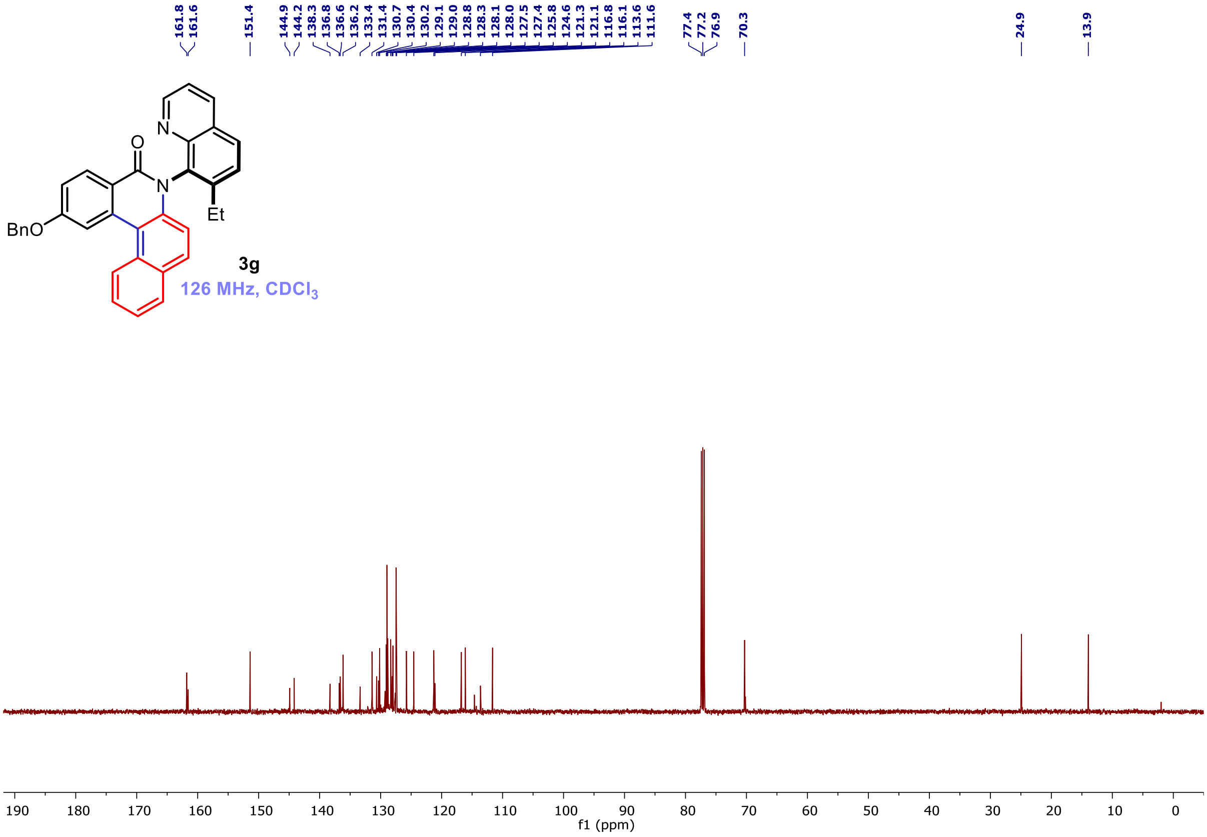

3g
126 MHz, $CDCl_3$

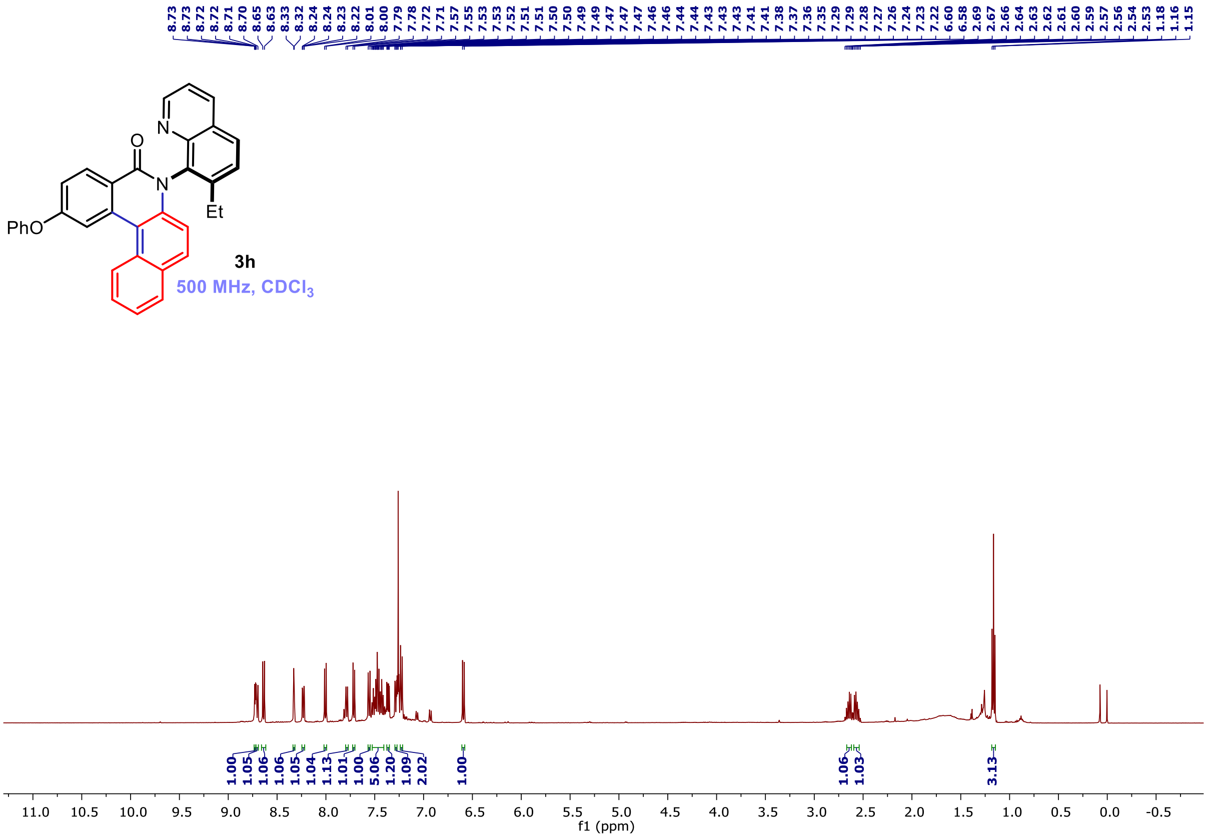
8.73 8.73 8.72 8.72 8.71 8.70 8.65 8.63 8.33 8.32 8.24 8.24 8.23 8.22 8.01 8.00 7.79 7.78 7.72 7.71 7.57 7.55 7.53 7.53 7.52 7.51 7.51 7.50 7.50 7.49 7.49 7.47 7.47 7.47 7.46 7.46 7.44 7.44 7.43 7.43 7.41 7.38 7.37 7.36 7.35 7.29 7.28 7.27 7.26 7.24 7.23 7.23 7.22 6.60 6.58 2.69 2.67 2.66 2.64 2.63 2.62 2.61 2.60 2.59 2.57 2.56 2.54 2.53 1.18 1.16 1.15
PhO
O
N
N
Et
3h
500 MHz, $CDCl_3$
1.00 1.05 1.06 1.06 1.05 1.04 1.13 1.01 1.00 5.06 1.20 1.09 2.02 1.00 1.06 1.03 3.13
11.0 10.5 10.0 9.5 9.0 8.5 8.0 7.5 7.0 6.5 6.0 5.5 5.0 4.5 4.0 3.5 3.0 2.5 2.0 1.5 1.0 0.5 0.0 -0.5
f1 (ppm)

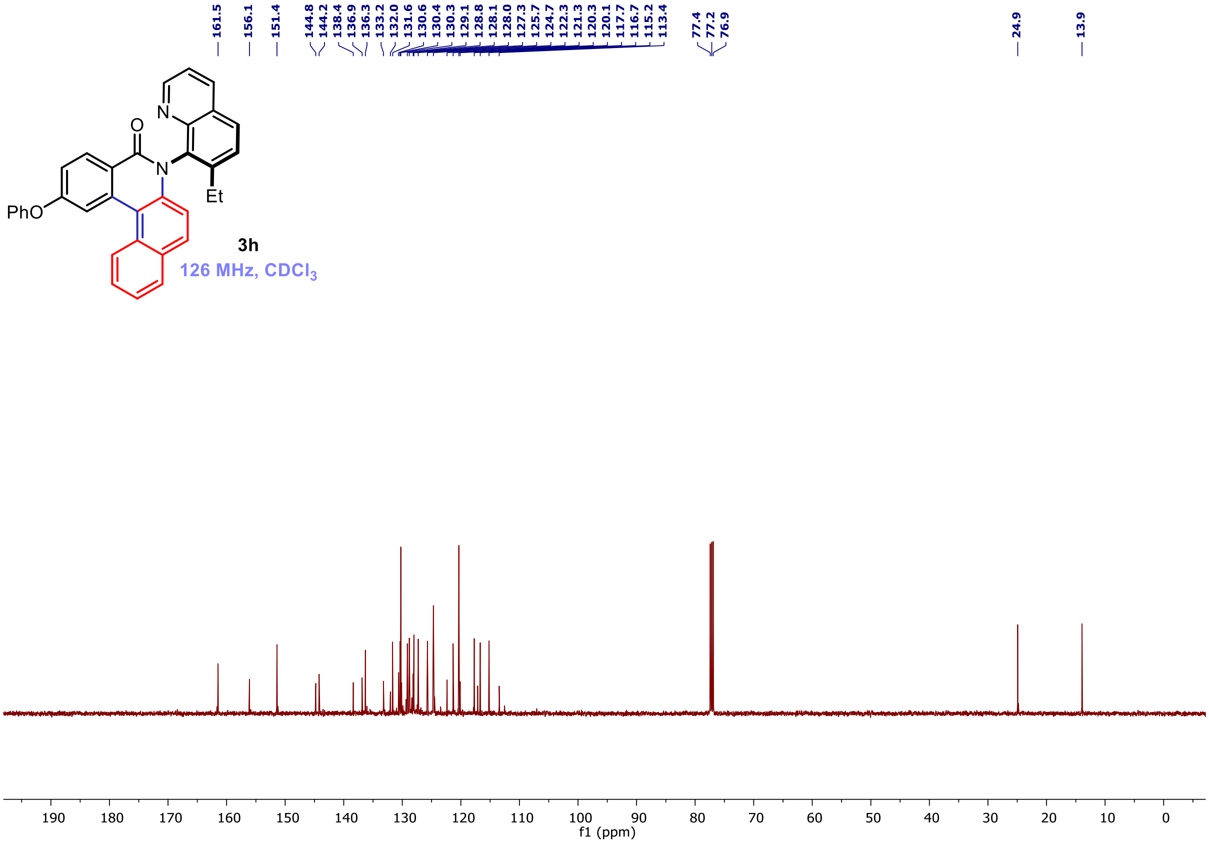
161.5 156.1 151.4 144.8 144.2 138.4 136.9 136.3 133.2 132.0 131.6 130.4 130.3 129.1 128.8 128.1 128.0 127.3 125.7 124.7 122.3 121.3 120.3 120.1 117.7 116.7 115.2 113.4 77.4 77.2 76.9 24.9 13.9
PhO
O
N
N
Et
3h
126 MHz, $CDCl_3$
190 180 170 160 150 140 130 120 110 100 90 80 70 60 50 40 30 20 10 0
f1 (ppm)

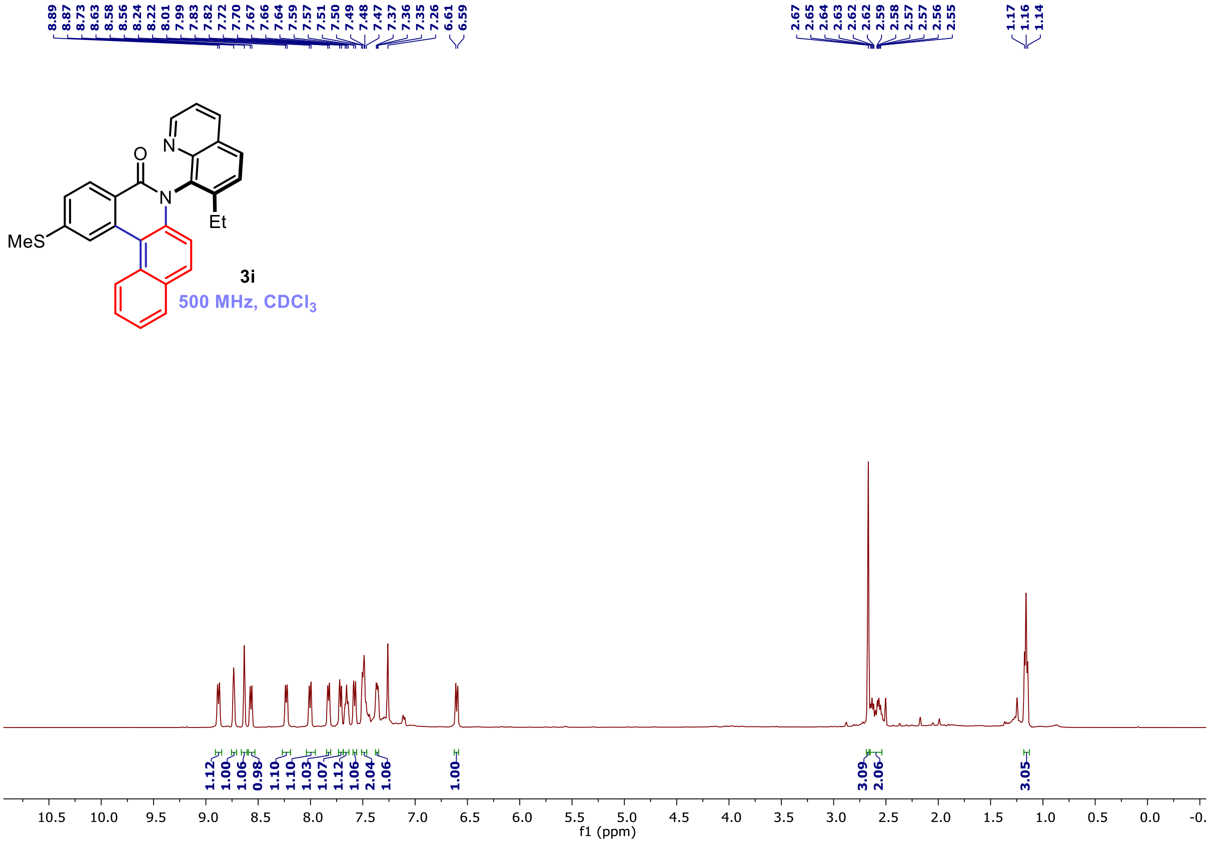
8.89 8.87 8.63 8.58 8.56 8.24 8.22 8.01 7.99 7.83 7.82 7.72 7.70 7.67 7.66 7.64 7.59 7.57 7.51 7.50 7.49 7.48 7.47 7.37 7.36 7.35 7.26 6.61 6.59
2.67 2.65 2.64 2.63 2.62 2.62 2.59 2.58 2.57 2.57 2.56 2.55
1.17 1.16 1.14
O
N
N
Et
MeS
3i
500 MHz, CDCl3
1.12 1.00 1.06 0.98 1.10 1.10 1.03 1.07 1.12 1.06 2.04 1.06
1.00
3.09 2.06
3.05
10.5 10.0 9.5 9.0 8.5 8.0 7.5 7.0 6.5 6.0 5.5 5.0 4.5 4.0 3.5 3.0 2.5 2.0 1.5 1.0 0.5 0.0 -0.
f1 (ppm)

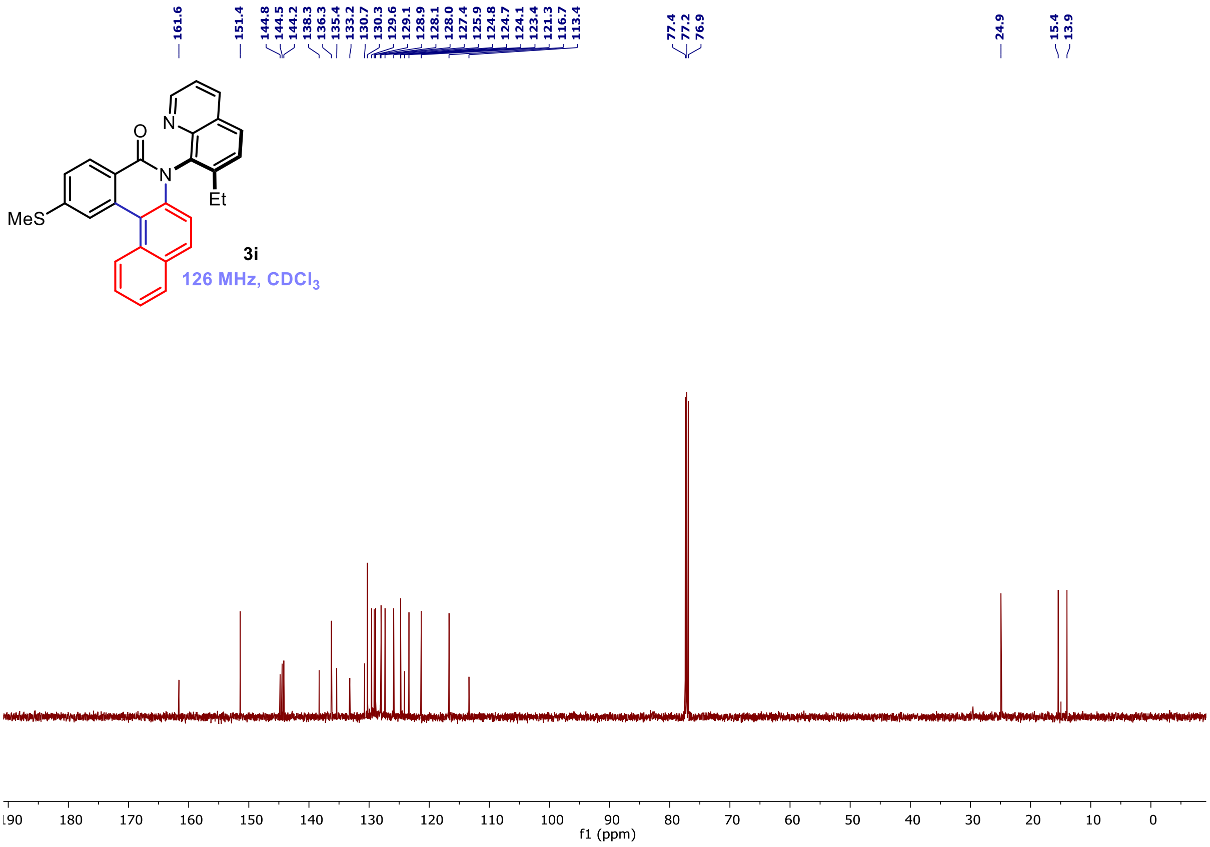
161.6
151.4
144.8 144.5 144.2 138.3 136.3 135.4 133.2 130.7 130.3 129.6 129.1 128.9 128.1 128.0 127.4 125.9 124.8 124.7 124.1 123.4 121.3 116.7 113.4
77.4 77.2 76.9
24.9
15.4 13.9
O
N
N
Et
MeS
3i
126 MHz, CDCl3
190 180 170 160 150 140 130 120 110 100 90 80 70 60 50 40 30 20 10 0
f1 (ppm)

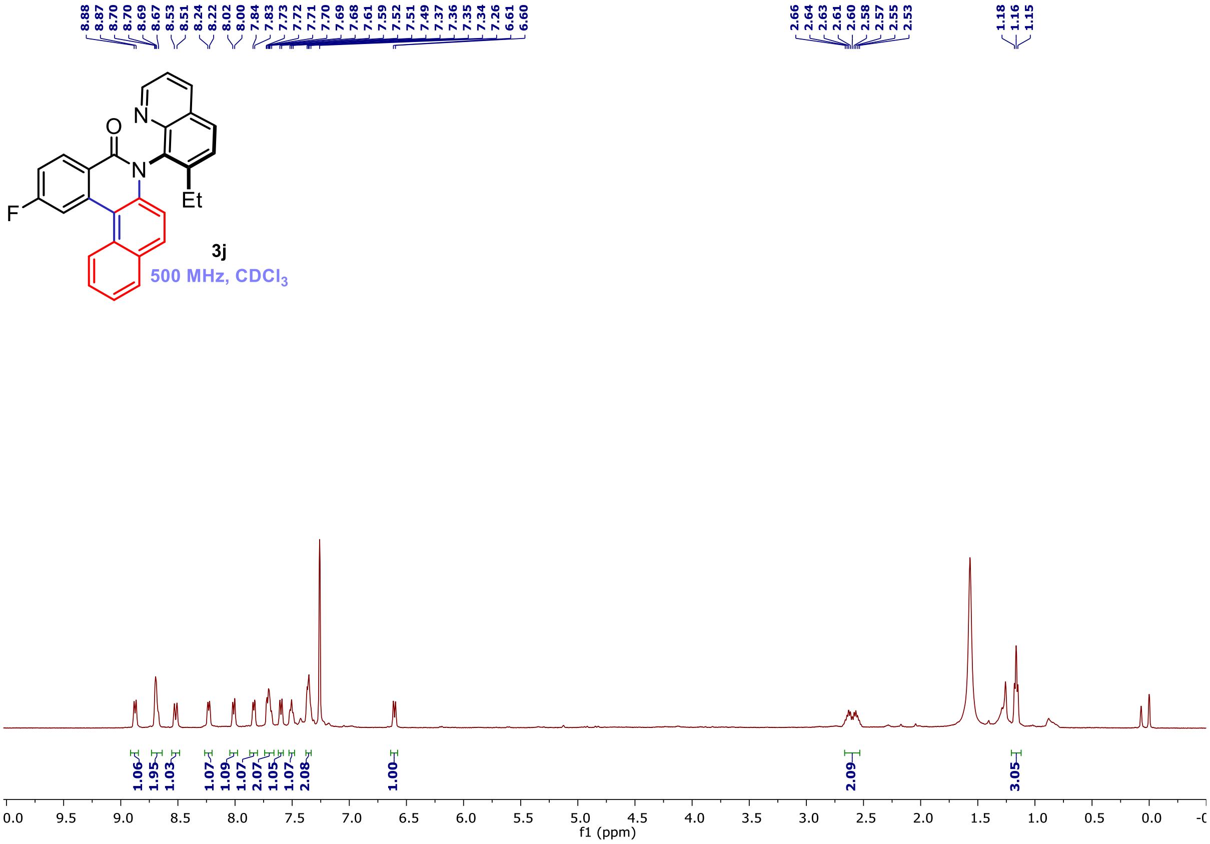
8.88 8.87 8.70 8.70 8.69 8.67 8.53 8.51 8.24 8.22 8.02 8.00 7.84 7.83 7.73 7.72 7.71 7.70 7.69 7.68 7.61 7.59 7.52 7.51 7.49 7.37 7.36 7.35 7.34 7.26 6.61 6.60
2.66 2.64 2.63 2.61 2.60 2.58 2.57 2.55 2.53
1.18 1.16 1.15
O
N
N
F
Et
3j
500 MHz, CDCl3
1.06 1.95 1.03 1.07 1.09 1.07 2.07 1.05 1.07 2.08 1.00 2.09 3.05
f1 (ppm)

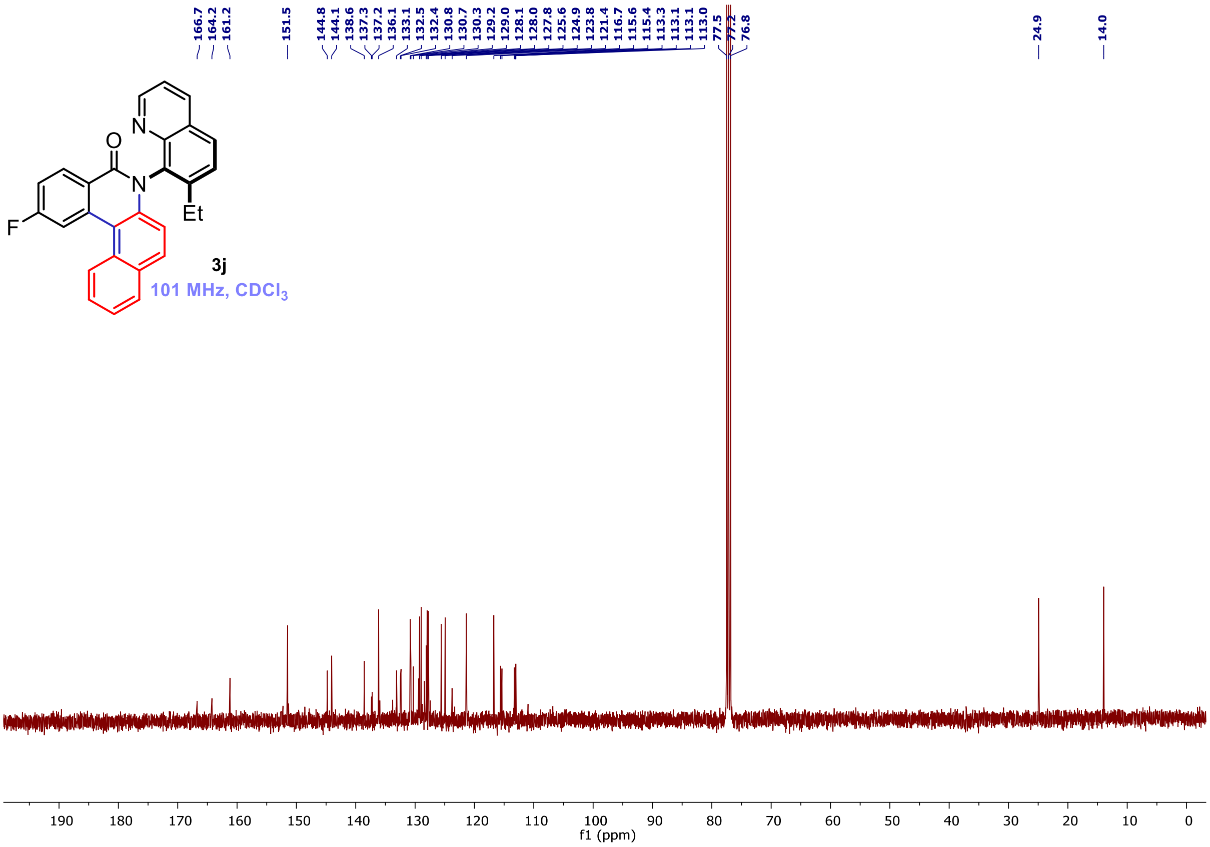
166.7 164.2 161.2 151.5 144.8 144.1 138.6 137.3 137.2 136.1 133.1 132.5 132.4 130.8 130.7 130.3 129.2 129.0 128.1 128.0 127.8 125.6 124.9 123.8 121.4 116.7 115.6 115.4 113.3 113.1 113.1 113.0 77.5 77.2 76.8 24.9 14.0
O
N
N
F
Et
3j
101 MHz, CDCl3
f1 (ppm)

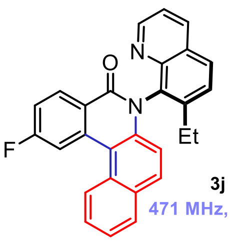

3j

471 MHz, $CDCl_3$

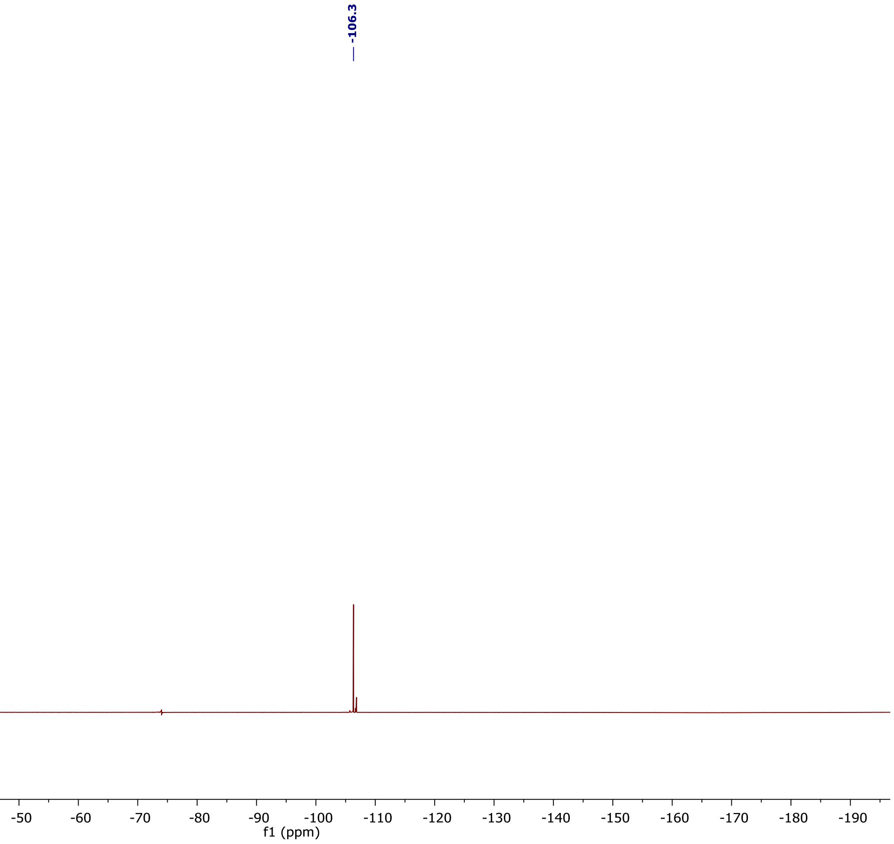

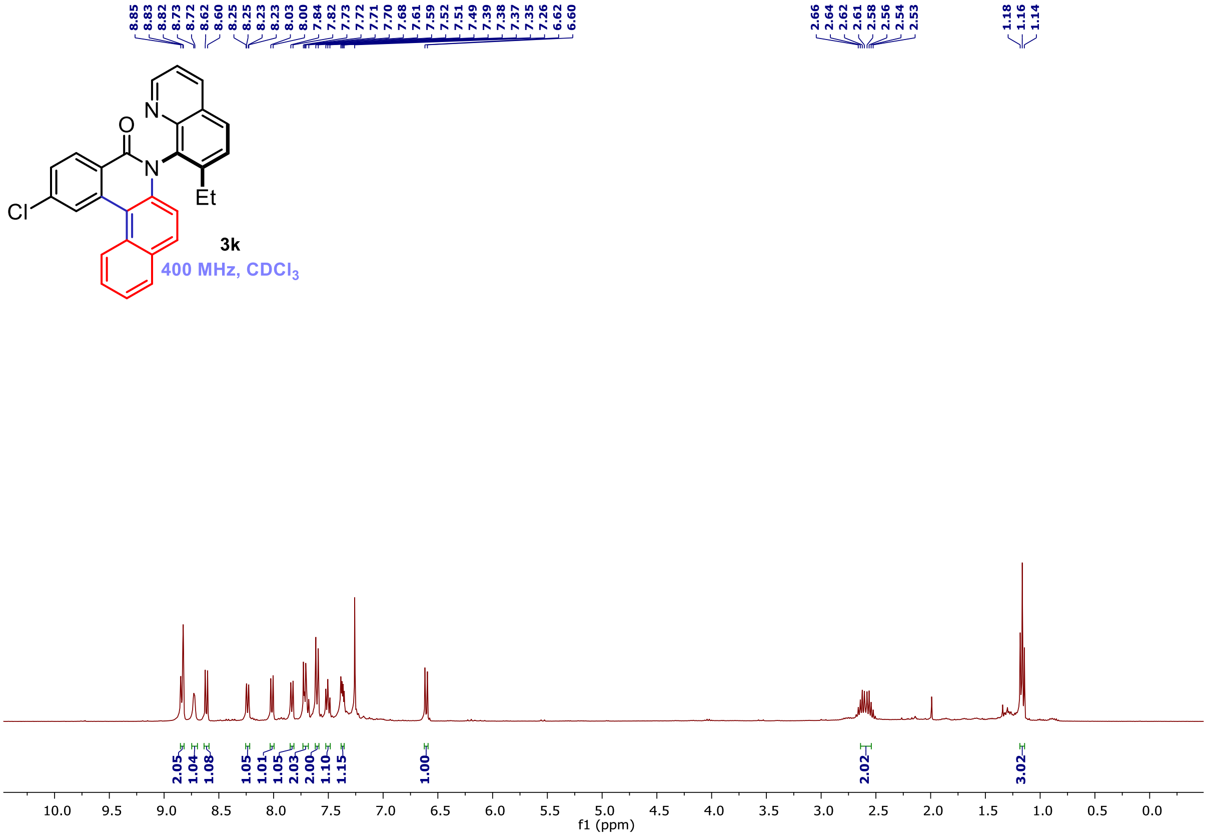
8.85 8.83 8.82 8.73 8.72 8.62 8.60 8.25 8.25 8.23 8.23 8.03 8.00 7.84 7.82 7.73 7.72 7.71 7.70 7.68 7.61 7.59 7.52 7.51 7.49 7.39 7.38 7.37 7.35 7.26 6.62 6.60
2.66 2.64 2.62 2.61 2.58 2.56 2.54 2.53
1.18 1.16 1.14
O
N
N
Cl
Et
3k
400 MHz, CDCl3
2.05 1.04 1.08 1.05 1.01 1.05 2.03 2.00 1.10 1.15 1.00 2.02 3.02
10.0 9.5 9.0 8.5 8.0 7.5 7.0 6.5 6.0 5.5 5.0 4.5 4.0 3.5 3.0 2.5 2.0 1.5 1.0 0.5 0.0
f1 (ppm)

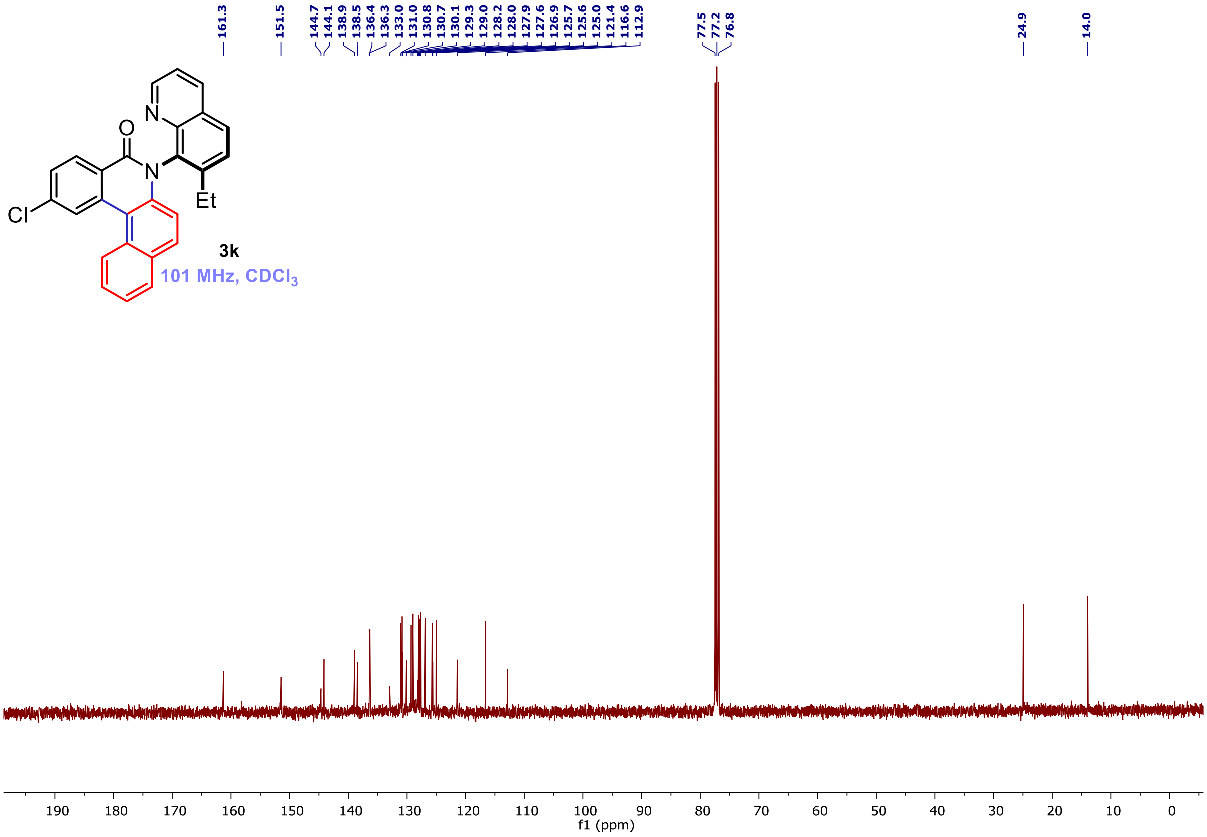
161.3
151.5
144.7 144.1 138.9 138.5 136.4 136.3 133.0 131.0 130.8 130.7 130.1 129.3 129.0 128.2 128.0 127.9 127.6 126.9 125.7 125.6 125.0 121.4 116.6 112.9
77.5 77.2 76.8
24.9
14.0
O
N
N
Cl
Et
3k
101 MHz, CDCl3
190 180 170 160 150 140 130 120 110 100 90 80 70 60 50 40 30 20 10 0
f1 (ppm)

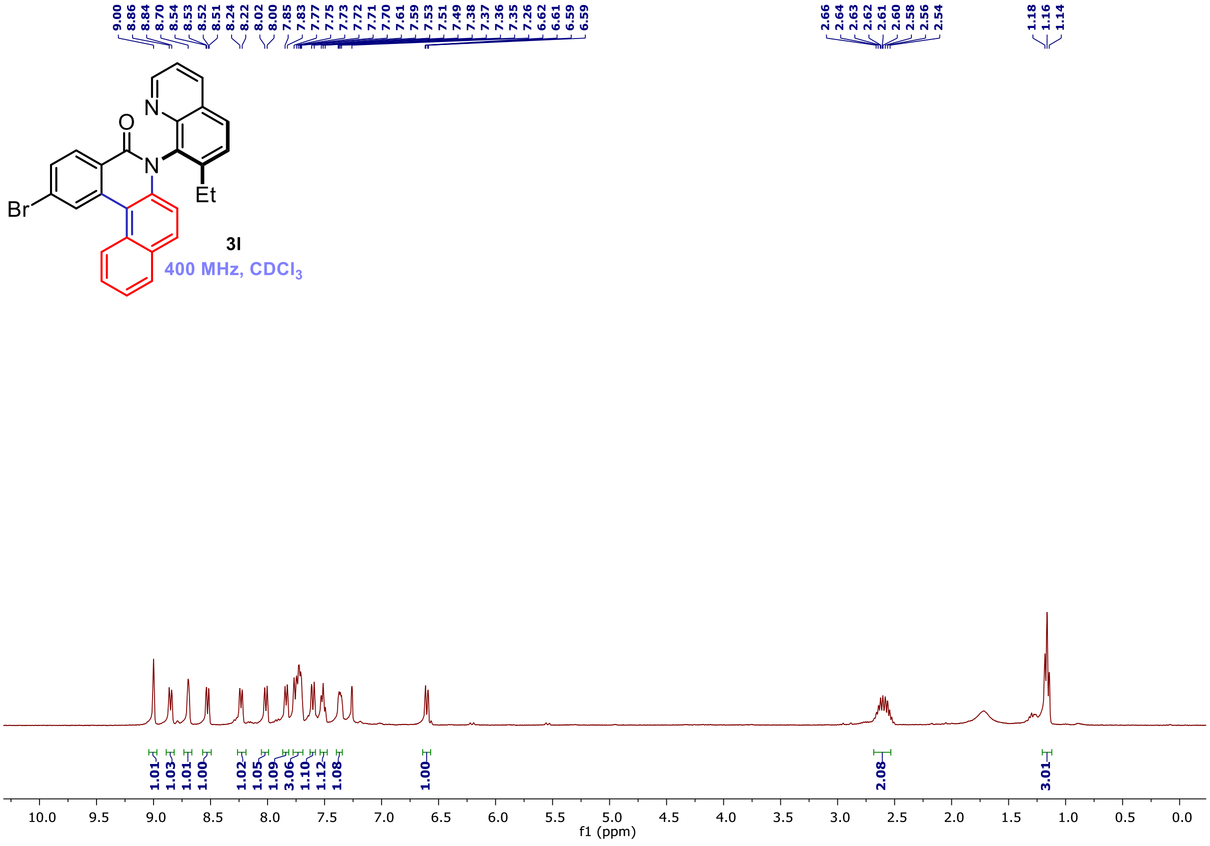
9.00 8.86 8.84 8.70 8.54 8.53 8.52 8.51 8.24 8.22 8.02 8.00 7.85 7.83 7.77 7.75 7.73 7.72 7.71 7.70 7.61 7.59 7.53 7.51 7.49 7.38 7.37 7.36 7.35 7.26 6.62 6.61 6.59 6.59
2.66 2.64 2.63 2.62 2.61 2.60 2.58 2.56 2.54
1.18 1.16 1.14
O
N
N
Br
Et
3l
400 MHz, CDCl3
1.01 1.03 1.01 1.00 1.02 1.05 1.09 3.06 1.10 1.12 1.08 1.00 2.08 3.01
10.0 9.5 9.0 8.5 8.0 7.5 7.0 6.5 6.0 5.5 5.0 4.5 4.0 3.5 3.0 2.5 2.0 1.5 1.0 0.5 0.0
f1 (ppm)

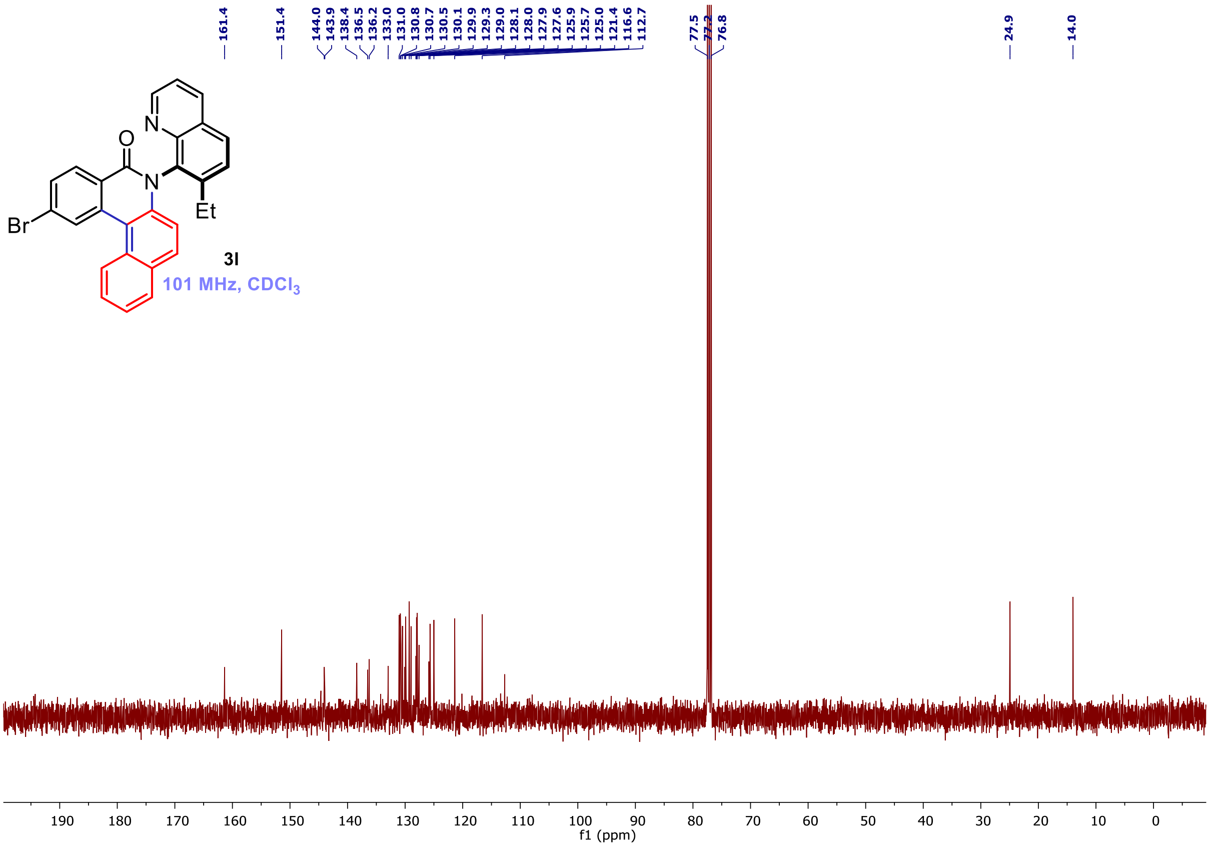
161.4
151.4
144.0 143.9 138.4 136.5 136.2 133.0 131.0 130.8 130.7 130.5 130.1 129.9 129.3 129.0 128.1 128.0 127.9 127.6 125.9 125.7 125.0 121.4 116.6 112.7
77.5 77.2 76.8
24.9
14.0
O
N
N
Br
Et
3l
101 MHz, CDCl3
190 180 170 160 150 140 130 120 110 100 90 80 70 60 50 40 30 20 10 0
f1 (ppm)

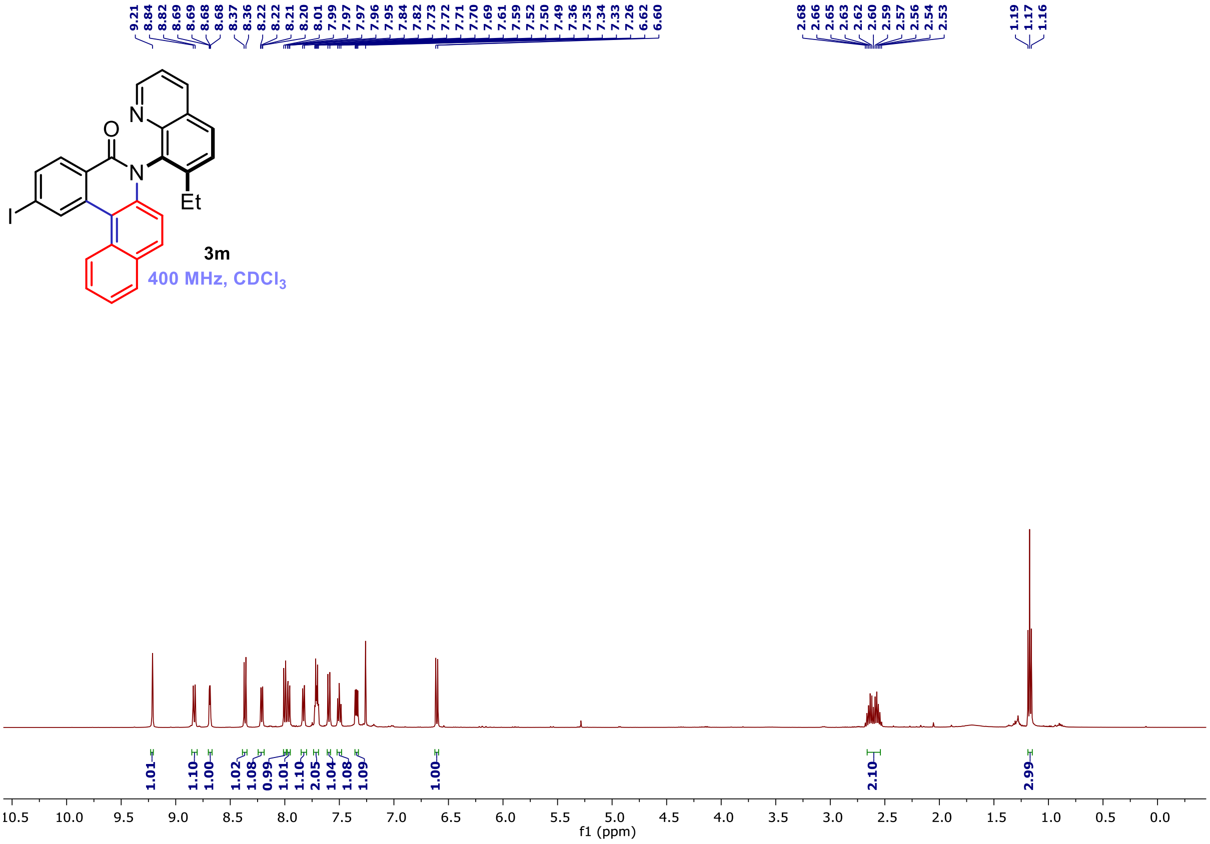
9.21 8.84 8.82 8.69 8.69 8.68 8.68 8.37 8.36 8.22 8.22 8.21 8.20 8.01 7.99 7.97 7.97 7.96 7.95 7.84 7.82 7.73 7.72 7.71 7.70 7.69 7.61 7.59 7.52 7.50 7.49 7.36 7.35 7.34 7.33 7.26 6.62 6.60
2.68 2.66 2.65 2.63 2.62 2.60 2.59 2.57 2.56 2.54 2.53
1.19 1.17 1.16
O
N
N
I
Et
3m
400 MHz, CDCl3
1.01 1.10 1.00 1.02 1.08 0.99 1.01 1.10 2.05 1.04 1.08 1.09 1.00 2.10 2.99
10.5 10.0 9.5 9.0 8.5 8.0 7.5 7.0 6.5 6.0 5.5 5.0 4.5 4.0 3.5 3.0 2.5 2.0 1.5 1.0 0.5 0.0
f1 (ppm)

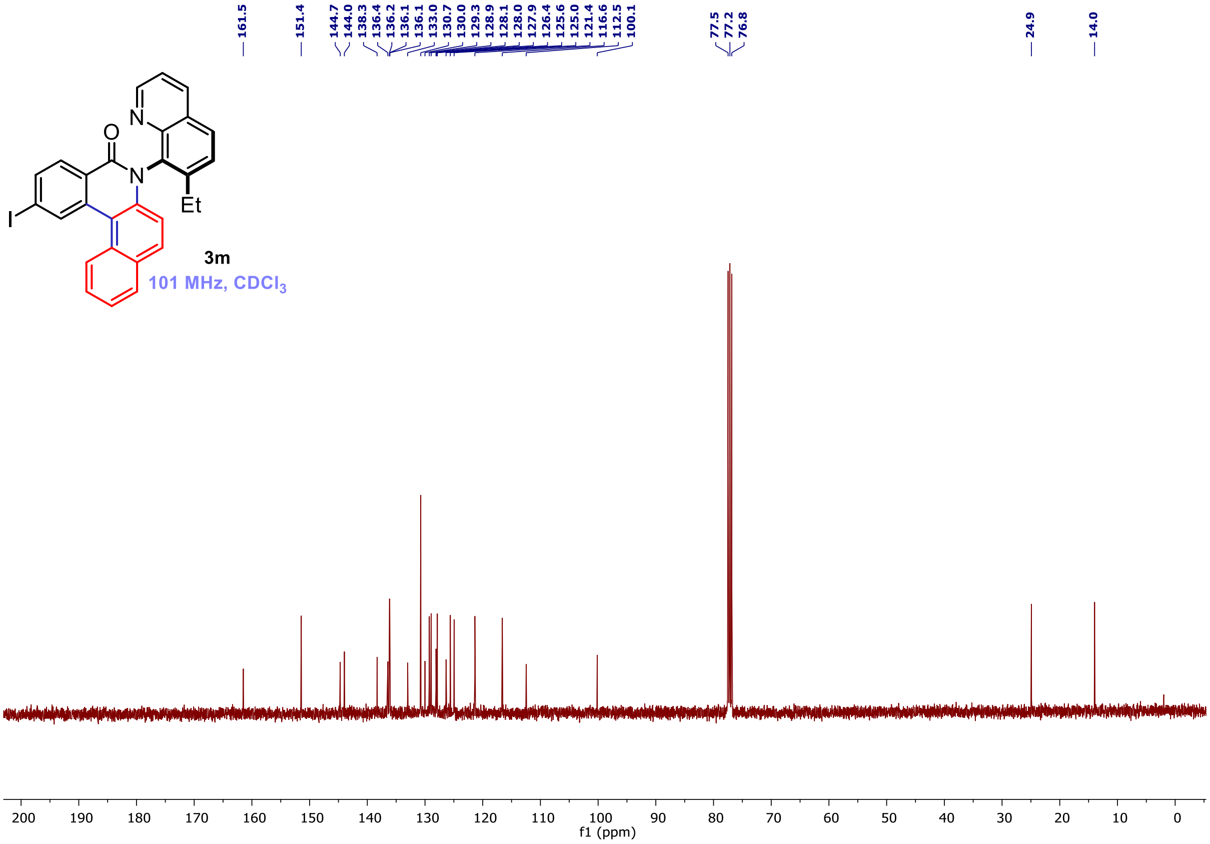
161.5
151.4
144.7 144.0 138.3 136.4 136.2 136.1 136.1 133.0 130.7 130.0 129.3 128.9 128.1 128.0 127.9 126.4 125.6 125.0 121.4 116.6 112.5 100.1
77.5 77.2 76.8
24.9
14.0
O
N
N
I
Et
3m
101 MHz, CDCl3
200 190 180 170 160 150 140 130 120 110 100 90 80 70 60 50 40 30 20 10 0
f1 (ppm)

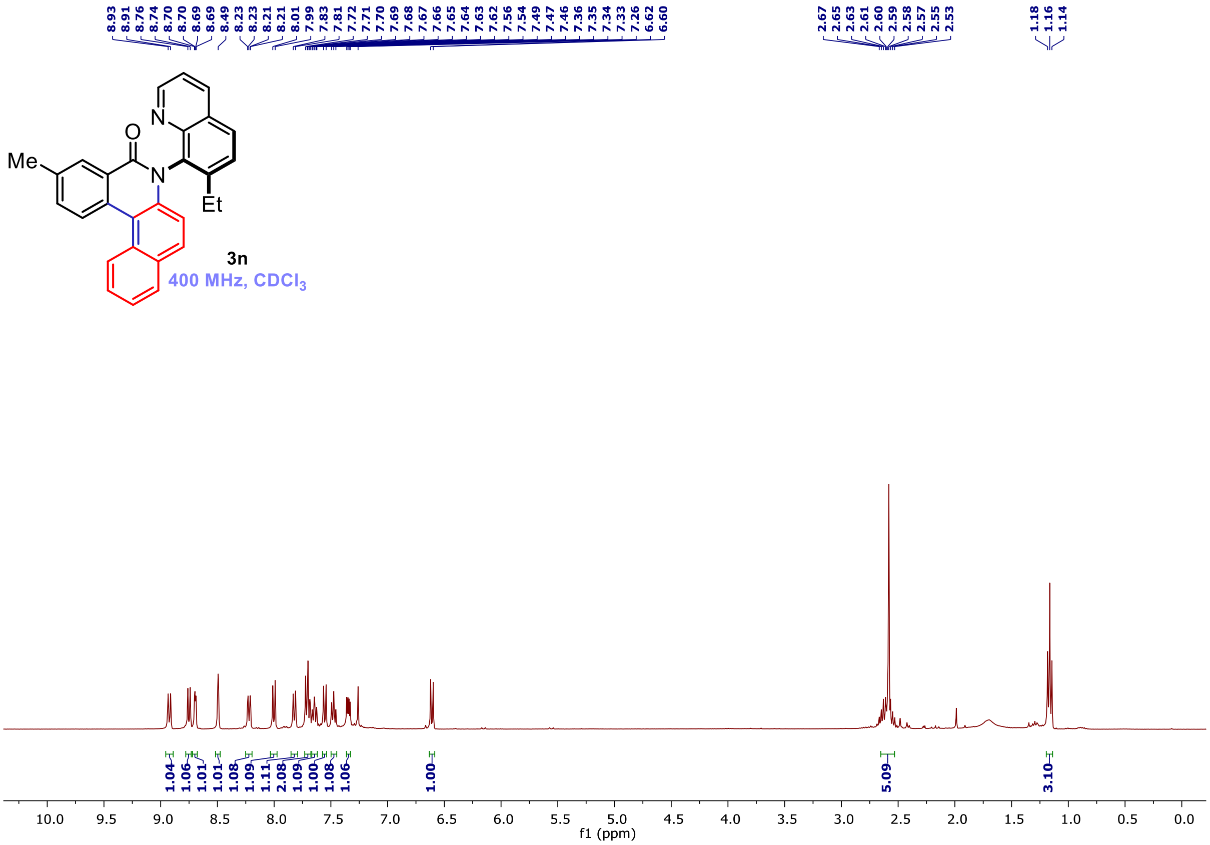
8.93
8.91
8.76
8.74
8.70
8.70
8.69
8.69
8.49
8.23
8.23
8.21
8.21
8.01
7.99
7.83
7.81
7.72
7.71
7.70
7.69
7.68
7.67
7.66
7.65
7.64
7.63
7.62
7.56
7.54
7.49
7.47
7.46
7.36
7.35
7.34
7.33
7.26
6.62
6.60
2.67
2.65
2.63
2.61
2.60
2.59
2.58
2.57
2.55
2.53
1.18
1.16
1.14
Me
O
N
N
Et
3n
400 MHz, CDCl3
1.04
1.06
1.01
1.01
1.08
1.09
1.11
2.08
1.09
1.00
1.08
1.06
1.00
5.09
3.10
f1 (ppm)

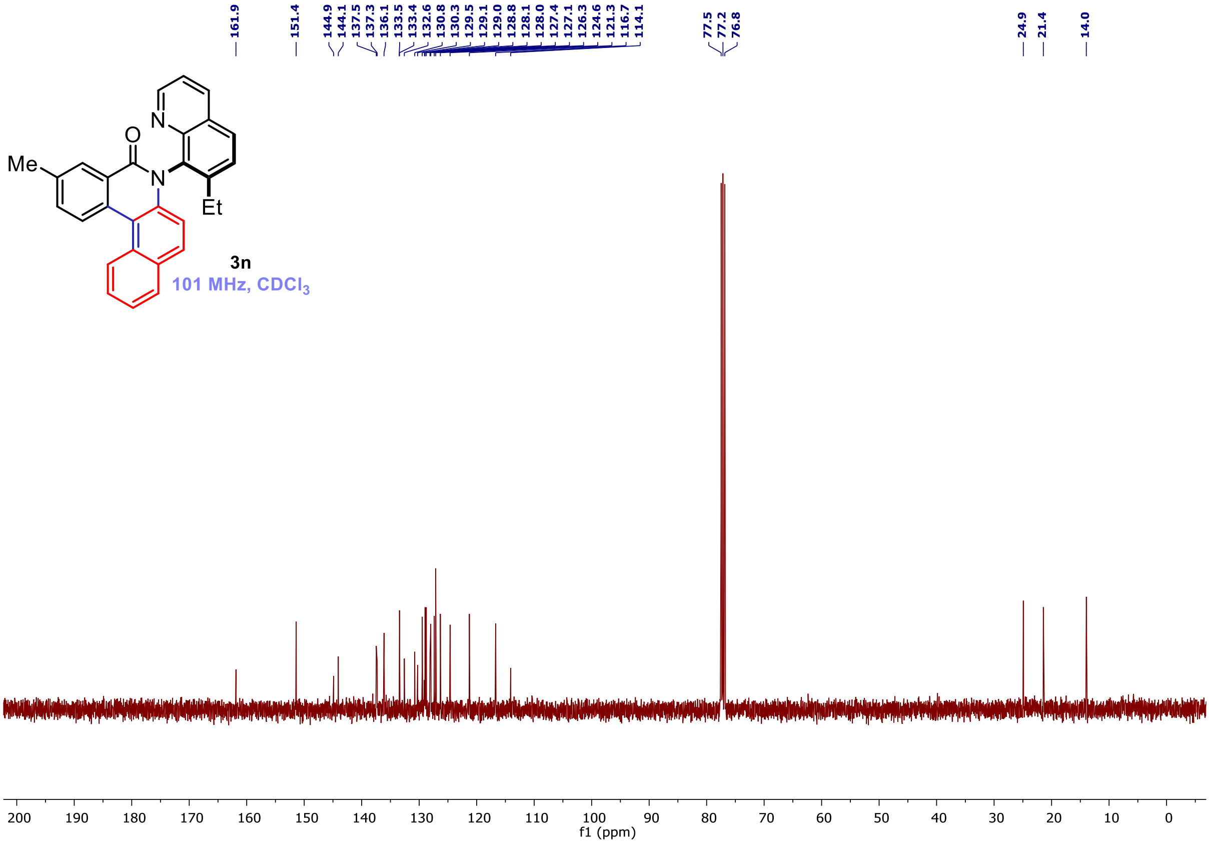
161.9
151.4
144.9
144.1
137.5
137.3
136.1
133.5
133.4
132.6
130.8
130.3
129.5
129.1
129.0
128.8
128.1
128.0
127.4
127.1
126.3
124.6
121.3
116.7
114.1
77.5
77.2
76.8
24.9
21.4
14.0
Me
O
N
N
Et
3n
101 MHz, CDCl3
f1 (ppm)

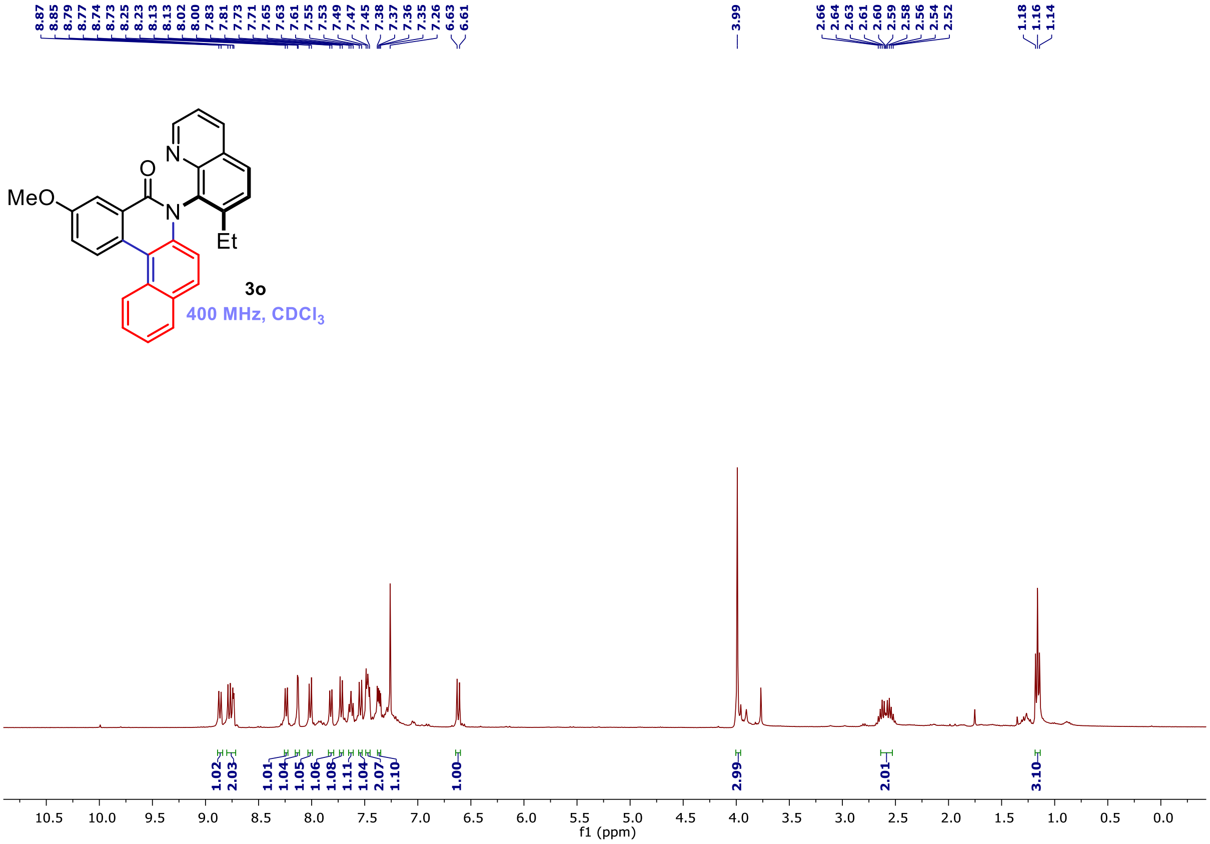
8.87 8.85 8.79 8.77 8.74 8.73 8.25 8.23 8.13 8.13 8.02 8.00 7.83 7.81 7.73 7.71 7.65 7.63 7.61 7.55 7.53 7.49 7.47 7.45 7.38 7.37 7.36 7.35 7.26 6.63 6.61
3.99
2.66 2.64 2.63 2.61 2.60 2.59 2.58 2.56 2.54 2.52
1.18 1.16 1.14
MeO
O
N
N
Et
3o
400 MHz, CDCl3
1.02 2.03 1.01 1.04 1.05 1.06 1.08 1.11 1.04 2.07 1.10 1.00 2.99 2.01 3.10
10.5 10.0 9.5 9.0 8.5 8.0 7.5 7.0 6.5 6.0 5.5 5.0 4.5 4.0 3.5 3.0 2.5 2.0 1.5 1.0 0.5 0.0
f1 (ppm)

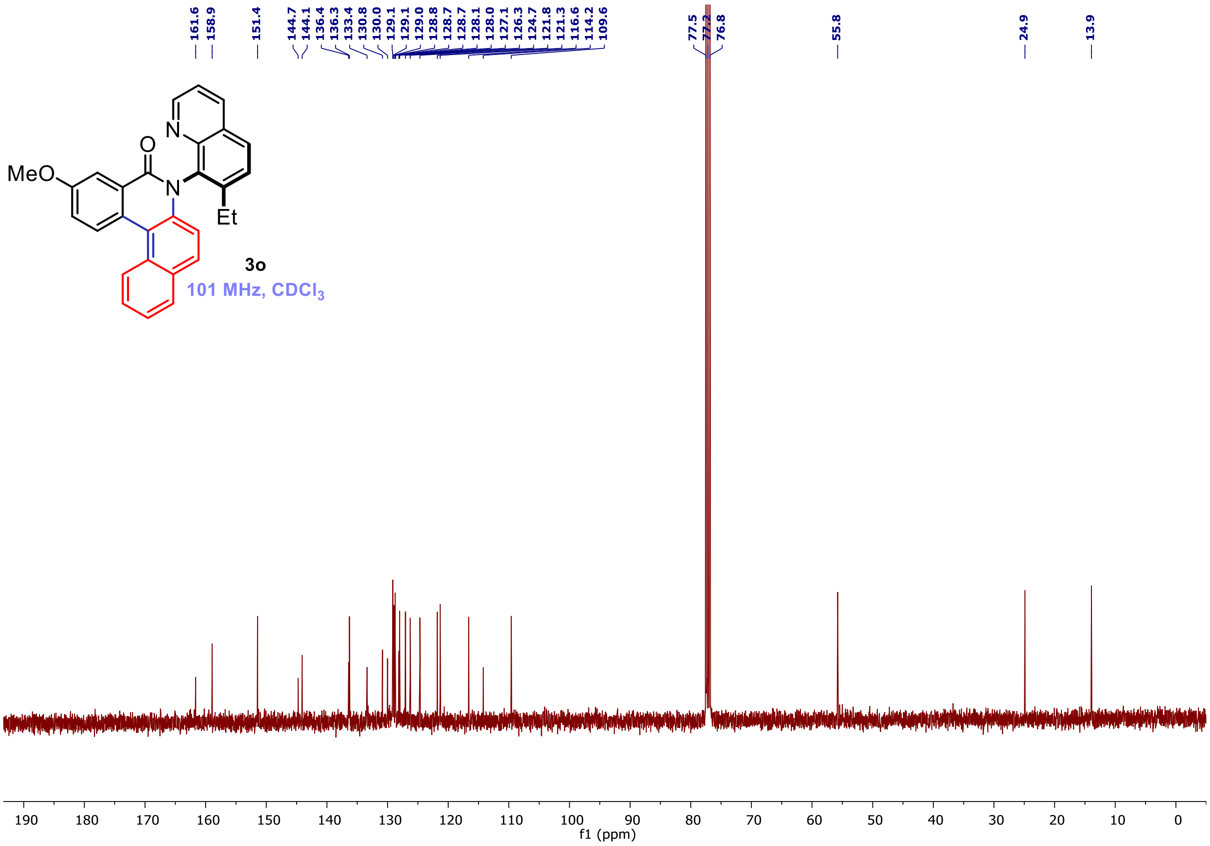
161.6 158.9 151.4 144.7 144.1 136.4 136.3 133.4 130.8 130.0 129.1 129.1 129.0 128.8 128.7 128.7 128.1 128.0 127.1 126.3 124.7 121.8 121.3 116.6 114.2 109.6
77.5 77.2 76.8
55.8
24.9
13.9
MeO
O
N
N
Et
3o
101 MHz, CDCl3
190 180 170 160 150 140 130 120 110 100 90 80 70 60 50 40 30 20 10 0
f1 (ppm)

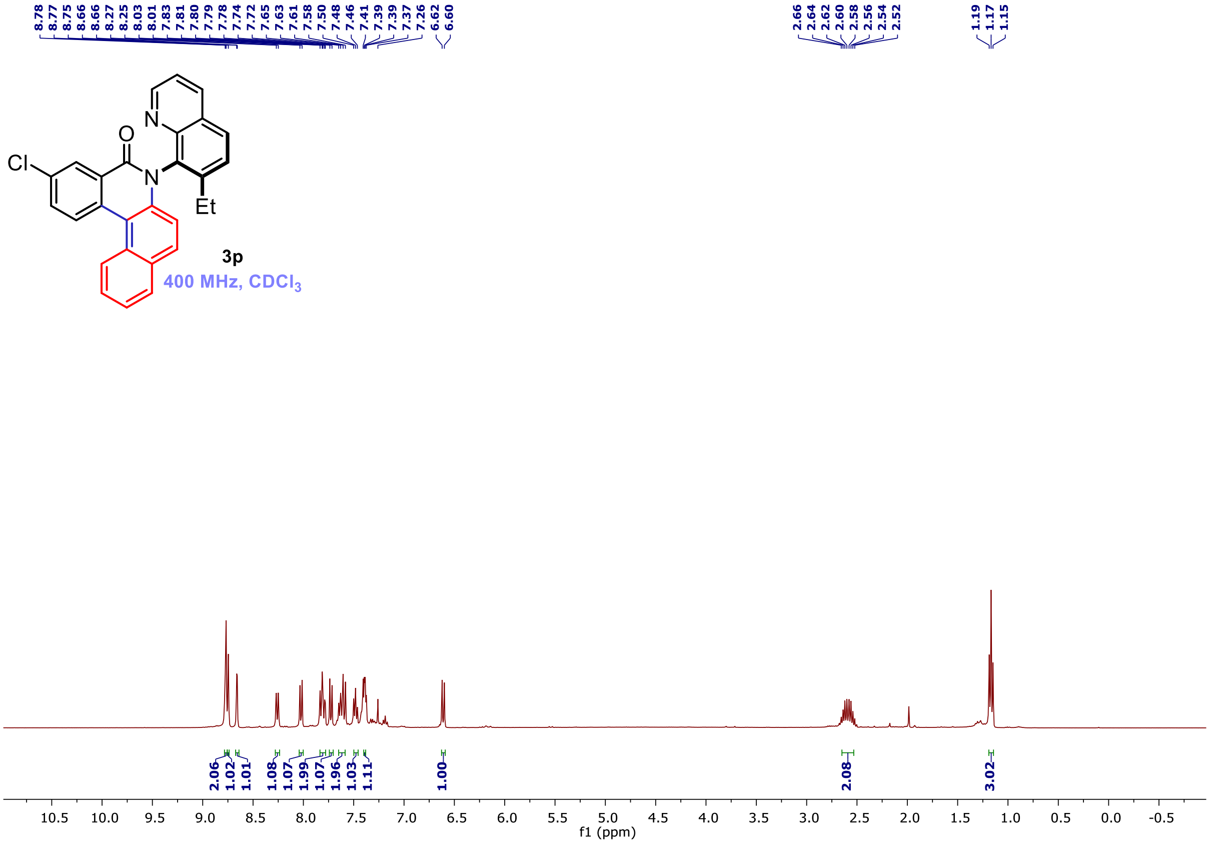
8.78 8.77 8.75 8.66 8.27 8.25 8.03 8.01 7.83 7.81 7.80 7.79 7.78 7.74 7.72 7.65 7.63 7.61 7.58 7.50 7.48 7.46 7.41 7.39 7.39 7.37 7.26 6.62 6.60
2.66 2.64 2.62 2.60 2.58 2.56 2.54 2.52
1.19 1.17 1.15
Cl
O
N
N
Et
3p
400 MHz, CDCl3
2.06 1.02 1.01 1.08 1.07 1.99 1.07 1.96 1.03 1.11 1.00 2.08 3.02
10.5 10.0 9.5 9.0 8.5 8.0 7.5 7.0 6.5 6.0 5.5 5.0 4.5 4.0 3.5 3.0 2.5 2.0 1.5 1.0 0.5 0.0 -0.5
f1 (ppm)

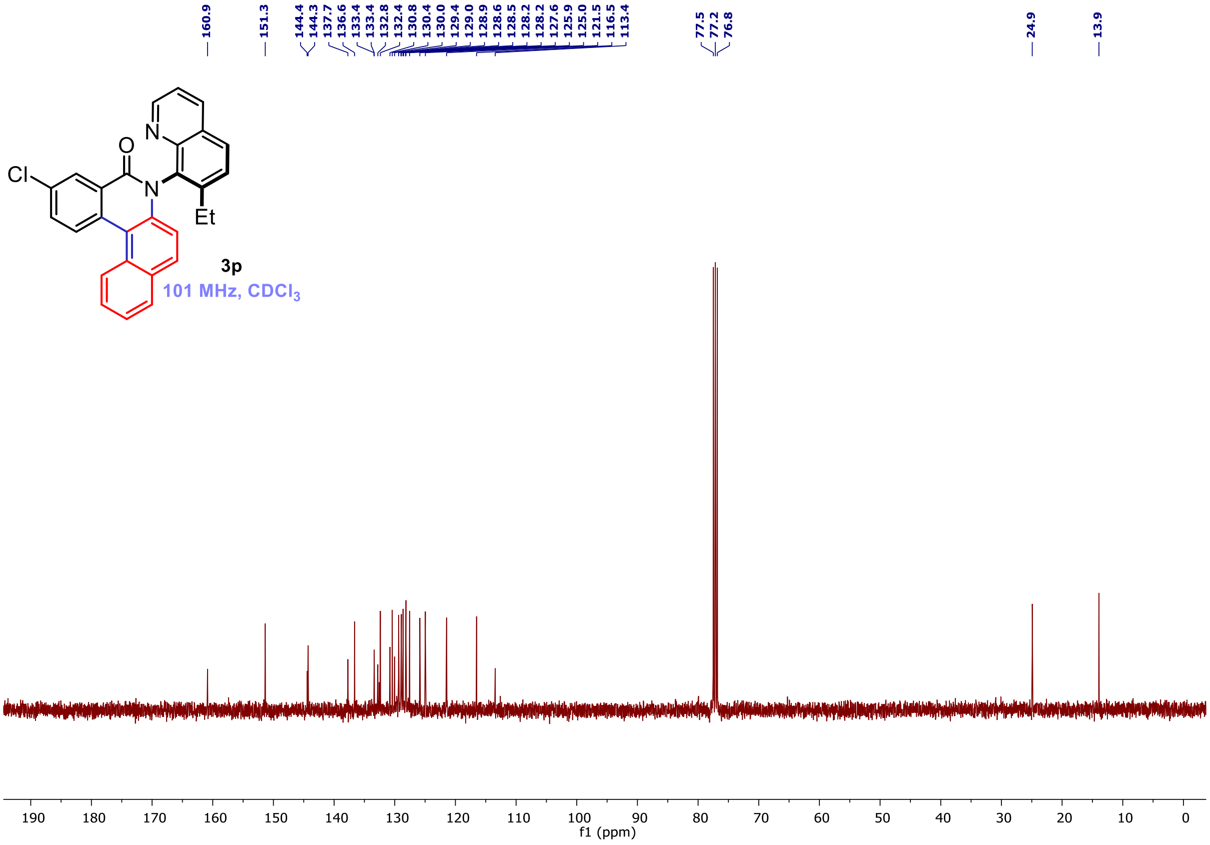
160.9
151.3
144.4 144.3 137.7 136.6 133.4 133.4 132.8 132.4 130.8 130.4 130.0 129.4 129.0 128.9 128.6 128.5 128.2 127.6 125.9 125.0 121.5 116.5 113.4
77.5 77.2 76.8
24.9
13.9
Cl
O
N
N
Et
3p
101 MHz, CDCl3
190 180 170 160 150 140 130 120 110 100 90 80 70 60 50 40 30 20 10 0
f1 (ppm)

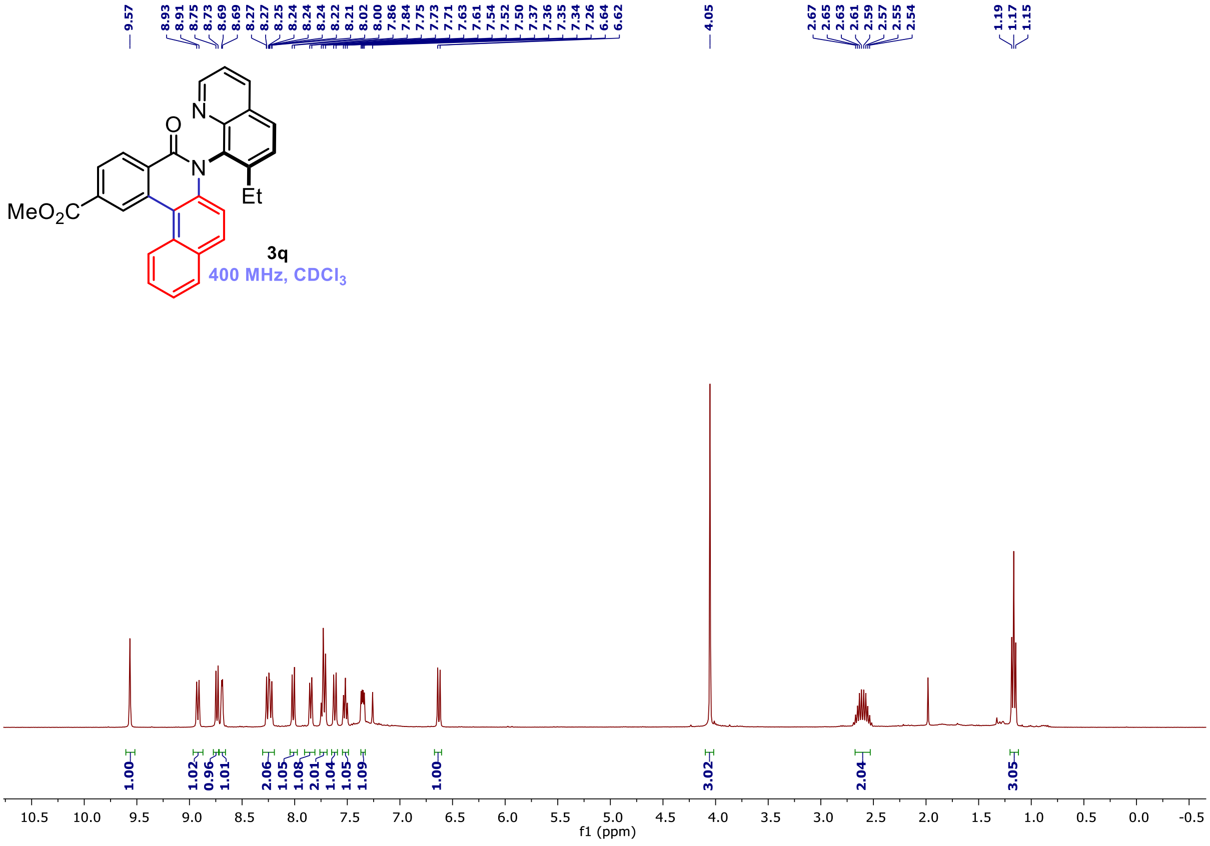

9.57
8.93
8.91
8.75
8.73
8.69
8.69
8.27
8.25
8.24
8.24
8.24
8.22
8.21
8.02
8.00
7.86
7.84
7.75
7.73
7.71
7.63
7.61
7.54
7.52
7.50
7.37
7.36
7.35
7.34
7.26
6.64
6.62
4.05
2.67
2.65
2.63
2.61
2.59
2.57
2.55
2.54
1.19
1.17
1.15
MeO2C
O
N
N
Et
3q
400 MHz, CDCl3
1.00
1.02
0.96
1.01
2.06
1.05
1.08
2.01
1.04
1.05
1.09
1.00
3.02
2.04
3.05
f1 (ppm)

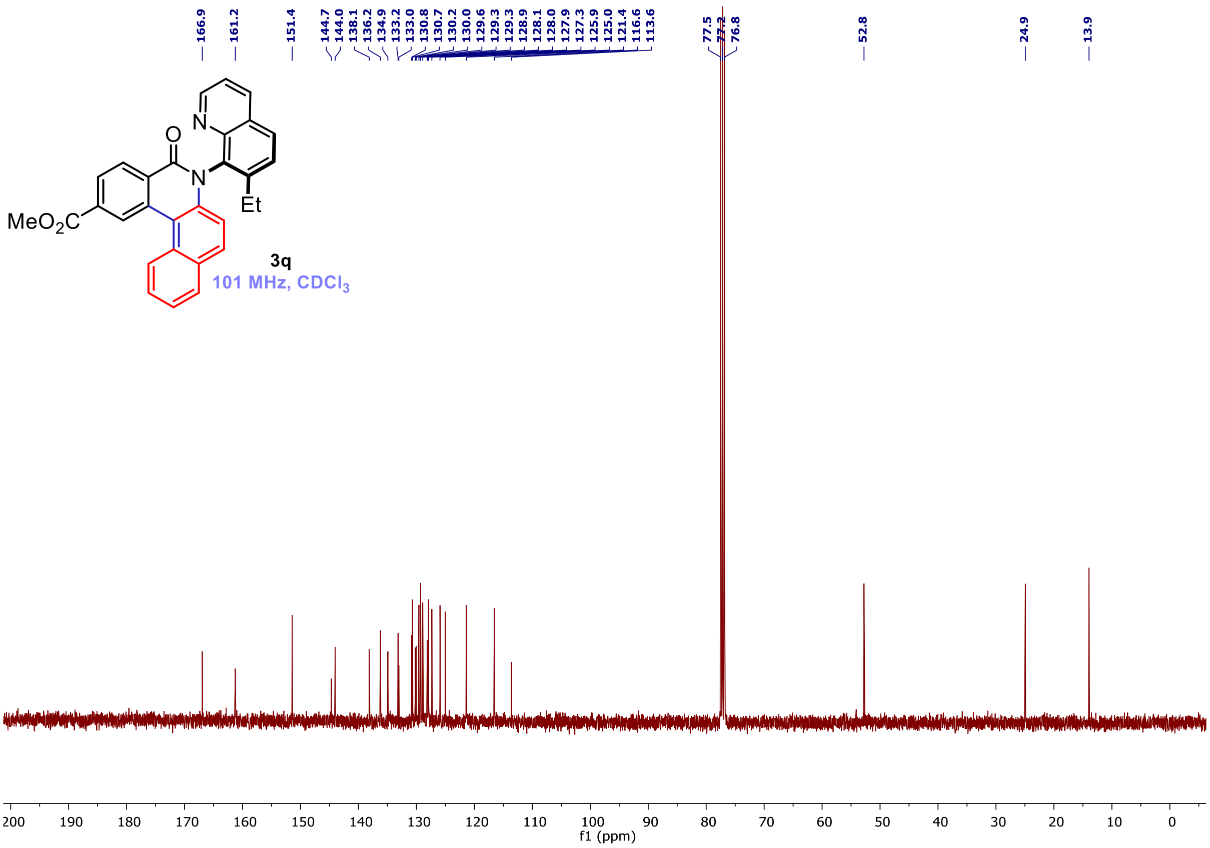

166.9
161.2
151.4
144.7
144.0
138.1
136.2
134.9
133.2
133.0
130.8
130.7
130.2
130.0
129.6
129.3
129.3
128.9
128.1
128.0
127.9
127.3
125.9
125.0
121.4
116.6
113.6
77.5
77.2
76.8
52.8
24.9
13.9
MeO2C
O
N
N
Et
3q
101 MHz, CDCl3
f1 (ppm)

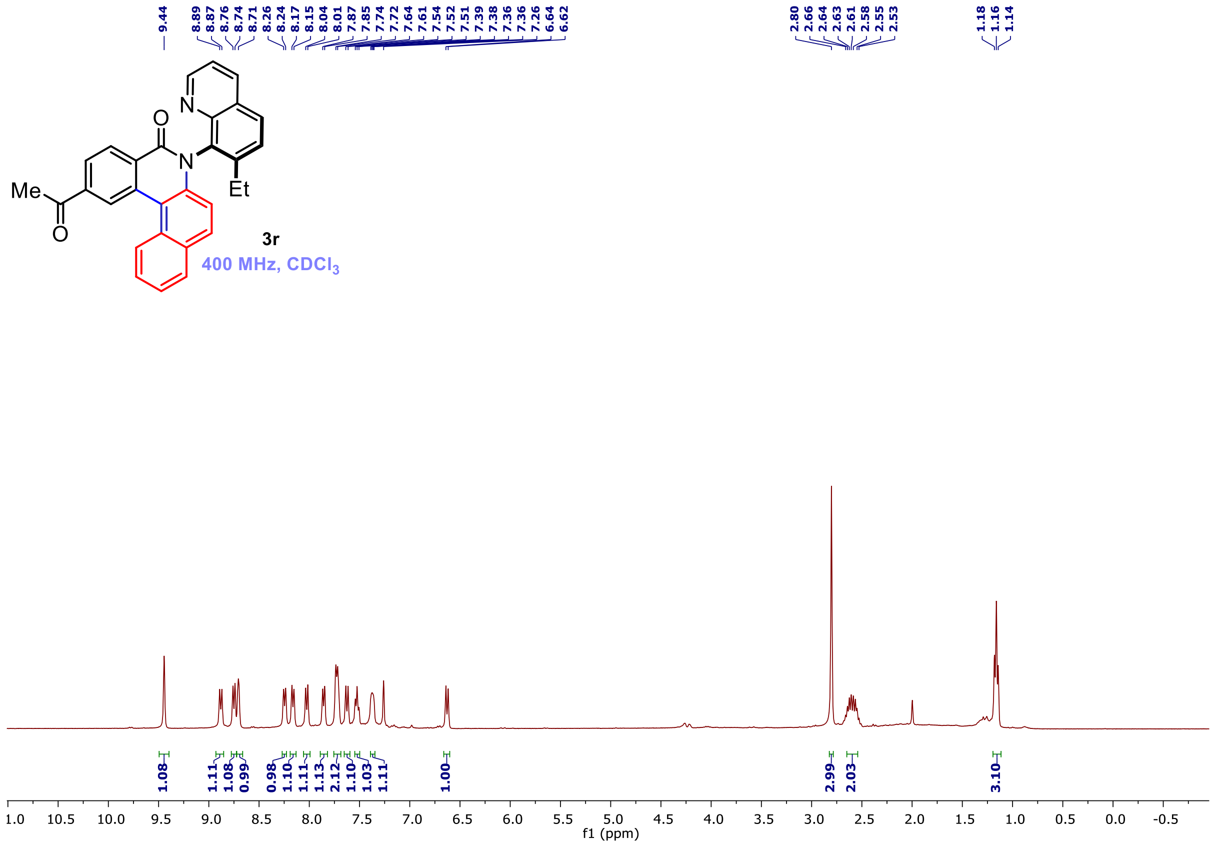

Me
O
N
O
N
Et
3r
400 MHz, CDCl3
f1 (ppm)

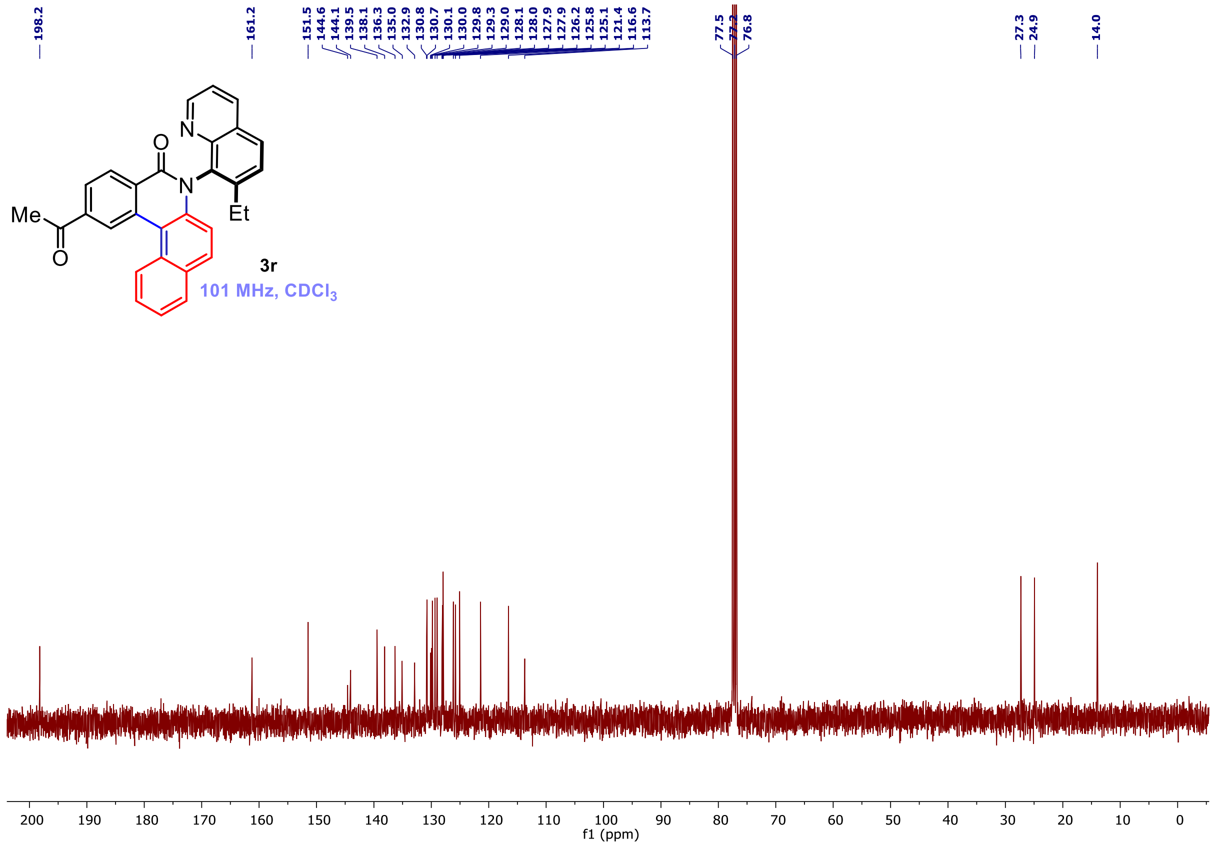

Me
O
N
O
N
Et
3r
101 MHz, CDCl3
f1 (ppm)

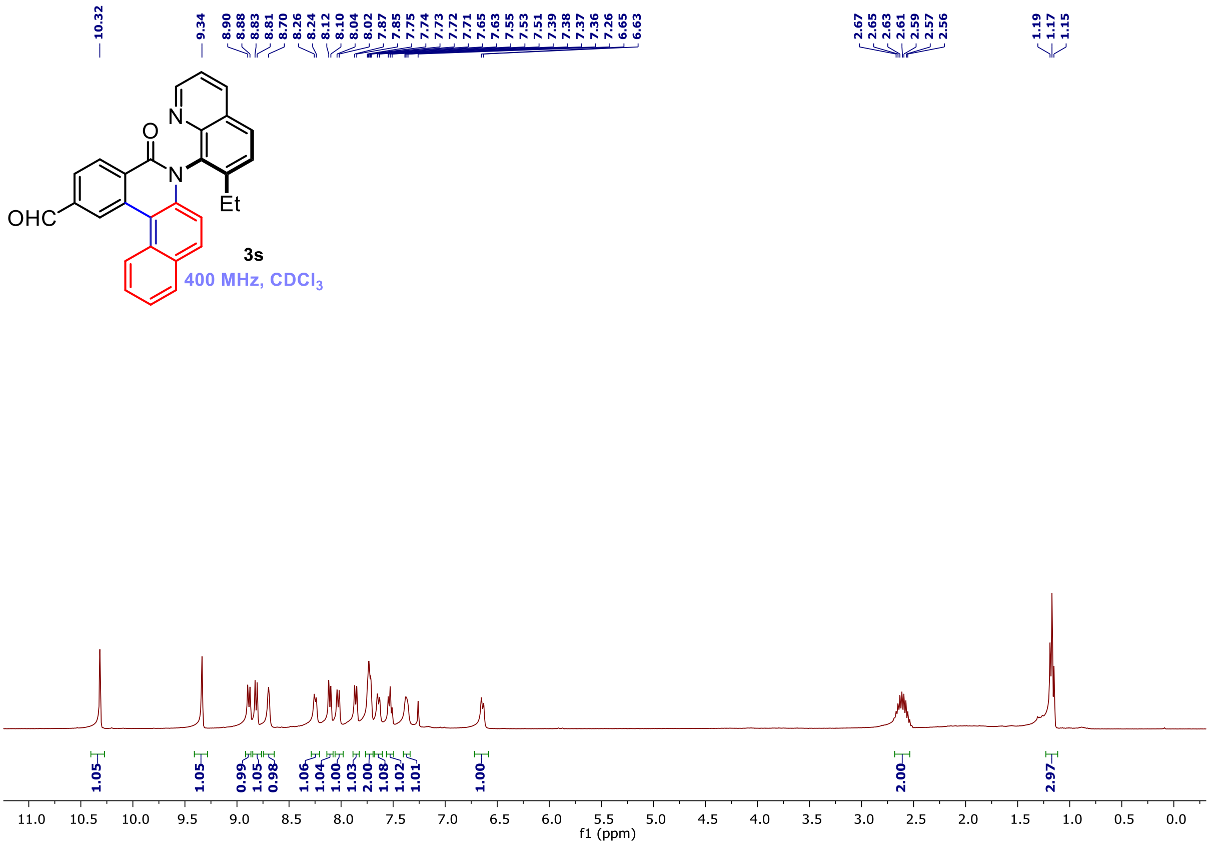

10.32
9.34
8.90 8.88 8.83 8.81 8.70 8.26 8.24 8.12 8.10 8.04 8.02 7.87 7.85 7.75 7.74 7.73 7.72 7.71 7.65 7.63 7.55 7.53 7.51 7.39 7.38 7.37 7.36 7.26 6.65 6.63
2.67 2.65 2.63 2.61 2.59 2.57 2.56
1.19 1.17 1.15
O
N
N
OHC
Et
3s
400 MHz, CDCl3
1.05 1.05 0.99 1.05 0.98 1.06 1.04 1.00 1.03 2.00 1.08 1.02 1.01 1.00 2.00 2.97
11.0 10.5 10.0 9.5 9.0 8.5 8.0 7.5 7.0 6.5 6.0 5.5 5.0 4.5 4.0 3.5 3.0 2.5 2.0 1.5 1.0 0.5 0.0
f1 (ppm)

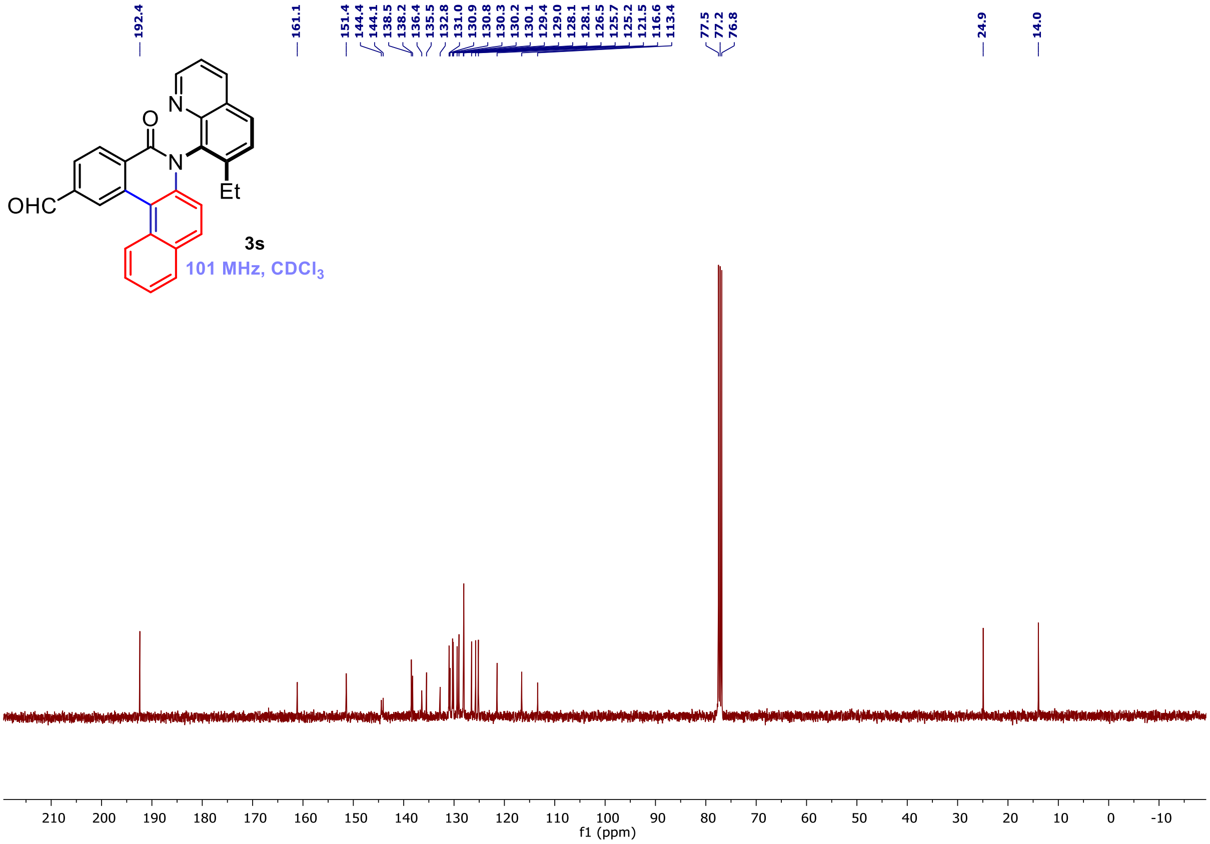

192.4
161.1
151.4 144.4 144.1 138.5 138.2 136.4 135.5 132.8 131.0 130.9 130.8 130.3 130.2 130.1 129.4 129.0 128.1 126.5 125.7 125.2 121.5 116.6 113.4
77.5 77.2 76.8
24.9
14.0
O
N
N
OHC
Et
3s
101 MHz, CDCl3
210 200 190 180 170 160 150 140 130 120 110 100 90 80 70 60 50 40 30 20 10 0 -10
f1 (ppm)

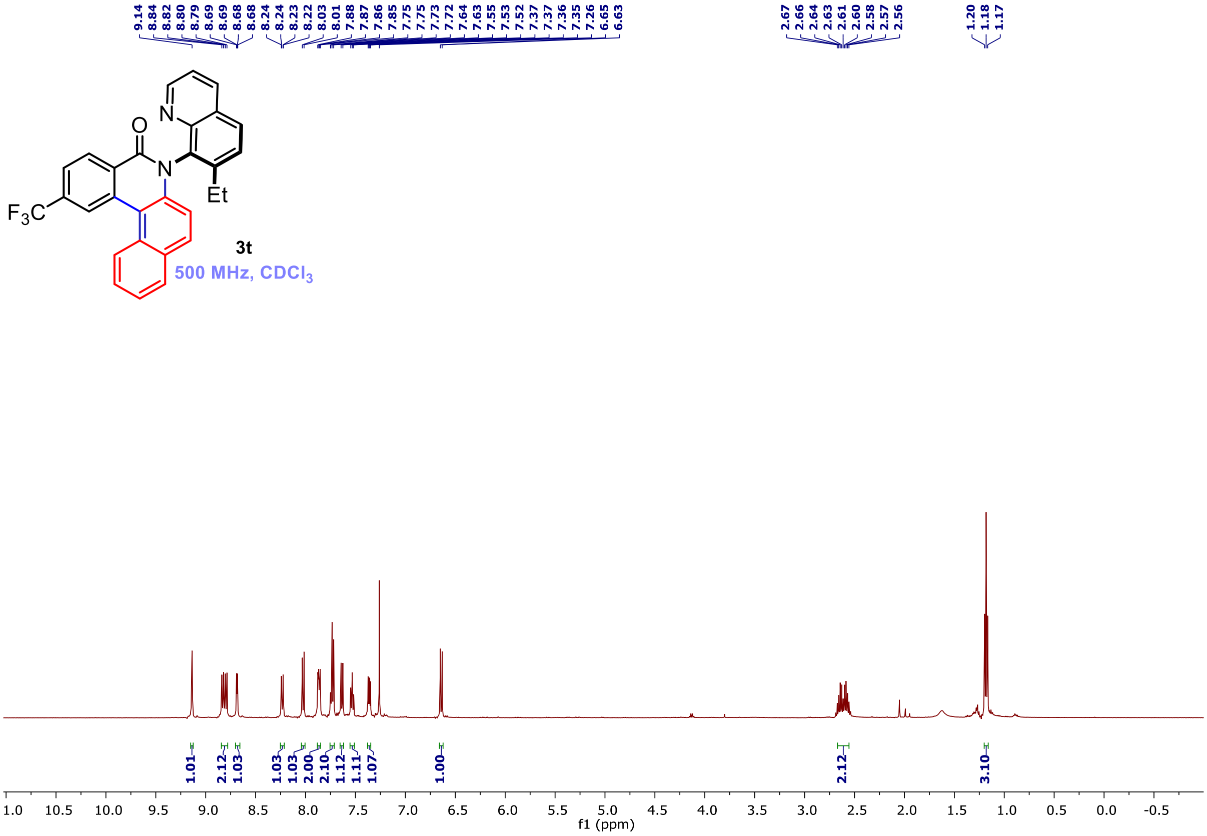
9.14
8.84
8.82
8.80
8.79
8.69
8.69
8.68
8.24
8.24
8.23
8.22
8.03
8.01
7.88
7.87
7.86
7.85
7.75
7.75
7.73
7.72
7.64
7.63
7.55
7.53
7.52
7.37
7.37
7.36
7.35
7.26
6.65
6.63
2.67
2.66
2.64
2.63
2.61
2.60
2.58
2.57
2.56
1.20
1.18
1.17
F3C
N
O
N
Et
3t
500 MHz, CDCl3
1.01
2.12
1.03
1.03
1.03
2.00
2.10
1.12
1.11
1.07
1.00
2.12
3.10
f1 (ppm)

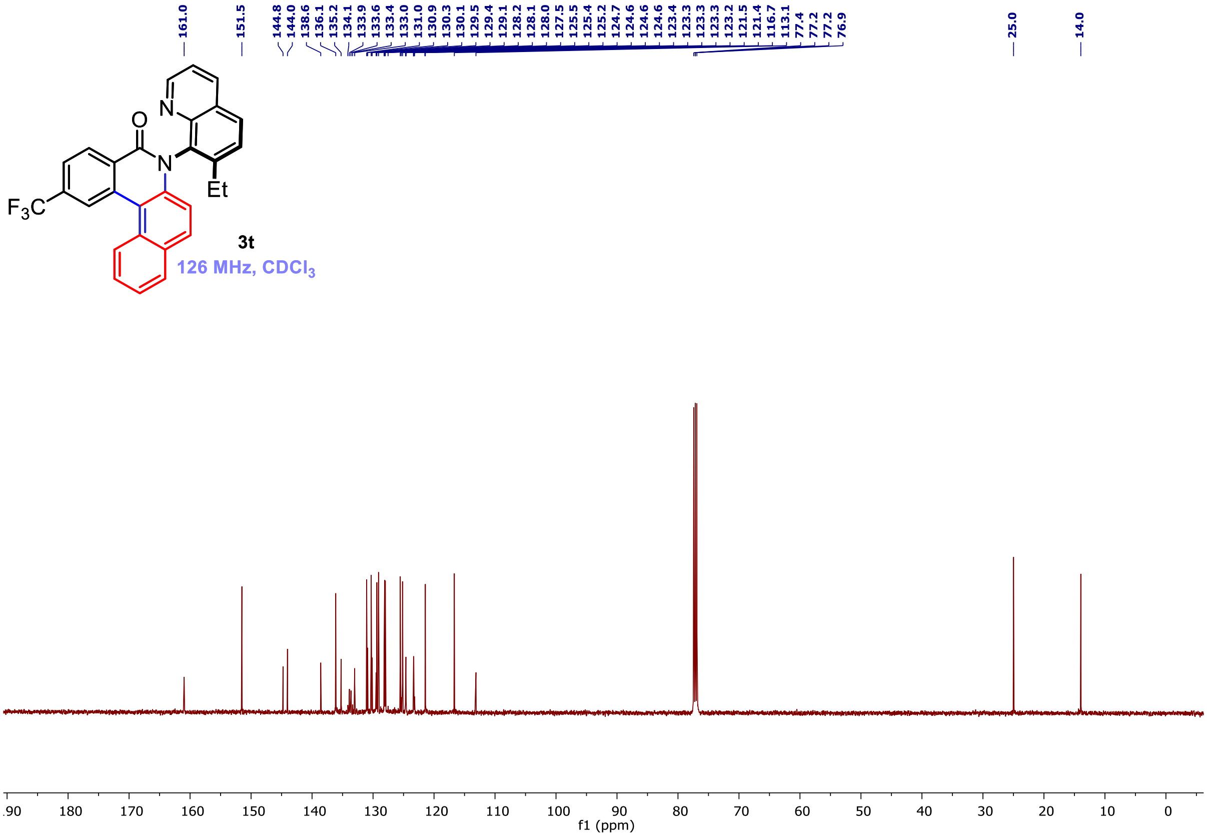
161.0
151.5
144.8
144.0
138.6
136.1
135.2
134.1
133.9
133.6
133.4
133.0
131.0
130.9
130.3
130.1
129.5
129.4
129.1
128.2
128.1
128.0
127.5
125.5
125.4
125.2
124.7
124.6
124.6
123.4
123.3
123.3
123.3
123.2
121.5
121.4
116.7
113.1
77.4
77.2
77.2
76.9
25.0
14.0
F3C
N
O
N
Et
3t
126 MHz, CDCl3
f1 (ppm)

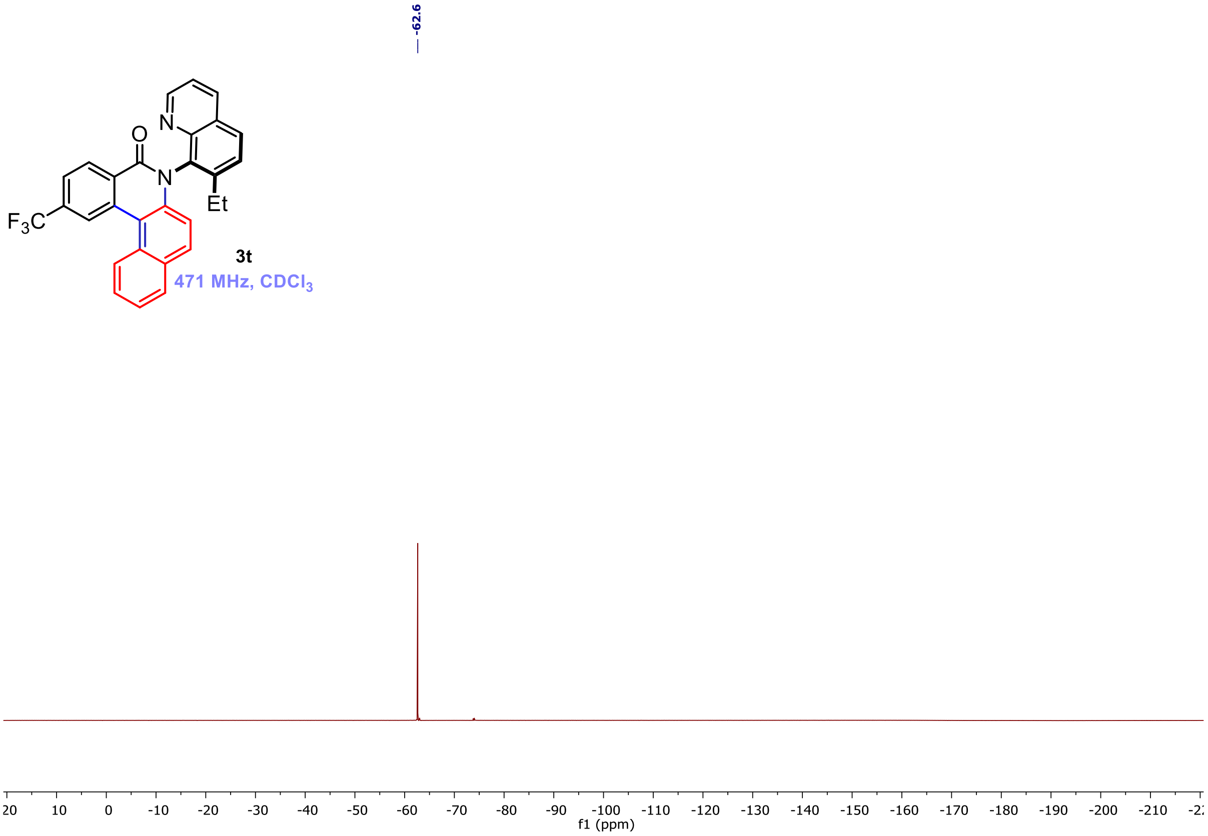
-62.6
3t
471 MHz, CDCl3
20
10
0
-10
-20
-30
-40
-50
-60
-70
-80
-90
-100
-110
-120
-130
-140
-150
-160
-170
-180
-190
-200
-210
f1 (ppm)

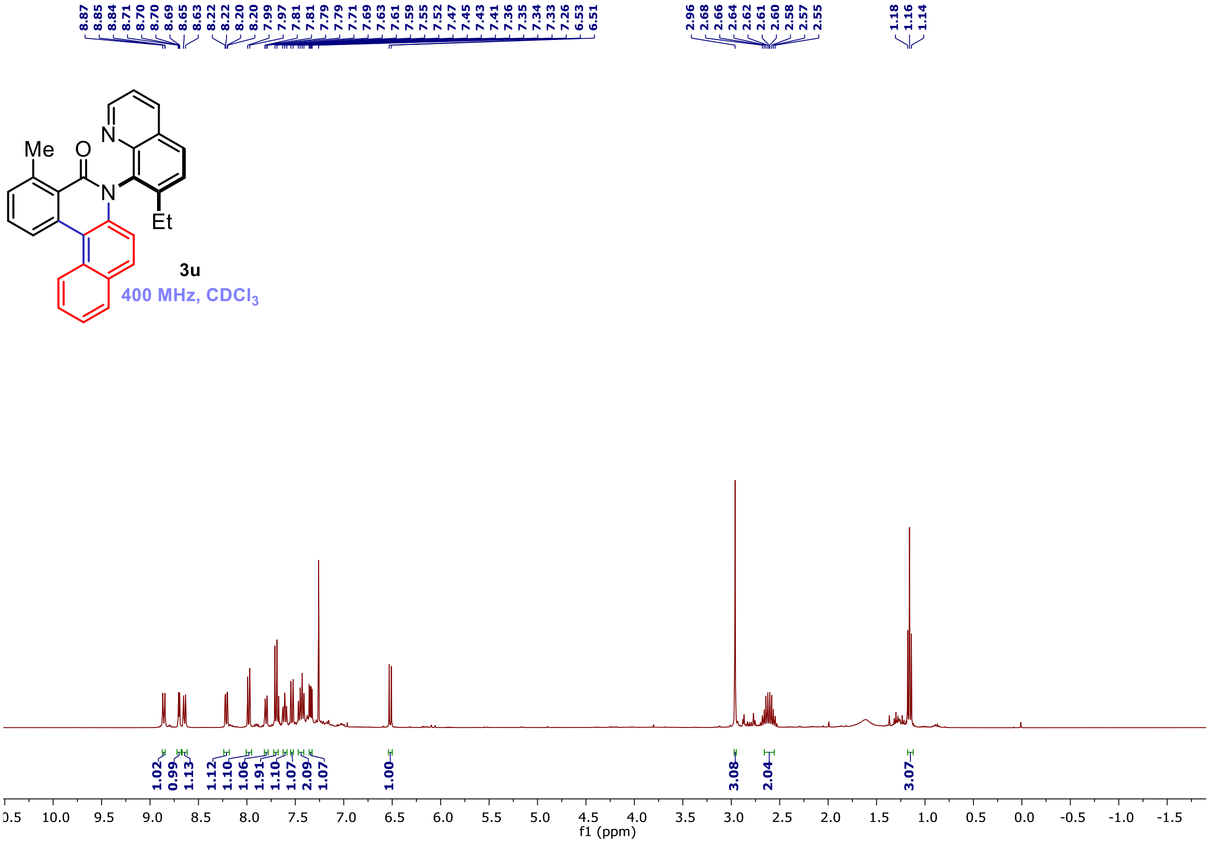
8.87 8.85 8.84 8.71 8.70 8.70 8.69 8.65 8.63 8.22 8.22 8.20 8.20 7.99 7.97 7.81 7.79 7.79 7.71 7.69 7.63 7.61 7.59 7.55 7.52 7.47 7.45 7.43 7.41 7.36 7.35 7.34 7.33 7.26 6.53 6.51
2.96 2.68 2.66 2.64 2.62 2.61 2.60 2.58 2.57 2.55
1.18 1.16 1.14
Me
O
N
N
Et
3u
400 MHz, CDCl3
1.02 0.99 1.13 1.12 1.10 1.06 1.91 1.10 1.07 2.09 1.07
1.00
3.08 2.04
3.07
f1 (ppm)

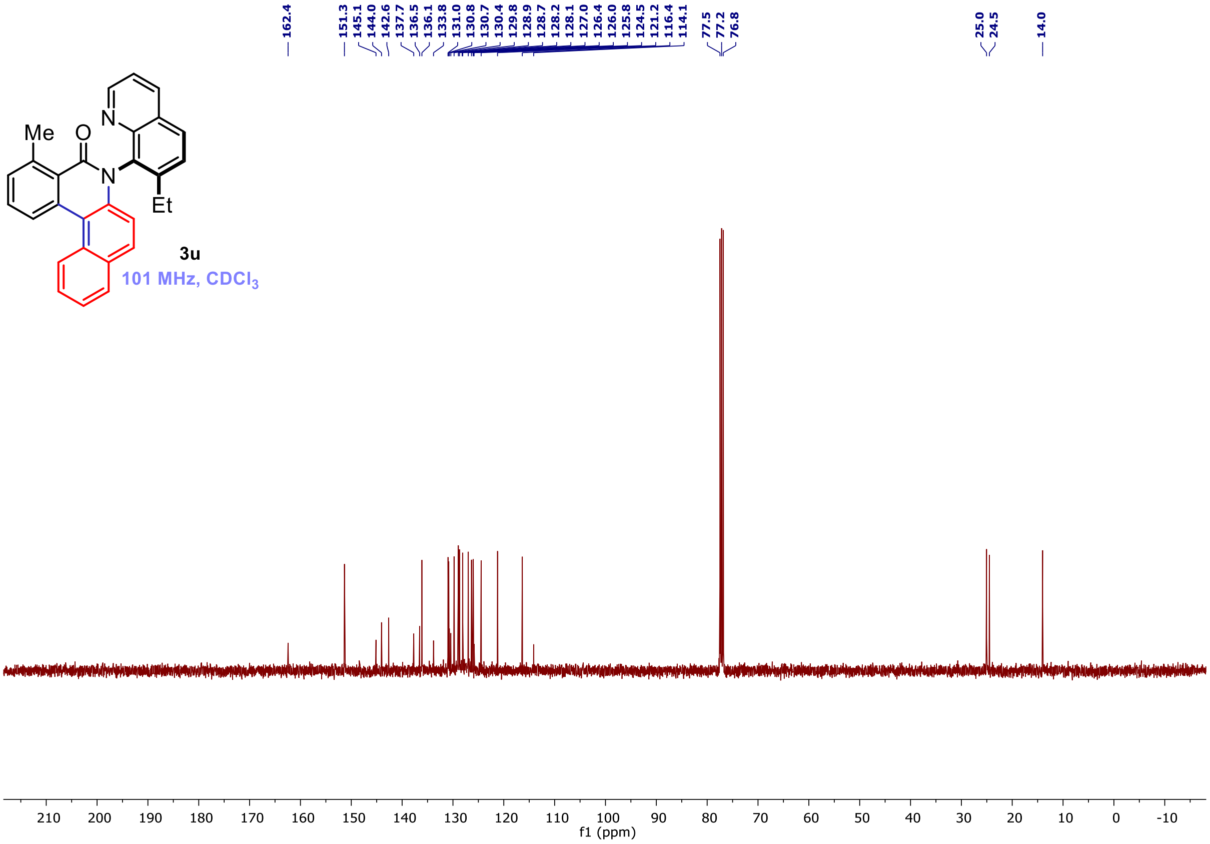
162.4
151.3 145.1 144.0 142.6 137.7 136.5 136.1 133.8 131.0 130.8 130.7 130.4 129.8 128.9 128.7 128.2 128.1 127.0 126.4 126.0 125.8 124.5 121.2 116.4 114.1
77.5 77.2 76.8
25.0 24.5
14.0
Me
O
N
N
Et
3u
101 MHz, CDCl3
f1 (ppm)

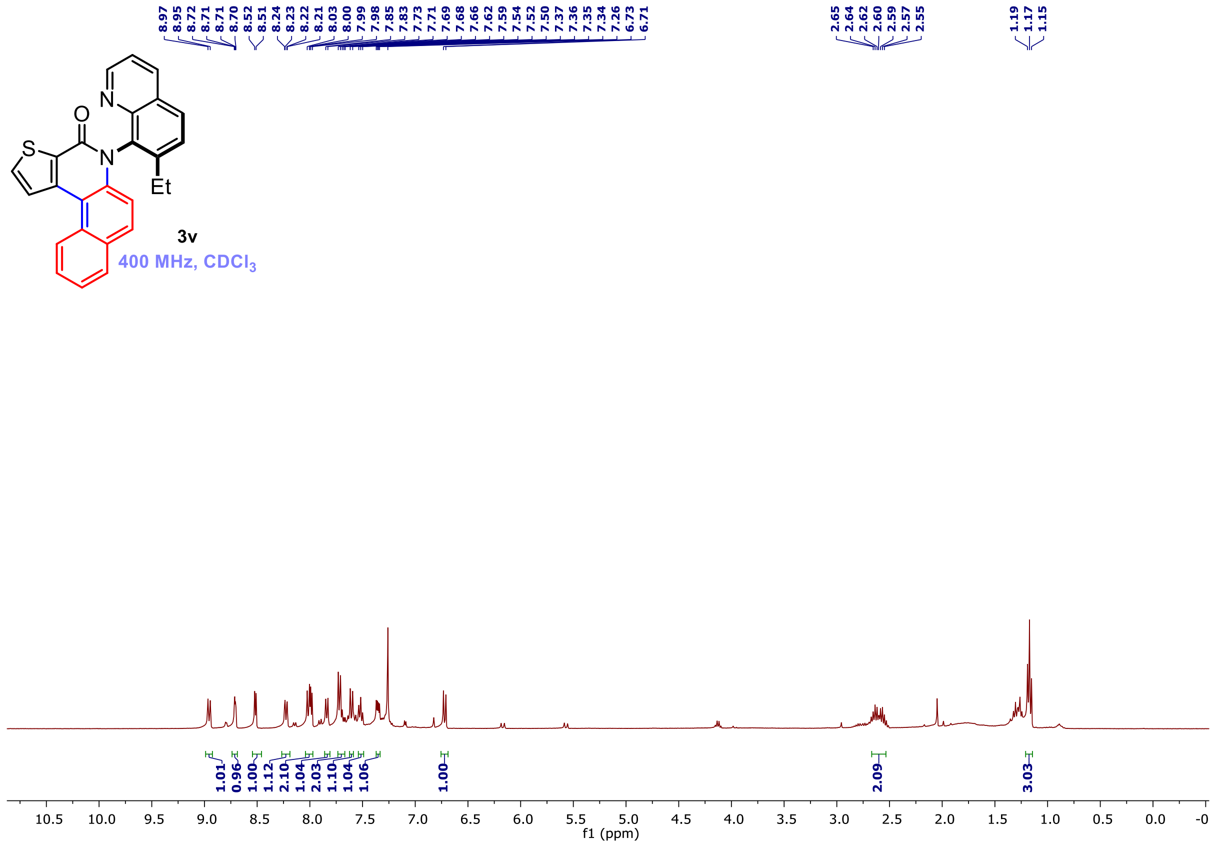
8.97
8.95
8.72
8.71
8.71
8.70
8.52
8.51
8.24
8.23
8.22
8.21
8.03
8.00
7.99
7.98
7.85
7.83
7.73
7.71
7.69
7.68
7.66
7.62
7.59
7.54
7.52
7.50
7.37
7.36
7.35
7.34
7.26
6.73
6.71
2.65
2.64
2.62
2.60
2.59
2.57
2.55
1.19
1.17
1.15
S
O
N
N
Et
3v
400 MHz, CDCl3
1.01
0.96
1.00
1.12
2.10
1.04
2.03
1.10
1.04
1.06
1.00
2.09
3.03
10.5
10.0
9.5
9.0
8.5
8.0
7.5
7.0
6.5
6.0
5.5
5.0
4.5
4.0
3.5
3.0
2.5
2.0
1.5
1.0
0.5
0.0
-0
f1 (ppm)

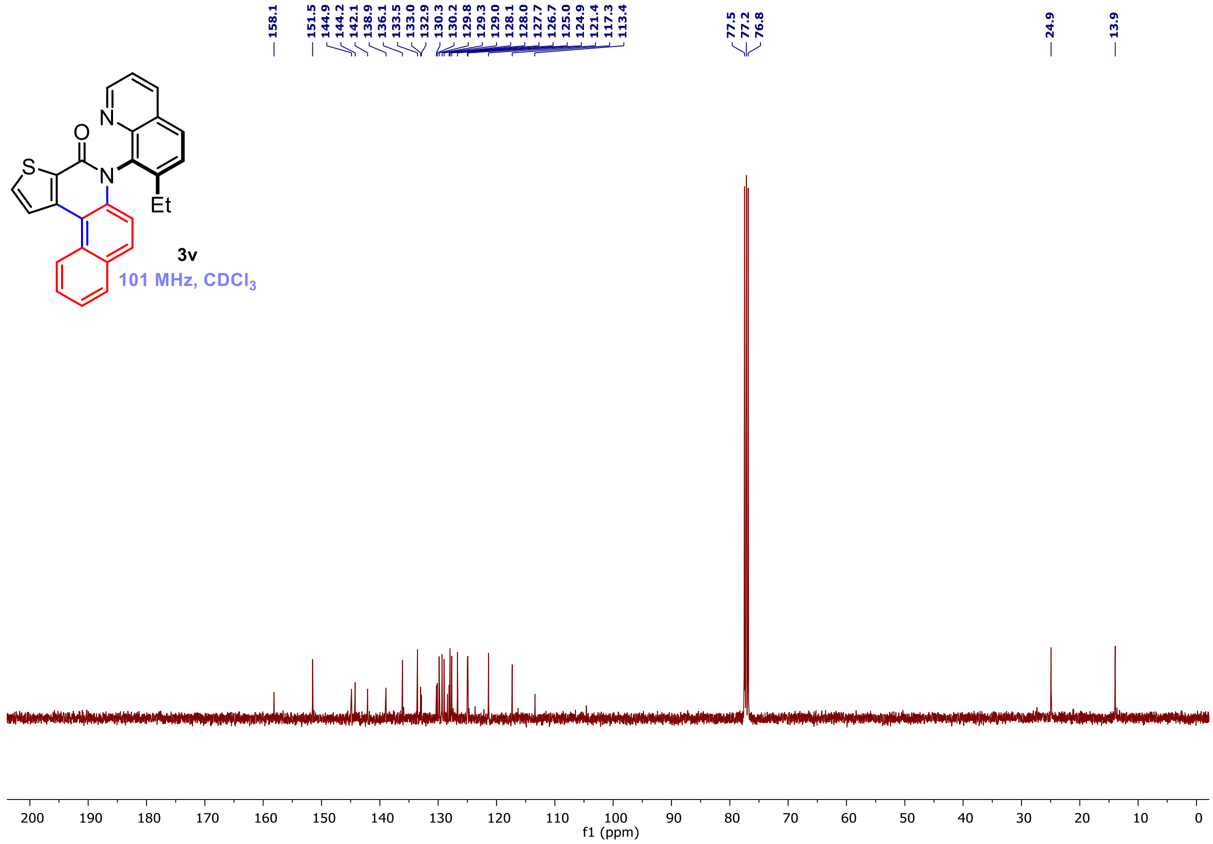
158.1
151.5
144.9
144.2
142.1
138.9
136.1
133.5
133.0
132.9
130.3
130.2
129.8
129.3
129.0
128.1
128.0
127.7
126.7
125.0
124.9
121.4
117.3
113.4
77.5
77.2
76.8
24.9
13.9
S
O
N
N
Et
3v
101 MHz, CDCl3
200
190
180
170
160
150
140
130
120
110
100
90
80
70
60
50
40
30
20
10
0
f1 (ppm)

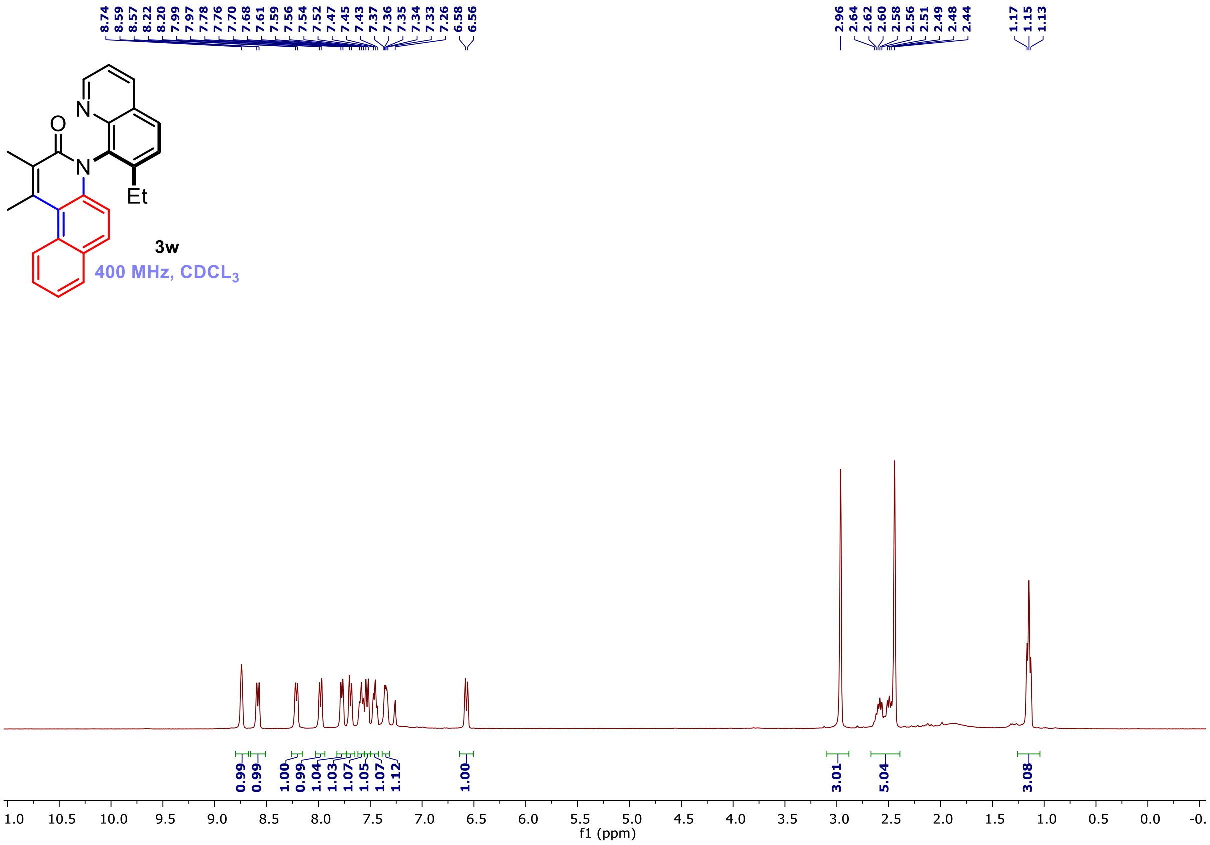

8.74 8.59 8.57 8.22 8.20 7.99 7.97 7.78 7.76 7.70 7.68 7.61 7.59 7.56 7.54 7.52 7.47 7.45 7.43 7.37 7.36 7.35 7.34 7.33 7.26 6.58 6.56
2.96 2.64 2.62 2.60 2.58 2.56 2.51 2.49 2.48 2.44
1.17 1.15 1.13
O
N
N
Et
3w
400 MHz, CDCL3
0.99 0.99 1.00 0.99 1.04 1.03 1.07 1.05 1.07 1.12 1.00 3.01 5.04 3.08
f1 (ppm)

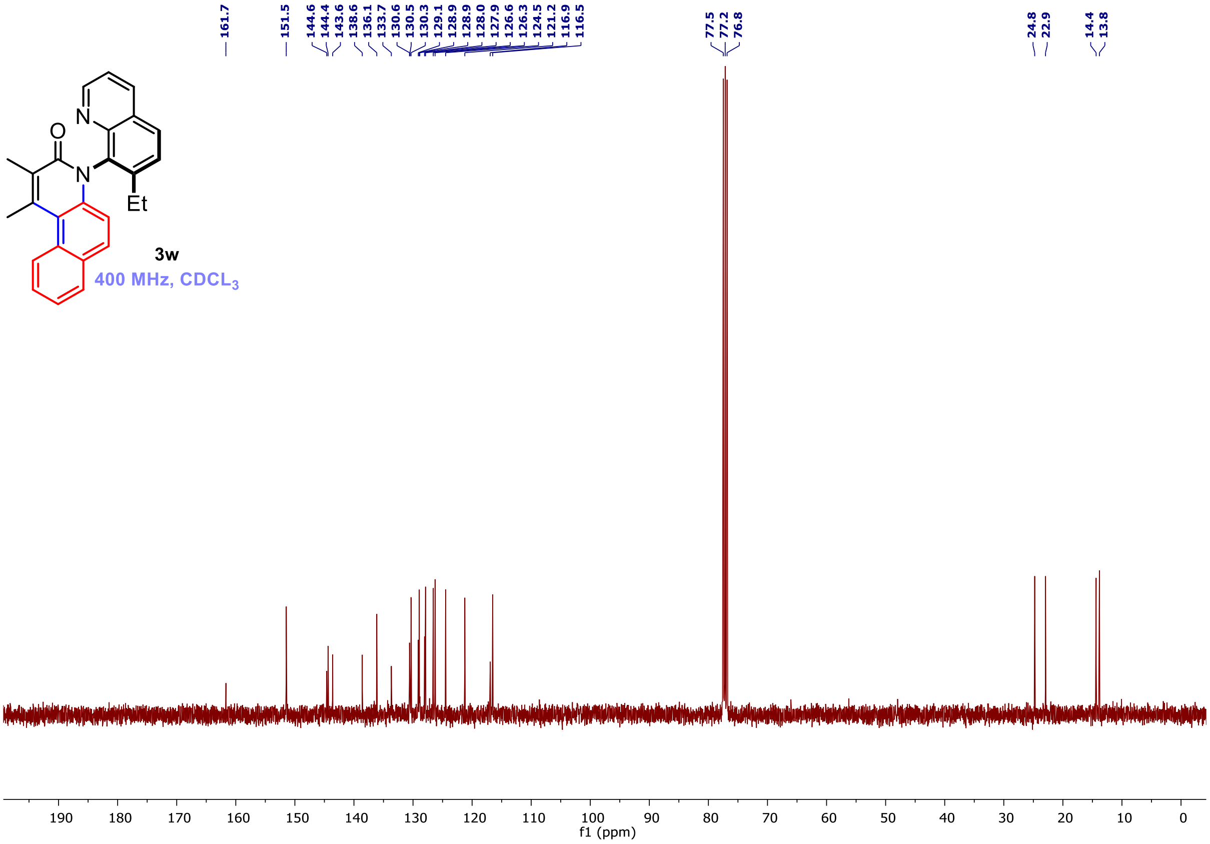

161.7 151.5 144.6 144.4 143.6 138.6 136.1 133.7 130.6 130.5 130.3 129.1 128.9 128.9 128.0 127.9 126.6 126.3 124.5 121.2 116.9 116.5
77.5 77.2 76.8
24.8 22.9 14.4 13.8
O
N
N
Et
3w
400 MHz, CDCL3
f1 (ppm)

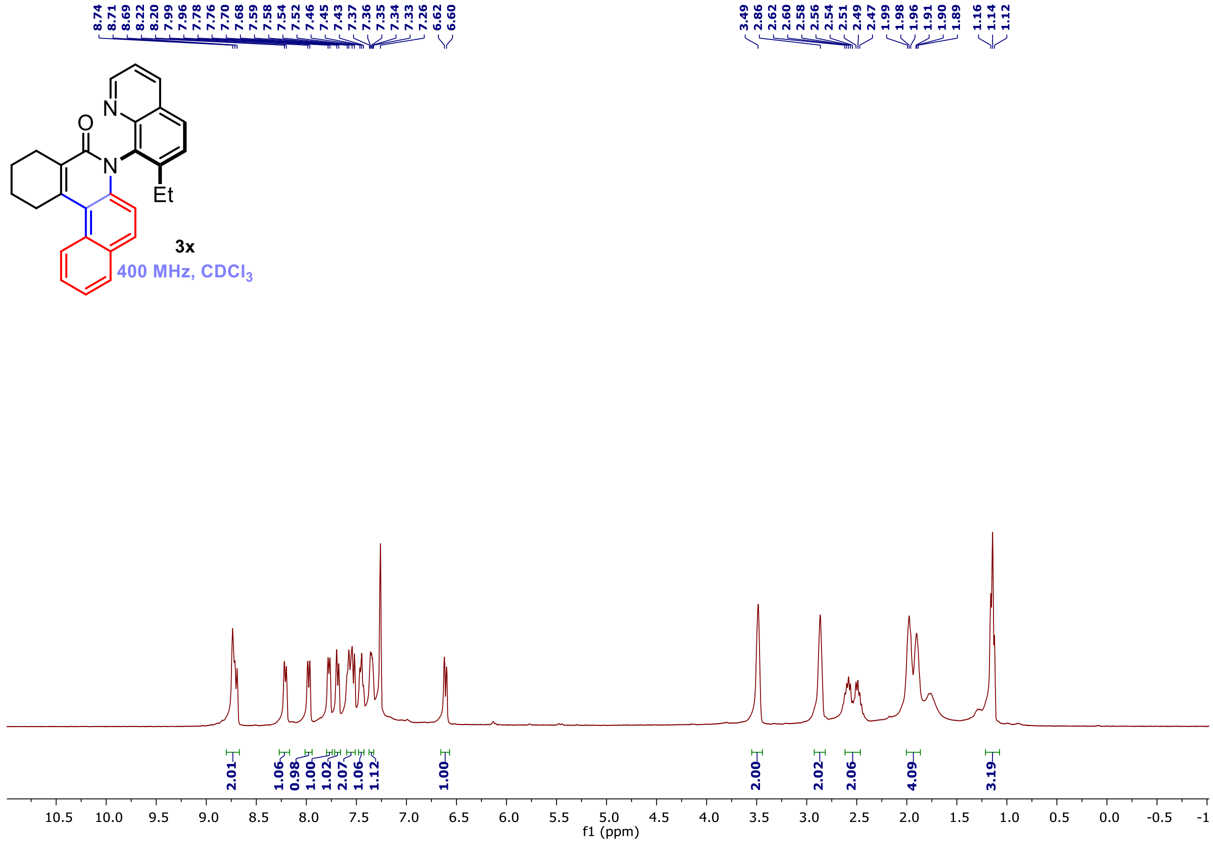
8.74 8.71 8.69 8.22 8.20 7.99 7.96 7.78 7.76 7.70 7.68 7.59 7.58 7.54 7.52 7.46 7.45 7.43 7.37 7.36 7.35 7.34 7.33 7.26 6.62 6.60
3.49 2.86 2.62 2.60 2.58 2.56 2.54 2.51 2.49 2.47 1.99 1.98 1.96 1.91 1.90 1.89 1.16 1.14 1.12
O
N
N
Et
3x
400 MHz, CDCl3
2.01 1.06 0.98 1.00 1.02 2.07 1.06 1.12 1.00 2.00 2.02 2.06 4.09 3.19
10.5 10.0 9.5 9.0 8.5 8.0 7.5 7.0 6.5 6.0 5.5 5.0 4.5 4.0 3.5 3.0 2.5 2.0 1.5 1.0 0.5 0.0 -0.5 -1
f1 (ppm)

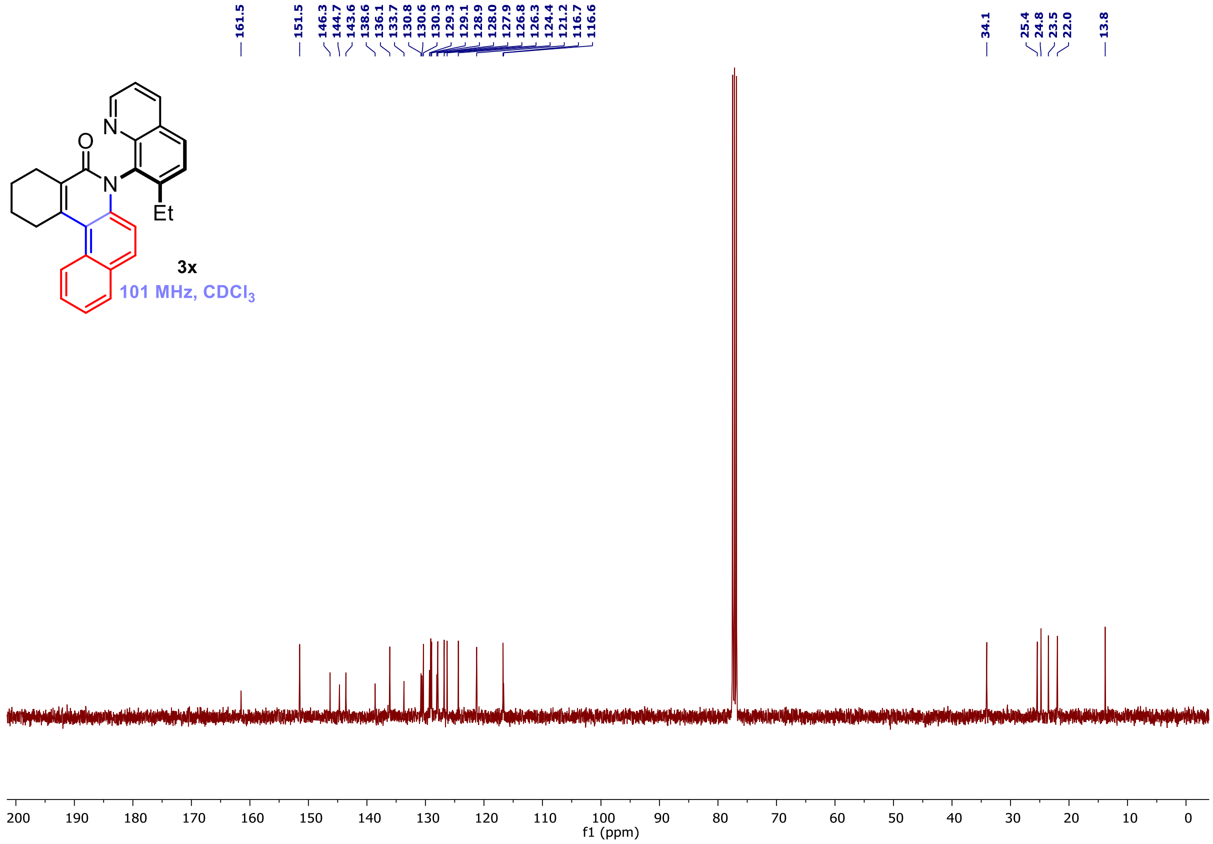
161.5
151.5
146.3 144.7 143.6 138.6 136.1 133.7 130.8 130.6 130.3 129.3 129.1 128.9 128.0 127.9 126.8 126.3 124.4 121.2 116.7 116.6
34.1
25.4 24.8 23.5 22.0
13.8
O
N
N
Et
3x
101 MHz, CDCl3
200 190 180 170 160 150 140 130 120 110 100 90 80 70 60 50 40 30 20 10 0
f1 (ppm)

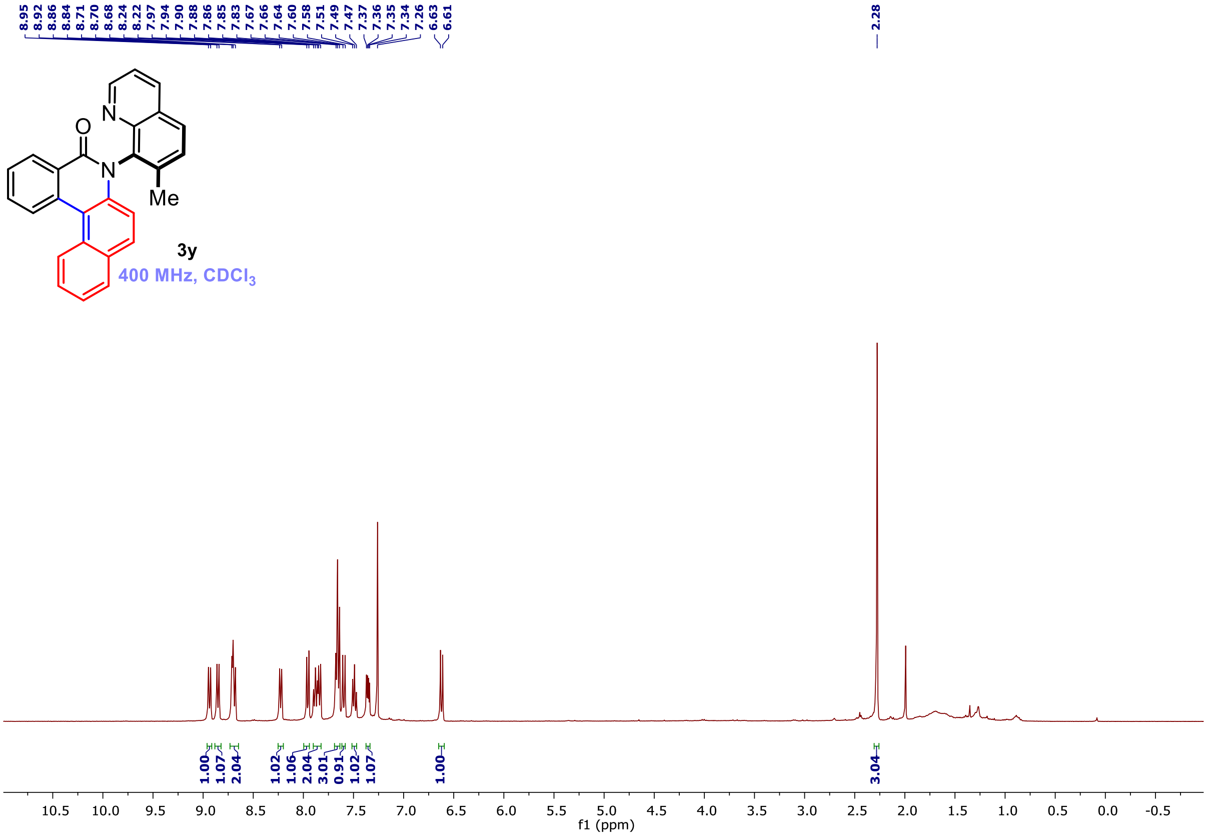
8.95 8.92 8.86 8.84 8.71 8.70 8.68 8.24 8.22 7.97 7.94 7.90 7.88 7.86 7.85 7.83 7.67 7.66 7.64 7.60 7.58 7.51 7.49 7.47 7.37 7.36 7.35 7.34 7.26 6.63 6.61
2.28
O
N
N
Me
3y
400 MHz, $CDCl_3$
1.00 1.07 2.04 1.02 1.06 2.04 3.01 0.91 1.02 1.07 1.00
3.04
f1 (ppm)

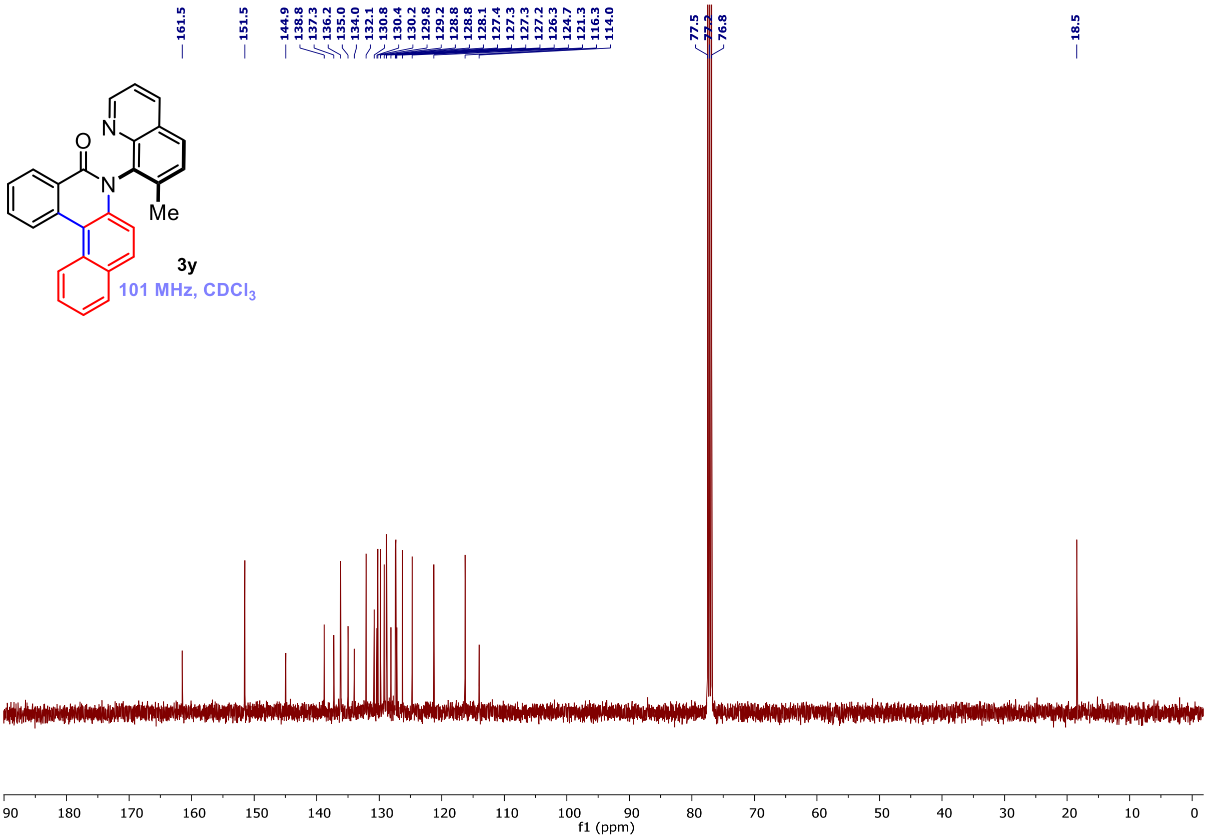
161.5
151.5
144.9 138.8 137.3 136.2 135.0 134.0 132.1 130.8 130.4 130.2 129.8 129.2 128.8 128.1 127.4 127.3 127.2 126.3 124.7 121.3 116.3 114.0
77.5 77.2 76.8
18.5
O
N
N
Me
3y
101 MHz, $CDCl_3$
f1 (ppm)

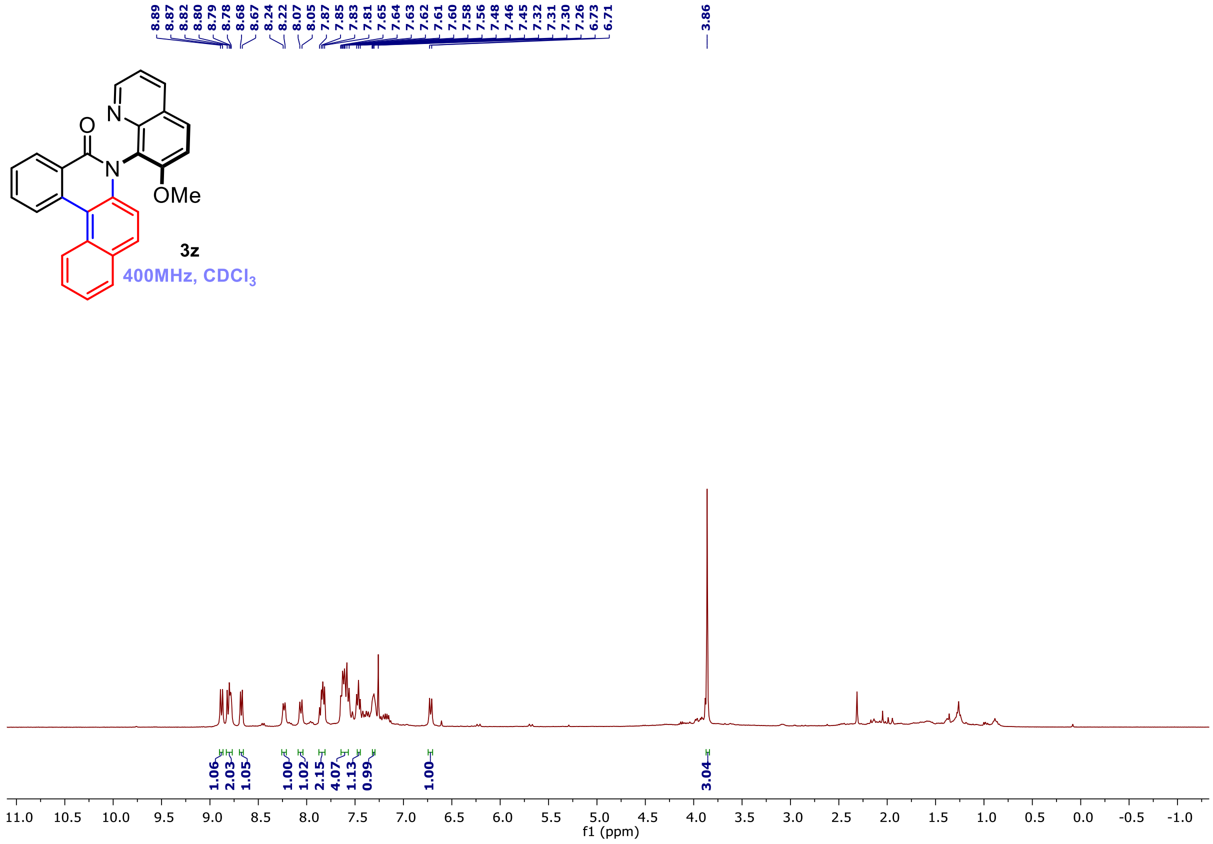
8.89 8.87 8.82 8.80 8.79 8.78 8.68 8.67 8.24 8.22 8.07 8.05 7.87 7.85 7.83 7.81 7.65 7.64 7.63 7.62 7.61 7.60 7.58 7.56 7.48 7.46 7.45 7.32 7.31 7.30 7.26 6.73 6.71
3.86
N
O
N
OMe
3z
400MHz, CDCl3
1.06 2.03 1.05 1.00 1.02 2.15 4.07 1.13 0.99 1.00 3.04
f1 (ppm)

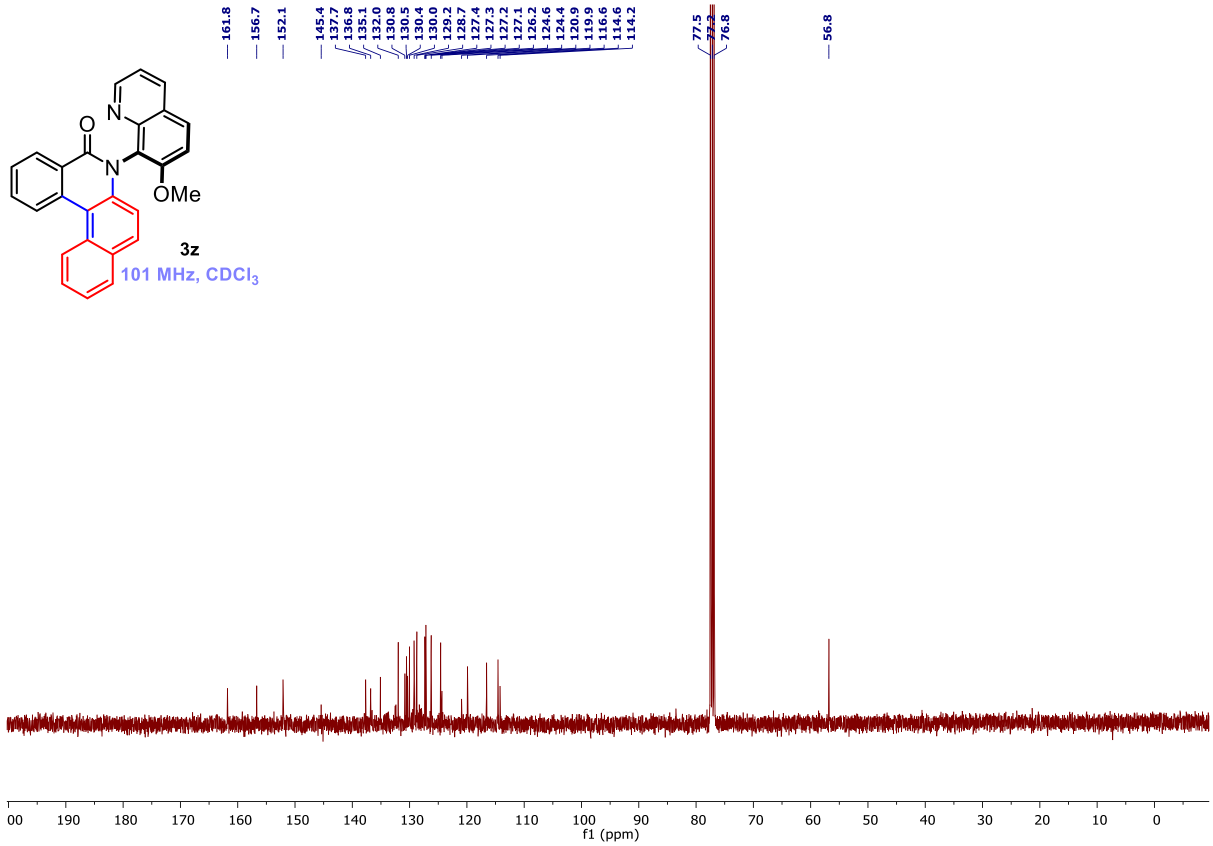
161.8 156.7 152.1 145.4 137.7 136.8 135.1 132.0 130.8 130.5 130.4 130.0 129.2 128.7 127.4 127.3 127.2 127.1 126.2 124.6 124.4 120.9 119.9 116.6 114.6 114.2
77.5 77.2 76.8
56.8
N
O
N
OMe
3z
101 MHz, CDCl3
f1 (ppm)

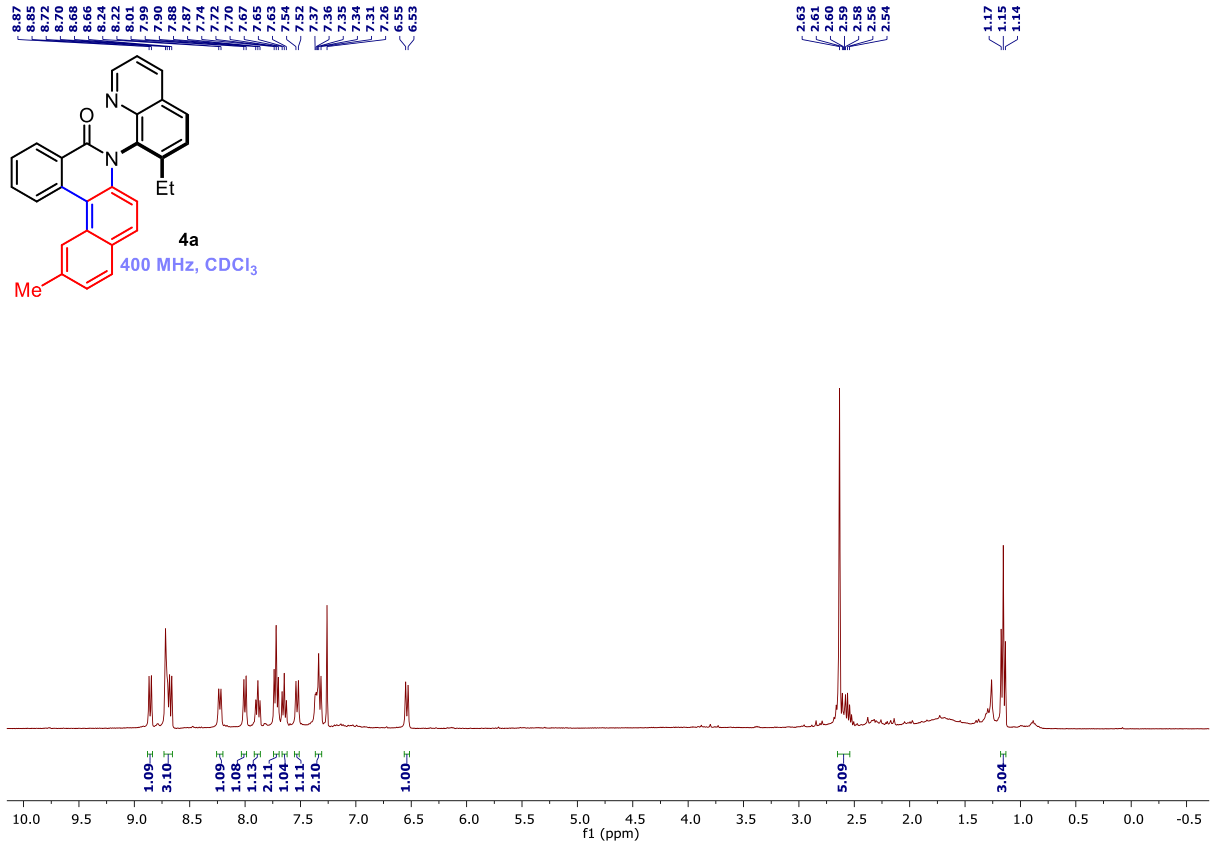

8.87
8.85
8.72
8.70
8.68
8.66
8.24
8.22
8.01
7.99
7.90
7.88
7.87
7.74
7.72
7.70
7.67
7.65
7.63
7.54
7.52
7.37
7.36
7.35
7.34
7.31
7.26
6.55
6.53
2.63
2.61
2.60
2.59
2.58
2.56
2.54
1.17
1.15
1.14
N
O
N
Et
Me
4a
400 MHz, CDCl3
1.09
3.10
1.09
1.08
1.13
2.11
1.04
1.11
2.10
1.00
5.09
3.04
f1 (ppm)

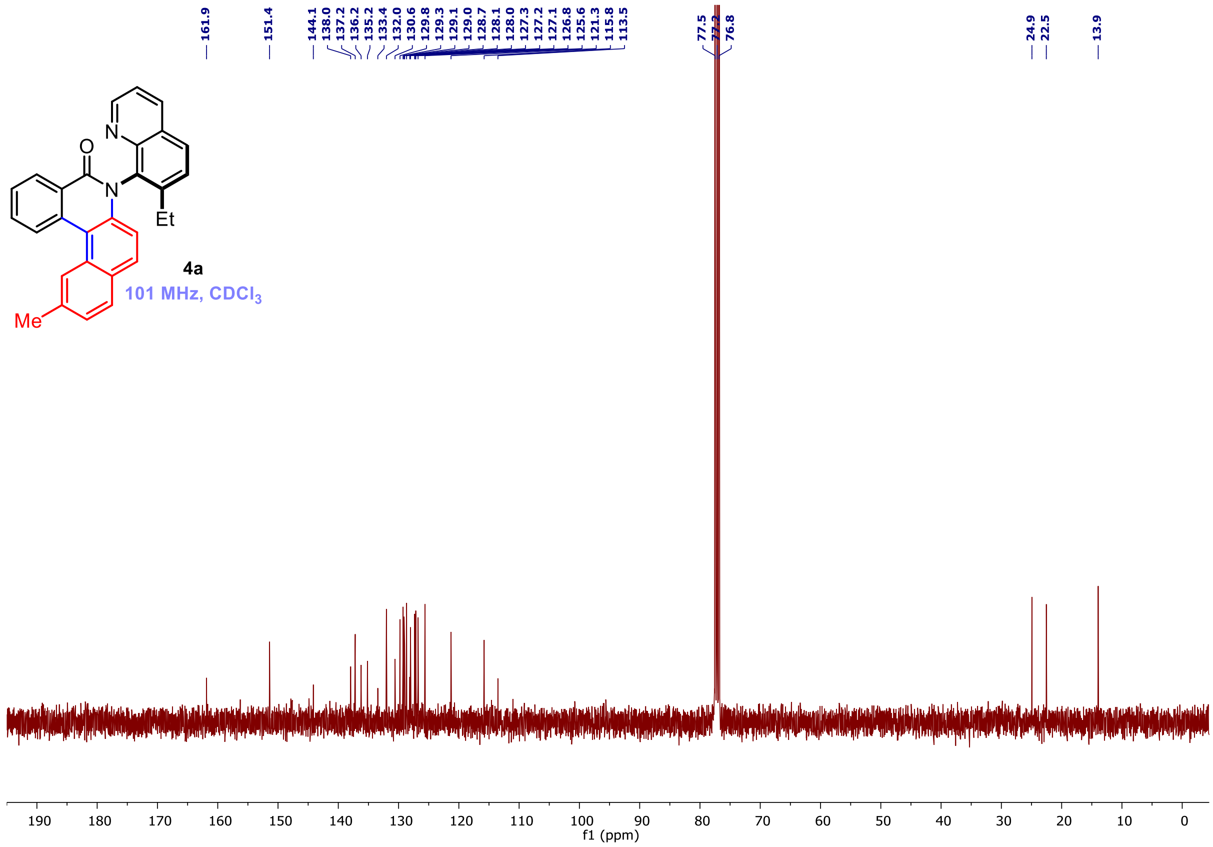

161.9
151.4
144.1
138.0
137.2
136.2
135.2
133.4
132.0
130.6
129.8
129.3
129.1
129.0
128.7
128.1
128.0
127.3
127.2
127.1
126.8
125.6
121.3
115.8
113.5
77.5
76.8
24.9
22.5
13.9
N
O
N
Et
Me
4a
101 MHz, CDCl3
f1 (ppm)

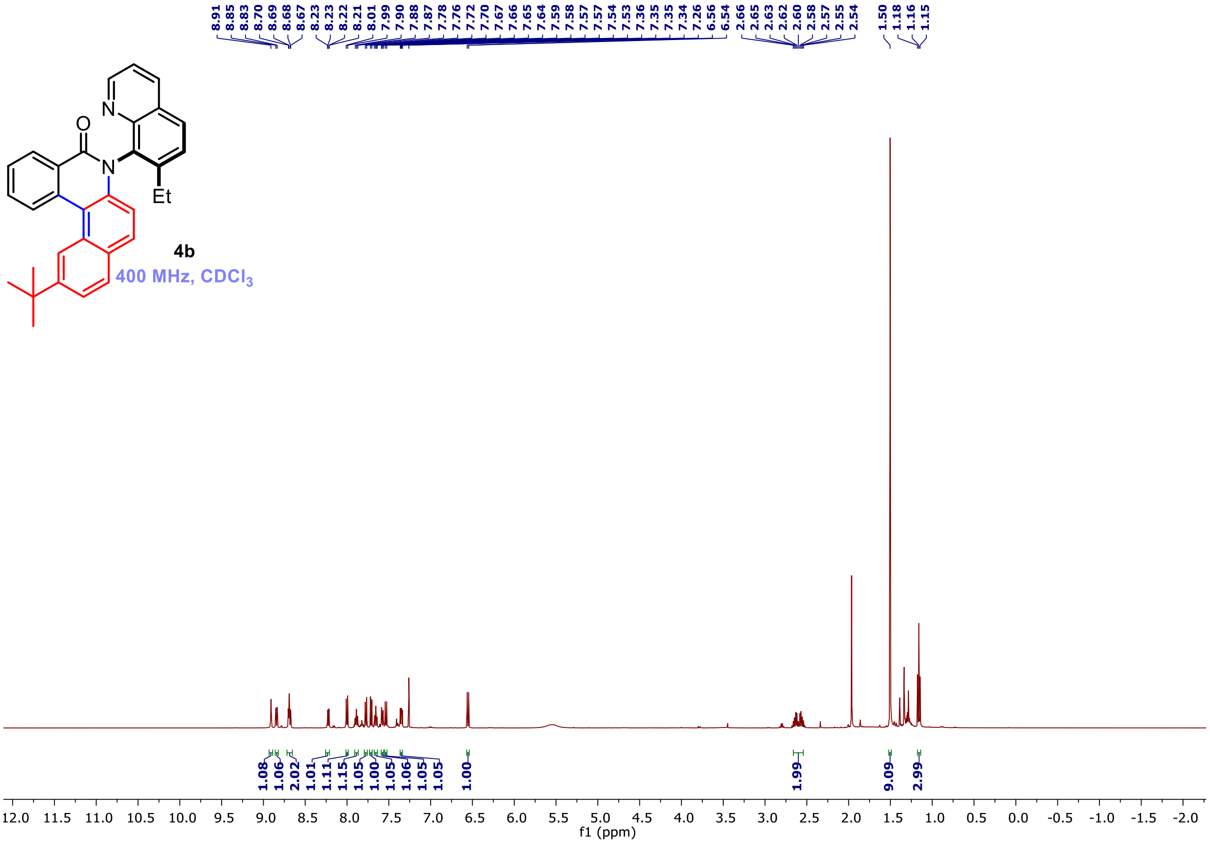
8.91 8.85 8.83 8.70 8.69 8.68 8.67 8.23 8.23 8.22 8.21 8.01 7.99 7.90 7.88 7.87 7.78 7.76 7.72 7.70 7.67 7.66 7.65 7.64 7.59 7.58 7.57 7.57 7.54 7.53 7.36 7.35 7.35 7.34 7.26 6.56 6.54 2.66 2.65 2.63 2.62 2.60 2.58 2.57 2.55 2.54 1.50 1.18 1.16 1.15
O
N
N
Et
4b
400 MHz, CDCl3
1.08 1.06 2.02 1.01 1.11 1.15 1.05 1.00 1.05 1.06 1.05 1.05 1.00 1.99 9.09 2.99
12.0 11.5 11.0 10.5 10.0 9.5 9.0 8.5 8.0 7.5 7.0 6.5 6.0 5.5 5.0 4.5 4.0 3.5 3.0 2.5 2.0 1.5 1.0 0.5 0.0 -0.5 -1.0 -1.5 -2.0
f1 (ppm)

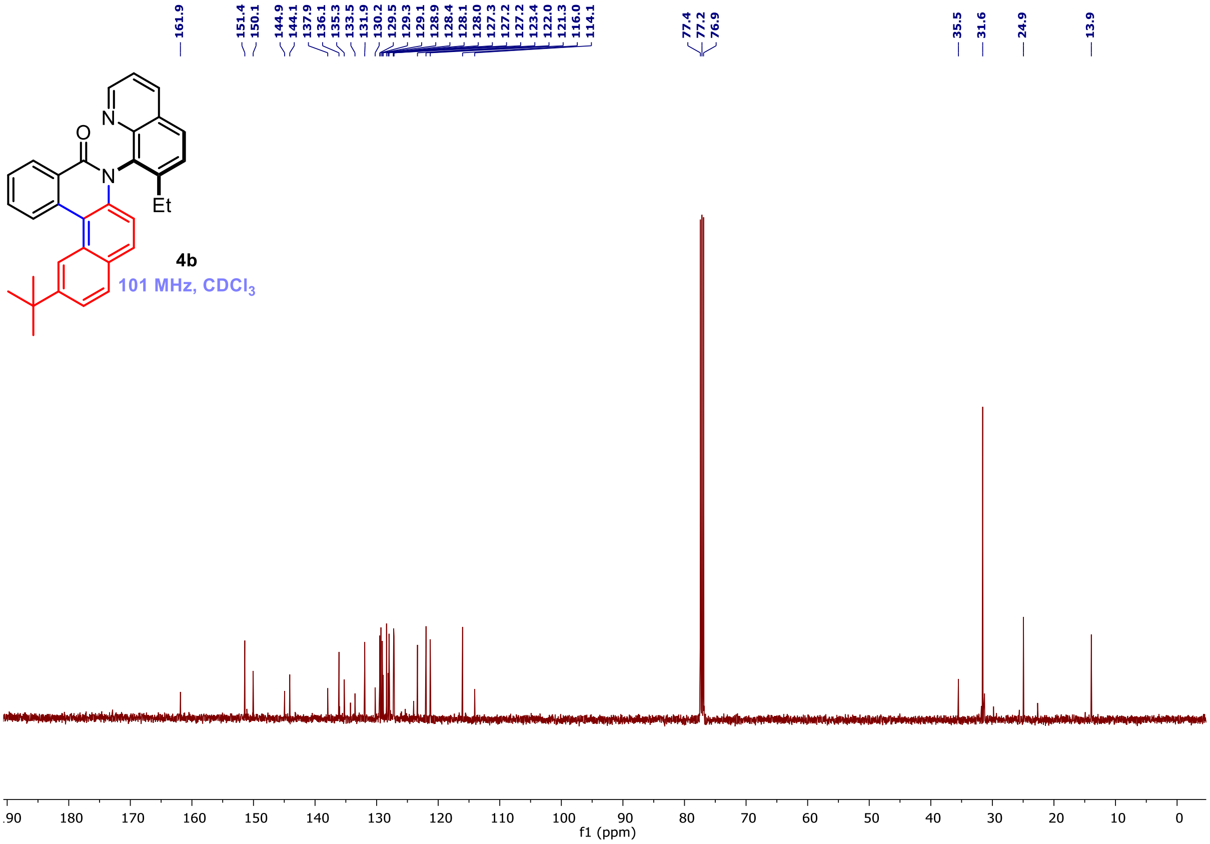
161.9 151.4 150.1 144.9 144.1 137.9 136.1 135.3 133.5 131.9 130.2 129.5 129.3 129.1 128.9 128.4 128.1 128.0 127.3 127.2 123.4 122.0 121.3 116.0 114.1 77.4 77.2 76.9 35.5 31.6 24.9 13.9
O
N
N
Et
4b
101 MHz, CDCl3
190 180 170 160 150 140 130 120 110 100 90 80 70 60 50 40 30 20 10 0
f1 (ppm)

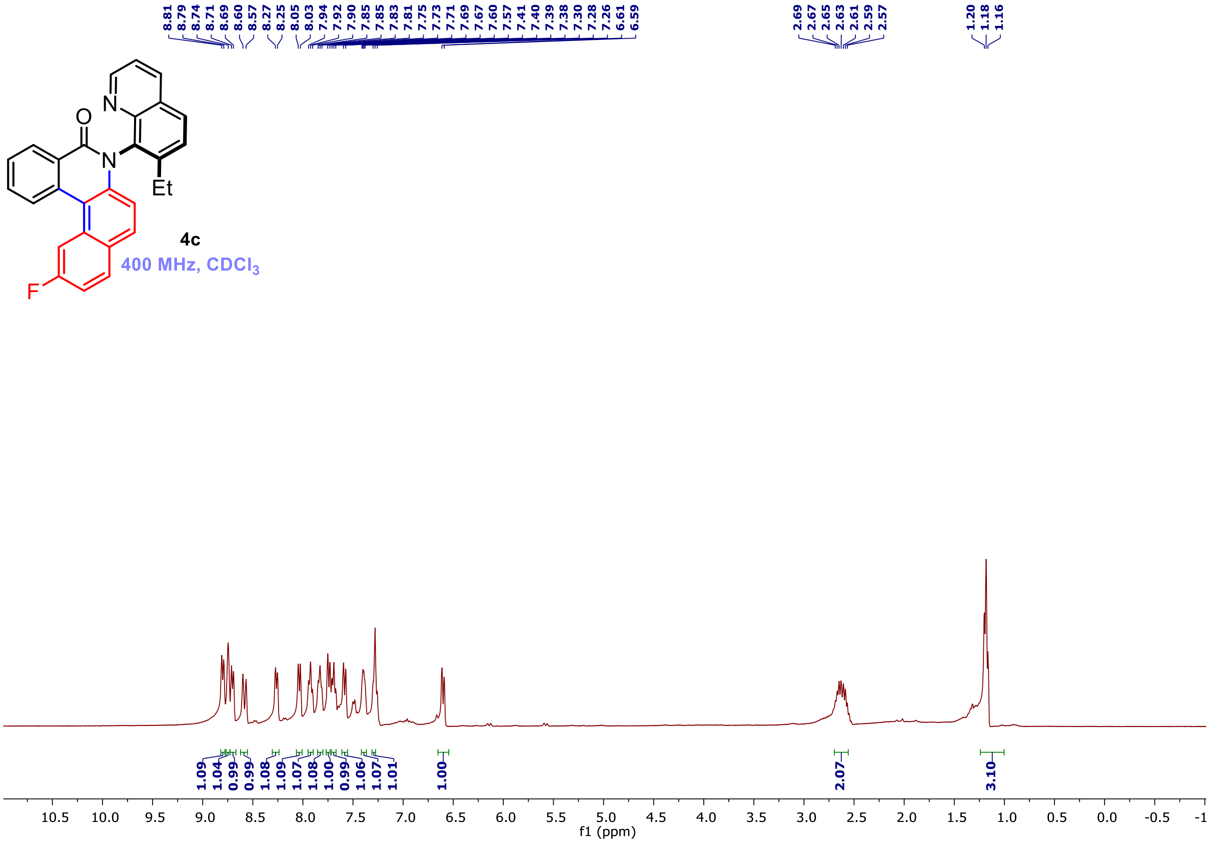
8.81 8.79 8.74 8.71 8.69 8.60 8.57 8.27 8.25 8.05 8.03 7.94 7.92 7.90 7.85 7.83 7.81 7.75 7.73 7.71 7.69 7.67 7.60 7.57 7.41 7.40 7.39 7.38 7.30 7.28 7.26 6.61 6.59
2.69 2.67 2.65 2.63 2.61 2.59 2.57
1.20 1.18 1.16
O
N
N
Et
F
4c
400 MHz, CDCl3
1.09 1.04 0.99 0.99 1.08 1.09 1.07 1.08 1.00 0.99 1.06 1.07 1.01 1.00 2.07 3.10
10.5 10.0 9.5 9.0 8.5 8.0 7.5 7.0 6.5 6.0 5.5 5.0 4.5 4.0 3.5 3.0 2.5 2.0 1.5 1.0 0.5 0.0 -0.5 -1
f1 (ppm)

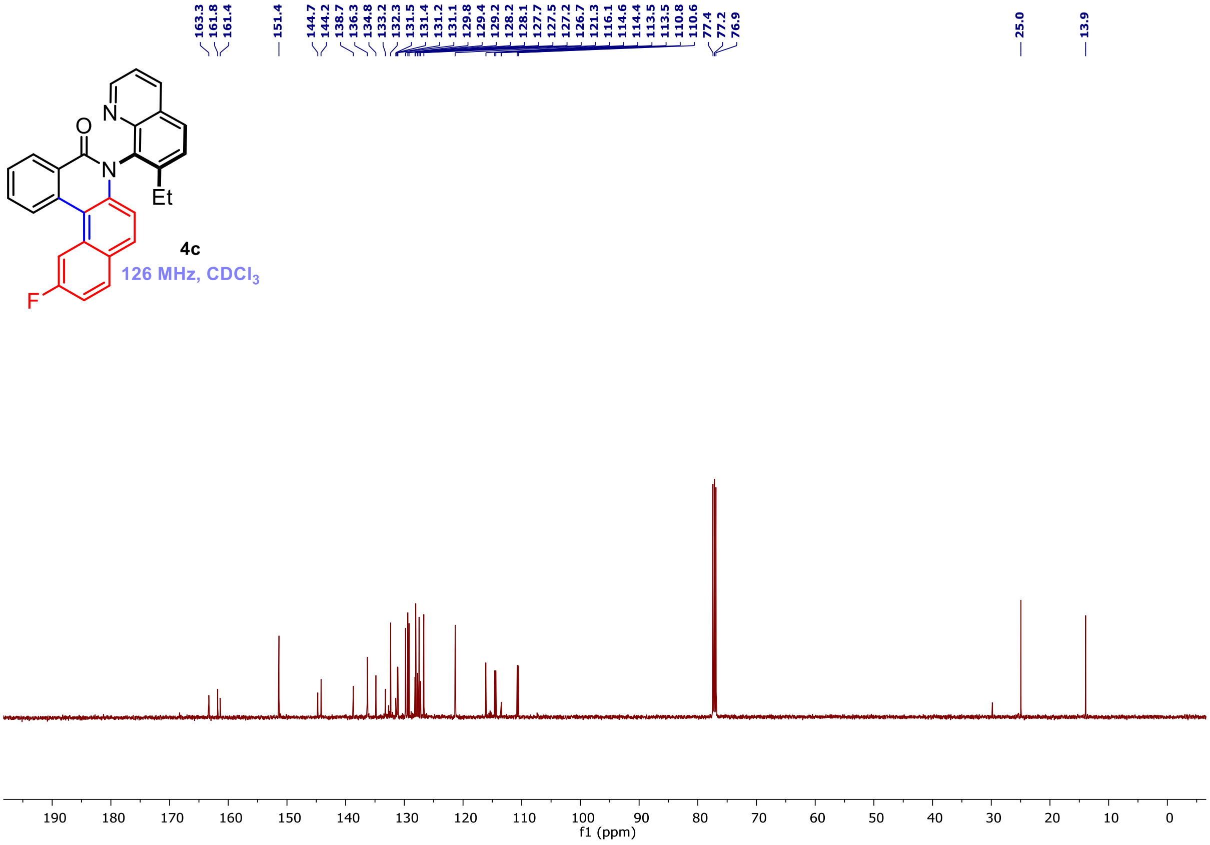
163.3 161.8 161.4 151.4 144.7 144.2 138.7 136.3 134.8 133.2 132.3 131.5 131.4 131.2 131.1 129.8 129.4 129.2 128.2 128.1 127.7 127.5 127.2 126.7 121.3 116.1 114.6 114.4 113.5 110.8 110.6 77.4 77.2 76.9
25.0
13.9
O
N
N
Et
F
4c
126 MHz, CDCl3
190 180 170 160 150 140 130 120 110 100 90 80 70 60 50 40 30 20 10 0
f1 (ppm)

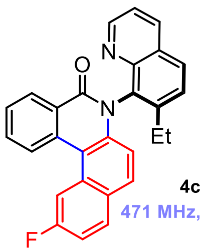

4c

471 MHz, $CDCl_3$

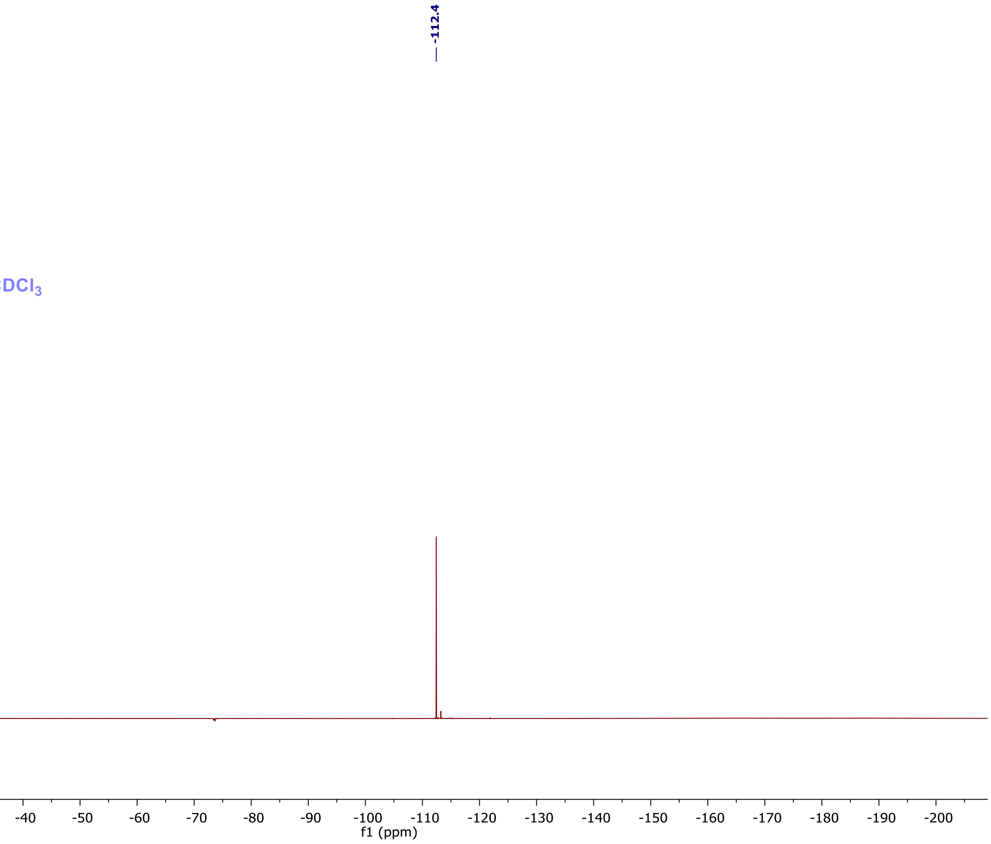

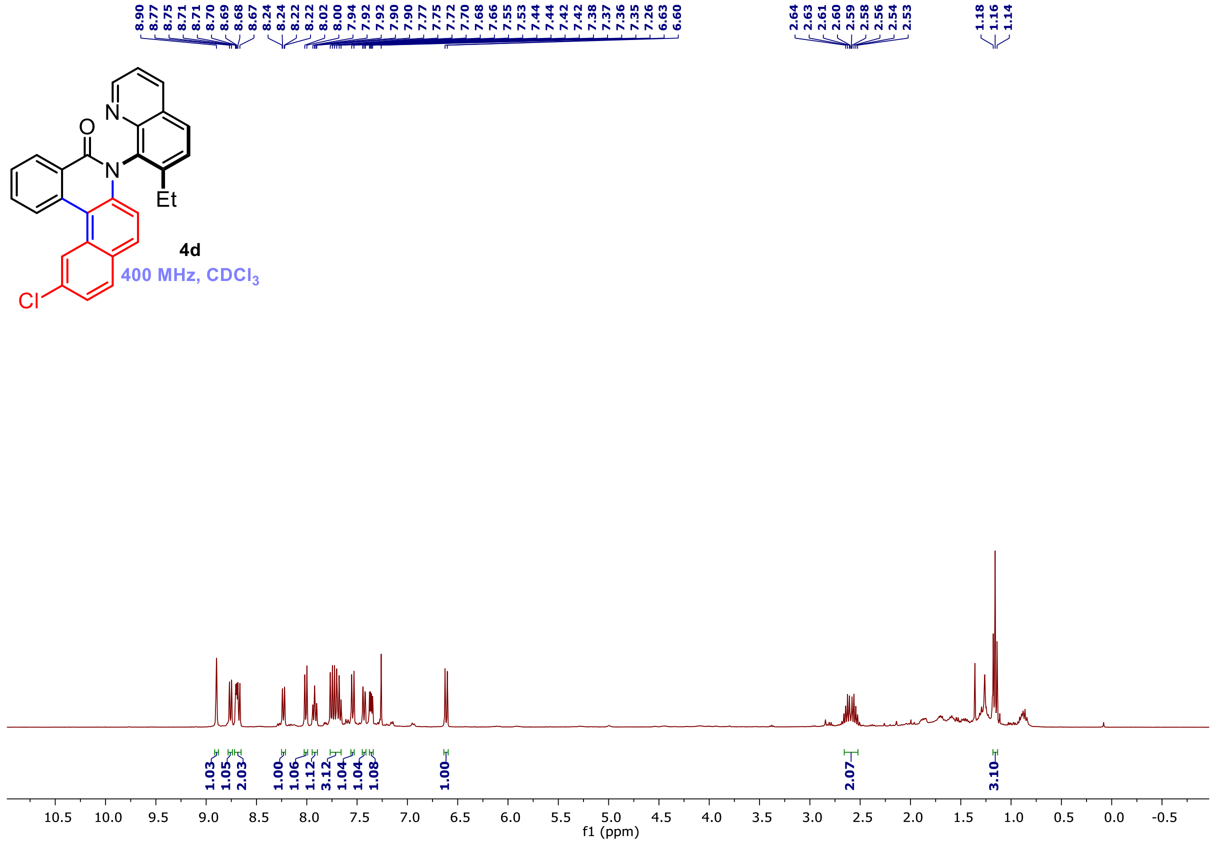
8.90 8.77 8.75 8.71 8.70 8.69 8.68 8.67 8.24 8.24 8.22 8.22 8.02 8.00 7.94 7.92 7.92 7.90 7.90 7.77 7.75 7.72 7.70 7.68 7.66 7.55 7.53 7.44 7.44 7.42 7.42 7.38 7.37 7.36 7.35 7.26 6.63 6.60
2.64 2.63 2.61 2.60 2.59 2.58 2.56 2.54 2.53
1.18 1.16 1.14
O
N
N
Et
Cl
4d
400 MHz, CDCl3
1.03 1.05 2.03 1.00 1.06 1.12 3.12 1.04 1.04 1.08 1.00 2.07 3.10
10.5 10.0 9.5 9.0 8.5 8.0 7.5 7.0 6.5 6.0 5.5 5.0 4.5 4.0 3.5 3.0 2.5 2.0 1.5 1.0 0.5 0.0 -0.5
f1 (ppm)

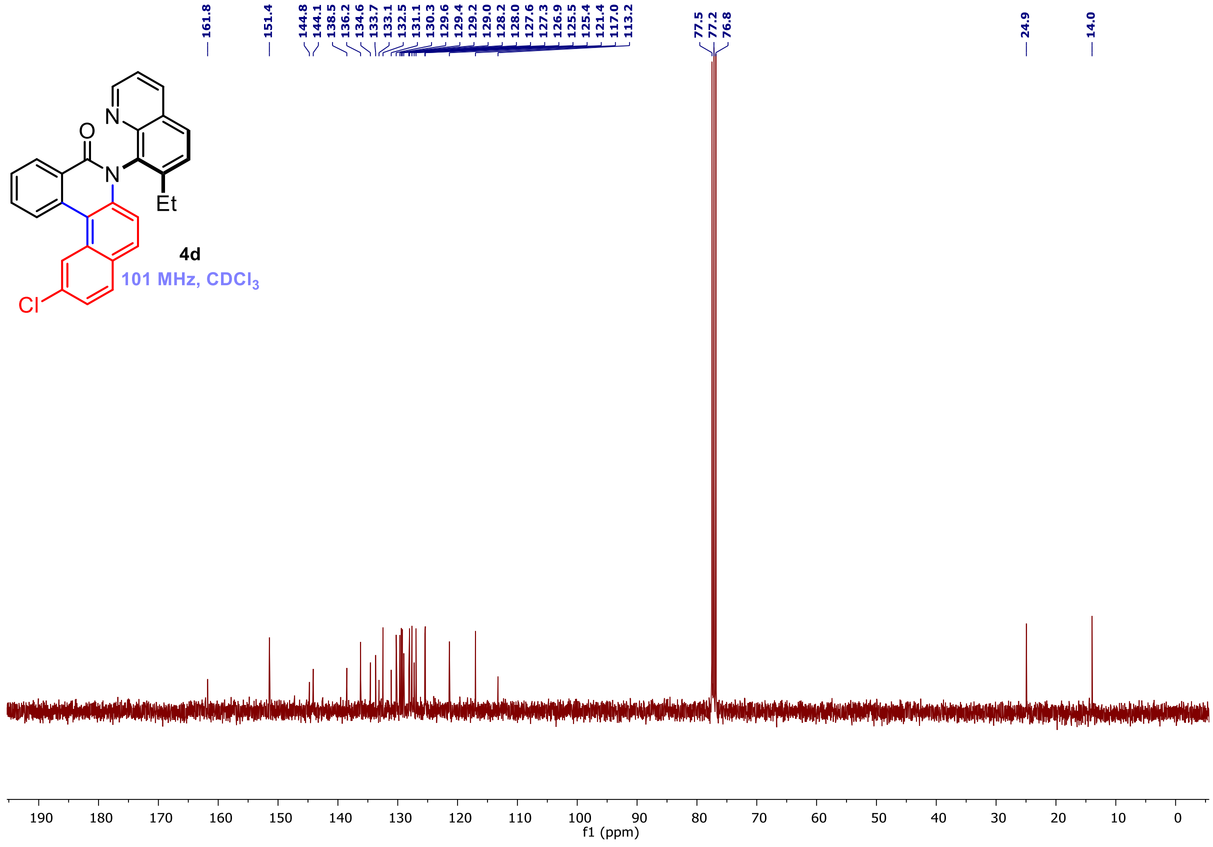
161.8
151.4
144.8 144.1 138.5 136.2 134.6 133.7 133.1 132.5 131.1 130.3 129.6 129.4 129.2 129.0 128.2 128.0 127.6 127.3 126.9 125.5 125.4 121.4 117.0 113.2
77.5 77.2 76.8
24.9
14.0
O
N
N
Et
Cl
4d
101 MHz, CDCl3
190 180 170 160 150 140 130 120 110 100 90 80 70 60 50 40 30 20 10 0
f1 (ppm)

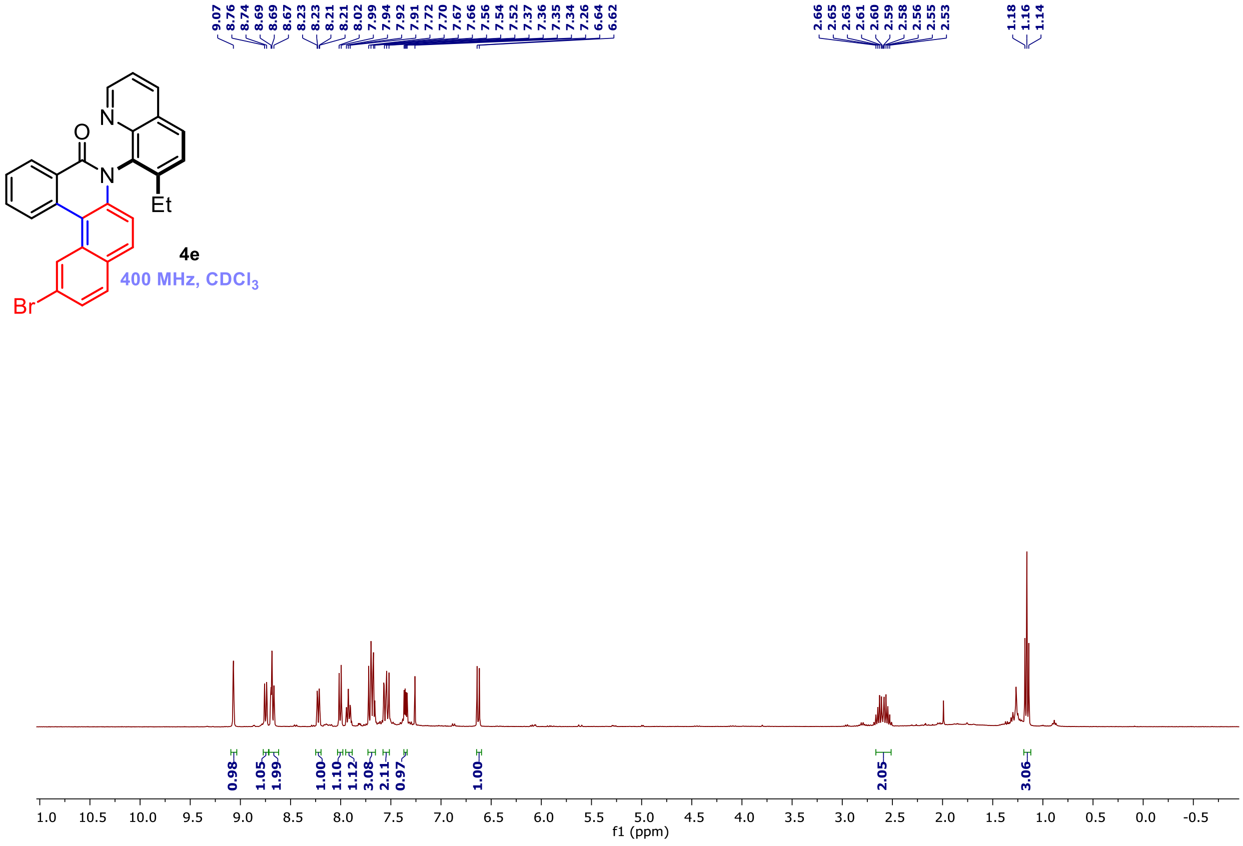
9.07 8.76 8.74 8.69 8.69 8.67 8.23 8.23 8.21 8.21 8.02 7.99 7.94 7.92 7.91 7.72 7.70 7.67 7.66 7.56 7.54 7.52 7.37 7.36 7.35 7.34 7.26 6.64 6.62
2.66 2.65 2.63 2.61 2.60 2.59 2.58 2.56 2.55 2.53
1.18 1.16 1.14
O
N
N
Et
Br
4e
400 MHz, CDCl3
0.98 1.05 1.99 1.00 1.10 1.12 3.08 2.11 0.97 1.00 2.05 3.06
f1 (ppm)

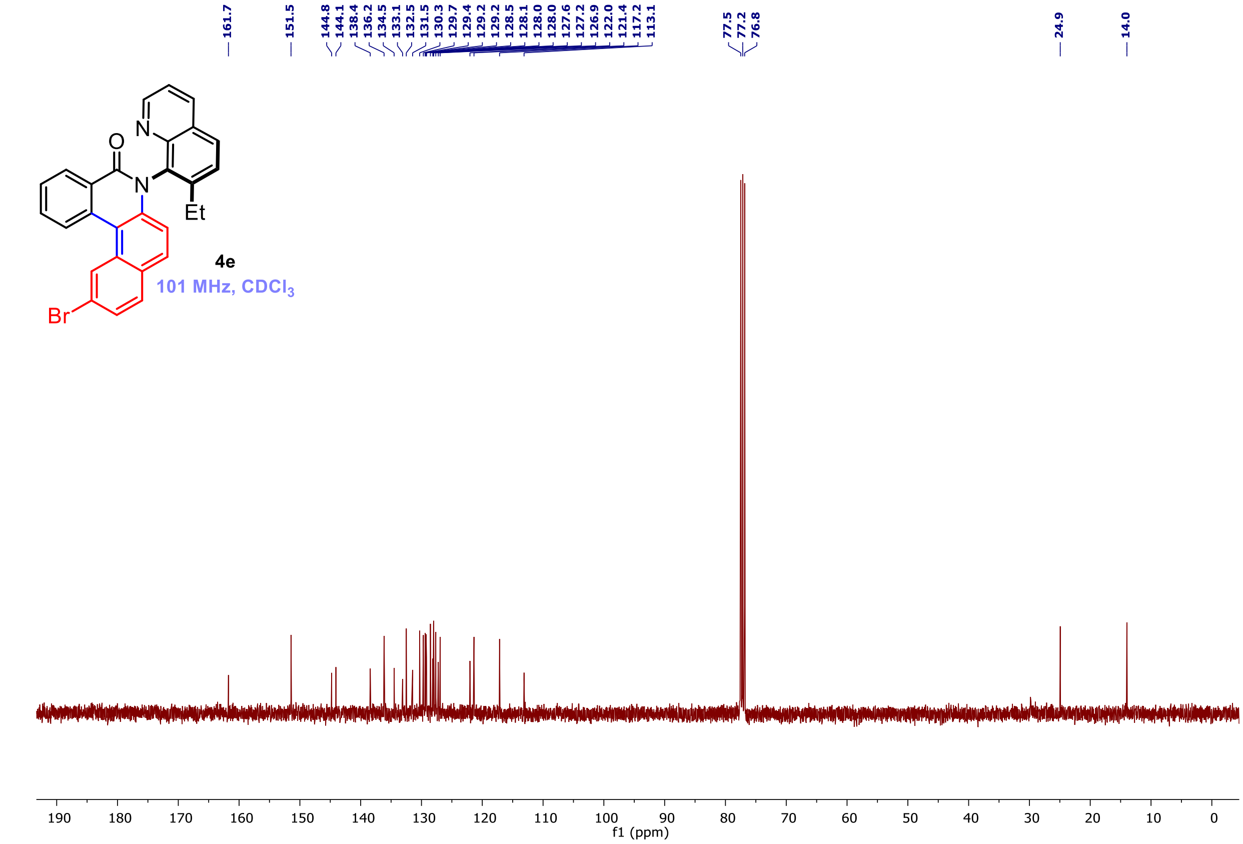
161.7
151.5
144.8 144.1 138.4 136.2 134.5 133.1 132.5 131.5 130.3 129.7 129.4 129.2 129.2 128.5 128.1 128.0 127.6 127.2 126.9 122.0 121.4 117.2 113.1
77.5 77.2 76.8
24.9
14.0
O
N
N
Et
Br
4e
101 MHz, CDCl3
f1 (ppm)

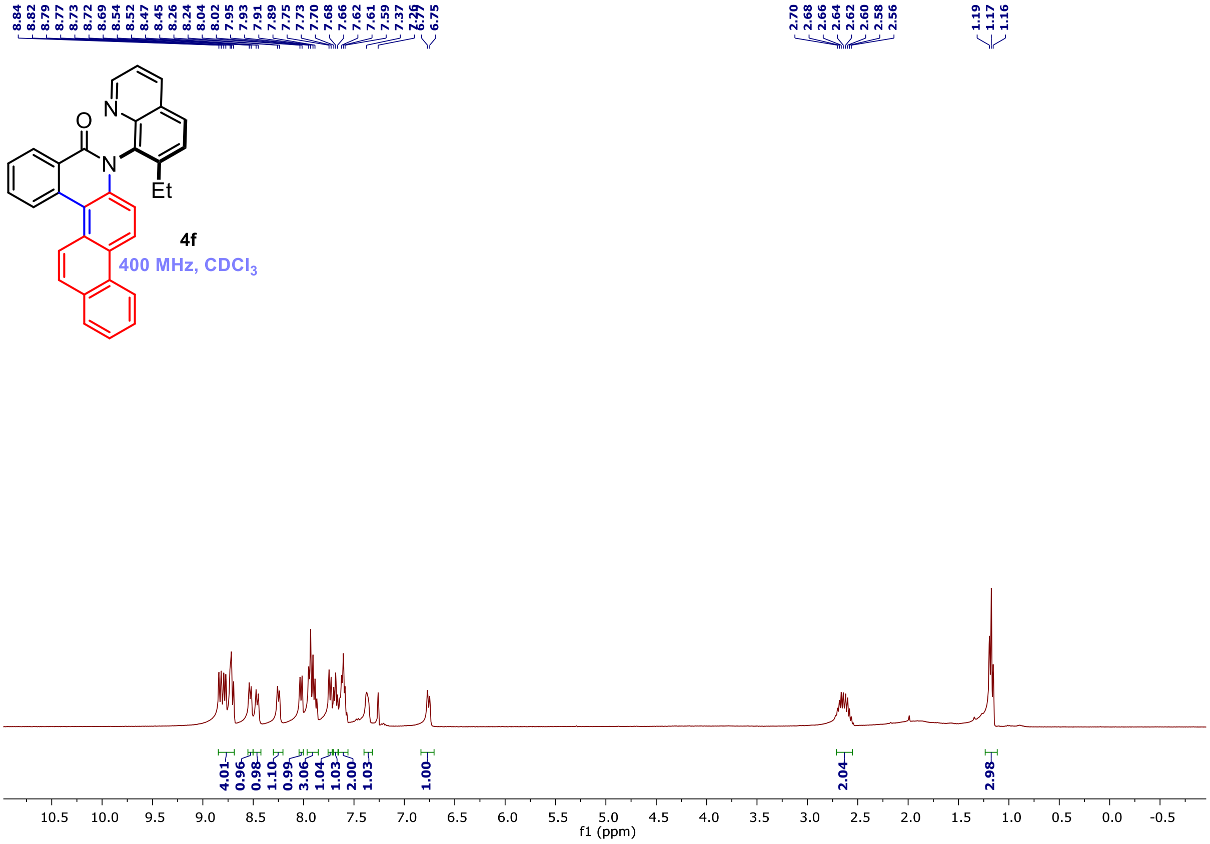
8.84
8.82
8.79
8.77
8.73
8.72
8.69
8.54
8.52
8.47
8.45
8.26
8.24
8.04
8.02
7.95
7.93
7.91
7.89
7.75
7.73
7.70
7.68
7.66
7.62
7.61
7.59
7.37
7.26
6.77
6.75
2.70
2.68
2.66
2.64
2.62
2.60
2.58
2.56
1.19
1.17
1.16
O
N
N
Et
4f
400 MHz, CDCl3
4.01
0.96
0.98
1.10
0.99
3.06
1.04
1.03
2.00
1.03
1.00
2.04
2.98
f1 (ppm)

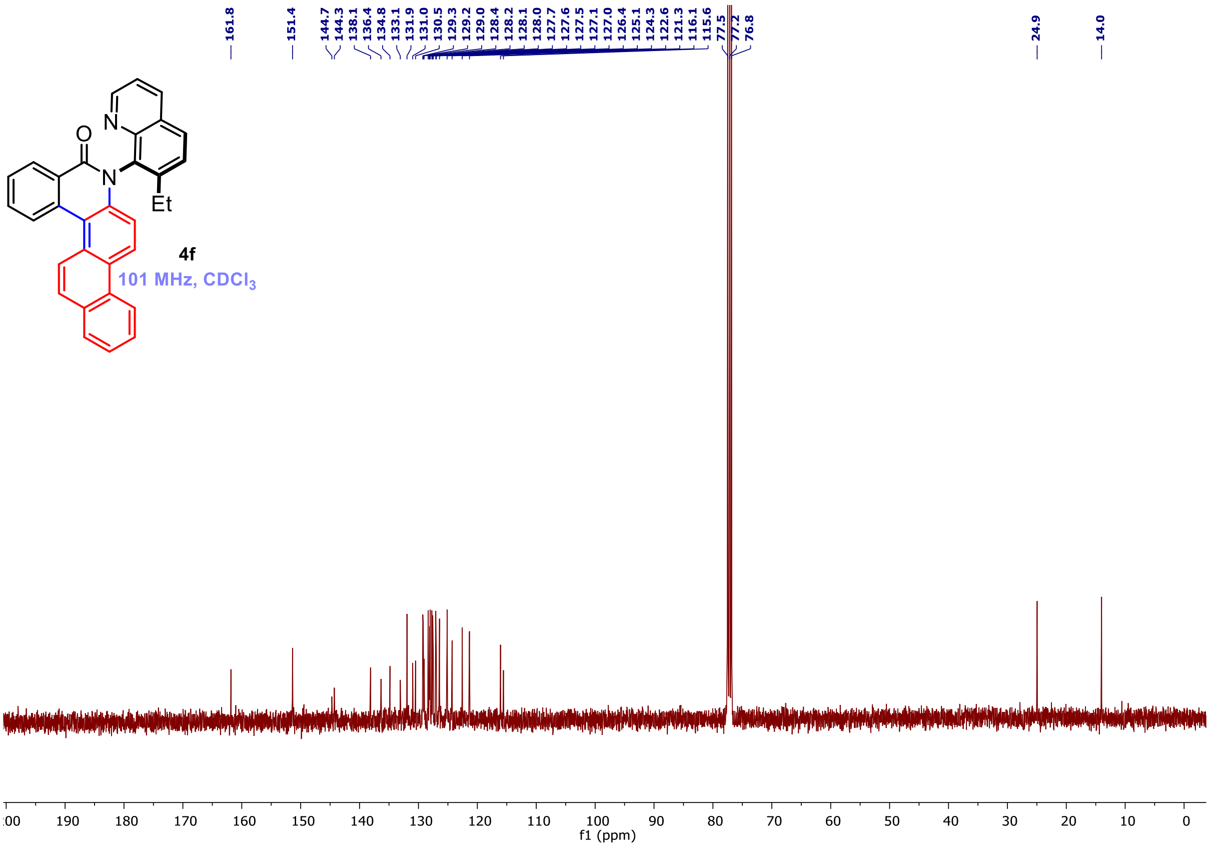
161.8
151.4
144.7
144.3
138.1
136.4
134.8
133.1
131.9
131.0
130.5
129.3
129.2
129.0
128.4
128.2
128.1
128.0
127.7
127.6
127.5
127.1
127.0
126.4
125.1
124.3
122.6
121.3
116.1
115.6
77.5
77.2
76.8
24.9
14.0
O
N
N
Et
4f
101 MHz, CDCl3
f1 (ppm)

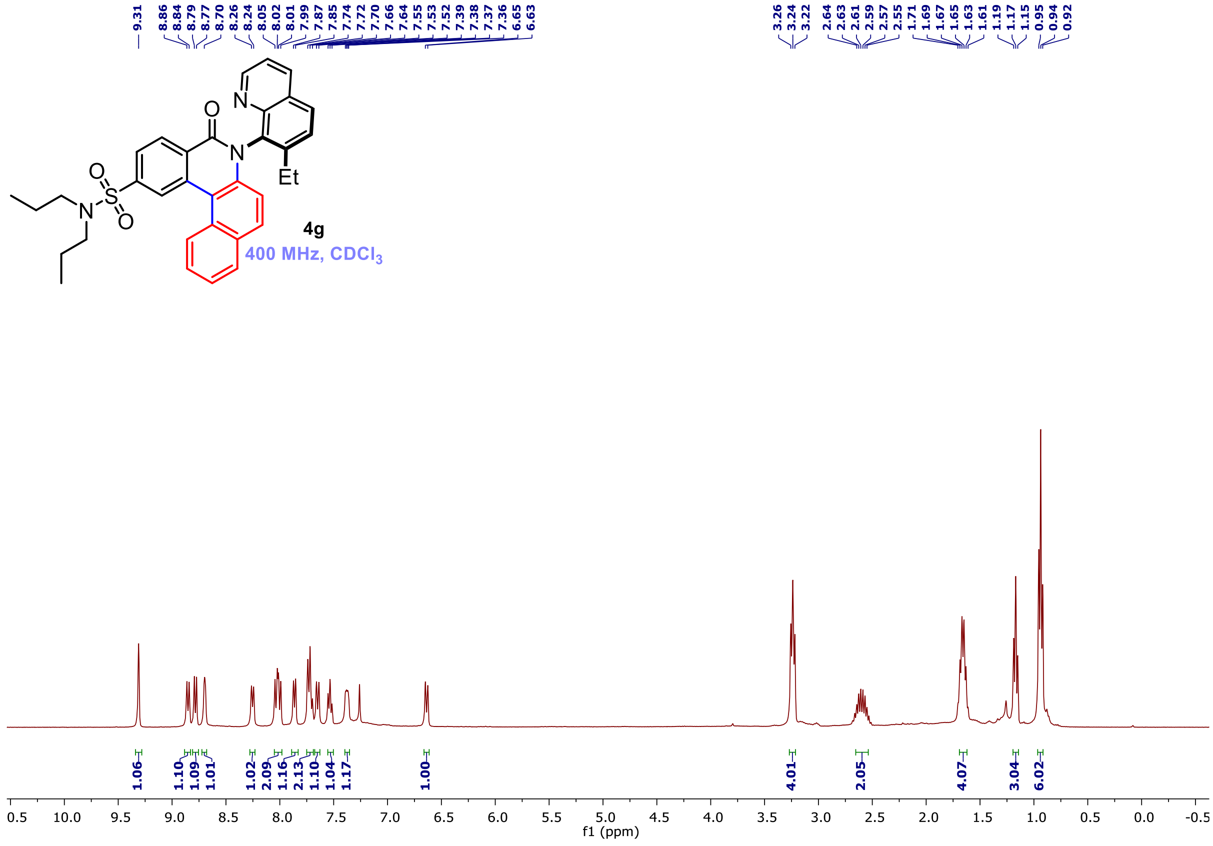
9.31
8.86
8.84
8.79
8.77
8.70
8.26
8.24
8.05
8.02
8.01
7.99
7.87
7.85
7.74
7.72
7.70
7.66
7.64
7.55
7.53
7.52
7.39
7.38
7.37
7.36
6.65
6.63
3.26
3.24
3.22
2.64
2.63
2.61
2.59
2.57
2.55
1.71
1.69
1.67
1.65
1.63
1.61
1.19
1.17
1.15
0.95
0.94
0.92
N
O
N
Et
O
S
O
N
4g
400 MHz, CDCl3
1.06
1.10
1.09
1.01
1.02
2.09
1.16
2.13
1.10
1.04
1.17
1.00
4.01
2.05
4.07
3.04
6.02
f1 (ppm)

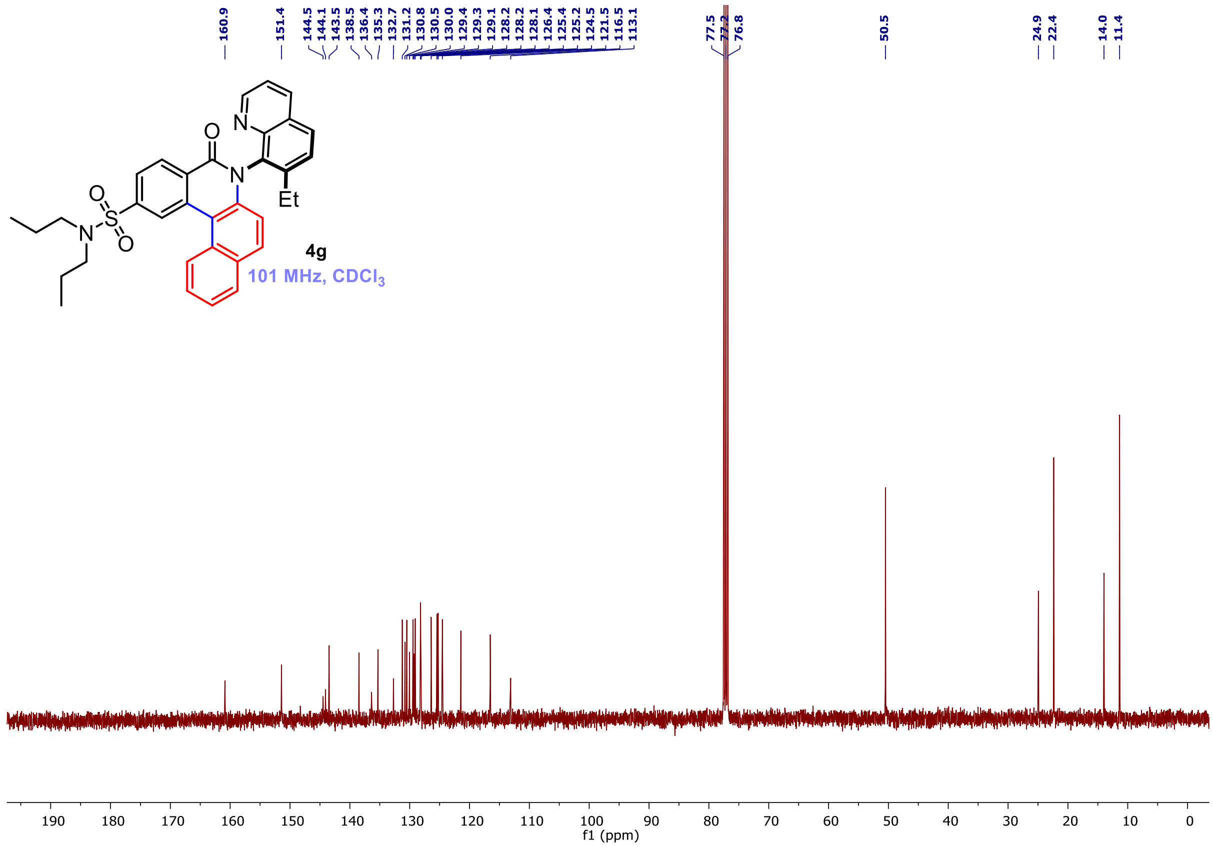
160.9
151.4
144.5
144.1
143.5
138.5
136.4
135.3
132.7
131.2
130.8
130.5
130.0
129.4
129.3
129.1
128.2
128.1
126.4
125.4
125.2
124.5
121.5
116.5
113.1
77.5
77.2
76.8
50.5
24.9
22.4
14.0
11.4
N
O
N
Et
O
S
O
N
4g
101 MHz, CDCl3
f1 (ppm)

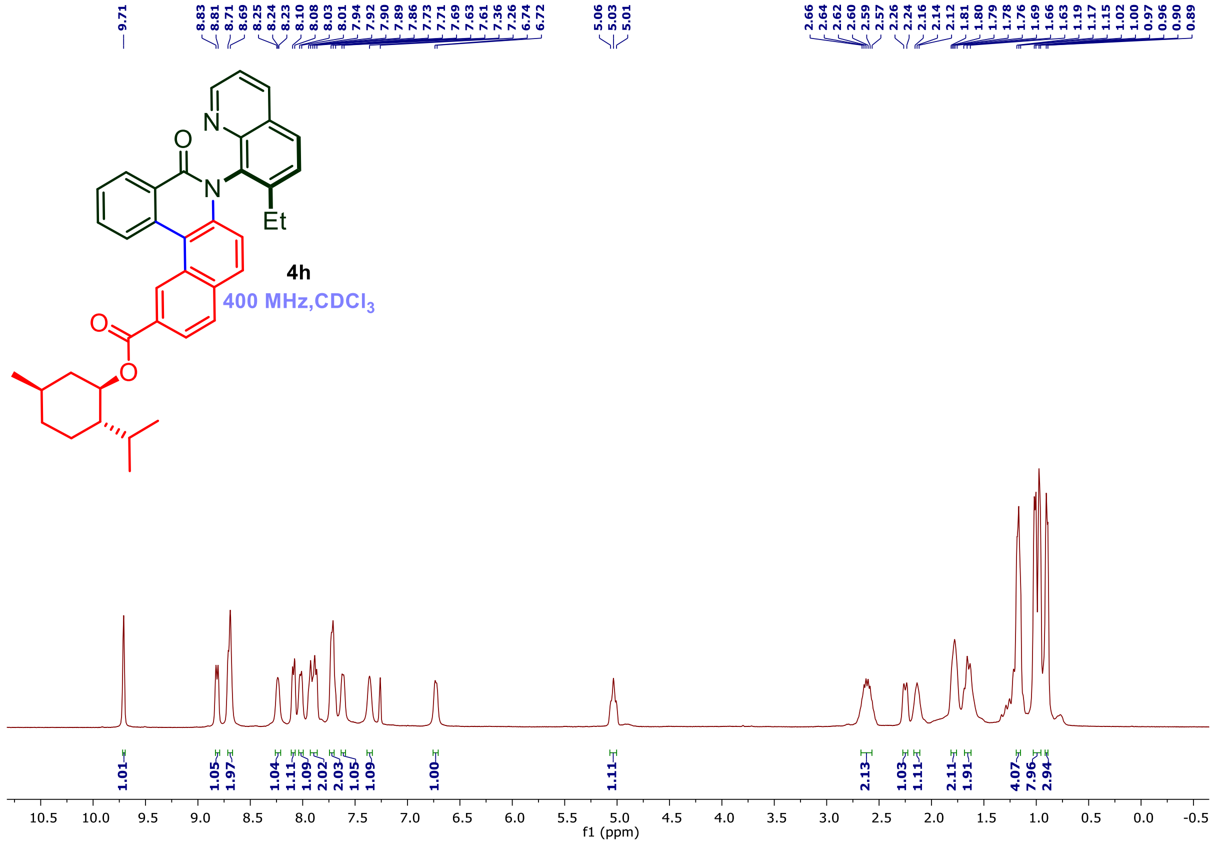
4h
400 MHz,CDCl3
f1 (ppm)

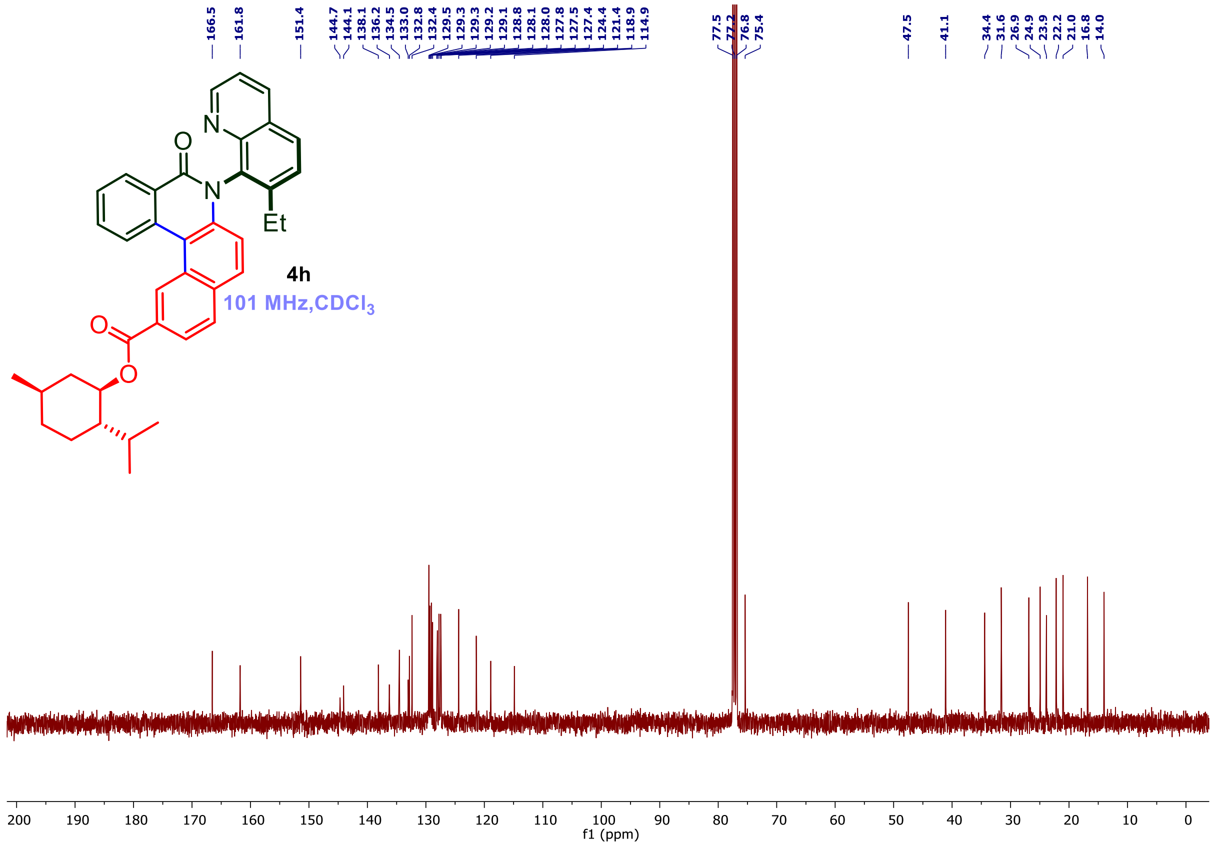
4h
101 MHz,CDCl3
f1 (ppm)

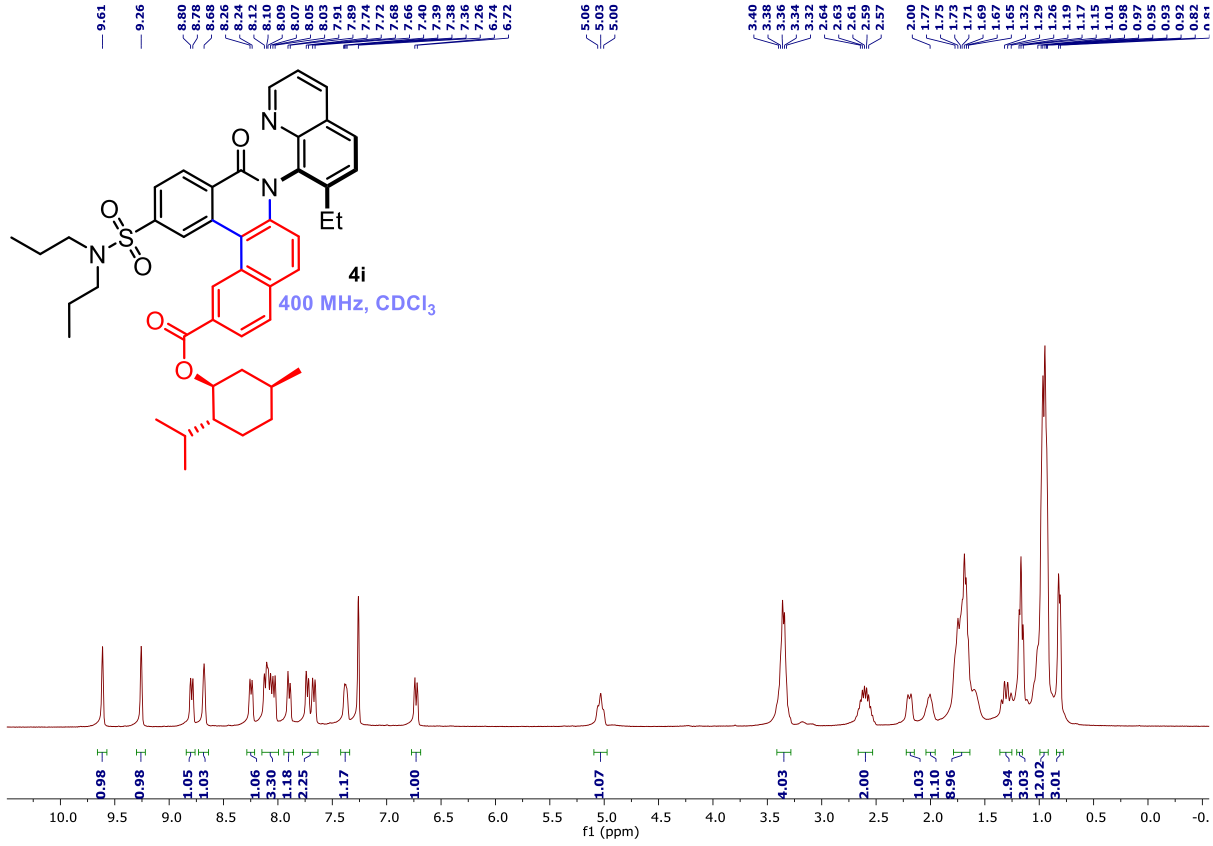
9.61
9.26
8.80
8.78
8.68
8.26
8.24
8.12
8.10
8.09
8.07
8.05
8.03
7.91
7.89
7.74
7.72
7.68
7.66
7.40
7.39
7.38
7.36
7.26
6.74
6.72
5.06
5.03
5.00
3.40
3.38
3.36
3.34
3.32
2.64
2.63
2.61
2.59
2.57
2.00
1.77
1.75
1.73
1.71
1.69
1.67
1.65
1.32
1.29
1.26
1.19
1.17
1.15
1.01
0.98
0.97
0.95
0.93
0.92
0.82
N
O
N
Et
O
S
N
O
4i
400 MHz, CDCl3
O
O
0.98
0.98
1.05
1.03
1.06
3.30
1.18
2.25
1.17
1.00
1.07
4.03
2.00
1.03
1.10
8.96
1.94
3.03
12.02
3.01
10.0
9.5
9.0
8.5
8.0
7.5
7.0
6.5
6.0
5.5
5.0
4.5
4.0
3.5
3.0
2.5
2.0
1.5
1.0
0.5
0.0
f1 (ppm)

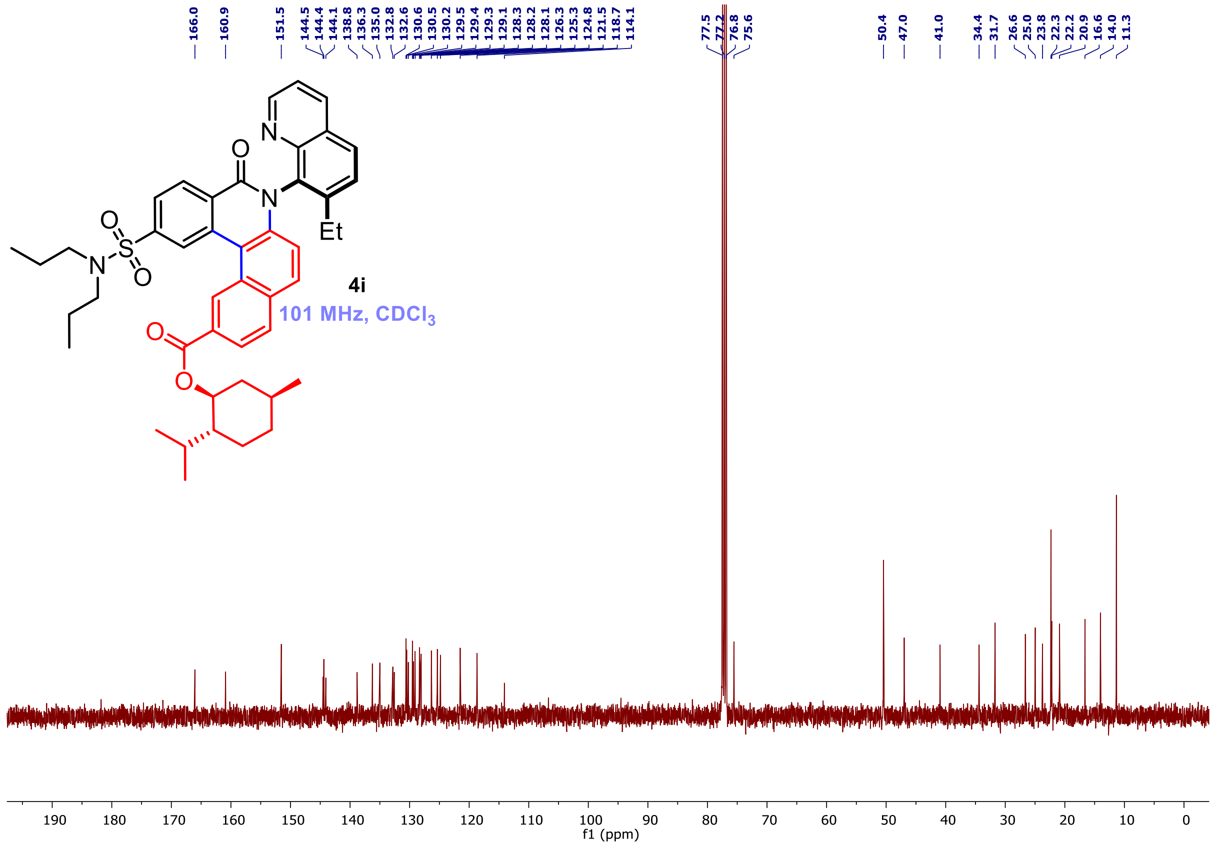
166.0
160.9
151.5
144.5
144.4
144.1
138.8
136.3
135.0
132.8
132.6
130.6
130.5
130.2
129.5
129.4
129.3
129.1
128.3
128.2
128.1
126.3
125.3
124.8
121.5
118.7
114.1
77.5
77.2
76.8
75.6
50.4
47.0
41.0
34.4
31.7
26.6
25.0
23.8
22.3
22.2
20.9
16.6
14.0
11.3
N
O
N
Et
O
S
N
O
4i
101 MHz, CDCl3
O
O
190
180
170
160
150
140
130
120
110
100
90
80
70
60
50
40
30
20
10
0
f1 (ppm)

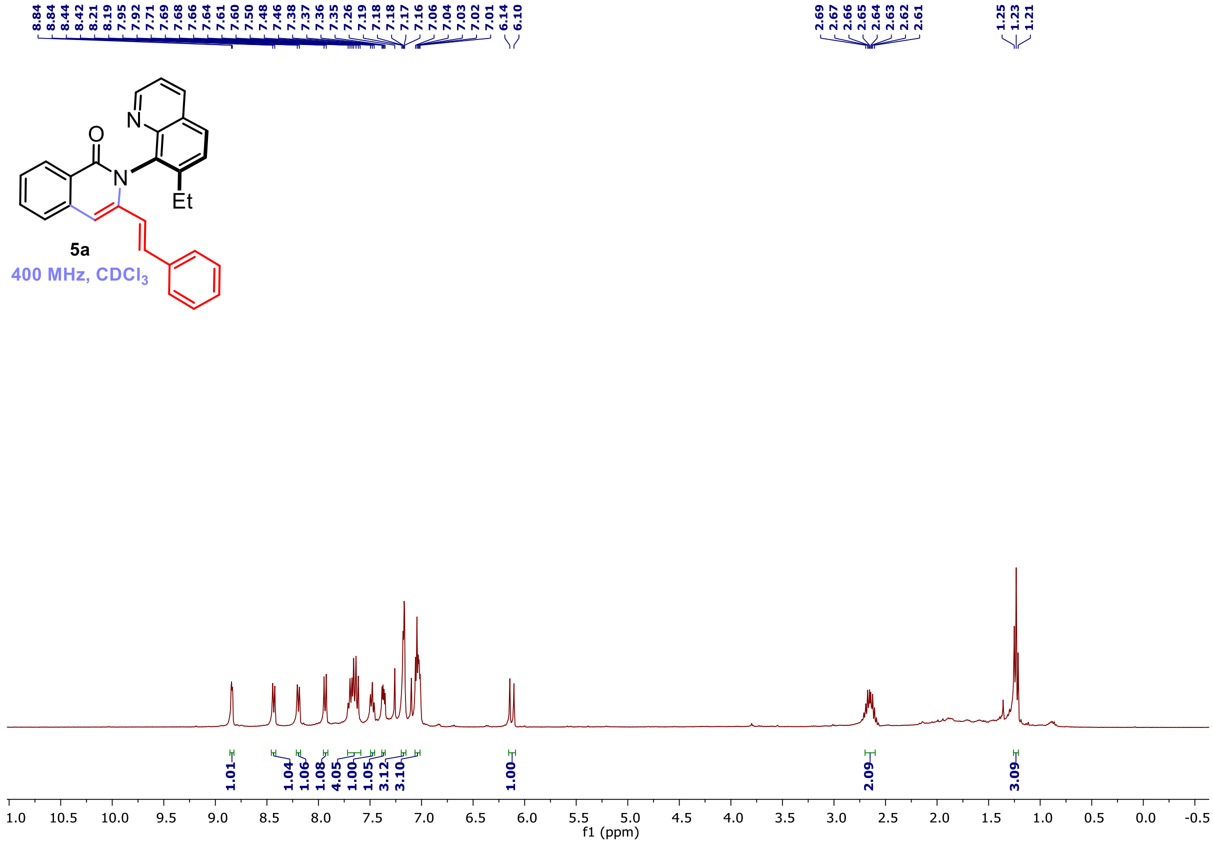
5a
400 MHz, CDCl3
f1 (ppm)

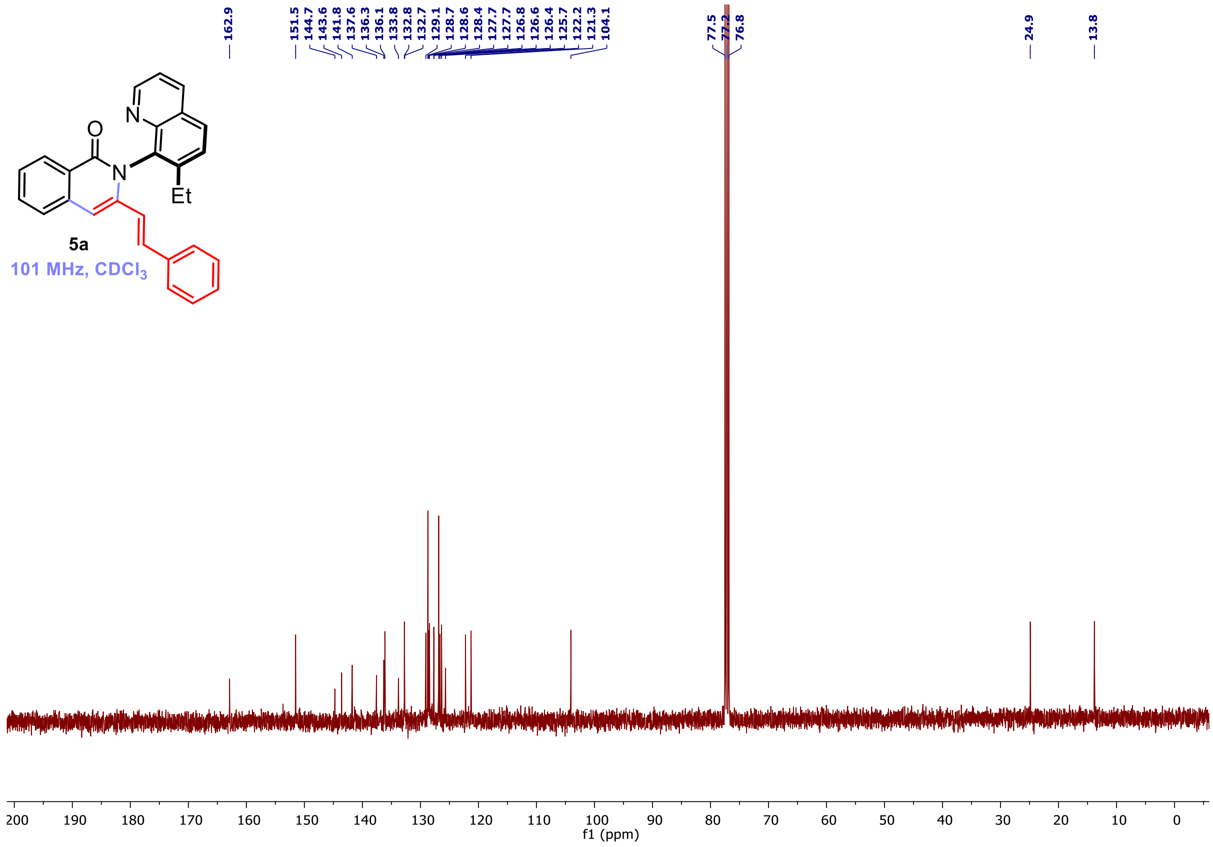
5a
101 MHz, CDCl3
f1 (ppm)

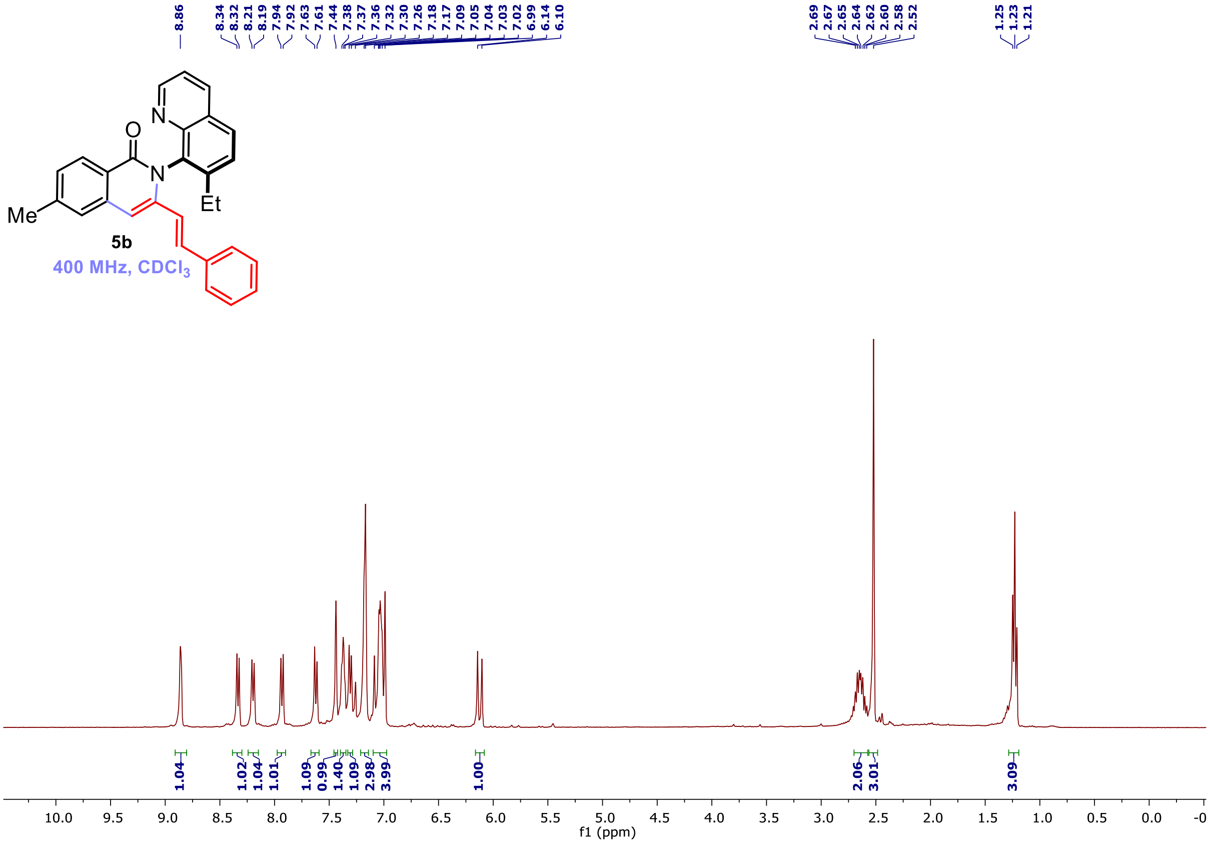
8.86
8.34
8.32
8.21
8.19
7.94
7.92
7.63
7.61
7.44
7.38
7.37
7.36
7.32
7.30
7.26
7.18
7.17
7.09
7.05
7.04
7.03
7.02
6.99
6.14
6.10
2.69
2.67
2.65
2.64
2.62
2.60
2.58
2.52
1.25
1.23
1.21
Me
O
N
N
Et
5b
400 MHz, CDCl3
1.04
1.02
1.04
1.01
1.09
0.99
1.40
1.09
2.98
3.99
1.00
2.06
3.01
3.09
f1 (ppm)

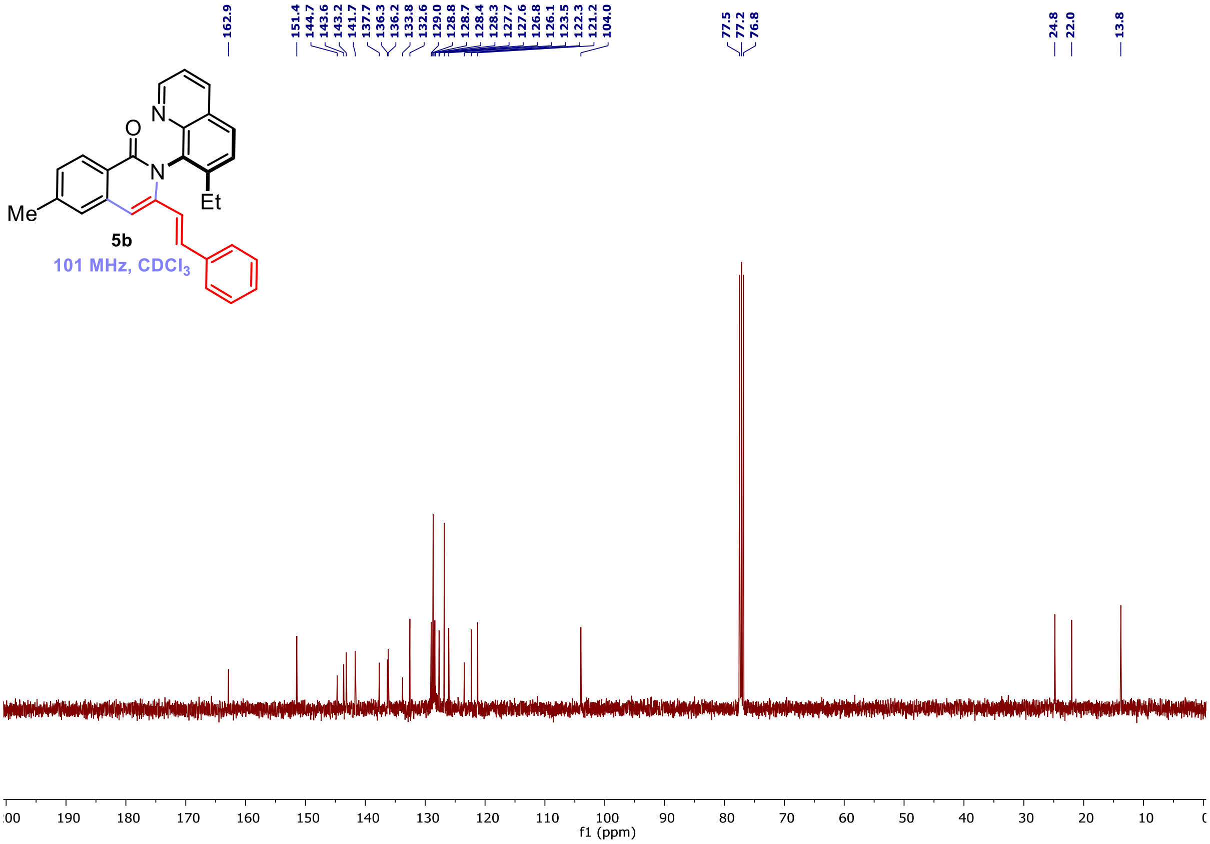
162.9
151.4
144.7
143.6
143.2
141.7
137.7
136.3
136.2
133.8
132.6
129.0
128.8
128.7
128.4
128.3
127.7
127.6
126.8
126.1
123.5
122.3
121.2
104.0
77.5
77.2
76.8
24.8
22.0
13.8
Me
O
N
N
Et
5b
101 MHz, CDCl3
f1 (ppm)

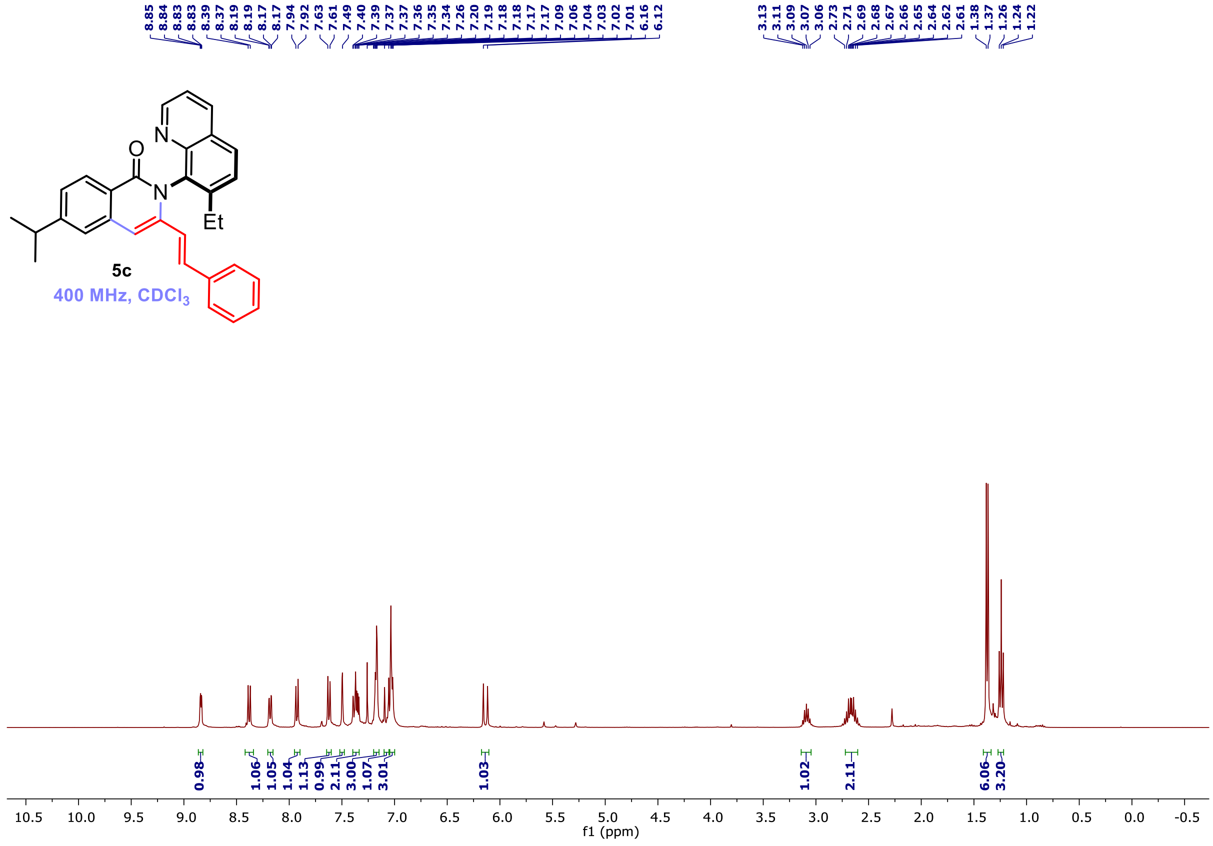
8.85 8.84 8.83 8.83 8.39 8.37 8.19 8.19 8.17 8.17 7.94 7.92 7.63 7.61 7.49 7.40 7.39 7.37 7.37 7.36 7.35 7.34 7.26 7.20 7.19 7.18 7.18 7.17 7.09 7.06 7.04 7.03 7.02 7.01 6.16 6.12
3.13 3.11 3.09 3.07 3.06 2.73 2.71 2.69 2.68 2.67 2.66 2.65 2.64 2.62 2.61 1.38 1.37 1.26 1.24 1.22
O
N
N
Et
5c
400 MHz, $CDCl_3$
0.98 1.06 1.05 1.04 1.13 0.99 2.11 3.00 1.07 3.01 1.03 1.02 2.11 6.06 3.20
10.5 10.0 9.5 9.0 8.5 8.0 7.5 7.0 6.5 6.0 5.5 5.0 4.5 4.0 3.5 3.0 2.5 2.0 1.5 1.0 0.5 0.0 -0.5
f1 (ppm)

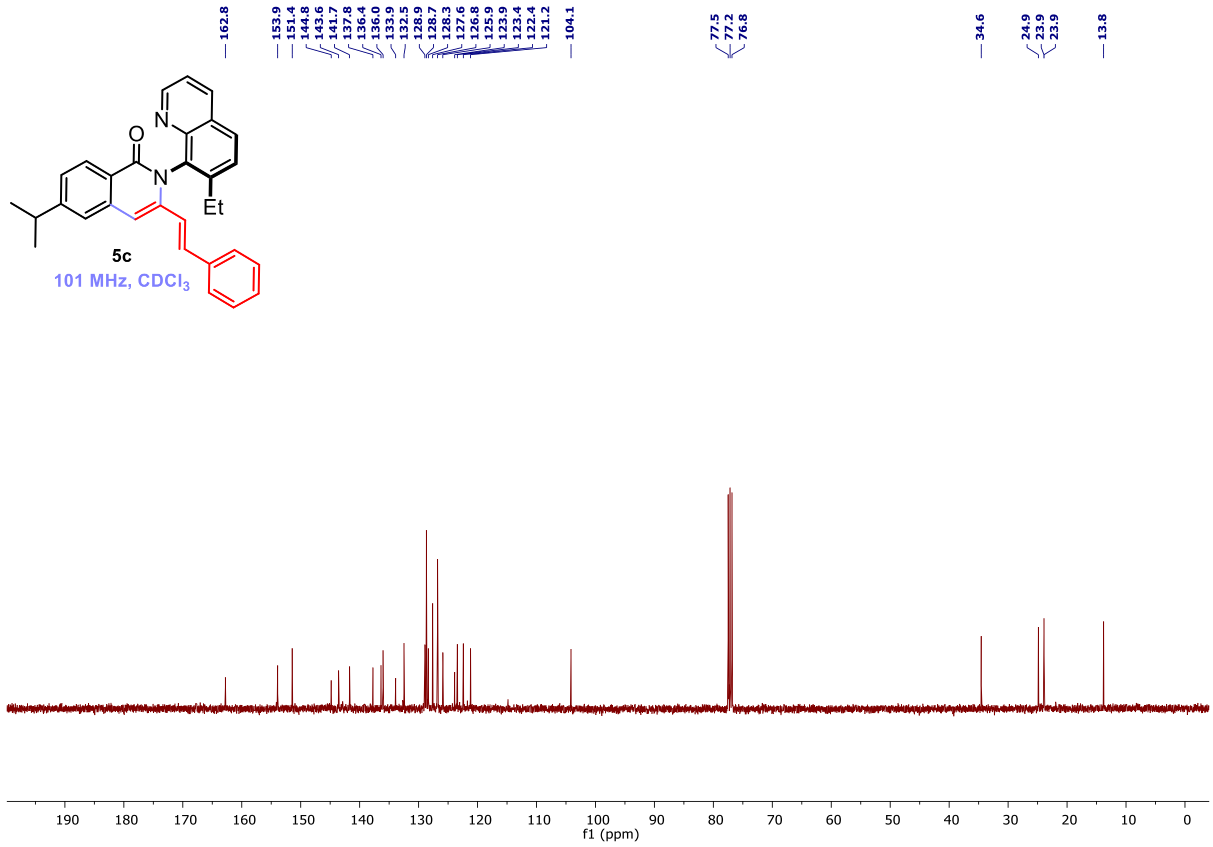
162.8 153.9 151.4 144.8 143.6 141.7 137.8 136.4 136.0 133.9 132.5 128.9 128.7 128.3 127.6 126.8 125.9 123.9 123.4 122.4 121.2 104.1
77.5 77.2 76.8
34.6 24.9 23.9 23.9 13.8
O
N
N
Et
5c
101 MHz, $CDCl_3$
190 180 170 160 150 140 130 120 110 100 90 80 70 60 50 40 30 20 10 0
f1 (ppm)

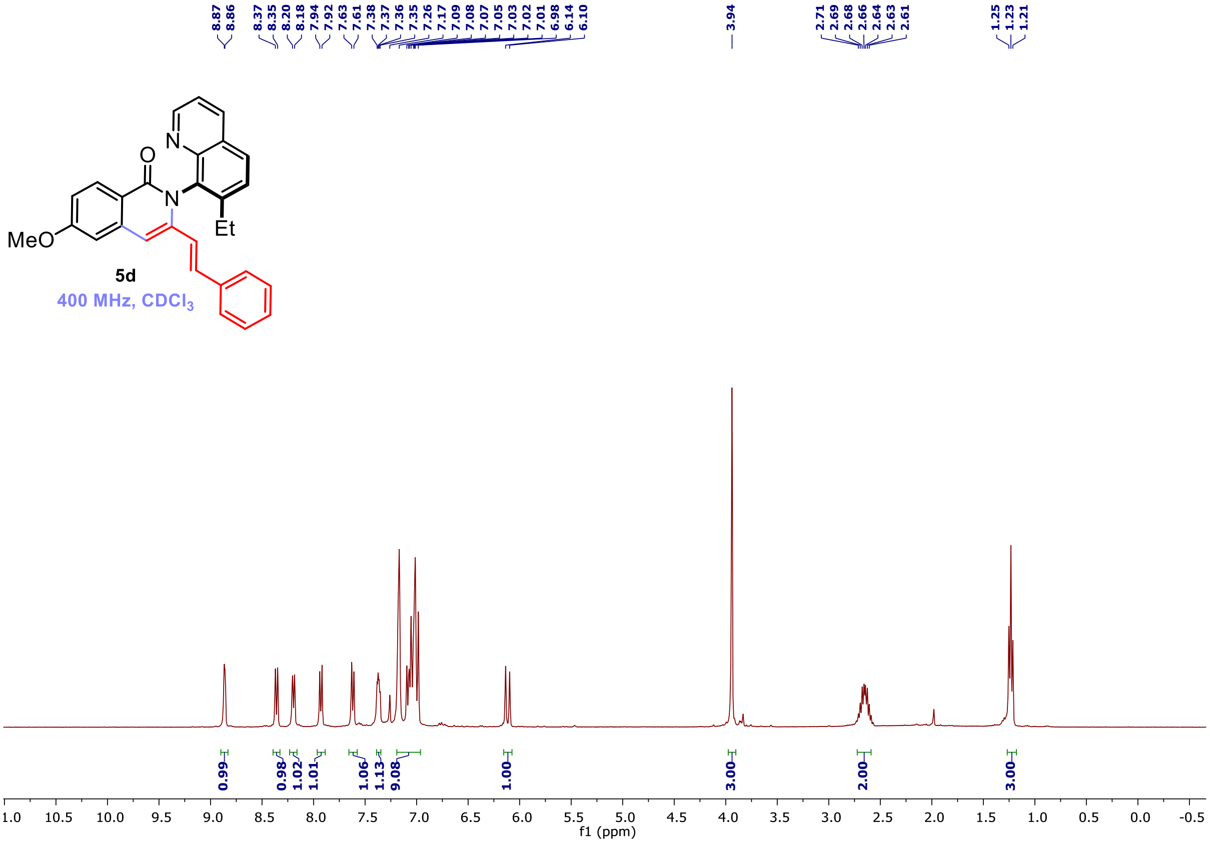
8.87 8.86 8.37 8.35 8.20 8.18 7.94 7.92 7.63 7.61 7.38 7.37 7.36 7.35 7.26 7.17 7.09 7.08 7.07 7.05 7.03 7.02 7.01 6.98 6.14 6.10
3.94
2.71 2.69 2.68 2.66 2.64 2.63 2.61
1.25 1.23 1.21
O
N
N
Et
MeO
5d
400 MHz, CDCl3
0.99 0.98 1.02 1.01 1.06 1.13 9.08 1.00 3.00 2.00 3.00
1.0 10.5 10.0 9.5 9.0 8.5 8.0 7.5 7.0 6.5 6.0 5.5 5.0 4.5 4.0 3.5 3.0 2.5 2.0 1.5 1.0 0.5 0.0 -0.5
f1 (ppm)

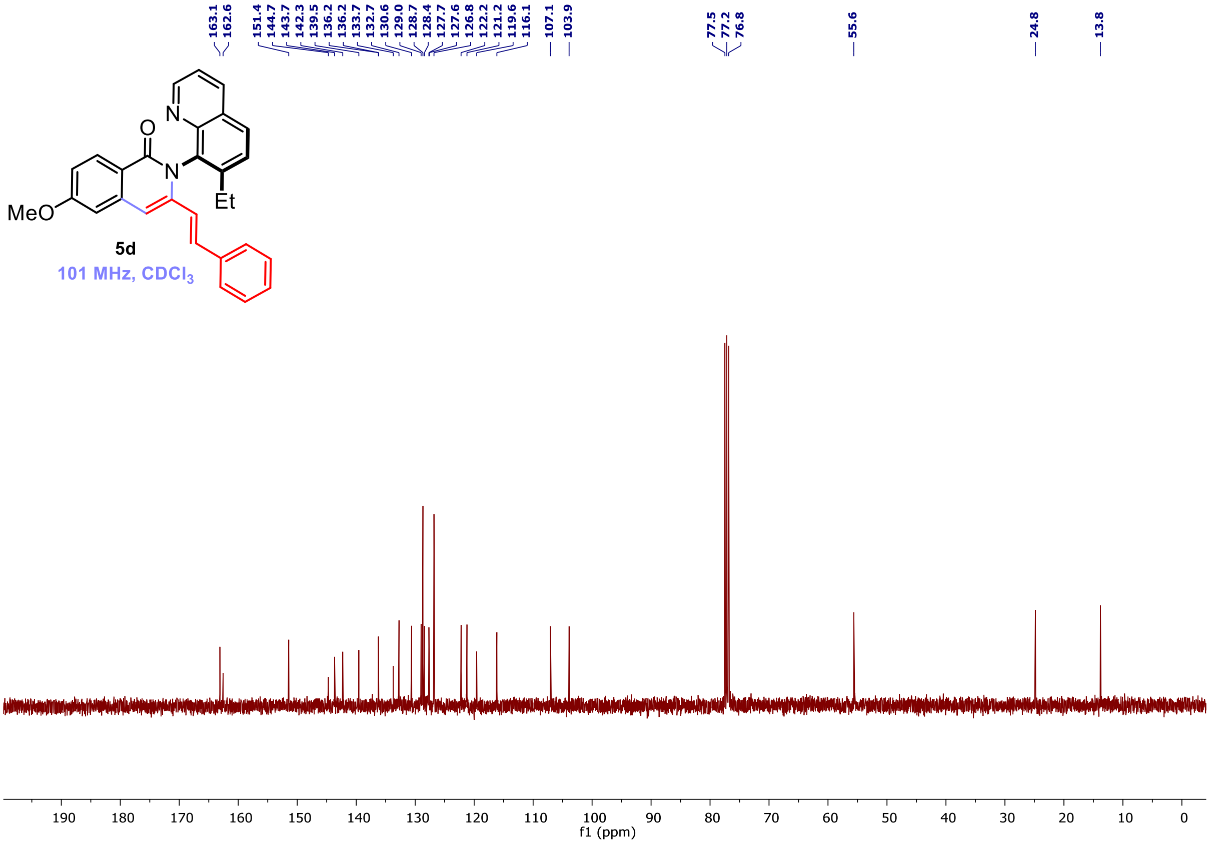
163.1 162.6 151.4 144.7 143.7 142.3 139.5 136.2 136.2 133.7 132.7 130.6 129.0 128.7 128.4 127.7 127.6 126.8 122.2 121.2 119.6 116.1 107.1 103.9
77.5 77.2 76.8
55.6
24.8
13.8
O
N
N
Et
MeO
5d
101 MHz, CDCl3
190 180 170 160 150 140 130 120 110 100 90 80 70 60 50 40 30 20 10 0
f1 (ppm)

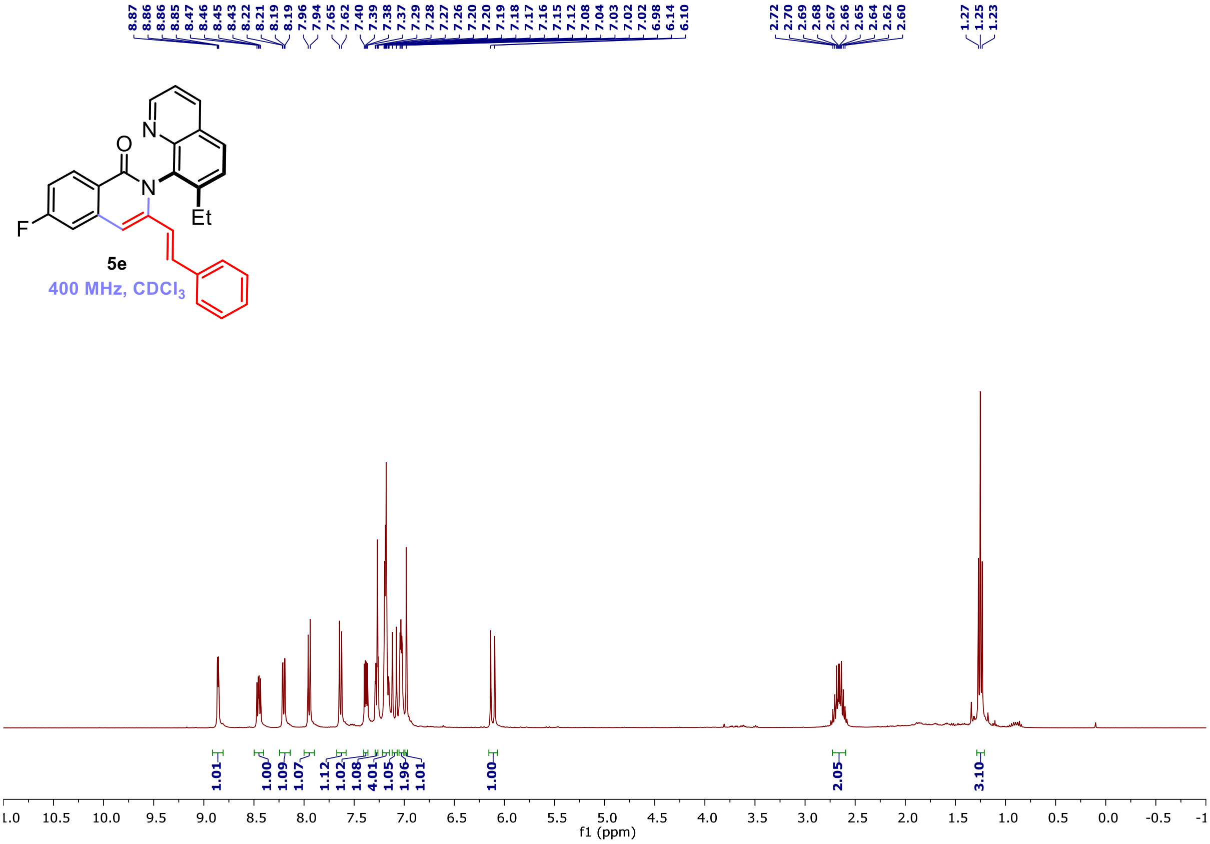
8.87
8.86
8.86
8.85
8.47
8.46
8.45
8.43
8.22
8.21
8.19
8.19
7.96
7.94
7.65
7.62
7.40
7.39
7.38
7.37
7.29
7.28
7.27
7.26
7.20
7.20
7.19
7.18
7.17
7.16
7.15
7.12
7.08
7.04
7.03
7.02
7.02
6.98
6.14
6.10
2.72
2.70
2.69
2.68
2.67
2.66
2.65
2.64
2.62
2.60
1.27
1.25
1.23
N
O
N
F
Et
5e
400 MHz, CDCl3
1.01
1.00
1.09
1.07
1.12
1.02
1.08
4.01
1.05
1.96
1.01
1.00
2.05
3.10
f1 (ppm)

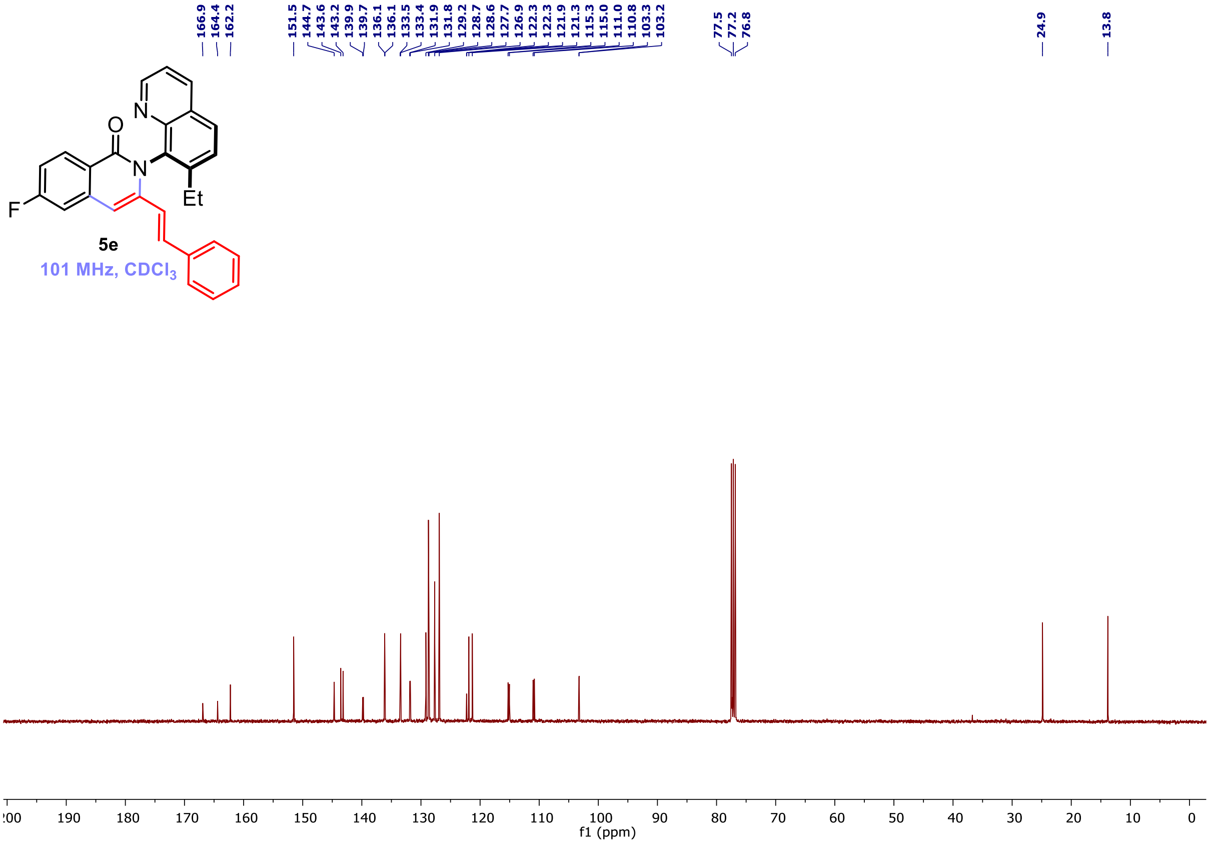
166.9
164.4
162.2
151.5
144.7
143.6
143.2
139.9
139.7
136.1
136.1
133.5
133.4
131.9
131.8
129.2
128.7
128.6
127.7
126.9
122.3
122.3
121.9
121.3
115.3
115.0
111.0
110.8
103.3
103.2
77.5
77.2
76.8
24.9
13.8
N
O
N
F
Et
5e
101 MHz, CDCl3
f1 (ppm)

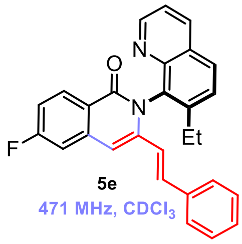

5e

471 MHz, $CDCl_3$

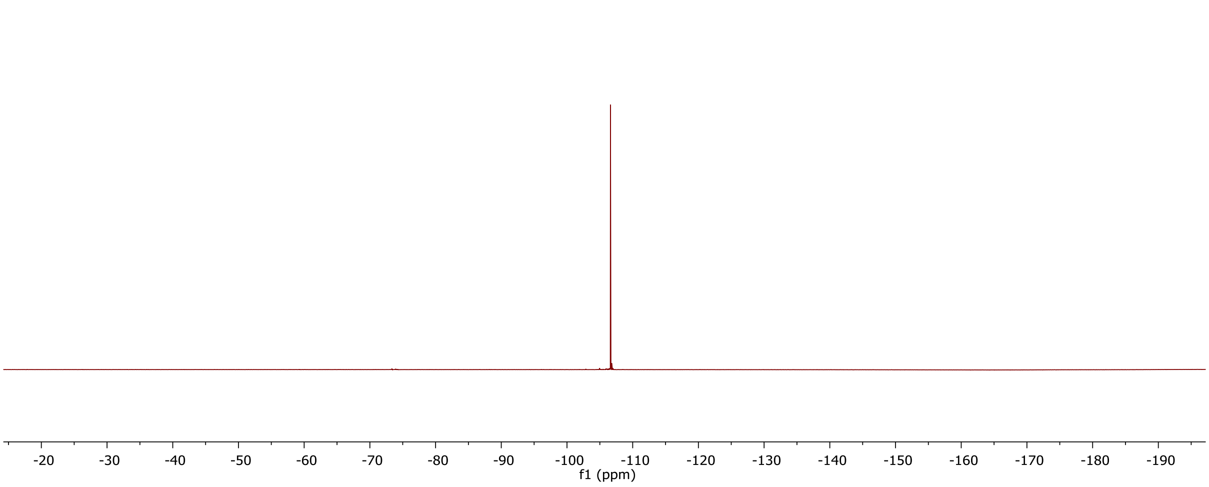

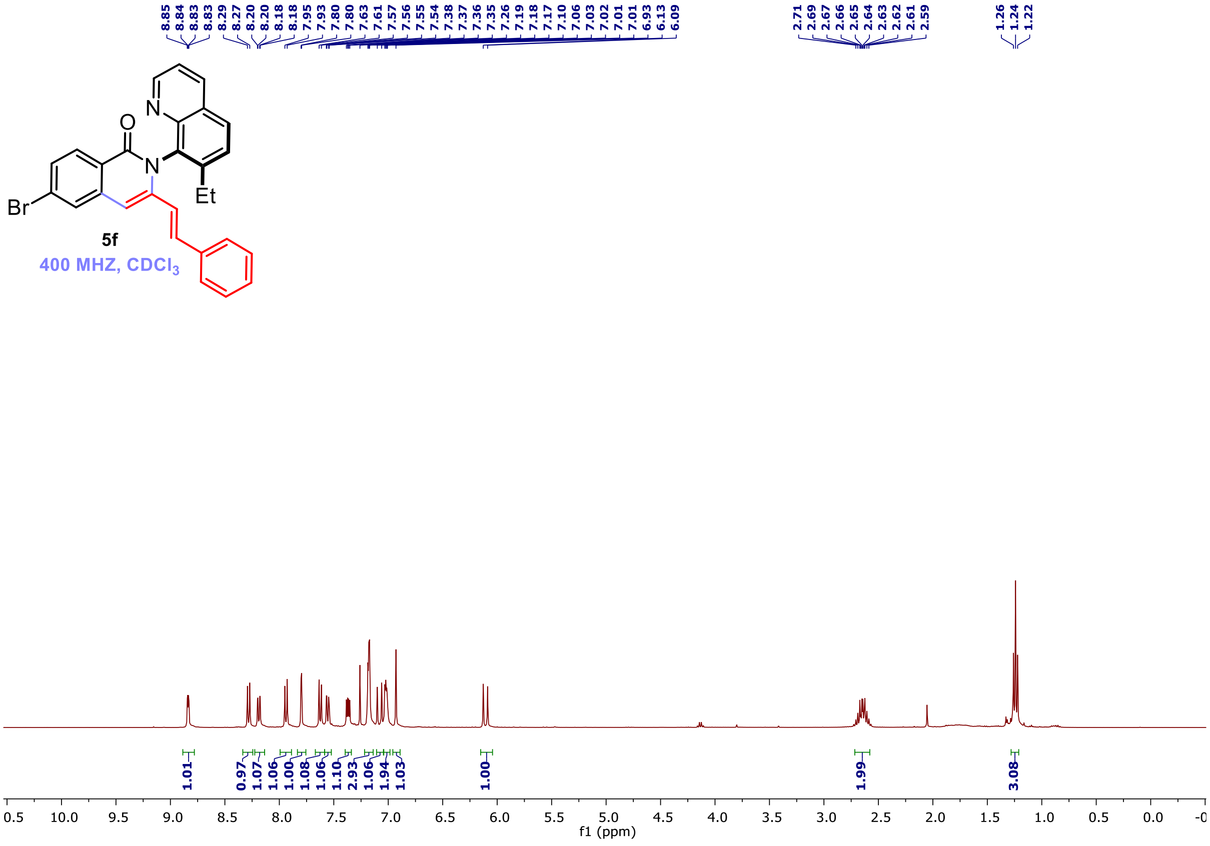
8.85 8.84 8.83 8.83 8.29 8.27 8.20 8.20 8.18 8.18 7.95 7.93 7.80 7.80 7.63 7.61 7.57 7.56 7.55 7.54 7.38 7.37 7.36 7.35 7.26 7.19 7.18 7.17 7.10 7.06 7.03 7.02 7.01 7.01 6.93 6.13 6.09
2.71 2.69 2.67 2.66 2.65 2.64 2.63 2.62 2.61 2.59
1.26 1.24 1.22
Br
N
O
N
Et
5f
400 MHZ, CDCl3
1.01 0.97 1.07 1.06 1.00 1.08 1.06 1.10 2.93 1.06 1.94 1.03 1.00 1.99 3.08
f1 (ppm)

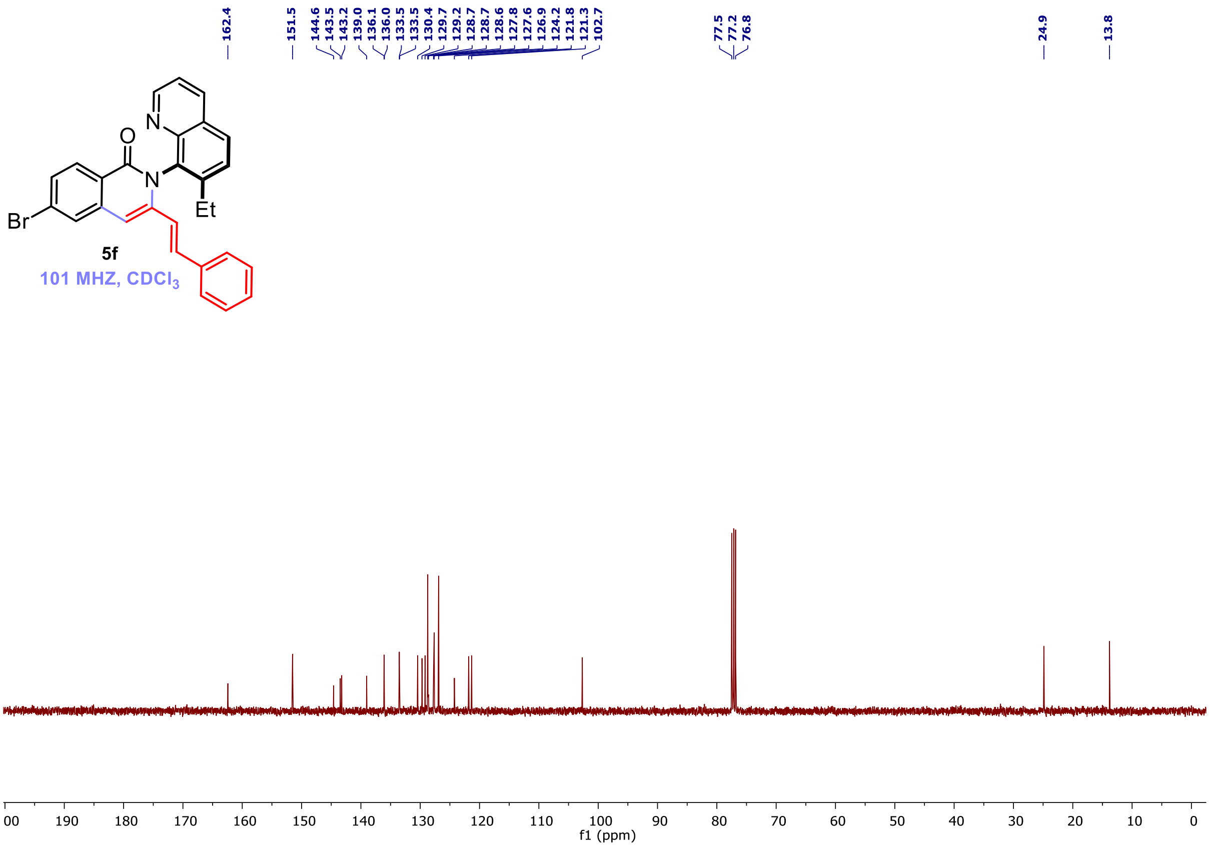
162.4
151.5
144.6 143.5 143.2 139.0 136.1 136.0 133.5 133.5 130.4 129.7 129.2 128.7 128.7 128.6 127.8 127.6 126.9 124.2 121.8 121.3 102.7
77.5 77.2 76.8
24.9
13.8
Br
N
O
N
Et
5f
101 MHZ, CDCl3
f1 (ppm)

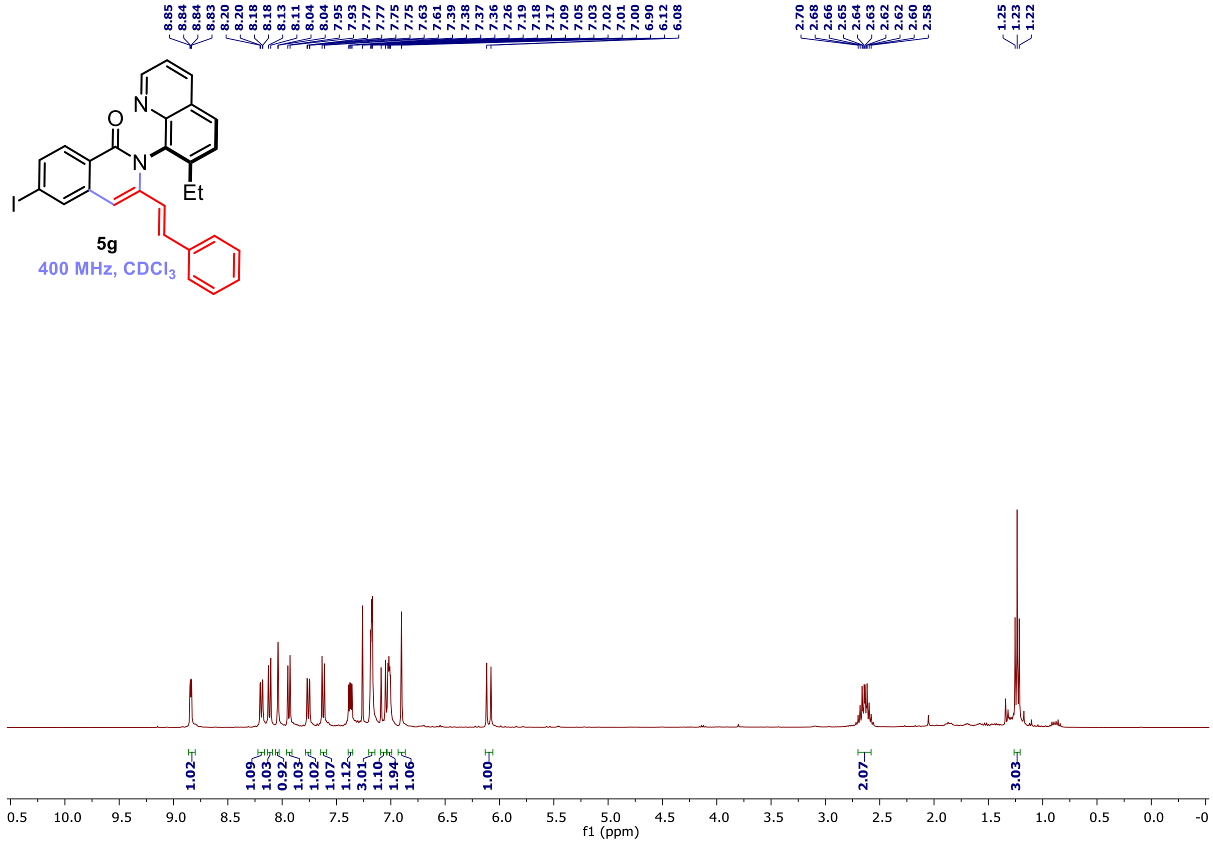
5g
400 MHz, CDCl3
f1 (ppm)

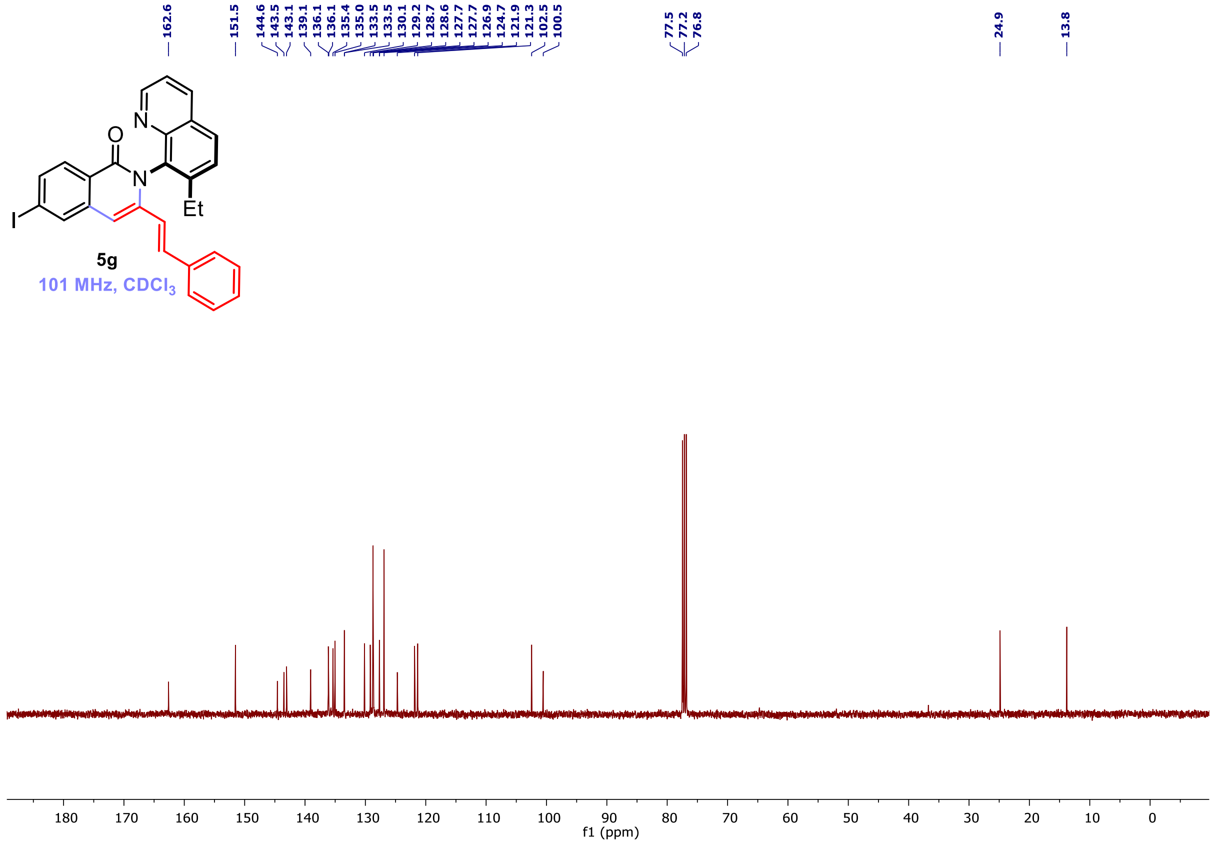
5g
101 MHz, CDCl3
f1 (ppm)

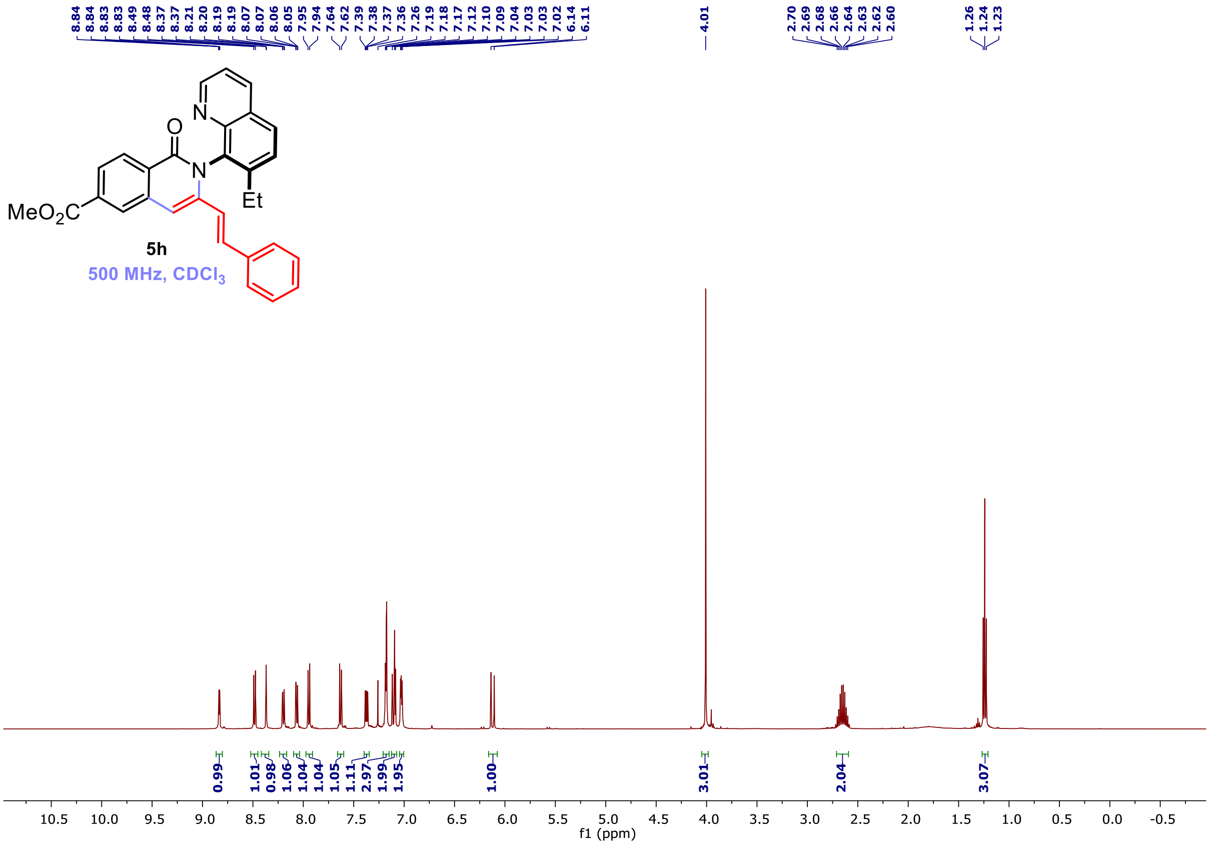
8.84 8.84 8.83 8.83 8.49 8.48 8.37 8.37 8.21 8.20 8.19 8.19 8.07 8.06 8.05 7.95 7.94 7.64 7.62 7.39 7.38 7.37 7.36 7.26 7.19 7.18 7.17 7.12 7.10 7.09 7.04 7.03 7.03 7.02 6.14 6.11
4.01
2.70 2.69 2.68 2.66 2.64 2.63 2.62 2.60
1.26 1.24 1.23
MeO2C
O
N
N
Et
5h
500 MHz, CDCl3
0.99 1.01 0.98 1.06 1.04 1.04 1.05 1.11 2.97 1.99 1.95 1.00 3.01 2.04 3.07
10.5 10.0 9.5 9.0 8.5 8.0 7.5 7.0 6.5 6.0 5.5 5.0 4.5 4.0 3.5 3.0 2.5 2.0 1.5 1.0 0.5 0.0 -0.5
f1 (ppm)

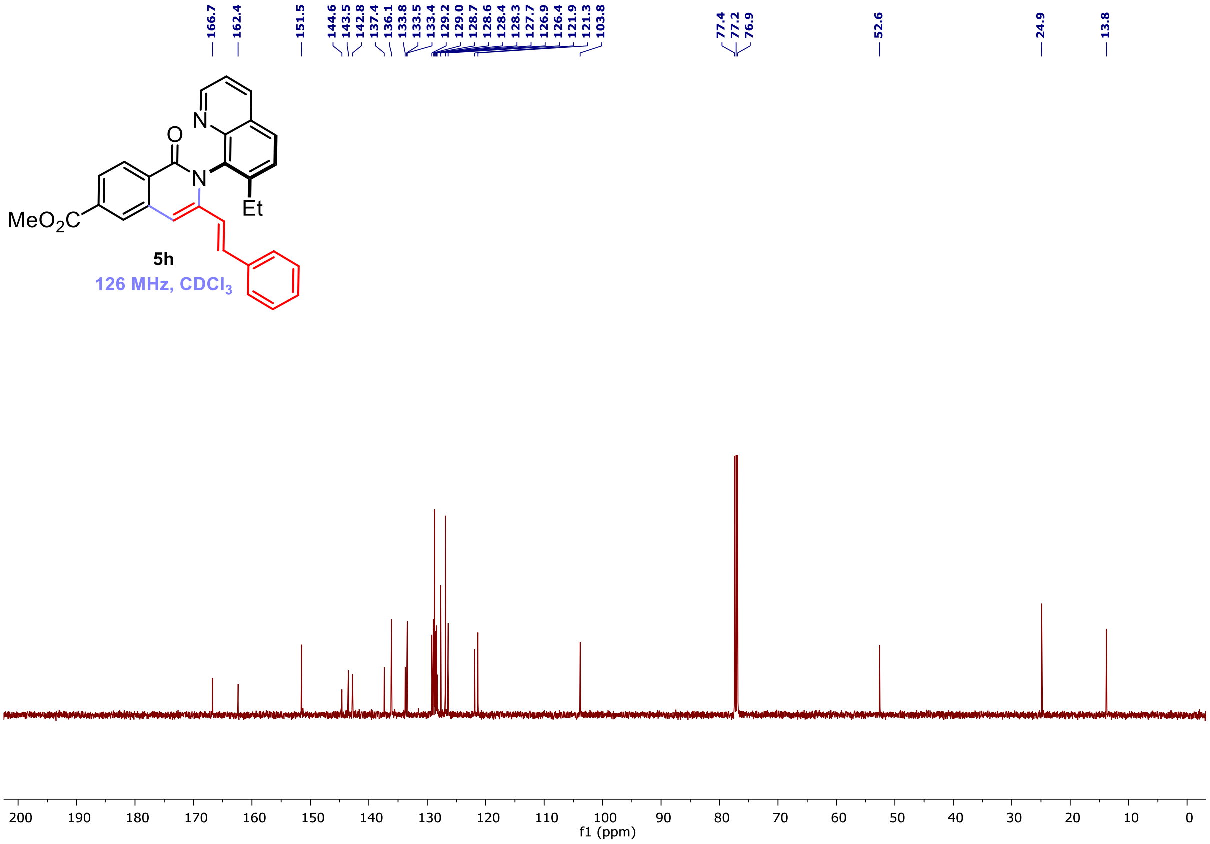
166.7
162.4
151.5
144.6 143.5 142.8 137.4 136.1 133.8 133.5 133.4 129.2 129.0 128.7 128.6 128.4 128.3 127.7 126.9 126.4 121.9 121.3 103.8
77.4 77.2 76.9
52.6
24.9
13.8
MeO2C
O
N
N
Et
5h
126 MHz, CDCl3
200 190 180 170 160 150 140 130 120 110 100 90 80 70 60 50 40 30 20 10 0
f1 (ppm)

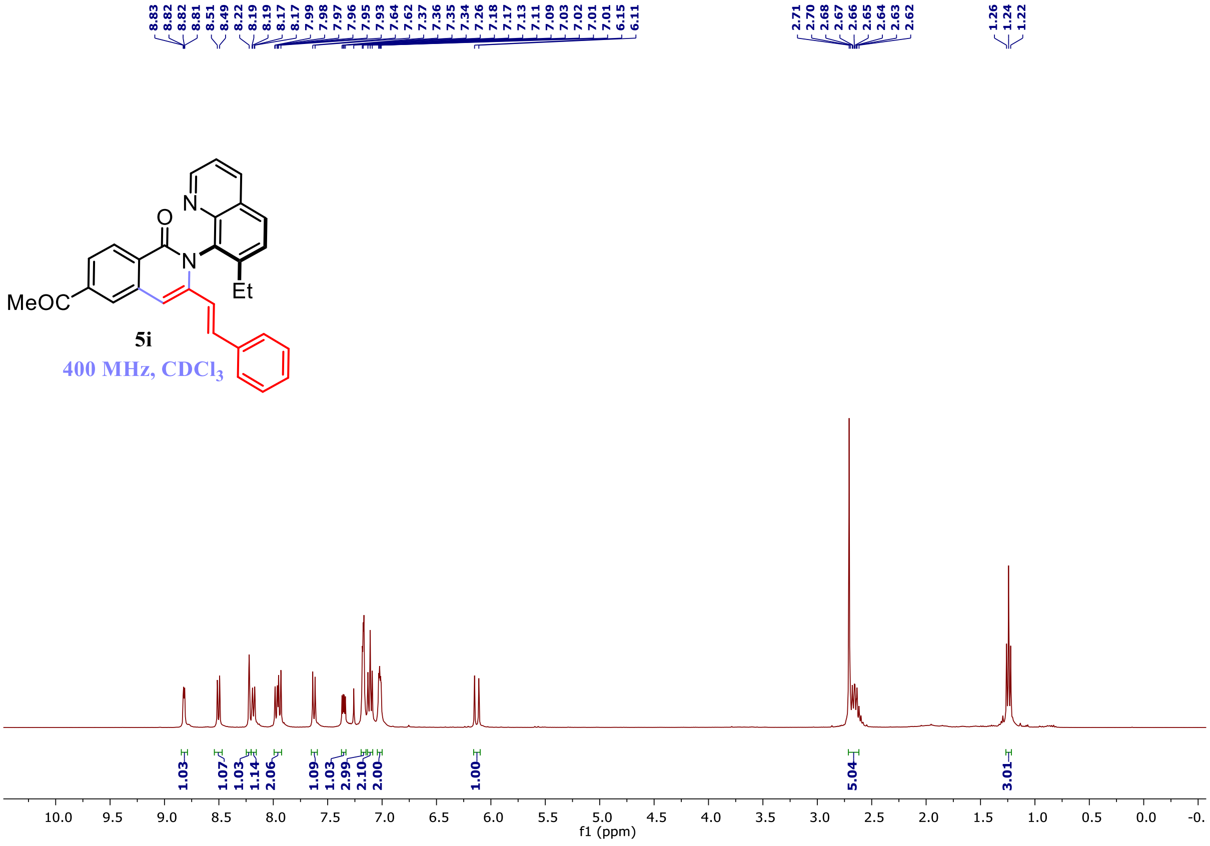

8.83
8.82
8.82
8.81
8.51
8.49
8.22
8.19
8.19
8.17
8.17
7.99
7.98
7.97
7.96
7.95
7.93
7.64
7.62
7.37
7.36
7.35
7.34
7.26
7.18
7.17
7.13
7.11
7.09
7.03
7.02
7.01
6.15
6.11
2.71
2.70
2.68
2.67
2.66
2.65
2.64
2.63
2.62
1.26
1.24
1.22
MeOC
N
O
N
Et
5i
400 MHz, CDCl3
1.03
1.07
1.03
1.14
2.06
1.09
1.03
2.99
2.10
2.00
1.00
5.04
3.01
f1 (ppm)

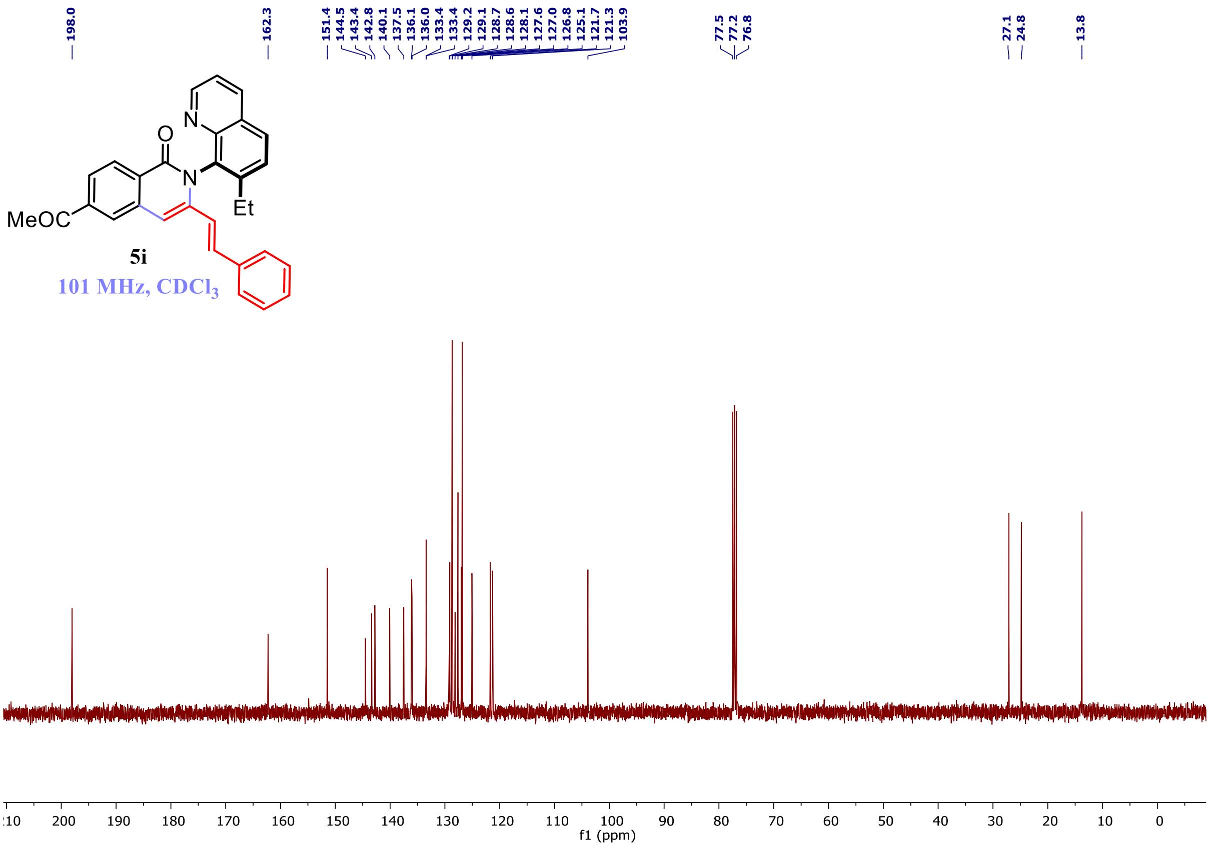

198.0
162.3
151.4
144.5
143.4
142.8
140.1
137.5
136.1
136.0
133.4
133.4
129.2
129.1
128.7
128.6
128.1
127.6
127.0
126.8
125.1
121.7
121.3
103.9
77.5
77.2
76.8
27.1
24.8
13.8
MeOC
N
O
N
Et
5i
101 MHz, CDCl3
f1 (ppm)

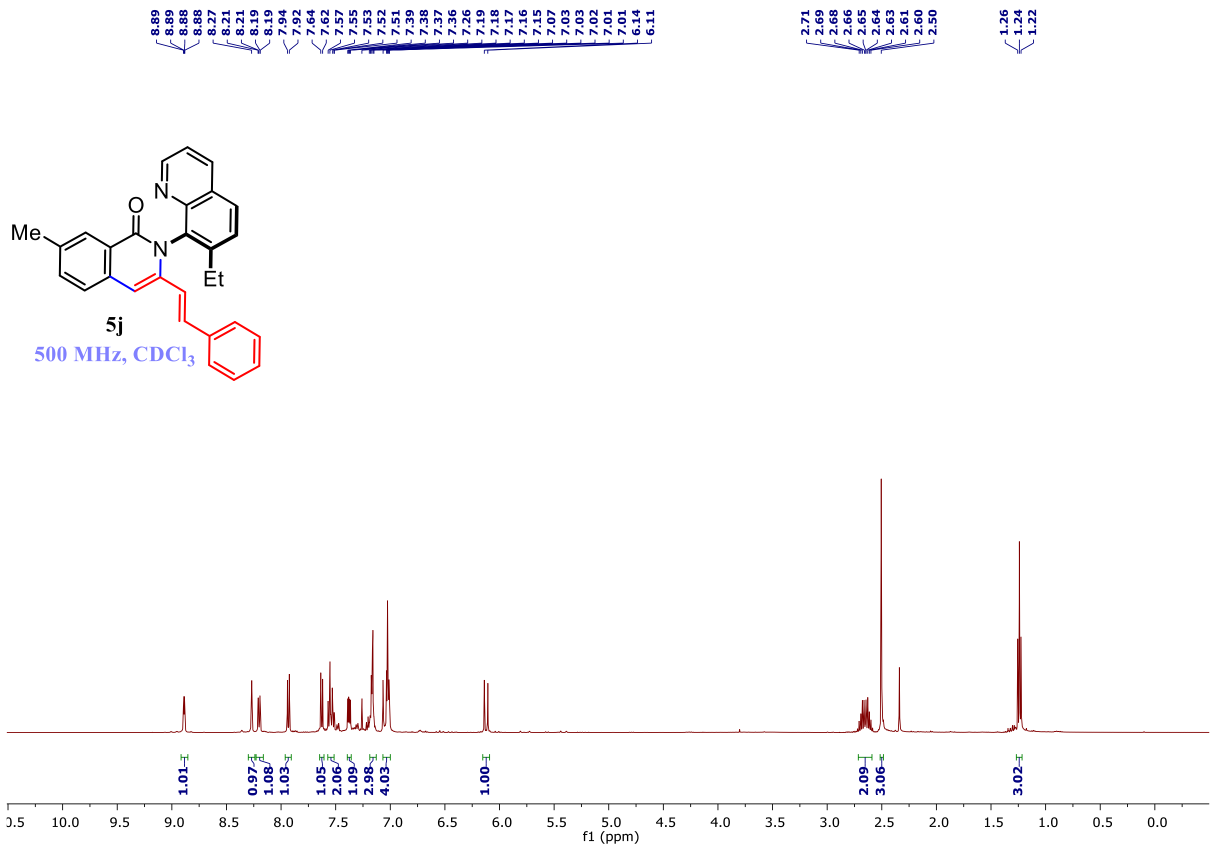

5j
500 MHz, CDCl3
f1 (ppm)

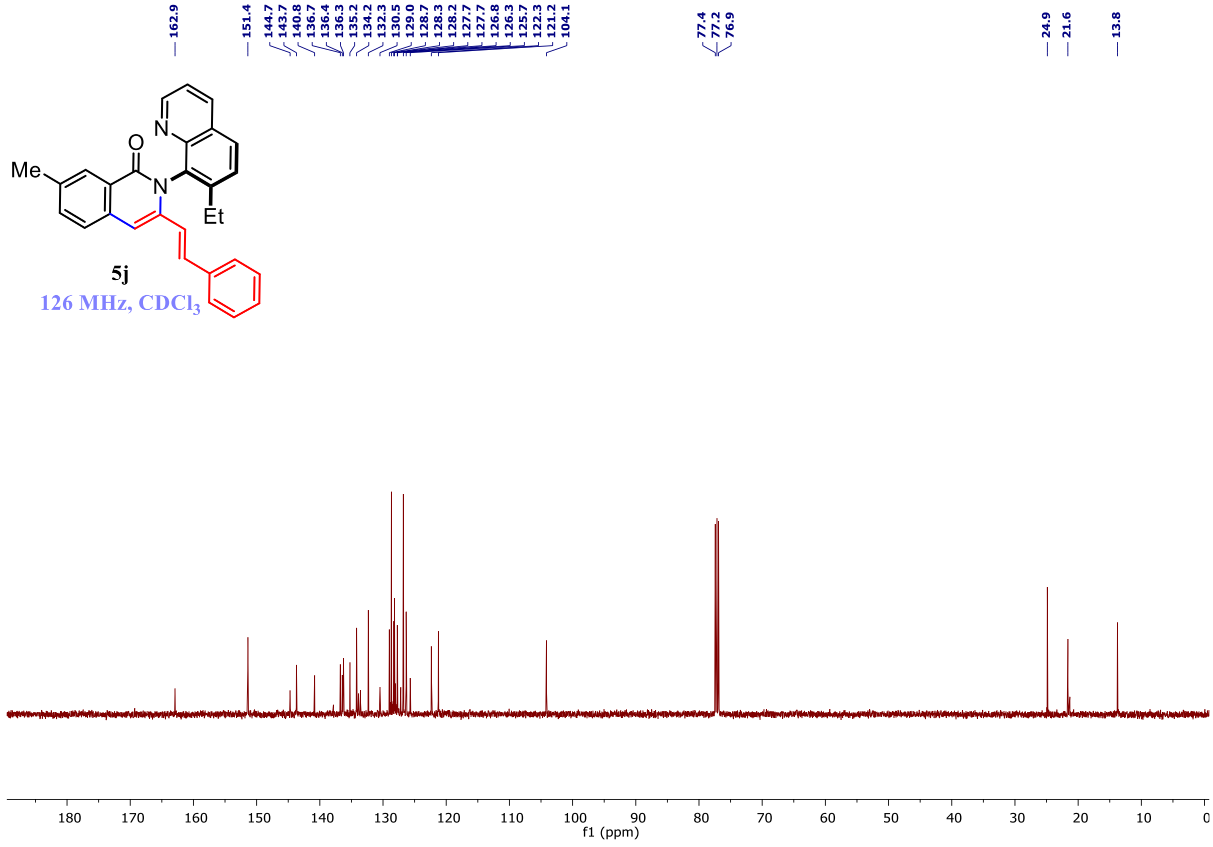

5j
126 MHz, CDCl3
f1 (ppm)

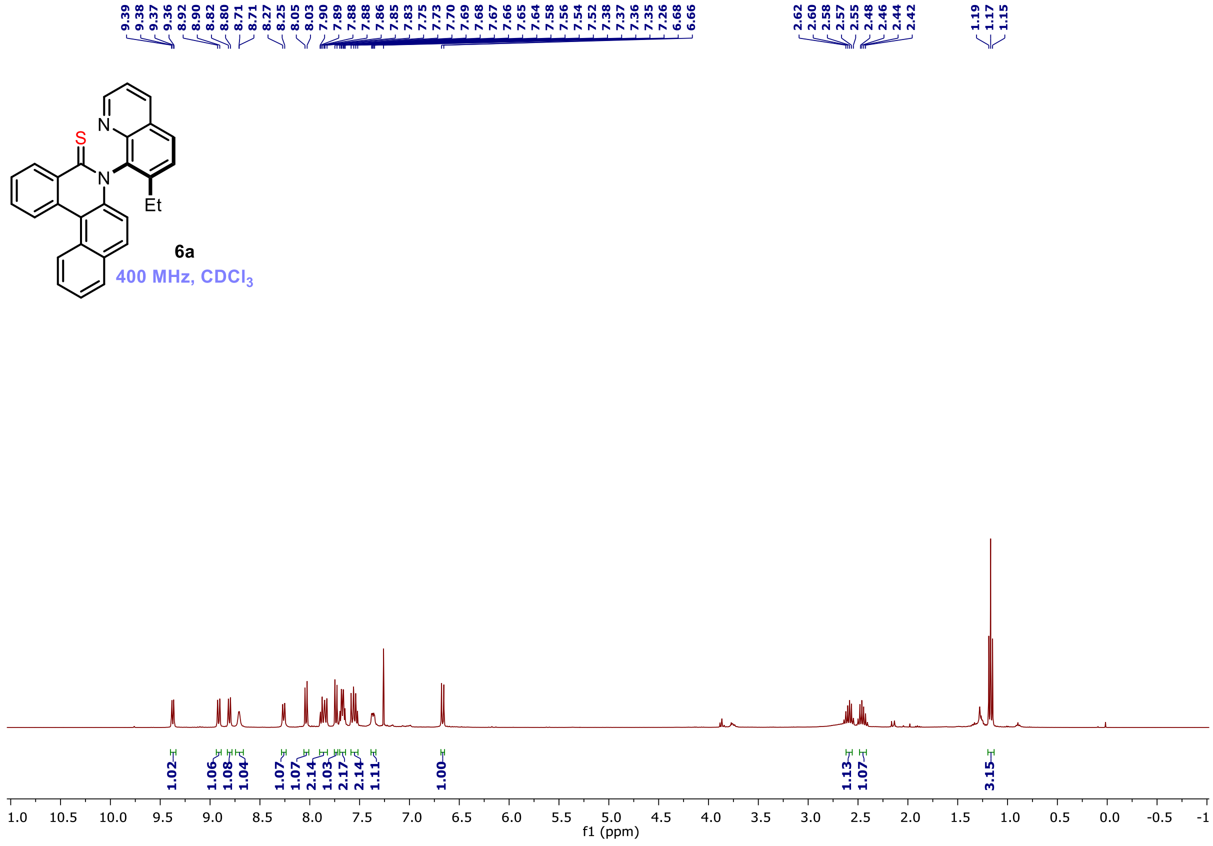
9.39 9.38 9.37 9.36 8.92 8.90 8.82 8.80 8.71 8.27 8.25 8.05 8.03 7.90 7.89 7.88 7.86 7.85 7.83 7.75 7.73 7.70 7.69 7.68 7.67 7.66 7.65 7.64 7.58 7.56 7.54 7.52 7.38 7.37 7.36 7.35 7.26 6.68 6.66
2.62 2.60 2.58 2.57 2.55 2.48 2.46 2.44 2.42
1.19 1.17 1.15
S
N
N
Et
6a
400 MHz, CDCl3
1.02 1.06 1.08 1.04 1.07 1.07 2.14 1.03 2.17 2.14 1.11 1.00 1.13 1.07 3.15
f1 (ppm)

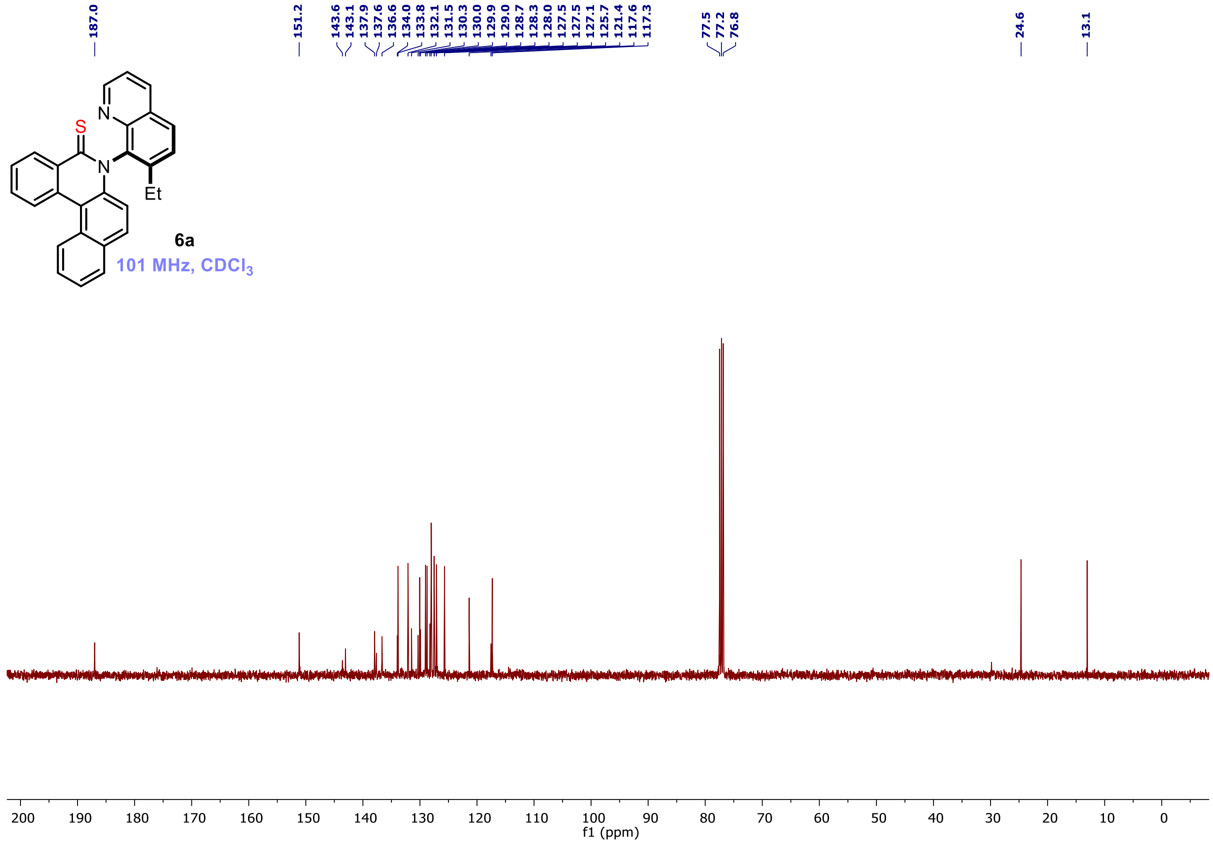
187.0
151.2
143.6 143.1 137.9 137.6 136.6 134.0 133.8 132.1 131.5 130.3 130.0 129.9 129.0 128.7 128.3 128.0 127.5 127.5 127.1 125.7 121.4 117.6 117.3
77.5 77.2 76.8
24.6
13.1
S
N
N
Et
6a
101 MHz, CDCl3
f1 (ppm)

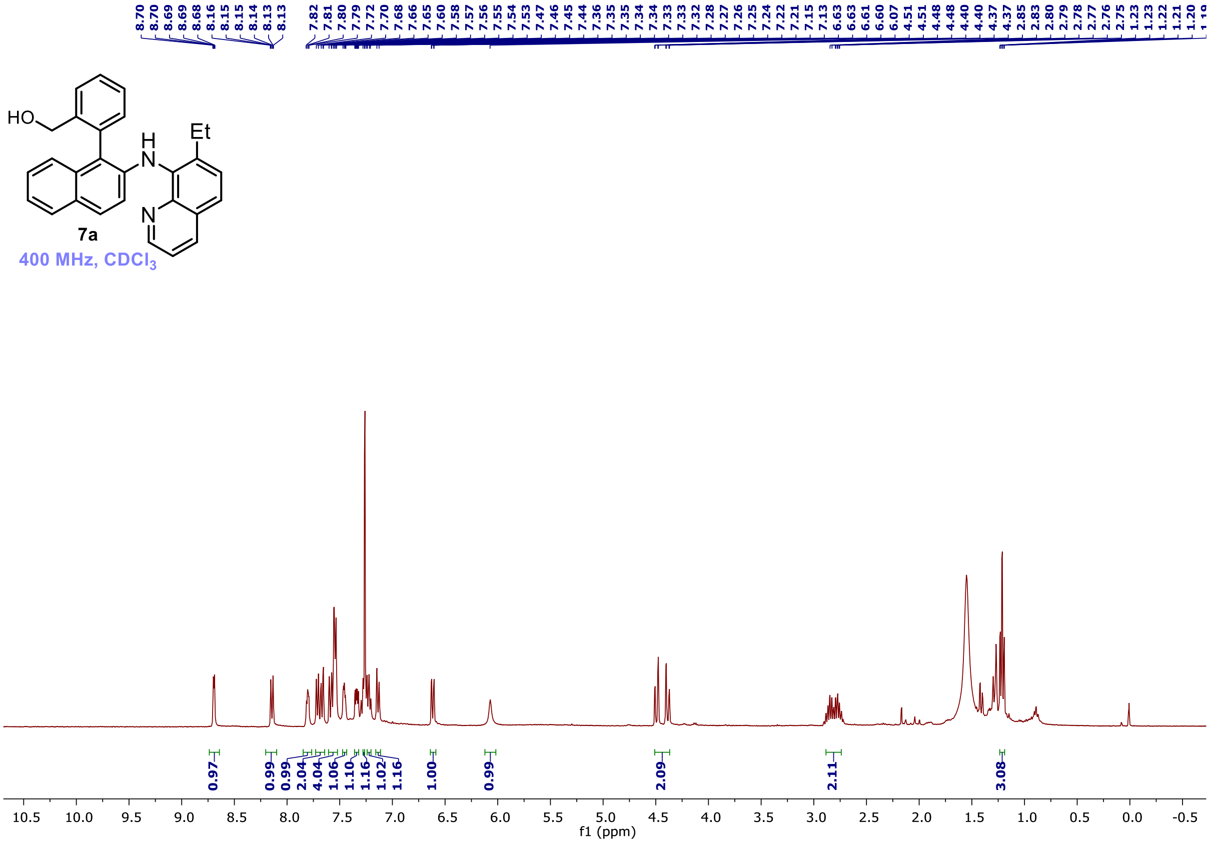
HO
H
N
Et
N
7a
400 MHz, CDCl3
f1 (ppm)

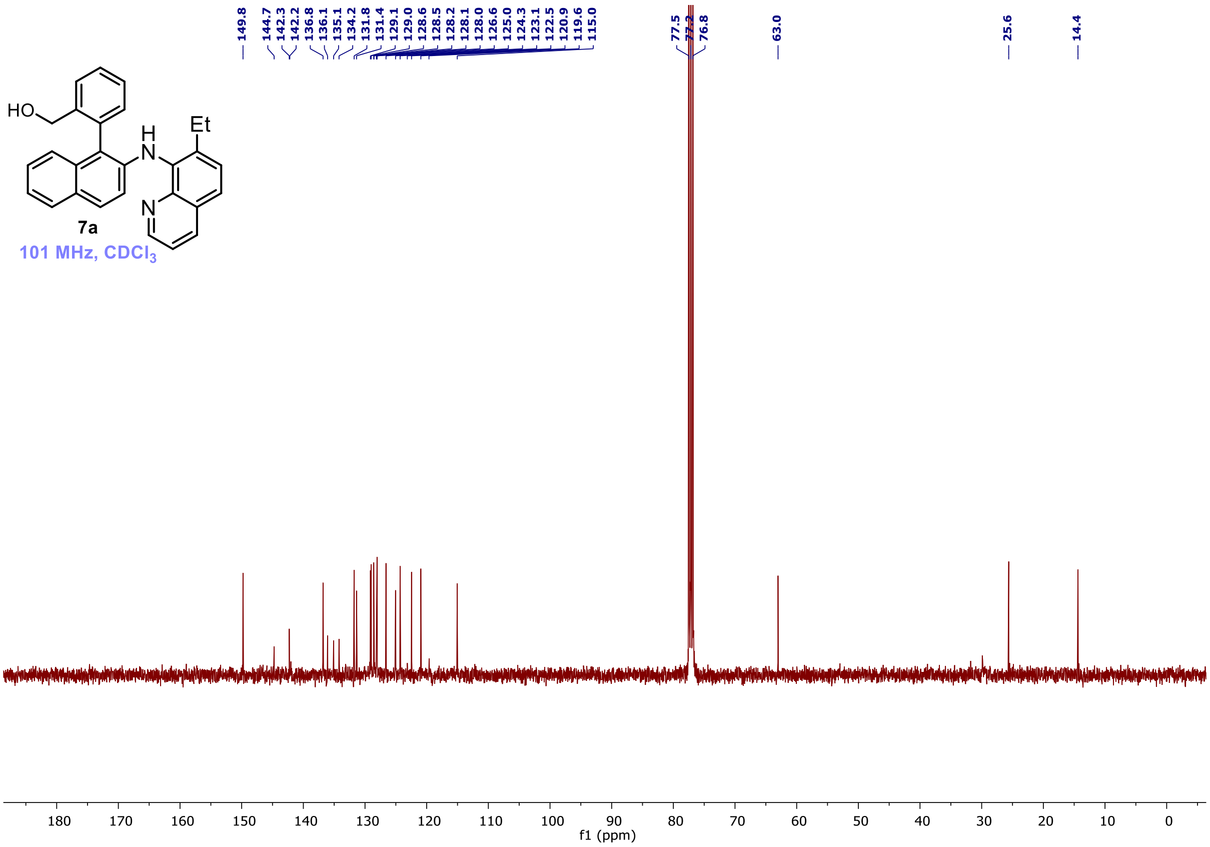
HO
H
N
Et
N
7a
101 MHz, CDCl3
149.8
144.7
142.3
142.2
136.8
136.1
135.1
134.2
131.8
131.4
129.1
129.0
128.6
128.5
128.2
128.1
128.0
126.6
125.0
124.3
123.1
122.5
120.9
119.6
115.0
77.5
76.8
63.0
25.6
14.4
f1 (ppm)
