# Supplementary material for: Multifaceted photocatalysis enables cobalt catalyzed enantioselective C–H activation and APEX reaction for C–N axially chiral molecules
Source: Chem Sci. 2025 Sep 11;16(41):19296–303. doi: 10.1039/d5sc05287d (PMC12447758; doi:10.1039/d5sc05287d)
Supplement: SC-016-D5SC05287D-s002 [file SC-016-D5SC05287D-s002.pdf]

Supplementary Information (SI) for Chemical Science.
This journal is © The Royal Society of Chemistry 2025

Electronic Supporting Information

PART B

Multifaceted Photocatalysis Enables Cobalt Catalyzed Enantioselective C–H Activation and APEX Reaction for C–N Axially Chiral Molecules

Mainak Koner,[a] Nityananda Ballav,[a†] Anirudh J Varma,[b†] Suman Ghosh,[a] Tuhin Mondal,[a] Rositha Kuniyil,*[b] and Mahiuddin Baidya*[a]

[a]Department of Chemistry, Indian Institute of Technology Madras

Chennai 600036, Tamil Nadu, India

E-mail: mbaidya@iitm.ac.in

[b]Department of Chemistry, Indian Institute of Technology Palakkad

Palakkad 678623, Kerala, India

E-mail: rosithak@iitpkd.ac.in

[†]These authors contributed equally to the work.

Computational Details

Gaussian 16 software suite was used for all the optimization and single point energy calculations.[1] All the structures were optimized in the gas phase using M06 functional [2-4] along with Grimme's D3 dispersion correction.[5] The basis set used was 6-31g(d)[6] for all the atoms except cobalt. An SDD basis set along with the ECP was used for cobalt.[7] A vibrational analysis was performed on the optimized geometries to ensure that they are the equilibrium geometries. Transition states were characterized by a single imaginary frequency and all the intermediates were characterized by the absence of any imaginary vibrational frequency.

Single-point energy calculation on the optimized geometries were performed using the same functional and 6-311++g(d,p) basis set for all the atoms except cobalt. An SDD basis set along with the ECP was used for cobalt. The solvation effects were considered in single point energy calculation for the solvent 2,2,2-Trifluoroethanol (ε = 26.7260) by using the SMD solvation model.[8] The transition state geometries were generated using CYLview.[9] The Non covalent interactions present in the selective transition states were plotted using Non covalent interaction plot by Multiwfn version 3.8(dev).[10] Unless otherwise stated, all the reported energies are in Gibbs free energies (kcal/ mol).

Activation Strain Analysis

Activation strain analysis was conducted on the migratory insertion transition states **TS3** and **TS3′** (Table S1). From Table S1, it is clear that the destabilizing distortion energy for **TS3** is lower than **TS3′**, and the stabilizing interaction energy is found to be higher for **TS3**. Hence, the total activation strain energy favors the transition state **TS3** over **TS3′**.

Table S1. Activation strain analysis of migratory insertion transition states **TS3** and **TS3′**. Energies are in kcal/mol.

TRANSITION STATES	TS3	TS3′
Distortion Energy	13.33	16.60
Interaction Energy	-43.09	-37.83
Activation Strain Energy	-29.75	-21.22

The degree of distortion (E_{dis}) and interaction energies (E_{int}) in transition states **TS3** and **TS3′** were calculated using the activation strain analysis. The activation strain energy ($E^{‡}_{act}$) can be written as the sum of distortion (ΔE_{dis}) and interaction energies (ΔE_{int}).[11]

$$\Delta E^{‡}_{act} = \Delta E_{int} + \Delta E_{dis} \quad (1)$$

For this analysis, at first, seven different points were selected from the reaction profile obtained from IRC calculations (Figure S1 a). The corresponding structures were made into two fragments; one allene part and metal complex part. Single-point computations were carried on those structures (Figure S1b). The IRC points toward the product side (P1, P2, P3) and the reactant side (R1, R2, R3) along the reaction coordinate are shown in Figure S1a. ΔE_{dis} and ΔE_{int} of the structures in each point are calculated.

Equations used for calculating distortion energies and interaction energies are given below.[12]

$$\Delta E^{\ddagger}_{dis} = [\{ E_{f2(TS)} + E_{f1(TS)}\} - \{E_{f2} + E_{f1}\}] \quad (2)$$

$$\Delta E^{\ddagger}_{int} = \{ E_{(TS)} - (E_{f1(TS)} + E_{f2(TS)})\} \quad (3)$$

Where $E_{f1(TS)}$ is the single point energy of f1 fragment in transition state, $E_{f2(TS)}$ is the single point energy of f2 fragment in transition state, E_{f1} is the single point energy of f1 fragment in the intermediates and E_{f2} is the single point energy of f2 fragment in the intermediates

a)

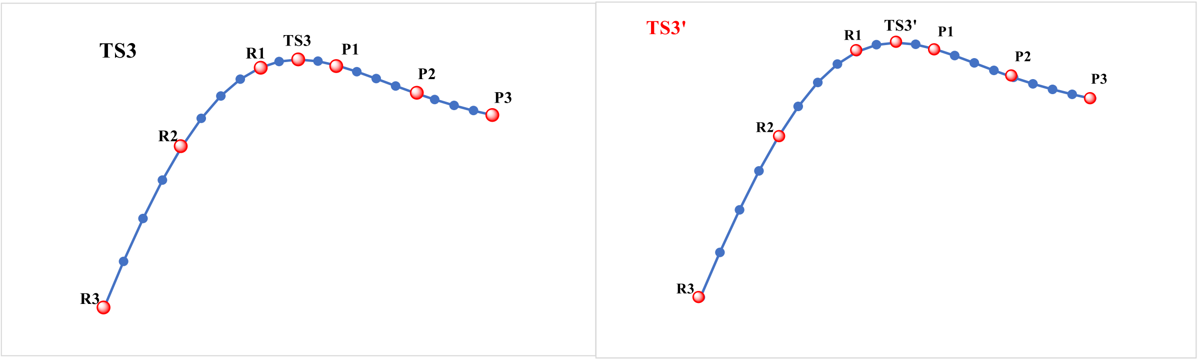

b)

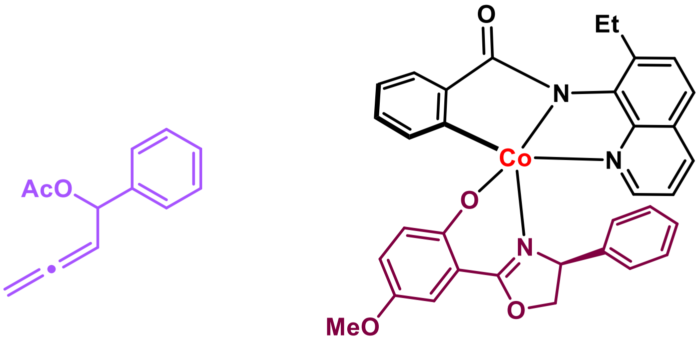

F1 F2

Figure S1: a) The reaction profile along the intrinsic reaction coordinate (IRC) trajectory for **TS3** and **TS3′**. b) **F1** and **F2** fragments used for activation strain analysis.

Table S2: Relative distortion energy and interaction energy (in kcal/mol) for **TS3** and **TS3′**

**Transition States**	**Distortion Energy of Fragments**		**Total Distortion energy (E_{dis})**	**Interaction Energy (E_i)**	**Activation Strain Energy**
	**F1**	**F2**	**F1+F2**		
**TS3**	10.27	3.06	13.33	-43.09	-29.75
P1	8.02	2.31	10.34	-40.09	-29.75
P2	4.98	1.43	6.42	-36.17	-29.75
P3	3.32	0.89	4.22	-33.98	-29.75
R1	13.02	4.12	17.15	-46.90	-29.75
R2	20.12	7.37	27.49	-57.25	-29.75
R3	28.43	12.05	40.48	-70.24	-29.75
**TS3′**	12.02	4.57	16.60	-37.83	-21.22
P1	9.78	3.57	13.36	-34.58	-21.22
P2	6.83	2.32	9.16	-30.38	-21.22
P3	5.04	1.60	6.64	-27.87	-21.22
R1	29.84	15.23	45.08	-66.30	-21.22
R2	21.81	9.80	31.62	-52.84	-21.22
R3	14.78	5.90	20.69	-41.91	-21.22

From the table S2 it is evident that the total distortion energy, which has a destabilizing effect, is smaller for **TS3** compared to the **TS3′**. In the case of **TS3**, on moving along the IRC path from **R3** to **R1**, **TS3** followed by **P1** to **P3** the distortion energy is found to be decreasing. While for **TS3′**, on moving along the IRC path from **R3** to **R1,** the distortion energy increases, and from **TS3′** followed by **P1** to **P3,** the distortion energy decreases. It is also clear that the total de-stabilising distortion energy of **TS3′** is higher than **TS3**, whereas the stabilising interaction energy is higher in **TS3.** Therefore, the lower distortion energy and higher interaction energy selectively prefers **TS3** over **TS3′**.

Reference:

1. Gaussian 16, Revision C.01, M. J. Frisch, G. W. Trucks, H. B. Schlegel, G. E. Scuseria, M. A. Robb, J. R. Cheeseman, G. Scalmani, V. Barone, G. A. Petersson, H. Nakatsuji, X. Li, M. Caricato, A. V. Marenich, J. Bloino, B. G. Janesko, R. Gomperts, B. Mennucci, H. P. Hratchian, J. V. Ortiz, A. F. Izmaylov, J. L. Sonnenberg, D. Williams-Young, F. Ding, F. Lipparini, F. Egidi, J. Goings, B. Peng, A. Petrone, T. Henderson, D. Ranasinghe, V. G. Zakrzewski, J. Gao, N. Rega, G. Zheng, W. Liang, M. Hada, M. Ehara, K. Toyota, R. Fukuda, J. Hasegawa, M. Ishida, T. Nakajima, Y. Honda, O. Kitao,

H. Nakai, T. Vreven, K. Throssell, J. A. Montgomery, Jr., J. E. Peralta, F. Ogliaro, M. J. Bearpark, J. J. Heyd, E. N. Brothers, K. N. Kudin, V. N. Staroverov, T. A. Keith, R. Kobayashi, J. Normand, K. Raghavachari, A. P. Rendell, J. C. Burant, S. S. Iyengar, J. Tomasi, M. Cossi, J. M. Millam, M. Klene, C. Adamo, R. Cammi, J. W. Ochterski, R. L. Martin, K. Morokuma, O. Farkas, J. B. Foresman and D. J. Fox, Gaussian, Inc., Wallingford CT (**2016**).

2. Y. Zhao and D. G. Truhlar, *Acc. Chem. Res.,* 2008, **41**, 157–167.
3. C. W. Davis, Y. Zhang, Y. Li, M. Martinelli, J. Zhang, C. Ungarean, P. Galer, P. Liu and D. Sarlah, *Angew. Chem. Int. Ed.,* 2024, **63**. No. e202407281.
4. H. M. Omer and P. Liu, *J. Am. Chem. Soc.,* 2017, **139**, 9909–9920.
5. S. Grimme, J. Antony, S. Ehrlich and H. Krieg, *J. Chem. Phys*., 2010, **132**, 154104–154119.
6. W. J. Hehre, R. Ditchfield and J. A. Pople, *J. Chem. Phys.,* 1972, **56**, 2257–2261.
7. D. Andrae, U. Haussermann, M. Dolg, H. Stoll and H. Preuss, *Theor. Chim. Acta,* 1990, **77***,* 123–141.
8. A. V. Marenich, C. J. Cramer and D. G. Truhlar, *J. Phys. Chem. B*., 2009, **113**, 6378–6396.
9. C. Y. Legault, CYLview20; Université de Sherbrooke, **2020**; http://www.cylview.org.
10. T. Lu, F. Chen and *J. Comput. Chem*., 2012, **33**, 580−592.
11. W. J. Van Zeist and F. M. Bickelhaupt, *Org. Biomol. Chem.,* 2010, **8**, 3118–3127.
12. S. Singh, K. Surya and R. B. Sunoj, *J. Org. Chem.,* 2017, **82**, 9619–9626.

Cartesian Coordinates of the Optimized Geometries

A

SCF Energy (a.u.) = -2616.705239

Thermal correction to Gibbs free Energy (a.u.) = 0.766141

Charge = 0, Multiplicity = 1

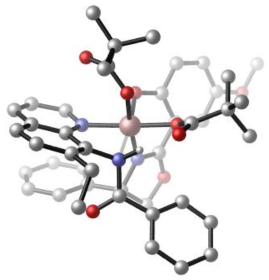

A

Co	-0.05242400	-0.01001700	-0.41665000
O	2.69819400	0.89171500	2.23626900
O	-1.24997400	0.40011400	-1.78453400
O	-2.39533100	2.85307400	1.30830500
N	1.38262400	0.93620600	-1.29566500
N	1.49658300	-0.56013400	0.92918800

N	-0.73452900	1.55795100	0.56246100
C	1.25486200	1.75957100	-2.31165000
H	0.24261200	2.09885500	-2.52197800
C	2.35370800	2.13701400	-3.10412700
H	2.20478100	2.84660600	-3.91318200
C	3.57146200	1.54720400	-2.87932100
H	4.42882500	1.77222900	-3.51357500
C	3.71929900	0.61464200	-1.82895000
C	2.59116300	0.37425000	-1.01615100
C	2.65946200	-0.52395600	0.06808600
C	3.80224100	-1.25095100	0.31025300
C	4.91947500	-1.03620700	-0.53947200
H	5.82146800	-1.62123400	-0.35635600
C	4.89846700	-0.11800600	-1.55709300
H	5.77571300	0.04009800	-2.18366900
C	-2.51716800	0.54802000	-1.49265500
C	-3.49216900	0.06092300	-2.38127800
H	-3.14276500	-0.47719600	-3.26029100
C	-4.84434200	0.23997200	-2.14247400
H	-5.55958000	-0.16466000	-2.85556400
C	-5.28705500	0.90852100	-0.99069400
C	-4.35040800	1.41488200	-0.10406900
H	-4.69347000	1.94665800	0.78097800
C	-2.98307400	1.24415600	-0.34562800
C	-2.00267100	1.84206000	0.51790400
C	-1.24991900	3.22472700	2.09351500
H	-1.36446500	2.78163200	3.09219500
H	-1.22648200	4.31593700	2.16785900
C	-0.06352200	2.62580100	1.33589700
H	0.64916400	2.19504900	2.04564400
C	0.67387600	3.58232000	0.43092800
C	-0.02084700	4.38701100	-0.47577400
H	-1.11046800	4.34031300	-0.52297500
C	0.66881800	5.24204800	-1.32752400

H	0.11821900	5.86719400	-2.02876200
C	2.06064900	5.29505000	-1.28571700
H	2.60099800	5.96139400	-1.95649900
C	2.75703800	4.49323300	-0.38703800
H	3.84518500	4.52371400	-0.35509600
C	2.06626800	3.64306800	0.47208500
H	2.60900300	2.99819000	1.16509100
C	-1.19290400	-2.28106000	0.86006800
C	-2.48119900	-3.10274600	0.97201500
O	-0.11170000	-2.73541300	1.26061900
O	-1.39205500	-1.08824200	0.40859600
C	-3.28546700	-2.98091500	-0.32105900
H	-3.64150500	-1.95512400	-0.47469200
H	-2.67801800	-3.25486200	-1.19607200
H	-4.15651800	-3.65148700	-0.28488800
C	-3.28780400	-2.51078700	2.13215900
H	-2.73099500	-2.59184200	3.07816500
H	-3.52075000	-1.45202100	1.94592200
H	-4.23570100	-3.05528400	2.25036400
C	-2.15370200	-4.56165800	1.26200000
H	-1.55298200	-4.66131300	2.17349500
H	-3.08357000	-5.13473900	1.38804600
H	-1.58380800	-5.01284600	0.43860900
C	1.75349200	0.12713700	2.15459400
O	-6.59583900	1.11074900	-0.66952800
C	-7.56527100	0.57296300	-1.52770800
H	-7.47985600	-0.52255800	-1.60572000
H	-8.53721200	0.82377300	-1.09398700
H	-7.50371500	1.00573800	-2.53899800
C	3.91950800	-2.23030800	1.44346900
H	4.29655200	-3.18377300	1.04172300
H	2.92824000	-2.45692400	1.86257200
C	4.85276400	-1.73180500	2.54476400
H	4.51478300	-0.76304200	2.93323400

H	4.89557800	-2.44629100	3.37539000
H	5.87448300	-1.59306700	2.16715200
C	0.89283200	-0.12721700	3.34005800
C	-0.48838200	-0.33215800	3.29281400
C	1.53897000	-0.05806500	4.58098900
C	-1.20733400	-0.46380600	4.47635500
H	-1.00850000	-0.35627500	2.33805500
C	0.82151700	-0.22638400	5.75584700
H	2.61058600	0.13060600	4.60423900
C	-0.55652200	-0.42391800	5.70485300
H	-2.28672500	-0.60229600	4.43119600
H	1.33549700	-0.19219100	6.71470100
H	-1.12441000	-0.54212000	6.62639600
H	1.04628700	-1.50702100	1.06425700
C	0.85785900	-1.77581300	-2.51175000
C	1.16205500	-3.26620600	-2.76629500
O	0.46195900	-1.59462300	-1.28419600
O	1.04042200	-0.92795000	-3.37219900
C	2.33499200	-3.65957100	-1.86466800
H	3.21537500	-3.02814800	-2.06573800
H	2.06330600	-3.55043800	-0.80555500
H	2.62137300	-4.70651200	-2.04479000
C	1.53738600	-3.46918300	-4.22737100
H	0.71462400	-3.17602000	-4.89172300
H	2.40832400	-2.86021500	-4.49864600
H	1.77502500	-4.52721400	-4.41216300
C	-0.05793000	-4.11249600	-2.40899800
H	-0.93888200	-3.80373300	-2.99123100
H	0.13593400	-5.17338000	-2.62693400
H	-0.29710500	-4.01538400	-1.34231200

TS1

SCF Energy (a.u.) = -2616.697262

Thermal correction to Gibbs free Energy (a.u.) = 0.762705

Charge = 0, Multiplicity = 1

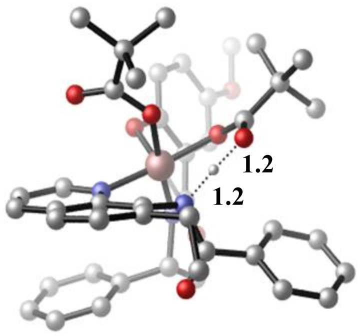

TS1

Co	-0.05003800	0.06825300	-0.44124400
O	2.58441600	0.52425700	2.48213700
O	-1.21884700	0.62143100	-1.79672400
O	-2.37520600	2.82744300	1.46877300
N	1.40702900	1.05546100	-1.18282600
N	1.38558200	-0.63798200	0.89571100
N	-0.72978000	1.55640900	0.65301800
C	1.32735000	1.96293300	-2.12957200
H	0.32811500	2.33064600	-2.35283600
C	2.46448000	2.39057100	-2.83755200
H	2.35911200	3.16749400	-3.58961000
C	3.66504800	1.76671200	-2.61028200
H	4.55157600	2.03347600	-3.18573200
C	3.75939100	0.74708500	-1.63766100
C	2.59523400	0.45336600	-0.89532100
C	2.59189300	-0.52614700	0.11983200
C	3.72301700	-1.28320100	0.34485800
C	4.87949700	-1.01377500	-0.43259500
H	5.76657600	-1.62337100	-0.25545300
C	4.91883600	-0.01799500	-1.37444400
H	5.82611600	0.17843400	-1.94477500
C	-2.48838200	0.74920800	-1.50798400
C	-3.45894200	0.31933400	-2.43089600
H	-3.10441400	-0.15374800	-3.34463900
C	-4.81304200	0.47231200	-2.18337600
H	-5.52413300	0.11259200	-2.92428800
C	-5.26315500	1.06077900	-0.99137300
C	-4.33197600	1.51082800	-0.06962700
H	-4.68036400	1.97916800	0.84863800
C	-2.96309300	1.36191200	-0.31717400

C	-1.98759700	1.88022500	0.60220000
C	-1.24323100	3.09864700	2.31449200
H	-1.41055100	2.59352200	3.27514600
H	-1.18346000	4.18025400	2.46750600
C	-0.05021400	2.51209300	1.55480400
H	0.60650100	1.97557500	2.24714600
C	0.77555900	3.51232600	0.78426800
C	0.16330500	4.45311900	-0.04708100
H	-0.92496700	4.47997400	-0.13046800
C	0.93301200	5.35055400	-0.77806900
H	0.44694800	6.08221700	-1.42162500
C	2.32294300	5.30927800	-0.68962800
H	2.92650600	6.00859000	-1.26639100
C	2.93713800	4.37142400	0.13438900
H	4.02334500	4.32790800	0.20018200
C	2.16655400	3.47864900	0.87390500
H	2.64398100	2.72681900	1.50514800
C	-1.17681000	-2.28374300	0.59500200
C	-2.36193800	-3.23463000	0.64236000
O	-0.03525700	-2.68813500	0.97291800
O	-1.42961200	-1.10030800	0.23122000
C	-3.18594800	-3.05734900	-0.63373000
H	-3.60695100	-2.04598100	-0.69760300
H	-2.57168900	-3.21907600	-1.53096300
H	-4.01089600	-3.78346600	-0.64443900
C	-3.20723500	-2.82783700	1.85481200
H	-2.63503400	-2.92285300	2.78921700
H	-3.55423200	-1.78891300	1.75752000
H	-4.08926000	-3.47939700	1.92560800
C	-1.89727600	-4.67784000	0.79149300
H	-1.29855900	-4.81435500	1.69966800
H	-2.77130300	-5.34153400	0.84624100
H	-1.28414600	-4.99149000	-0.06390000
C	1.59376400	-0.13534200	2.19374500

O	-6.57443600	1.23333900	-0.66112600
C	-7.53832400	0.76319600	-1.56376200
H	-7.45475400	-0.32378800	-1.72282900
H	-8.51306800	0.98309800	-1.11961600
H	-7.46864200	1.26980900	-2.53960500
C	3.78587300	-2.35815400	1.39277700
H	4.13880900	-3.28820100	0.91931200
H	2.77774100	-2.57925900	1.77250800
C	4.71019000	-1.98930400	2.55129700
H	4.39982900	-1.03932500	3.00359000
H	4.70027200	-2.76761300	3.32420800
H	5.74745900	-1.86946800	2.21105700
C	0.64066600	-0.48968300	3.29453900
C	-0.75245000	-0.49835200	3.19801000
C	1.23669000	-0.75120200	4.53448700
C	-1.52954200	-0.75739100	4.32345900
H	-1.23811000	-0.28801300	2.24889600
C	0.46009100	-1.04550500	5.64592000
H	2.32183000	-0.71489800	4.60988800
C	-0.92858600	-1.04116400	5.54498400
H	-2.61544100	-0.74034700	4.23737100
H	0.93969900	-1.26612800	6.59800600
H	-1.54151200	-1.25506700	6.41917500
H	0.74289600	-1.75285000	0.90849400
C	0.88754800	-1.49968600	-2.68739200
C	1.20062400	-2.95962600	-3.07613500
O	0.45433400	-1.43525400	-1.46038900
O	1.09801800	-0.57571800	-3.45724200
C	2.36854800	-3.42818300	-2.20426900
H	3.24262500	-2.76964100	-2.32858400
H	2.08730900	-3.42900700	-1.14211200
H	2.66999400	-4.44835200	-2.48541400
C	1.59073600	-3.02423000	-4.54613100
H	0.77480700	-2.66893200	-5.18831800

H	2.46392100	-2.39279200	-4.74928400
H	1.83098200	-4.06013500	-4.82765000
C	-0.01631100	-3.84333900	-2.81379400
H	-0.89171200	-3.49233900	-3.38042400
H	0.18966500	-4.88022500	-3.11862200
H	-0.27313100	-3.84072600	-1.74634400

B

SCF Energy (a.u.) = -2616.70413

Thermal correction to Gibbs free Energy (a.u.) = 0.765071

Charge = 0, Multiplicity = 1

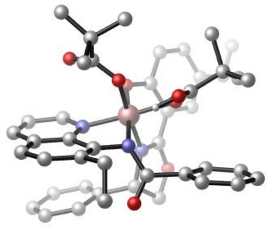

B

Co	-0.00425100	0.17501800	-0.42302300
O	2.19440600	-0.65255600	2.85802700
O	-1.13356500	1.05250900	-1.65686100
O	-2.11817900	2.70074300	2.01239300
N	1.48036000	1.25542800	-0.88944900
N	1.30787100	-0.81216900	0.73726500
N	-0.59670800	1.45110800	0.95740600
C	1.44073900	2.33587200	-1.63597900
H	0.44867500	2.71792000	-1.86632700
C	2.61679000	2.92002300	-2.13838700
H	2.54590000	3.82738500	-2.73156400
C	3.81913300	2.29701600	-1.91815800
H	4.74149300	2.69908200	-2.33773500
C	3.87437100	1.11252200	-1.15191100
C	2.66105200	0.64092200	-0.60494800
C	2.59086800	-0.50531200	0.21789800
C	3.73574600	-1.27248400	0.38080600
C	4.95009400	-0.80579400	-0.18286700
H	5.84239300	-1.41645600	-0.03637100
C	5.04253700	0.35933500	-0.90204700
H	5.99409600	0.69395100	-1.31347000

C	-2.37692300	1.29435600	-1.34262800
C	-3.38222400	1.14069300	-2.31782900
H	-3.07436400	0.77998700	-3.29749300
C	-4.70850400	1.43727200	-2.05142800
H	-5.44450000	1.29578800	-2.84047700
C	-5.10068500	1.90063200	-0.78582200
C	-4.13841900	2.06248500	0.19595400
H	-4.44088600	2.41969900	1.17802800
C	-2.79570000	1.76157200	-0.06685200
C	-1.79930800	1.94547200	0.95118700
C	-1.01841300	2.61604400	2.93497400
H	-1.32384200	1.96831400	3.76693200
H	-0.81121400	3.62212300	3.31191700
C	0.13180200	2.01836300	2.11508400
H	0.61710800	1.21103100	2.67513200
C	1.18905000	3.01370100	1.70602000
C	0.83504200	4.23511100	1.12752500
H	-0.21772000	4.47700500	0.96708400
C	1.81626800	5.14121800	0.74476900
H	1.53152300	6.09130400	0.29516400
C	3.16180300	4.82942700	0.92998100
H	3.93176400	5.53574300	0.62296100
C	3.51902800	3.61490800	1.50589300
H	4.56908000	3.36156700	1.64594200
C	2.53575900	2.71073300	1.89973200
H	2.81048500	1.74844500	2.33846300
C	-1.51933700	-2.23297800	-0.04405500
C	-2.82322600	-2.98229500	-0.15620500
O	-0.43863700	-2.96364400	-0.00700500
O	-1.51692300	-0.99647600	-0.03995600
C	-2.96402400	-3.30975100	-1.65115300
H	-2.90277100	-2.39396800	-2.25802000
H	-2.18154500	-4.00180100	-1.98754700
H	-3.94170900	-3.77838300	-1.82822300

C	-3.97491700	-2.07801900	0.27254700
H	-3.84720000	-1.73143600	1.30716900
H	-4.04963600	-1.19341300	-0.37287400
H	-4.91821300	-2.63675700	0.20987200
C	-2.78797400	-4.26181700	0.67458300
H	-2.63830800	-4.03750700	1.74022100
H	-3.74270700	-4.79381700	0.56551000
H	-1.98137400	-4.92969600	0.35142600
C	1.26805200	-0.95680900	2.10573500
O	-6.38295200	2.20761900	-0.43644800
C	-7.37834200	2.03385000	-1.40731700
H	-7.45243300	0.98453900	-1.73556400
H	-8.32277200	2.32653300	-0.94020400
H	-7.20556100	2.66883500	-2.29102400
C	3.74277100	-2.60660800	1.07341700
H	4.18097400	-3.34104700	0.37726000
H	2.70927300	-2.94133800	1.24806100
C	4.53022000	-2.62041200	2.38047200
H	4.10685400	-1.90027700	3.08851400
H	4.50905600	-3.61801900	2.83773800
H	5.58307500	-2.35736400	2.21040300
C	0.09143700	-1.64772200	2.75219900
C	-1.20057000	-1.13694800	2.87574400
C	0.39584300	-2.86013500	3.38193500
C	-2.16364300	-1.82299400	3.61328500
H	-1.47154000	-0.20665500	2.38427600
C	-0.57261300	-3.56084200	4.08784300
H	1.41433600	-3.24436600	3.32153300
C	-1.85759100	-3.03835100	4.21529900
H	-3.16206800	-1.39781600	3.71384900
H	-0.31817500	-4.50988100	4.55685500
H	-2.61475500	-3.57438300	4.78548100
H	0.34526200	-2.34545600	-0.03969700
C	1.00843800	-0.90105000	-2.91231100

C	1.44148400	-2.23156800	-3.56155800
O	0.47475500	-1.10086500	-1.73673300
O	1.22142800	0.16867500	-3.45814700
C	2.59140500	-2.79401400	-2.72113700
H	3.42405700	-2.07637600	-2.65796300
H	2.25885200	-3.01862300	-1.69788200
H	2.97579400	-3.72188400	-3.17011800
C	1.92195800	-1.97097500	-4.98279800
H	1.12045200	-1.54412000	-5.59907200
H	2.75535700	-1.25880600	-4.99265400
H	2.25469800	-2.91138800	-5.44626500
C	0.28333900	-3.22636700	-3.57518700
H	-0.58654400	-2.81692900	-4.10956200
H	0.58582300	-4.15331100	-4.08483600
H	-0.03078500	-3.48176800	-2.55573600

C

SCF Energy (a.u.) = -2269.756352

Thermal correction to Gibbs free Energy (a.u.) = 0.623342

Charge = 0, Multiplicity = 1

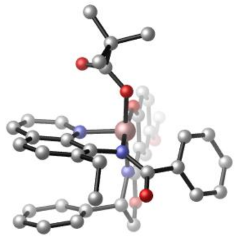

C

Co	0.16879200	0.58263300	0.32757400
O	-2.24984400	-1.47795900	2.75061100
O	1.73074800	1.47819300	-0.18112300
O	2.76702700	-2.59844400	0.22895300
N	-0.61231000	0.22483600	-1.31319900
N	-1.46769900	-0.08921800	1.06789200
N	1.07000200	-1.15260300	0.37131000
C	-0.01572800	0.37629200	-2.47786400
H	1.03764400	0.64540000	-2.45503200
C	-0.72976900	0.22852300	-3.67558800
H	-0.20934900	0.34832700	-4.62143800
C	-2.08106800	-0.01531800	-3.62120800
H	-2.66795200	-0.10242200	-4.53560300
C	-2.73480300	-0.14163500	-2.37840300
C	-1.94444900	-0.04025400	-1.21384700

C	-2.46822400	-0.18235400	0.10348200
C	-3.84909800	-0.29594900	0.24332200
C	-4.63348700	-0.39929500	-0.93664800
H	-5.71153400	-0.50453900	-0.80428500
C	-4.12117200	-0.35150600	-2.20709800
H	-4.76492000	-0.43513400	-3.08143900
C	2.92142800	1.00993400	-0.40496400
C	3.92699500	1.91449200	-0.81961900
H	3.63269400	2.95465200	-0.94382000
C	5.22433500	1.50865100	-1.05195700
H	5.95712200	2.24961800	-1.36530800
C	5.59381600	0.16083600	-0.88985800
C	4.63581300	-0.75121200	-0.49769700
H	4.92130200	-1.79329800	-0.37753200
C	3.30890400	-0.34902100	-0.25713600
C	2.33780900	-1.33177400	0.12760400
C	1.68788400	-3.36717100	0.77372300
H	1.90838100	-3.55212800	1.83383800
H	1.63495500	-4.31739700	0.23361700
C	0.45221700	-2.48155700	0.58864900
H	-0.12888500	-2.46878400	1.51605900
C	-0.46316100	-2.90950400	-0.53027300
C	-0.00186100	-3.00879400	-1.84425500
H	1.03635000	-2.76086100	-2.07608500
C	-0.86384400	-3.40038200	-2.86176300
H	-0.49869600	-3.46401500	-3.88591200
C	-2.19401400	-3.70112000	-2.57308400
H	-2.87053900	-3.99913600	-3.37283300
C	-2.65649900	-3.61282100	-1.26464900
H	-3.69829400	-3.83098400	-1.03288800
C	-1.79283700	-3.21943200	-0.24649800
H	-2.15857200	-3.11422900	0.77694400
C	-1.43808700	-0.67207300	2.29290700
O	6.85080600	-0.32458400	-1.09849500

C	7.84010300	0.58229900	-1.50334100
H	8.00293900	1.37416300	-0.75484700
H	8.76254300	0.00566900	-1.61439500
H	7.59584700	1.05301200	-2.46905100
C	-4.59727300	-0.26774000	1.54689500
H	-5.40043100	0.48102300	1.44747900
H	-3.94995800	0.07003200	2.36096300
C	-5.20468800	-1.62051600	1.90521900
H	-4.40723500	-2.35535200	2.06011000
H	-5.79087900	-1.55480500	2.83000200
H	-5.87116100	-1.98563300	1.11091300
C	-0.16427600	-0.35798900	3.02327500
C	0.54689700	0.83131200	2.80327300
C	0.40385100	-1.35851800	3.81197800
C	1.84625600	0.97257700	3.29516200
H	0.06354600	1.68026700	2.31366400
C	1.69416700	-1.20888300	4.30661000
H	-0.17672100	-2.26154900	3.99886900
C	2.42760400	-0.05365500	4.02895700
H	2.39540700	1.89089700	3.09361200
H	2.14009700	-2.00243700	4.90495900
H	3.44548600	0.04778100	4.40107400
C	-1.00027800	3.07755200	-0.50726100
C	-1.97248100	4.16122700	-0.00378900
O	-0.66379100	2.26512600	0.45497800
O	-0.69339300	2.99640400	-1.68745700
C	-3.31184100	3.45083500	0.22619500
H	-3.65643800	2.95417700	-0.69423100
H	-3.22020400	2.68720100	1.01140300
H	-4.08204500	4.17462900	0.53140500
C	-2.13435900	5.23989800	-1.06621000
H	-1.17883000	5.74040500	-1.27107300
H	-2.48903200	4.81196800	-2.01083500
H	-2.85584400	5.99779900	-0.72737500

C	-1.48653100	4.78036600	1.30396500
H	-0.50385200	5.25617700	1.17713800
H	-2.19303500	5.55328900	1.64116600
H	-1.39918500	4.02620000	2.09527800

TS2

SCF Energy (a.u.) = -2269.738069

Thermal correction to Gibbs free Energy (a.u.) = 0.621191

Charge = 0, Multiplicity = 1

Co	0.18034600	0.63036200	-0.07930500
O	-2.22164300	-0.23115300	2.94680300
O	1.72291000	1.12080500	-1.06954000
O	2.75027400	-2.31610800	1.17200900
N	-0.61611500	-0.42990500	-1.49028500
N	-1.47514000	0.33827100	0.81939600
N	1.05562000	-0.96286100	0.64550900
C	-0.04595800	-0.79506500	-2.61812600
H	1.01203700	-0.55397900	-2.71791300
C	-0.77509300	-1.43977300	-3.63206600
H	-0.27124100	-1.74169500	-4.54650400
C	-2.11982700	-1.65532000	-3.44644700
H	-2.71916400	-2.13848400	-4.21850300
C	-2.75191900	-1.23903500	-2.25552300
C	-1.94373100	-0.63299400	-1.26714800

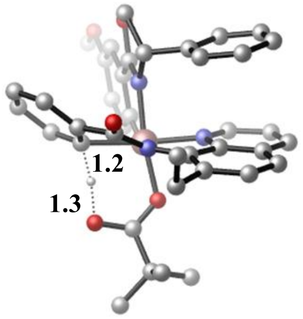

TS2

C	-2.46204300	-0.18712700	-0.01047000
C	-3.84103100	-0.24386400	0.18263100
C	-4.63485300	-0.85945600	-0.82148200
H	-5.71002300	-0.90780100	-0.64073300
C	-4.13354700	-1.36717400	-1.99155600
H	-4.78313100	-1.83049900	-2.73299100
C	2.93618300	0.67576600	-0.94737700
C	3.97497500	1.35056800	-1.63180800
H	3.69326900	2.22151200	-2.22071900
C	5.29060300	0.94005200	-1.56001800
H	6.04534000	1.50358800	-2.10535600
C	5.65090000	-0.18051800	-0.79140800
C	4.66496100	-0.86497000	-0.11169300
H	4.94330900	-1.73017400	0.48494500
C	3.31937400	-0.45844300	-0.17950700
C	2.32796700	-1.21060500	0.53635100
C	1.66412100	-2.76667200	1.98945200
H	1.86986100	-2.45576500	3.02306500
H	1.61649100	-3.85824700	1.93010700
C	0.43160800	-2.06038500	1.41716700
H	-0.15912400	-1.64392800	2.23990800
C	-0.47080300	-2.93740100	0.58713400
C	0.00381700	-3.58585200	-0.55422600
H	1.04232000	-3.44910700	-0.86321000
C	-0.84565200	-4.38481800	-1.31059200
H	-0.47008300	-4.87842500	-2.20595900
C	-2.17675800	-4.54540800	-0.92942200
H	-2.84363500	-5.16462500	-1.52784900
C	-2.65271700	-3.90840200	0.21157700
H	-3.69519000	-4.01661400	0.50873000
C	-1.80140700	-3.10798300	0.96773600
H	-2.17606400	-2.57779500	1.84568000
C	-1.02980200	3.14138400	-0.47779300
C	-2.08456300	3.97831500	-1.18921300

O	-0.61445500	3.50986300	0.65248300
O	-0.69646200	2.05650800	-1.03185300
C	-3.41613200	3.49003700	-0.59994800
H	-3.56304500	2.41784500	-0.80246800
H	-3.44647200	3.64385500	0.48730500
H	-4.25036100	4.04379900	-1.05339400
C	-2.06694100	3.72188600	-2.69178900
H	-1.10036400	3.99762000	-3.13384700
H	-2.24871700	2.66355600	-2.91632300
H	-2.84959300	4.32172000	-3.17736100
C	-1.88886100	5.45986200	-0.88966500
H	-0.93031100	5.82250100	-1.28508300
H	-2.69214800	6.04361100	-1.36062100
H	-1.90263200	5.65208600	0.18894000
C	-1.41504300	0.29680900	2.18082400
O	6.92585300	-0.65266800	-0.66187200
C	7.94289300	0.03546200	-1.33534500
H	8.02822100	1.08035900	-0.99550800
H	8.87605100	-0.48734300	-1.10731000
H	7.79047700	0.03128300	-2.42686300
C	-4.57718000	0.34253300	1.35617200
H	-5.38438900	0.97820400	0.95498200
H	-3.92110300	0.99353000	1.94108100
C	-5.17820200	-0.72320400	2.26727200
H	-4.37711500	-1.31402100	2.72413200
H	-5.76386900	-0.26442200	3.07339200
H	-5.84380300	-1.40002000	1.71283500
C	-0.11327200	0.86221200	2.65649400
C	0.70540800	1.52277300	1.71762300
C	0.34362700	0.57983700	3.93745800
C	2.00462500	1.88291900	2.09620500
H	0.10637500	2.45720700	1.06627800
C	1.64129700	0.94397900	4.29035500
H	-0.31393500	0.05131900	4.62724200

C	2.47217800	1.59123900	3.37350000
H	2.64579600	2.39987900	1.38129400
H	2.01404400	0.71780500	5.28878300
H	3.48497500	1.86840500	3.66250100

D

SCF Energy (a.u.) = -2269.760557

Thermal correction to Gibbs free Energy (a.u.) = 0.625997

Charge = 0, Multiplicity = 1

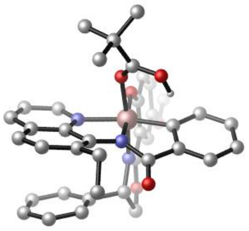

D

Co	0.29856400	0.58193600	-0.05209600
O	-2.22904100	0.08432600	2.95080100
O	1.83976200	1.03300400	-1.05428200
O	2.89427200	-2.11499900	1.55015900
N	-0.46600500	-0.67496100	-1.44690800
N	-1.40312800	0.28955300	0.77830900
N	1.18616200	-0.86208300	0.85298400
C	0.12206600	-1.13384600	-2.52970100
H	1.17413000	-0.86960900	-2.64808900
C	-0.56732200	-1.90635800	-3.48193900
H	-0.04391800	-2.27820100	-4.35930300
C	-1.90349800	-2.15689600	-3.28231200
H	-2.47805300	-2.73691900	-4.00528800
C	-2.56162000	-1.65049200	-2.14058100
C	-1.78388000	-0.91712700	-1.21275900
C	-2.34171200	-0.36332900	-0.00994000
C	-3.72722600	-0.43663300	0.15341100
C	-4.48094800	-1.18640700	-0.78670600
H	-5.55805300	-1.24687600	-0.61951400
C	-3.94215800	-1.80196800	-1.88609400
H	-4.56193000	-2.36366500	-2.58394100
C	3.05499700	0.60863900	-0.90425000
C	4.08621400	1.20784400	-1.66680900
H	3.79836900	2.01239900	-2.34119500
C	5.40195800	0.80284300	-1.56974000

H	6.15055500	1.30319900	-2.18125800
C	5.77038700	-0.23246200	-0.69311900
C	4.79295800	-0.83849700	0.06864500
H	5.07840600	-1.63678900	0.74938600
C	3.44731600	-0.43859600	-0.02531000
C	2.46247100	-1.10444700	0.77873400
C	1.80695000	-2.49688700	2.40060500
H	2.01609700	-2.11602900	3.40823500
H	1.75685100	-3.59004100	2.42385000
C	0.57020900	-1.84301400	1.77013000
H	-0.00347000	-1.31364700	2.54029100
C	-0.34193000	-2.81613300	1.06621400
C	0.12114900	-3.57714300	-0.00894600
H	1.14906900	-3.45496900	-0.35706500
C	-0.72742200	-4.46807900	-0.65466400
H	-0.36147400	-5.04716100	-1.50159700
C	-2.04712100	-4.60858900	-0.22832600
H	-2.71594200	-5.29691600	-0.74331000
C	-2.51052600	-3.85920300	0.84718800
H	-3.54589300	-3.94735800	1.17471000
C	-1.65926200	-2.96763800	1.49392400
H	-2.02836200	-2.34714800	2.31253600
C	-1.37142700	2.87250200	-0.90265700
C	-2.37109400	3.38532600	-1.91400200
O	-1.45976000	3.42876700	0.28628800
O	-0.58288100	1.97291300	-1.17895500
C	-3.73445300	2.82642200	-1.47780500
H	-3.72592100	1.72548900	-1.46154500
H	-4.01456600	3.18460400	-0.47904800
H	-4.50295000	3.15140700	-2.19235700
C	-2.01909400	2.85395000	-3.29838800
H	-1.02643400	3.19369700	-3.61958900
H	-2.01681200	1.75615700	-3.31504300
H	-2.76083400	3.21115500	-4.02518300

C	-2.40370100	4.91315600	-1.91029400
H	-1.43072100	5.33493500	-2.19633200
H	-3.14880200	5.26222200	-2.63776300
H	-2.67340000	5.30923900	-0.92470300
C	-1.43140300	0.52944700	2.12605500
O	7.04650600	-0.69375800	-0.53275100
C	8.05518200	-0.08302200	-1.28743100
H	8.14698700	0.99152500	-1.06007400
H	8.99066600	-0.58184000	-1.01842400
H	7.88851300	-0.20057900	-2.37065400
C	-4.52053600	0.27699900	1.21571900
H	-5.34545000	0.80469000	0.70722300
H	-3.91450300	1.04211300	1.71060900
C	-5.10270700	-0.66886200	2.26107600
H	-4.29264700	-1.14467500	2.82335000
H	-5.73652800	-0.12588400	2.97323900
H	-5.71711200	-1.45199800	1.79404300
C	-0.28015700	1.40415300	2.49641600
C	0.67172400	1.64179600	1.48987500
C	-0.15339800	1.96685900	3.76517900
C	1.75089600	2.47828700	1.76519100
H	-0.81260100	2.98315900	0.88554500
C	0.92749600	2.80090700	4.02834200
H	-0.90904200	1.74414000	4.51841600
C	1.86923400	3.05803100	3.03061600
H	2.50193000	2.67447900	0.99880800
H	1.03933400	3.25799800	5.01046300
H	2.71152600	3.71663800	3.24310800

E

SCF Energy (a.u.) = -1922.806173

Thermal correction to Gibbs free Energy (a.u.) = 0.486921

Charge = 0, Multiplicity = 1

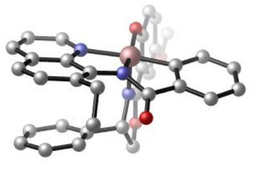

E

Co	0.19631100	0.56466800	-0.96192300
O	-2.67055600	2.60911600	0.71498700
O	1.82167100	0.05465000	-1.72238300
O	2.09706600	-0.31844400	2.45463600
N	-0.68518100	-1.21971700	-1.32103400
N	-1.59964500	1.12542500	-0.73682800
N	0.71627800	0.14302300	0.77727200
C	-0.09795100	-2.37635800	-1.56136000
H	0.98965200	-2.34938400	-1.64273700
C	-0.82595600	-3.56743900	-1.71080100
H	-0.30334700	-4.50194100	-1.89862700
C	-2.19809500	-3.51406100	-1.62637400
H	-2.80103700	-4.41433700	-1.74956600
C	-2.85049900	-2.28680600	-1.39222900
C	-2.03981200	-1.14028500	-1.21368800
C	-2.58451200	0.15675200	-0.93623800
C	-3.97122200	0.30140300	-1.01359200
C	-4.76240500	-0.86457600	-1.19359900
H	-5.84441100	-0.72444400	-1.22382300
C	-4.25225600	-2.12580800	-1.34802900
H	-4.90109000	-2.99055100	-1.48130500
C	2.93705600	-0.23956300	-1.12210100
C	4.10227800	-0.45546700	-1.89015500
H	4.01427300	-0.35144000	-2.96989100
C	5.31142600	-0.77897600	-1.30635600
H	6.17744000	-0.93051600	-1.94771700
C	5.42591700	-0.90460400	0.08891400
C	4.30705800	-0.69836500	0.87047000
H	4.39502100	-0.79076400	1.95038200
C	3.06939300	-0.37327000	0.28851100
C	1.92862600	-0.16874400	1.13450500

C	0.89266200	0.12896500	3.09704900
H	1.10071400	1.10248500	3.55693200
H	0.63039800	-0.59758300	3.87216800
C	-0.14803400	0.21901300	1.97016900
H	-0.64741500	1.19657400	1.98989900
C	-1.18749000	-0.87250000	2.00372600
C	-0.80527900	-2.21404800	1.93708600
H	0.25072500	-2.47320600	1.83393900
C	-1.76315000	-3.21934300	1.98258900
H	-1.45681500	-4.26271300	1.92075300
C	-3.11289000	-2.89087900	2.09695700
H	-3.86512200	-3.67806900	2.12279200
C	-3.49826700	-1.55663500	2.16433500
H	-4.55258100	-1.29123100	2.23256600
C	-2.53801600	-0.54955000	2.11810900
H	-2.83813800	0.49981100	2.13367200
C	-1.71395500	2.27640000	0.02233500
O	6.57953000	-1.22076300	0.74459200
C	7.73243600	-1.41826700	-0.02709900
H	8.00667200	-0.51485200	-0.59511300
H	8.53831100	-1.65353300	0.67359400
H	7.61706800	-2.25768200	-0.73160200
C	-4.73047700	1.60151100	-0.98286300
H	-5.40265700	1.60538000	-1.85680400
H	-4.05825100	2.45469500	-1.09927200
C	-5.56108100	1.77641100	0.28487600
H	-4.90189100	1.83601300	1.15731700
H	-6.15099600	2.70041900	0.24179300
H	-6.26037300	0.94013100	0.42769200
C	-0.44716500	3.04925500	-0.05206700
C	0.64836400	2.37228200	-0.59865500
C	-0.34041000	4.36156400	0.39733400
C	1.86828300	3.02749300	-0.71054900
C	0.88028000	5.01827700	0.27220900

H	-1.21535700	4.84782300	0.82882100
C	1.97303000	4.35488300	-0.28441700
H	2.73907000	2.51947000	-1.12628100
H	0.98418700	6.04890700	0.60849000
H	2.92638400	4.87489900	-0.38071000

2a

SCF Energy (a.u.) = -614.645252

Thermal correction to Gibbs free Energy (a.u.) = 0.165955

Charge = 0, Multiplicity = 1

C	1.93853800	-1.37055100	1.37160100
C	2.36334000	-2.24773900	0.37640400
C	1.84677500	-2.13960800	-0.90966100
C	0.90765000	-1.15530800	-1.20087100
C	0.49083400	-0.26664200	-0.21466500
C	1.00773500	-0.38352800	1.07678300
H	2.33664100	-1.45688000	2.38141400
H	3.09448800	-3.02082200	0.60747500
H	2.16690100	-2.83042700	-1.68777000
H	0.47879100	-1.08207100	-2.20025400
H	0.66841800	0.30639100	1.84956600
C	-0.52308500	0.79779600	-0.52927600
H	-0.87426500	0.66780800	-1.56381900
O	-1.67841900	0.65811700	0.33265300
C	-2.57947600	-0.27380900	-0.04309600
O	-2.49635600	-0.92284700	-1.05610200
C	-3.67367500	-0.37861600	0.97627700
H	-3.26754100	-0.79251600	1.90688500
H	-4.07711700	0.61205100	1.21170200
H	-4.46435200	-1.03214600	0.60157100
C	-0.04601800	2.20897300	-0.33339700
H	-0.82344500	2.97442100	-0.39270400
C	1.19715900	2.56287000	-0.14174000
C	2.43654400	2.91857200	0.04263700

H	2.85982700	3.01147800	1.04243800
H	3.10191500	3.12608300	-0.79488500

F

SCF Energy (a.u.) = -2537.482951

Thermal correction to Gibbs free Energy (a.u.) = 0.680310

Charge = 0, Multiplicity = 1

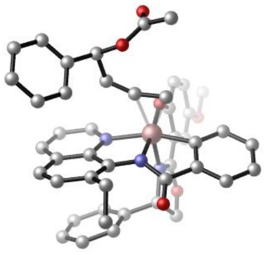

F

C	-5.70747200	0.53222300	-0.72546500
C	-6.60975200	1.19252000	-1.55579800
C	-6.29310600	2.44854400	-2.06388500
C	-5.07573800	3.04029600	-1.74229600
C	-4.16932200	2.38461500	-0.91197600
C	-4.49298700	1.12516100	-0.40414300
H	-5.94537800	-0.45562400	-0.33152500
H	-7.55980400	0.72505700	-1.81045500
H	-6.99391200	2.96770600	-2.71581000
H	-4.82172600	4.02344400	-2.14057400
H	-3.77913600	0.60069800	0.23480500
C	-2.85463300	3.03537000	-0.56450900
H	-2.65680400	3.87626200	-1.24687200
O	-2.96900500	3.56048600	0.76458500
C	-2.20201100	4.63356000	1.05754200
O	-1.47272200	5.17890200	0.26726500
C	-2.39658200	5.02742600	2.49099100
H	-2.04752600	4.22094100	3.14773500
H	-1.83643400	5.94101600	2.70168200
H	-3.46088900	5.17604200	2.70447800
C	-1.72499200	2.04348500	-0.65795400
H	-1.66419500	1.52057200	-1.61125100
C	-0.93198700	1.77397000	0.34865400
C	-0.37789000	1.90341700	1.56842600
H	0.38167700	2.66833700	1.73682900
H	-0.84656700	1.46214900	2.44654200
Co	0.60376800	0.30621600	0.51322800

O	-0.83672500	-2.47225700	2.95625400
O	1.69045700	1.66701400	-0.25490000
O	4.06085000	-1.83877900	-0.10784200
N	-0.17079000	-0.32212800	-1.23278400
N	-0.68807500	-0.91310700	1.23366100
N	2.00254100	-1.05698300	0.25503100
C	0.23639600	-0.01545700	-2.44795800
H	1.10156000	0.64579300	-2.50954300
C	-0.43055800	-0.48241300	-3.59393600
H	-0.06134300	-0.21254100	-4.58003900
C	-1.55865100	-1.25254300	-3.43347400
H	-2.11982900	-1.60844100	-4.29781200
C	-2.01921900	-1.58121400	-2.14101300
C	-1.25930000	-1.11650900	-1.04149800
C	-1.61374900	-1.40220400	0.31727000
C	-2.84084100	-2.02611500	0.54788400
C	-3.58444500	-2.48511700	-0.57153200
H	-4.52764100	-2.99414200	-0.36276700
C	-3.19906400	-2.30962100	-1.87438900
H	-3.80460900	-2.67800800	-2.70127900
C	2.92744400	1.61210100	-0.64899700
C	3.52761600	2.79370300	-1.14342500
H	2.91010000	3.68988400	-1.16403400
C	4.83615100	2.82479000	-1.58133300
H	5.24253900	3.76416400	-1.95131700
C	5.63057500	1.66614900	-1.54448500
C	5.07879700	0.49590300	-1.06490400
H	5.69509200	-0.39912100	-1.03285700
C	3.74263000	0.44801800	-0.62674700
C	3.21188200	-0.79739500	-0.14463000
C	3.40207700	-2.88485400	0.61679600
H	3.80853800	-2.89531400	1.63704800
H	3.61673800	-3.83612800	0.12059900
C	1.92152400	-2.49465000	0.58865900

H	1.48969500	-2.61567500	1.58868500
C	1.09744500	-3.28517800	-0.39722800
C	1.41427800	-3.28757600	-1.75731800
H	2.25555600	-2.69422800	-2.12113900
C	0.65363400	-4.02889900	-2.65389400
H	0.90329300	-4.01656200	-3.71415300
C	-0.43033800	-4.77719200	-2.19856100
H	-1.03249500	-5.34939000	-2.90306500
C	-0.74644100	-4.78304700	-0.84439400
H	-1.60386600	-5.34820000	-0.48035300
C	0.01678000	-4.04202400	0.05326000
H	-0.24856900	-4.01695600	1.11190300
C	-0.36364300	-1.45413200	2.45020200
O	6.93187900	1.60692500	-1.95433800
C	7.51422300	2.78746200	-2.43258200
H	7.52768400	3.57967900	-1.66664200
H	8.54463700	2.53909200	-2.70218300
H	6.99515400	3.17025700	-3.32631000
C	-3.48860200	-2.20705300	1.89445100
H	-4.51164700	-1.79830300	1.82441000
H	-2.96706800	-1.62532300	2.65922200
C	-3.56128600	-3.66743500	2.32884100
H	-2.54875700	-4.06204700	2.46408100
H	-4.09580800	-3.76721600	3.28172100
H	-4.08566100	-4.28431100	1.58507900
C	0.74213200	-0.68111500	3.07387700
C	1.32548100	0.33033300	2.30460100
C	1.23793800	-0.99243200	4.33910200
C	2.40113100	1.05044000	2.80875300
C	2.31297200	-0.27218100	4.84555200
H	0.76878700	-1.80187900	4.89834200
C	2.88890000	0.74365700	4.08029600
H	2.86551800	1.84095900	2.21704800
H	2.70868100	-0.49897300	5.83429500

H	3.73468000	1.30498400	4.47777100

TS3

SCF Energy (a.u.) = -2537.480041

Thermal correction to Gibbs free Energy (a.u.) = 0.679567

Charge = 0, Multiplicity = 1

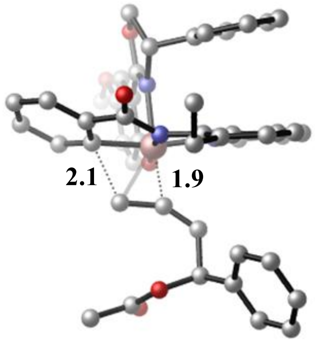

TS3

C	-5.76832100	0.59113400	-0.68649800
C	-6.70746100	1.30757000	-1.42410600
C	-6.38570800	2.56858600	-1.91689400
C	-5.12609300	3.10716600	-1.67523600
C	-4.18163300	2.39544200	-0.93827300
C	-4.51254600	1.13310500	-0.44233400
H	-6.01019000	-0.40099800	-0.30576600
H	-7.69014600	0.88042800	-1.61886700
H	-7.11519400	3.13185900	-2.49687300
H	-4.86693900	4.09241500	-2.06525200
H	-3.77244600	0.56712600	0.12730300
C	-2.82028200	2.99131400	-0.68138600
H	-2.62817200	3.80742100	-1.39484200
O	-2.84181000	3.56240700	0.63837700
C	-2.06494000	4.64607100	0.84319700
O	-1.37619700	5.15876900	-0.00410700
C	-2.18489700	5.10313400	2.26721800
H	-1.79619700	4.32965900	2.94133300
H	-1.61976600	6.02737600	2.40646300
H	-3.23705800	5.25729900	2.53080200
C	-1.74061100	1.95060000	-0.79386500
H	-1.78096900	1.36533900	-1.71488200
C	-0.86214800	1.68658100	0.14709800
C	-0.29264100	1.98559200	1.37200700
H	0.38484000	2.84086900	1.41814700
H	-0.85807300	1.77697700	2.28097600
Co	0.52678700	0.32474100	0.31903900
O	-0.88698300	-2.13107300	3.08858800

O	1.64523000	1.55602300	-0.61272700
O	4.07527100	-1.85349500	0.15925200
N	-0.18883700	-0.50100700	-1.30544800
N	-0.76255400	-0.79353500	1.18694800
N	1.99002200	-1.06706200	0.32083500
C	0.23505100	-0.31376400	-2.53935600
H	1.10358300	0.33533600	-2.64955700
C	-0.42032800	-0.89151600	-3.64021100
H	-0.03747200	-0.72076700	-4.64302100
C	-1.55142700	-1.64182500	-3.42021400
H	-2.09991100	-2.08279400	-4.25284000
C	-2.03166100	-1.83792600	-2.10813800
C	-1.28821400	-1.26481300	-1.05078000
C	-1.66607400	-1.40314300	0.32201500
C	-2.88880200	-2.01205700	0.60211500
C	-3.61603900	-2.58622000	-0.47424400
H	-4.55988600	-3.07647900	-0.22741900
C	-3.21481000	-2.53736500	-1.78352700
H	-3.80893900	-2.98880800	-2.57682000
C	2.92310500	1.49144900	-0.85464500
C	3.54814300	2.62766600	-1.41868700
H	2.91591100	3.49332600	-1.60855900
C	4.89593500	2.65672000	-1.71790300
H	5.31748700	3.56276100	-2.14907000
C	5.70660700	1.53886800	-1.46331200
C	5.13102300	0.41235500	-0.91092900
H	5.76022800	-0.45108500	-0.70959400
C	3.75837600	0.36725100	-0.60829200
C	3.21514700	-0.83585600	-0.03334600
C	3.38098600	-2.84056900	0.93047200
H	3.73728000	-2.77088700	1.96722700
H	3.61883500	-3.82805200	0.52255600
C	1.90136300	-2.45829400	0.80204800
H	1.42952200	-2.47569200	1.79215100

C	1.10441900	-3.34401800	-0.12308700
C	1.45342300	-3.47482900	-1.46867500
H	2.30891400	-2.92253500	-1.86310600
C	0.70551100	-4.28870100	-2.31157900
H	0.97941300	-4.37645400	-3.36226000
C	-0.39696600	-4.98242900	-1.81554900
H	-0.98898400	-5.61134200	-2.47920000
C	-0.74472100	-4.86123400	-0.47457100
H	-1.61615700	-5.38401200	-0.08144600
C	0.00511700	-4.04617000	0.36853600
H	-0.28444600	-3.91776700	1.41325800
C	-0.43696500	-1.17313300	2.45861200
O	7.04633000	1.47745500	-1.72256800
C	7.65228700	2.61746900	-2.26478100
H	7.56314500	3.48873300	-1.59574200
H	8.71150500	2.37650600	-2.39210600
H	7.22730300	2.88277800	-3.24649600
C	-3.54597000	-2.06144900	1.95475000
H	-4.57602900	-1.68371700	1.83597700
H	-3.04232700	-1.39092500	2.65622400
C	-3.59116300	-3.46914900	2.54027800
H	-2.57215600	-3.82324400	2.72881800
H	-4.13720100	-3.48150100	3.49164000
H	-4.08935300	-4.17340400	1.85862000
C	0.65990000	-0.31250900	2.98149900
C	1.15355600	0.70152000	2.14843800
C	1.27970400	-0.59097200	4.19718600
C	2.26158300	1.44486000	2.54399900
C	2.38811100	0.15018400	4.59123500
H	0.88297900	-1.40551500	4.80295900
C	2.87725300	1.16014400	3.76097300
H	2.65202700	2.23513100	1.90135700
H	2.87771800	-0.05861700	5.54095400
H	3.75186000	1.73474200	4.06448300

G

SCF Energy (a.u.) = -2537.516027

Thermal correction to Gibbs free Energy (a.u.) = 0.681997

Charge = 0, Multiplicity = 1

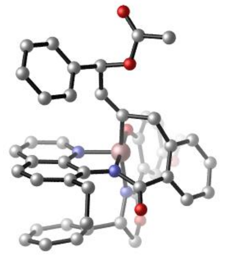

G

C	5.51448900	-0.04312700	-0.53945800
C	6.42442800	-0.43334400	-1.52019700
C	6.19656400	-1.59230500	-2.25464100
C	5.05993800	-2.35861800	-2.01219600
C	4.14194300	-1.96724400	-1.04150500
C	4.37823300	-0.80553900	-0.30254700
H	5.68594700	0.86656800	0.03641500
H	7.31275000	0.16767700	-1.71006900
H	6.90849000	-1.90485700	-3.01722800
H	4.88447700	-3.27801700	-2.57037800
H	3.65171200	-0.49885300	0.45425500
C	2.88837100	-2.77384700	-0.78628200
H	2.82497500	-3.58100800	-1.53182500
O	2.96313600	-3.38317000	0.51729300
C	3.66665200	-4.52545700	0.61331300
O	4.19341000	-5.08044700	-0.31983000
C	3.69643800	-4.99183200	2.03928600
H	2.68260100	-5.02100700	2.45579400
H	4.15758400	-5.98035200	2.09648000
H	4.27362100	-4.28270900	2.64491300
C	1.67966900	-1.88669300	-0.83977700
H	1.60012900	-1.37364000	-1.80339300
C	0.81782100	-1.58766700	0.12405600
C	0.66528000	-2.09831200	1.52208700
H	0.60803000	-3.19568500	1.54451800
H	1.53037500	-1.81236200	2.14062000
Co	-0.55877000	-0.29225000	0.12282000
O	0.61662300	1.67981000	3.30204500
O	-1.63194100	-1.46991300	-0.90959300

O	-4.31048100	1.56977600	0.45296400
N	0.16488100	0.65376700	-1.31325200
N	0.67545100	0.72886700	1.17456000
N	-2.15689200	0.96038500	0.40781100
C	-0.22902200	0.55345100	-2.57042800
H	-1.05586800	-0.13015300	-2.75355900
C	0.41626700	1.26481000	-3.59507300
H	0.06384300	1.15937000	-4.61768100
C	1.49439300	2.05969100	-3.28480700
H	2.02843200	2.60512000	-4.06286100
C	1.92955800	2.17498300	-1.94807200
C	1.21098700	1.45899500	-0.96508800
C	1.54275000	1.50496400	0.42157100
C	2.69395900	2.19359900	0.79825100
C	3.39515000	2.91797800	-0.20032100
H	4.28115700	3.47010600	0.11999100
C	3.03830800	2.94043600	-1.52411400
H	3.61226700	3.50660900	-2.25605900
C	-2.93089300	-1.52882500	-0.99590500
C	-3.49652200	-2.66595400	-1.61865400
H	-2.80169500	-3.42325300	-1.97785300
C	-4.85924700	-2.83111300	-1.76845800
H	-5.22876300	-3.73290900	-2.25302900
C	-5.74917200	-1.85246900	-1.29843100
C	-5.23551700	-0.72554400	-0.68839100
H	-5.92595600	0.03172000	-0.32492700
C	-3.85013100	-0.54514900	-0.53050600
C	-3.38009200	0.65476400	0.11393700
C	-3.64306100	2.58385800	1.21150300
H	-3.94343900	2.47391800	2.26237200
H	-3.96606400	3.56227600	0.84022100
C	-2.14637600	2.30601600	0.99995300
H	-1.62738700	2.27272700	1.96772500
C	-1.43343400	3.30783200	0.12761200

C	-1.84054800	3.53030700	-1.18931700
H	-2.68521800	2.96991200	-1.59589300
C	-1.16556000	4.44774600	-1.98617400
H	-1.48551000	4.61066400	-3.01451500
C	-0.07434700	5.14811800	-1.47414200
H	0.46074700	5.85861300	-2.10276500
C	0.33356800	4.93267800	-0.16221800
H	1.19535000	5.46333400	0.24112400
C	-0.34567900	4.01591100	0.63496900
H	-0.01384700	3.81851200	1.65638700
C	0.32503200	0.81180200	2.48065000
O	-7.10976000	-1.92913900	-1.39802400
C	-7.64846200	-3.06358600	-2.01707900
H	-7.38871600	-3.98964600	-1.47867200
H	-8.73513200	-2.94008600	-2.00320100
H	-7.31445500	-3.15996500	-3.06299900
C	3.28248800	2.19652600	2.18224800
H	4.36224800	1.99314600	2.08624200
H	2.85943300	1.38772000	2.78626200
C	3.08254700	3.52540400	2.90428200
H	2.01478600	3.69299700	3.08394500
H	3.59170700	3.52572000	3.87586000
H	3.47960900	4.36478900	2.31542300
C	-0.67759500	-0.27123100	2.77463600
C	-0.61408400	-1.54270100	2.15495600
C	-1.78691600	0.07601600	3.54841800
C	-1.74333600	-2.37208300	2.24327900
C	-2.88114600	-0.77088000	3.63426900
H	-1.77543700	1.04229600	4.05234100
C	-2.87305300	-1.98666100	2.94789300
H	-1.71639400	-3.33880200	1.73839700
H	-3.75248400	-0.48040100	4.21934000
H	-3.74294300	-2.64002400	2.97914100

TS4

SCF Energy (a.u.) = -2537.485276

Thermal correction to Gibbs free Energy (a.u.) = 0.680819

Charge = 0, Multiplicity = 1

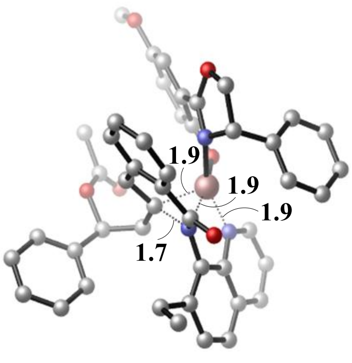

TS4

C	-4.05341500	3.98781300	0.13445300
C	-3.75085100	5.15157600	0.83635400
C	-2.44410500	5.62549200	0.83890900
C	-1.44684500	4.94596900	0.14421100
C	-1.74011000	3.77267600	-0.55211500
C	-3.05644600	3.30205300	-0.54834800
H	-5.07526300	3.60866600	0.12204000
H	-4.53043000	5.68417900	1.37886000
H	-2.19386800	6.53350100	1.38622200
H	-0.42571600	5.31979900	0.14908000
H	-3.29014300	2.37875300	-1.08237700
C	-0.69863900	3.01788300	-1.35380700
H	-0.82104000	3.27333800	-2.41696700
O	0.59824500	3.53340800	-0.95799900
C	1.60902400	3.33727800	-1.82358200
O	1.47864200	2.87322400	-2.93029300
C	2.90548200	3.76939400	-1.20354000
H	3.22492400	3.01058800	-0.47474500
H	3.67431100	3.85333800	-1.97638700
H	2.78932700	4.71833600	-0.66915200
C	-0.77725700	1.52265600	-1.24903800
H	-0.97098000	1.00809300	-2.18564200
C	-0.52820400	0.82013800	-0.11049900
C	-0.08480200	1.52317400	1.14022500
H	0.98700300	1.75387100	1.02126100
H	-0.57740800	2.51070700	1.18176900
Co	0.15745400	-0.96218300	-0.42862000
O	-2.64814300	-1.81935400	1.92134700
O	1.72417800	-0.87365800	-1.48261400
O	3.15324900	-1.57060800	2.22649800

N	-0.86189900	-1.41913200	-2.00386900
N	-1.62526800	-0.53459900	0.32833000
N	1.22814800	-1.25252300	1.13513600
C	-0.36846000	-1.89208100	-3.13341400
H	0.71882500	-1.92941800	-3.18542600
C	-1.19832000	-2.27977300	-4.20245300
H	-0.74776100	-2.67522200	-5.10912300
C	-2.55808900	-2.11533100	-4.09340200
H	-3.22240000	-2.37392800	-4.91813300
C	-3.10897200	-1.58804300	-2.90452400
C	-2.21044000	-1.29036500	-1.85527000
C	-2.64973500	-0.78142200	-0.61300500
C	-3.98333900	-0.43008700	-0.45915500
C	-4.87787900	-0.73297200	-1.51753800
H	-5.92854100	-0.47414500	-1.37839300
C	-4.47814200	-1.31388600	-2.69476100
H	-5.19646200	-1.53630900	-3.48306600
C	2.79049300	-0.24407300	-1.08326700
C	3.63319200	0.37908000	-2.03292800
H	3.30947100	0.36666900	-3.07142100
C	4.80780900	1.00787100	-1.66950700
H	5.40488900	1.48355000	-2.44561300
C	5.21828500	1.05522800	-0.32526100
C	4.43337100	0.44188200	0.63227400
H	4.75886800	0.44998500	1.67116000
C	3.23048400	-0.18596800	0.27417200
C	2.48932600	-0.96384400	1.22653100
C	2.18421100	-2.36881400	2.92975000
H	1.91763800	-1.84750900	3.86000400
H	2.64410800	-3.33485600	3.16100800
C	0.99220100	-2.44468300	1.97885600
H	0.05619900	-2.34142200	2.53682500
C	0.88287200	-3.63321000	1.05397900
C	2.00732200	-4.15293000	0.40912800

H	2.99988400	-3.76468100	0.64473700
C	1.86581700	-5.13759500	-0.56105500
H	2.74792800	-5.53389400	-1.06136300
C	0.59870600	-5.60763400	-0.90051400
H	0.48965200	-6.37506100	-1.66543300
C	-0.52499300	-5.09561200	-0.25968400
H	-1.51840600	-5.45717200	-0.52084200
C	-0.38414400	-4.11229200	0.71544600
H	-1.26736800	-3.69204600	1.20202900
C	-1.86940300	-0.91315400	1.64957400
O	6.35534500	1.67226700	0.11410700
C	7.09557400	2.40270500	-0.82379800
H	6.49151300	3.19799200	-1.29173600
H	7.92486300	2.86149800	-0.27787900
H	7.50666000	1.76003900	-1.61886700
C	-4.53366900	0.29045900	0.74025200
H	-5.09752300	1.16258400	0.37047000
H	-3.71825800	0.70987500	1.34723800
C	-5.45426200	-0.57186900	1.59857500
H	-4.91122100	-1.43678200	1.99306800
H	-5.85138000	0.00652300	2.44155300
H	-6.30831800	-0.93945200	1.01358800
C	-1.13287400	-0.19672100	2.72025900
C	-0.27773200	0.88218600	2.47876900
C	-1.29645300	-0.68609500	4.02389500
C	0.42159000	1.42843100	3.56019400
C	-0.60345800	-0.13070400	5.08528700
H	-1.97463800	-1.52556500	4.16530600
C	0.26839500	0.93301300	4.84599900
H	1.09933500	2.26264000	3.37379500
H	-0.73658100	-0.52032300	6.09279500
H	0.82936600	1.37694300	5.66718600

H

SCF Energy (a.u.) = -2537.506256

Thermal correction to Gibbs free Energy (a.u.) = 0.683697

Charge = 0, Multiplicity = 1

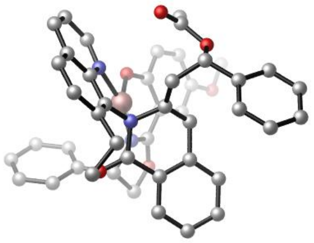

H

C	-4.64225200	3.50464200	1.81251200
C	-4.29960000	4.85298600	1.77889200
C	-3.51388100	5.33473300	0.73677900
C	-3.07125300	4.47660900	-0.26497500
C	-3.40120200	3.12107100	-0.23051600
C	-4.19506000	2.64522300	0.81570800
H	-5.25161900	3.11435400	2.62620300
H	-4.64136300	5.52535700	2.56414800
H	-3.23768700	6.38762100	0.70299700
H	-2.44391400	4.85220900	-1.07043800
H	-4.43951500	1.58337700	0.86233600
C	-2.93249800	2.17901500	-1.31626700
H	-3.67004600	2.16831000	-2.13288200
O	-1.70342100	2.70842300	-1.86144100
C	-1.23909500	2.06066500	-2.95668700
O	-1.89267100	1.25807700	-3.58054700
C	0.18823500	2.40480600	-3.21866100
H	0.79193400	1.64863400	-2.68809800
H	0.40427700	2.32459100	-4.28818200
H	0.46175100	3.39124800	-2.83314900
C	-2.69544300	0.75647600	-0.89341800
H	-3.19150200	-0.00455500	-1.48834500
C	-1.75032900	0.41266800	-0.01022200
C	-0.98640000	1.36561500	0.85559300
H	0.06712200	1.38016900	0.51842000
H	-1.36954700	2.38394100	0.74515200
Co	0.66266800	-0.72513900	-0.57573700
O	-1.09910900	-2.65216900	1.72930400
O	2.04016600	0.08635100	-1.61204200
O	3.43701800	0.18984200	2.29148900
N	-0.16258000	-1.36882400	-2.15747500

N	-1.36773600	-0.97621000	0.18007200
N	1.73677700	-0.53871200	1.01902300
C	0.40871700	-1.41149800	-3.35622900
H	1.34541100	-0.86210300	-3.43274900
C	-0.13670100	-2.11785500	-4.43965000
H	0.38787400	-2.10979400	-5.39189200
C	-1.31274600	-2.80727100	-4.27882100
H	-1.75914600	-3.37199900	-5.09663300
C	-1.96367200	-2.76760900	-3.02672400
C	-1.35411600	-2.02491300	-1.98901400
C	-1.99764700	-1.89924300	-0.73925100
C	-3.23099200	-2.47248800	-0.50109100
C	-3.79808800	-3.26270800	-1.53318600
H	-4.76439000	-3.73262600	-1.34634800
C	-3.19594000	-3.40541600	-2.75545700
H	-3.66852800	-3.99247900	-3.54267400
C	2.84357800	1.01780600	-1.19121500
C	3.47899200	1.86555300	-2.13090400
H	3.27101400	1.68527300	-3.18548300
C	4.32607600	2.88899200	-1.75179100
H	4.77055500	3.51422000	-2.52401800
C	4.61343900	3.11502700	-0.39544900
C	4.04949900	2.28289800	0.55093300
H	4.30064500	2.43011700	1.59946200
C	3.17017200	1.25325700	0.17767200
C	2.71623200	0.30634700	1.15619600
C	2.78140900	-0.81406600	3.07527300
H	2.15963600	-0.31236700	3.83327700
H	3.54770800	-1.42486300	3.56230500
C	1.92045700	-1.56745800	2.06223600
H	0.96646000	-1.84309400	2.51755700
C	2.53828500	-2.81489100	1.47910700
C	3.66365300	-2.75175500	0.65335000
H	4.09186800	-1.78516800	0.38403300

C	4.22850100	-3.91614200	0.14884300
H	5.10433100	-3.85502900	-0.49537200
C	3.67097500	-5.15574300	0.45509900
H	4.11233100	-6.06684200	0.05357700
C	2.54243400	-5.22408300	1.26430000
H	2.09115700	-6.18806500	1.49488400
C	1.97859800	-4.05723500	1.77200600
H	1.07620700	-4.10117500	2.38255200
C	-1.18724600	-1.45794700	1.51043200
O	5.43521400	4.10540600	0.06819800
C	6.04412000	4.93441100	-0.88016000
H	5.30406700	5.49483600	-1.47494000
H	6.66038900	5.64620800	-0.32313900
H	6.69099700	4.36743000	-1.56960200
C	-4.03513500	-2.25959000	0.75257100
H	-5.04639900	-1.94063600	0.45321400
H	-3.62815700	-1.42585000	1.34274900
C	-4.13124100	-3.51226500	1.61844000
H	-3.13444700	-3.82626400	1.94754000
H	-4.75110500	-3.32665400	2.50407400
H	-4.58265300	-4.34426200	1.06176100
C	-1.13771000	-0.43464100	2.57786700
C	-1.05155400	0.93124100	2.28448100
C	-1.17571100	-0.87387100	3.90509900
C	-1.01002100	1.84659300	3.33375800
C	-1.12752000	0.04503900	4.94157900
H	-1.24343700	-1.94415800	4.09347100
C	-1.04416800	1.40801200	4.65187400
H	-0.95965000	2.91155100	3.10503200
H	-1.16077200	-0.29426300	5.97506500
H	-1.01359600	2.13435000	5.46252300

I

SCF Energy (a.u.) = -1493.425404

Thermal correction to Gibbs free Energy (a.u.) = 0.432398

Charge = 0, Multiplicity = 1

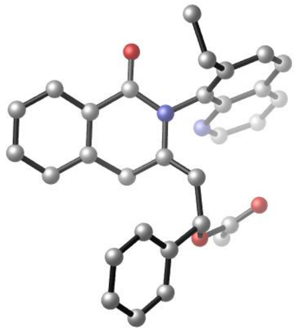

I

C	-4.59573100	-0.19912600	1.69991700
C	-5.51542600	-1.18408400	1.35996300
C	-5.06184700	-2.41368100	0.89076200
C	-3.69977800	-2.65316100	0.76131700
C	-2.76869500	-1.66592500	1.09310700
C	-3.23159000	-0.43922800	1.56895700
H	-4.93779900	0.76824300	2.06404700
H	-6.58316400	-0.99530400	1.45832300
H	-5.77420300	-3.19183700	0.62088000
H	-3.34828000	-3.61161500	0.38351700
H	-2.51440100	0.34113900	1.81996800
C	-1.28864500	-1.96286500	0.97376300
H	-0.99134100	-2.63478000	1.79291800
O	-1.12127500	-2.71930300	-0.25664600
C	0.08699000	-3.28796000	-0.43922500
O	0.97003200	-3.28716800	0.38265100
C	0.16138500	-3.90216500	-1.80531300
H	0.06207700	-3.11107700	-2.55931700
H	1.11962400	-4.41294800	-1.92741300
H	-0.66534300	-4.60434200	-1.95997700
C	-0.36006700	-0.78764700	0.98699400
H	0.41998900	-0.79400600	1.74604400
C	-0.31917000	0.14128700	0.02457000
C	-1.26247400	0.24871400	-1.12968000
H	-0.70792500	-0.08028900	-2.02696900
H	-2.11837300	-0.42315200	-1.01415000
O	1.51535100	3.14512200	-0.61956000
N	2.09018000	-0.71536000	-1.52171500
N	0.73119700	1.09193200	-0.00574500
C	2.71811200	-1.61608100	-2.24285600
H	2.23100800	-1.91451900	-3.17531200
C	3.95258900	-2.20221500	-1.89382800

H	4.40393100	-2.95140000	-2.54013300
C	4.55447400	-1.79779300	-0.73142900
H	5.50943500	-2.21510800	-0.41120500
C	3.93060000	-0.81861500	0.06930800
C	2.68012200	-0.30688300	-0.36796500
C	2.01758000	0.66412700	0.43898900
C	2.56792000	1.11337200	1.62000200
C	3.83072400	0.60108400	2.01618900
H	4.26034500	0.96079400	2.95188000
C	4.49225000	-0.33815100	1.27523900
H	5.45254900	-0.73515900	1.60447200
C	0.59019300	2.35276900	-0.57211800
C	1.88735600	2.11454100	2.50867400
H	1.91765200	1.73954300	3.54372600
H	0.82609100	2.19499900	2.23724200
C	2.55064900	3.48830900	2.44218700
H	2.52293100	3.87053700	1.41569800
H	2.03846700	4.20235100	3.09849200
H	3.60112900	3.43595700	2.75878600
C	-0.77533100	2.68222800	-1.05601100
C	-1.70690300	1.67016900	-1.30344500
C	-1.11171900	4.01917800	-1.25586400
C	-2.98883500	2.01252100	-1.72002900
C	-2.39071700	4.35349400	-1.67902900
H	-0.35313800	4.77653700	-1.06813500
C	-3.32965000	3.34877400	-1.90392600
H	-3.72221200	1.22434400	-1.89322300
H	-2.66054600	5.39700200	-1.83006700
H	-4.33721200	3.60896100	-2.22477600

J

SCF Energy (a.u.) = -1737.938098

Thermal correction to Gibbs free Energy (a.u.) = 0.500522

Charge = 0, Multiplicity = 1

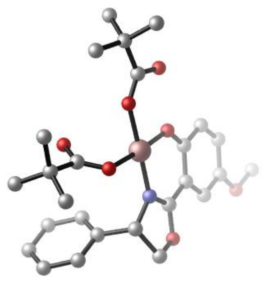

J

Co	0.13026700	0.51536100	-0.51711000
O	-1.49882600	1.36687100	-0.09075800
O	-2.33737900	-2.64736400	-1.06914400
N	-0.70070200	-1.13031000	-0.83844600
C	-2.70367400	0.86209200	-0.00088700
C	-3.77335700	1.68243800	0.40774600
H	-3.55264500	2.72249700	0.64764700
C	-5.06717000	1.20669900	0.50869800
H	-5.85312500	1.88650700	0.83110000
C	-5.35674600	-0.13099900	0.19741700
C	-4.33052100	-0.96108100	-0.21005400
H	-4.55854000	-1.99593600	-0.45386500
C	-3.00994100	-0.49083700	-0.30903100
C	-1.97429100	-1.38803100	-0.73668000
C	-1.19707700	-3.23809300	-1.69567700
H	-1.32949300	-3.17089100	-2.78501600
H	-1.13965100	-4.28905400	-1.39506800
C	-0.02480900	-2.38168800	-1.20274700
H	0.69304700	-2.18701500	-2.01107900
C	0.70670800	-3.02509400	-0.04600900
C	0.42584100	-2.71390300	1.28309900
H	-0.30437000	-1.93609700	1.50771500
C	1.08724900	-3.37631300	2.31439800
H	0.86503200	-3.11731000	3.34834900
C	2.02314700	-4.36541300	2.03025600
H	2.53590100	-4.88296800	2.83935900
C	2.30351100	-4.68825100	0.70413900
H	3.03615700	-5.45942600	0.47038100
C	1.65053700	-4.01775300	-0.32390100
H	1.87053600	-4.26782300	-1.36464000
O	-6.60175500	-0.68855700	0.26605300

C	-7.65779900	0.13624700	0.67161300
H	-7.80198000	0.98753000	-0.01345100
H	-8.55918500	-0.48320500	0.66021200
H	-7.50730200	0.52687200	1.69122900
C	2.72324400	-0.31963100	-0.00909200
C	4.14902700	-0.76602600	-0.22007400
O	2.42429500	0.03910100	1.22363400
O	1.90075400	-0.25154600	-0.92521800
C	4.99136300	0.51365400	-0.31124400
H	4.64859800	1.15248500	-1.13699800
H	4.93961100	1.09253200	0.62001100
H	6.04157000	0.24833500	-0.49468900
C	4.24955900	-1.54952500	-1.52363700
H	3.63078600	-2.45667800	-1.48147500
H	3.91645400	-0.95038700	-2.37895800
H	5.29162700	-1.85275300	-1.69150500
C	4.61911200	-1.62283800	0.95444900
H	3.99374400	-2.51969400	1.06684200
H	5.65381500	-1.94664200	0.77680400
H	4.58759200	-1.06628900	1.89794300
H	1.46516200	0.32993100	1.17963800
C	0.74236100	3.34356400	0.04084500
C	1.73424900	4.48027200	0.18007500
O	-0.50430800	3.66722600	0.25037100
O	1.10954100	2.20239300	-0.26341400
C	1.52149500	5.17155600	1.52894200
H	0.50512800	5.57136800	1.61812100
H	1.69203200	4.47787700	2.36382800
H	2.23256800	6.00282200	1.63138900
C	1.46801800	5.47311500	-0.95707800
H	1.60082600	4.99704200	-1.93828700
H	0.44903900	5.87490900	-0.90344500
H	2.17563500	6.31089300	-0.88806100
C	3.15598100	3.94125900	0.08495400

H	3.33637800	3.45968300	-0.88414900
H	3.87348800	4.76457200	0.20317400
H	3.35101900	3.19662100	0.86905700
H	-1.06642300	2.81959700	0.13368000

A′

SCF Energy (a.u.) = -2616.690722

Thermal correction to Gibbs free Energy (a.u.) = 0.764738

Charge = 0, Multiplicity = 1

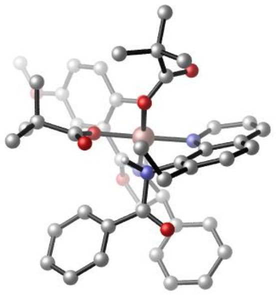

A′

Co	-0.13443400	0.40198100	-0.50543300
O	3.20289700	-1.63161500	0.85928000
O	-1.45173700	1.12656000	-1.61382100
O	-2.00149400	-2.72653600	-2.42003000
N	1.28969000	0.78352600	-1.74866700
N	1.48746300	-0.10144800	0.77268600
N	-0.54624200	-1.37068300	-1.37766400
C	1.14168300	1.09664600	-3.01688700
H	0.15689900	0.90272600	-3.44078100
C	2.17185800	1.70155800	-3.75902200
H	2.01643100	1.91619900	-4.81255800
C	3.31638800	2.09097000	-3.10803500
H	4.10546500	2.62531200	-3.63719900
C	3.46794000	1.83474400	-1.72783300
C	2.43751900	1.10981200	-1.08786200
C	2.51920300	0.78489300	0.28105600
C	3.55939800	1.25969600	1.05225300
C	4.56433200	2.02960600	0.41099500
H	5.38482800	2.40572200	1.02278700
C	4.54902600	2.28435500	-0.93664400
H	5.34972100	2.85451000	-1.40647000
C	-2.66017700	0.64077400	-1.47637400
C	-3.76670100	1.49947700	-1.37346400
H	-3.57206400	2.56999400	-1.34112600
C	-5.05938400	1.00244200	-1.31333000

H	-5.88690800	1.70363100	-1.22819600
C	-5.29955800	-0.37960900	-1.34990000
C	-4.22498400	-1.25142200	-1.45627100
H	-4.41348200	-2.32270300	-1.48930000
C	-2.92250900	-0.75261800	-1.50123200
C	-1.78675000	-1.61294100	-1.70232700
C	-0.73750000	-3.39115600	-2.50624300
H	-0.64809100	-3.84705700	-3.49559500
H	-0.71044900	-4.17510500	-1.73661000
C	0.26651400	-2.28028600	-2.23754400
H	0.43994700	-1.72062700	-3.17437100
C	1.60829600	-2.78058200	-1.77567800
C	1.74907400	-3.86776300	-0.90890400
H	0.87607400	-4.31712800	-0.43460000
C	3.00743300	-4.38154200	-0.61933200
H	3.09636000	-5.22449300	0.06445600
C	4.14377400	-3.81349000	-1.18339800
H	5.12845500	-4.21420200	-0.94939100
C	4.01820600	-2.72314500	-2.03749900
H	4.90207900	-2.26404000	-2.47671400
C	2.75902700	-2.22044600	-2.33661700
H	2.65903400	-1.37877000	-3.02474400
C	-1.34564100	0.33186600	2.08639400
C	-2.66860700	0.29364600	2.85764200
O	-0.28706700	0.55939300	2.69146500
O	-1.46794800	0.10274100	0.82545800
C	-3.20008800	1.72947000	2.85646700
H	-2.49928600	2.40739600	3.36274200
H	-3.34579400	2.08670600	1.82594900
H	-4.16684500	1.77591300	3.37890000
C	-2.40852100	-0.17479300	4.28527900
H	-2.00640600	-1.19873700	4.29517200
H	-1.68091700	0.47249800	4.78667600
H	-3.34556900	-0.16862700	4.86036500

C	-3.68234500	-0.62347400	2.18010100
H	-3.28315800	-1.64151500	2.05361700
H	-4.59112100	-0.69210900	2.79548300
H	-3.96342900	-0.25415800	1.18732800
C	2.03980300	-1.39460300	1.10206600
O	-6.53451600	-0.95076000	-1.28913500
C	-7.63619900	-0.10058500	-1.11725000
H	-7.75141600	0.59765800	-1.96145300
H	-8.51922100	-0.74348400	-1.06664500
H	-7.56138600	0.48020500	-0.18421600
C	3.63852900	1.02872300	2.53563200
H	4.69244300	1.08298800	2.84440100
H	3.30359200	0.01389700	2.78701700
C	2.81366100	2.04443200	3.32413100
H	1.74870500	1.97758800	3.06239400
H	2.90400800	1.86152600	4.40160600
H	3.15016500	3.07078600	3.12415900
C	1.20216300	-2.38901400	1.82853800
C	-0.18019500	-2.53254000	1.69621300
C	1.91143200	-3.29916100	2.62181000
C	-0.83678400	-3.57367300	2.34477200
C	1.25163600	-4.32616500	3.28012300
H	2.99199000	-3.19140700	2.69420900
C	-0.12706900	-4.46889100	3.13862600
H	-1.91412300	-3.68307800	2.22347900
H	1.81338500	-5.02110900	3.90193700
H	-0.64719600	-5.27924100	3.64722300
H	-0.74708400	-1.85335700	1.06726300
C	0.21863600	3.23205600	-0.34730800
C	0.24027600	4.43857500	0.61168000
O	0.07392700	2.10014100	0.28162500
O	0.42015400	3.40728200	-1.54078600
C	1.72440500	4.75258500	0.82269200
H	2.24869600	3.88891200	1.25796200

H	2.20768400	4.99488400	-0.13358500
H	1.84674100	5.60713400	1.50481600
C	-0.43633400	4.14611700	1.94413400
H	-1.50019200	3.91422200	1.79288700
H	0.01212500	3.28585000	2.45618300
H	-0.37080100	5.02579100	2.60213900
C	-0.44193000	5.62153100	-0.06807600
H	-1.51039800	5.41916000	-0.23002000
H	-0.35915600	6.52014400	0.56105800
H	0.01257000	5.82506500	-1.04370800
H	0.93309000	0.27715900	1.59337100

TS1′

SCF Energy (a.u.) = -2616.68656

Thermal correction to Gibbs free Energy (a.u.) = 0.760594

Charge = 0, Multiplicity = 1

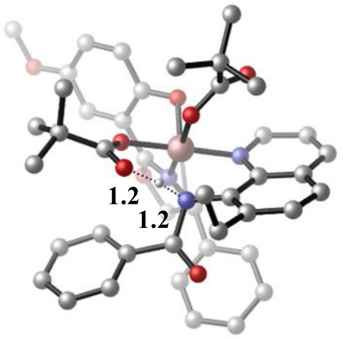

TS1′

Co	-0.08040500	0.41276300	-0.56386200
O	2.79817800	-2.09591400	1.12028100
O	-1.32611400	1.31148000	-1.64525000
O	-2.19735400	-2.39701800	-2.71501300
N	1.38444600	0.64237400	-1.77349800
N	1.38699500	-0.31281800	0.72409000
N	-0.66037400	-1.26616800	-1.53304800
C	1.29528700	1.02168000	-3.02953200
H	0.29438300	1.00617400	-3.45943100
C	2.41662700	1.47542000	-3.74586600
H	2.30654900	1.75438900	-4.78997100
C	3.60599700	1.64112900	-3.07900200
H	4.47730100	2.05432300	-3.58739800
C	3.70497700	1.31099900	-1.70996200
C	2.56294800	0.74909900	-1.09474100
C	2.56612100	0.36494800	0.26439800
C	3.66420100	0.65370600	1.05215000
C	4.79736500	1.24504600	0.43442200

H	5.66303300	1.45794300	1.06319600
C	4.84408200	1.53681700	-0.90452600
H	5.73686000	1.97058300	-1.35356700
C	-2.57186400	0.92887300	-1.51551700
C	-3.59791300	1.86845900	-1.31940700
H	-3.31166300	2.91390000	-1.21955800
C	-4.92726000	1.47943700	-1.25209300
H	-5.68803600	2.24061200	-1.09134700
C	-5.28964900	0.12970400	-1.37812800
C	-4.29908200	-0.82015800	-1.58209000
H	-4.58071600	-1.86612400	-1.68820500
C	-2.96011800	-0.43075600	-1.63116500
C	-1.90455100	-1.36830600	-1.90674900
C	-0.99457400	-3.16512400	-2.83944900
H	-0.90545400	-3.50567600	-3.87429400
H	-1.07346900	-4.03720500	-2.17617300
C	0.10379800	-2.20039200	-2.40485400
H	0.43304300	-1.60321900	-3.27298600
C	1.31948200	-2.88551800	-1.84652900
C	1.22589200	-3.95023200	-0.94706300
H	0.25559600	-4.25807300	-0.55658800
C	2.36906600	-4.60813500	-0.51222200
H	2.28033100	-5.42061600	0.20703200
C	3.62028900	-4.21488700	-0.97382000
H	4.51553000	-4.72506100	-0.62326200
C	3.72603600	-3.15610900	-1.86888800
H	4.70246200	-2.83464400	-2.22721100
C	2.58078900	-2.50231600	-2.30604200
H	2.66057300	-1.67890100	-3.01846300
C	-1.22513900	0.43934800	2.01594400
C	-2.44903400	0.57845800	2.90793200
O	-0.08054300	0.54129000	2.54357600
O	-1.43811800	0.23750300	0.78414100
C	-2.68211500	2.08535400	3.06020500

H	-1.82109900	2.57427400	3.53466400
H	-2.84682600	2.54952300	2.07618400
H	-3.57189400	2.26457900	3.68040300
C	-2.16799900	-0.05988000	4.26545100
H	-1.95352700	-1.13385900	4.15950900
H	-1.30597500	0.40815800	4.75350700
H	-3.04541300	0.05149400	4.91788400
C	-3.67260300	-0.05967900	2.25788000
H	-3.50454300	-1.12533700	2.04721000
H	-4.53360500	0.02277700	2.93616500
H	-3.92754600	0.43199700	1.31055300
C	1.67417500	-1.63485800	1.16824400
O	-6.56860900	-0.33565800	-1.31710100
C	-7.58587200	0.59338600	-1.05709100
H	-7.65644600	1.35700100	-1.84798200
H	-8.52160900	0.02855200	-1.02599500
H	-7.43955300	1.09832000	-0.08891700
C	3.68450400	0.44111100	2.54066900
H	4.70423500	0.16338000	2.84572300
H	3.04306300	-0.39900200	2.82893300
C	3.23577900	1.69141600	3.29327400
H	2.19614500	1.94041900	3.03885100
H	3.28707600	1.53265000	4.37732700
H	3.86213400	2.55953000	3.04560700
C	0.60880700	-2.40356800	1.89284200
C	-0.71401800	-2.56692100	1.47755700
C	1.04161300	-3.06302800	3.04824700
C	-1.58436200	-3.36948100	2.20896400
C	0.16304800	-3.83619900	3.79574800
H	2.08279500	-2.95904200	3.34845500
C	-1.15409800	-3.99655000	3.37455200
H	-2.60883200	-3.50091800	1.86159200
H	0.51098300	-4.32476800	4.70431300
H	-1.84258600	-4.61306700	3.95042200

H	-1.06397500	-2.07780100	0.57486400
C	0.61549700	3.17983300	-0.37836500
C	0.87748000	4.34816500	0.59353300
O	0.32516500	2.07139100	0.24368300
O	0.78685500	3.34651200	-1.57700000
C	2.40083800	4.38934900	0.75124700
H	2.77803500	3.43414400	1.14474700
H	2.88396600	4.56952000	-0.21874400
H	2.69802100	5.18862400	1.44657000
C	0.21059500	4.15684300	1.94884700
H	-0.88284700	4.11726100	1.84182800
H	0.52460500	3.22446200	2.43425200
H	0.45519800	5.00022400	2.61207100
C	0.39292100	5.64592000	-0.04397000
H	-0.69988700	5.64290200	-0.16347100
H	0.66171700	6.50334900	0.59064300
H	0.83808600	5.78159400	-1.03543300
H	0.76378800	0.22124000	1.63920400

B′

SCF Energy (a.u.) = -2616.696983

Thermal correction to Gibbs free Energy (a.u.) = 0.767336

Charge = 0, Multiplicity = 1

Co	-0.08039100	-0.42675000	-0.43128800
O	-2.95624600	1.80787200	1.63293500
O	1.29754400	-1.36427600	-1.35819400
O	1.56893300	2.37952200	-2.91345700
N	-1.40066200	-0.96994100	-1.66272700
N	-1.60531400	0.28499800	0.59276900
N	0.26569600	1.13686000	-1.58547100
C	-1.17758100	-1.45749600	-2.86622700

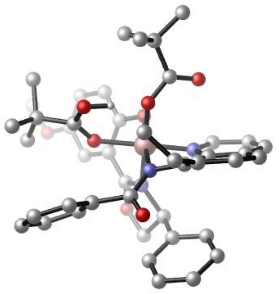

B′

H	-0.14378200	-1.43512200	-3.20597800
C	-2.21600500	-2.01965600	-3.62337500
H	-2.00486600	-2.39949000	-4.61919500
C	-3.46394400	-2.13799700	-3.05645000
H	-4.27761600	-2.62055200	-3.59828800
C	-3.70150200	-1.66301400	-1.75134700
C	-2.62870700	-1.02338400	-1.08799100
C	-2.73217000	-0.46190100	0.21230900
C	-3.87132000	-0.78968200	0.95477800
C	-4.94890700	-1.41696000	0.27425800
H	-5.85404300	-1.60525900	0.85290600
C	-4.91119500	-1.80534500	-1.04070000
H	-5.76825800	-2.27848000	-1.51774200
C	2.46921600	-0.80527700	-1.49348600
C	3.64307800	-1.57200100	-1.35391800
H	3.52989800	-2.62307200	-1.09132400
C	4.90058700	-1.01960100	-1.53768200
H	5.77441400	-1.65532100	-1.40906700
C	5.04893200	0.33412000	-1.87727200
C	3.91462100	1.11355700	-2.03292200
H	4.02702900	2.16178800	-2.30233600
C	2.64328000	0.56458700	-1.83116400
C	1.46203400	1.35192700	-2.05757400
C	0.28414300	3.01741200	-2.94082600
H	0.08256500	3.34643700	-3.96322800
H	0.31942600	3.89223700	-2.27612200
C	-0.66391600	1.94048400	-2.43661200
H	-0.93887200	1.28041500	-3.27798200
C	-1.94516900	2.46224600	-1.84611400
C	-1.99217800	3.56569700	-0.99118100
H	-1.07563400	4.06224000	-0.67460300
C	-3.20643700	4.02530300	-0.49748800
H	-3.22040900	4.86709800	0.19191300
C	-4.39281600	3.39570200	-0.85663600

H	-5.34029700	3.74675600	-0.45242400
C	-4.36181200	2.30985400	-1.72372900
H	-5.28321900	1.80644900	-2.01216300
C	-3.14624600	1.85763000	-2.22234100
H	-3.12570100	1.01634100	-2.91663800
C	1.63446300	-0.20276300	1.94317900
C	2.94761500	0.17854300	2.59028300
O	0.87021000	-0.98313800	2.65527500
O	1.34733200	0.16768700	0.79794300
C	3.76809900	-1.11610200	2.68996300
H	3.26854300	-1.86586900	3.31589500
H	3.94351800	-1.54744400	1.69273700
H	4.74594000	-0.88669900	3.13509900
C	2.68445500	0.73952000	3.98832900
H	2.09827700	1.66707800	3.94086000
H	2.14375900	0.02037700	4.61450600
H	3.64441600	0.96710800	4.47169800
C	3.70332900	1.18468100	1.73270300
H	3.11594100	2.09707400	1.57435800
H	4.63889800	1.45941900	2.23853200
H	3.95395200	0.76762900	0.74895000
C	-1.82786400	1.40523500	1.34627600
O	6.24574000	0.95734500	-2.07150300
C	7.40955200	0.19903300	-1.88434300
H	7.46773400	-0.64539400	-2.58956800
H	8.25164200	0.87190100	-2.06812400
H	7.48212000	-0.19223500	-0.85683700
C	-4.01143000	-0.60793600	2.44018800
H	-4.91202200	-1.15217500	2.76211000
H	-4.17048700	0.45400000	2.67011300
C	-2.81891700	-1.12601400	3.23827500
H	-1.90748300	-0.54686300	3.04455600
H	-3.02311600	-1.07416500	4.31493000
H	-2.59829700	-2.17193500	2.98262000

C	-0.70451700	2.21237400	1.94805300
C	0.33699700	2.81927400	1.24643900
C	-0.86801700	2.53218700	3.30181300
C	1.16921400	3.74591100	1.87043100
C	-0.01376400	3.42389500	3.93720900
H	-1.70281700	2.08905200	3.84261300
C	1.00139000	4.05003600	3.21707800
H	1.95770100	4.23049700	1.29439200
H	-0.15551000	3.64908200	4.99325200
H	1.65666900	4.77148900	3.70303200
H	0.49692100	2.57235000	0.20093900
C	-0.41247300	-3.24349400	0.18902300
C	0.19581400	-4.30086600	1.12496100
O	-0.30517200	-2.00977500	0.64528700
O	-0.91520200	-3.55747400	-0.87275000
C	-0.34644900	-4.17641400	2.54885300
H	-0.05735900	-3.23884800	3.03790300
H	-1.44368600	-4.24605000	2.56146700
H	0.04511300	-4.99832200	3.16568600
C	1.71175500	-4.08954500	1.11110700
H	2.10421000	-4.18954500	0.08874200
H	1.98204000	-3.09241000	1.48159100
H	2.20810900	-4.83872200	1.74513500
C	-0.12824200	-5.68967200	0.58726500
H	0.24394500	-5.81373500	-0.43595400
H	0.33292700	-6.45575000	1.22732600
H	-1.21174200	-5.86260900	0.56514300
H	0.20494000	-1.40856000	2.01667200

C′

SCF Energy (a.u.) = -2269.749937

Thermal correction to Gibbs free Energy (a.u.) = 0.62482

Charge = 0, Multiplicity = 1

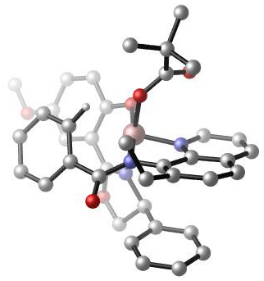

C′

Co	-0.17547100	0.41028500	-0.21082700
O	1.32459400	-1.75220900	2.79581200
O	-1.56487300	1.28019100	-1.15710500
O	-2.62981900	-2.69315200	-1.25901900
N	1.15637600	0.26797900	-1.49426900
N	1.13670000	-0.32601400	0.96920700
N	-0.93846200	-1.32442700	-0.74083900
C	0.99846200	0.56007500	-2.76921900
H	-0.01704600	0.78194800	-3.08911800
C	2.10578300	0.61788900	-3.63147800
H	1.94816900	0.84759400	-4.68135800
C	3.36821800	0.45149600	-3.11219500
H	4.24642800	0.53847500	-3.75212000
C	3.54971700	0.18518800	-1.73830500
C	2.38846800	0.06329000	-0.94457200
C	2.41756200	-0.22820400	0.44765000
C	3.66261900	-0.26242100	1.08134800
C	4.82093100	-0.15356700	0.26865600
H	5.78516000	-0.21256700	0.77456400
C	4.79529400	0.03977100	-1.09053400
H	5.71553000	0.11869100	-1.66739700
C	-2.80291800	0.90754900	-0.97952800
C	-3.82606400	1.88056600	-0.99398300
H	-3.52641400	2.91492500	-1.15068600
C	-5.15214700	1.54706000	-0.79170000
H	-5.89550900	2.34188900	-0.79120800
C	-5.53798300	0.21084200	-0.58612400
C	-4.56856200	-0.77316500	-0.60836300
H	-4.86152100	-1.81126900	-0.46360900
C	-3.21842800	-0.43949400	-0.78965700

C	-2.22576200	-1.46773700	-0.88911700
C	-1.45671700	-3.51851900	-1.23092500
H	-1.52555600	-4.24815300	-2.04136300
H	-1.42170900	-4.03935700	-0.26220500
C	-0.32022500	-2.51939700	-1.37730400
H	-0.19790000	-2.26789700	-2.44670900
C	1.00542800	-3.01120600	-0.87419000
C	1.16522800	-3.52523400	0.41077100
H	0.32485100	-3.53487800	1.10432200
C	2.40620500	-3.97833300	0.84175200
H	2.51825000	-4.32940000	1.86537900
C	3.49822000	-3.94835000	-0.01988700
H	4.47181900	-4.29670400	0.32083100
C	3.34639500	-3.45513500	-1.31221300
H	4.19820900	-3.41496800	-1.98951800
C	2.10666800	-2.98819600	-1.73269600
H	1.99029400	-2.58495600	-2.74073100
C	0.69822600	-0.97586100	2.07672000
O	-6.82056400	-0.19828800	-0.36429900
C	-7.82350200	0.78069000	-0.36168700
H	-7.88782300	1.30568300	-1.32812000
H	-8.76674900	0.25847700	-0.17907200
H	-7.66890000	1.52577300	0.43538600
C	3.83383100	-0.30818700	2.57153700
H	4.90634600	-0.38538900	2.80192300
H	3.33702400	-1.19787000	2.97593300
C	3.25644600	0.93281700	3.25062300
H	2.19635300	1.07118900	2.99390000
H	3.32834500	0.85132000	4.34185100
H	3.78838700	1.84263000	2.93854100
C	-0.75889600	-0.72216300	2.31972800
C	-1.32714800	0.54593700	2.12797300
C	-1.57164300	-1.78905800	2.71056600
C	-2.70492900	0.72601100	2.29706800

C	-2.93838100	-1.60391000	2.86357100
H	-1.10801500	-2.75776700	2.89476000
C	-3.51007400	-0.34619200	2.65116700
H	-3.13747600	1.71405100	2.14736100
H	-3.56989400	-2.44275200	3.15300600
H	-4.58466600	-0.21035400	2.76169400
H	-0.68788800	1.41035700	1.94118600
C	0.94891900	3.03192500	-0.41894900
C	1.68462900	4.11432000	0.39250100
O	0.48755200	2.07819600	0.34081800
O	0.93593100	3.07921300	-1.64031700
C	3.16231800	3.70890200	0.31992600
H	3.31510900	2.70057400	0.73640200
H	3.50988200	3.70166300	-0.72216400
H	3.78379900	4.41376100	0.89213900
C	1.23689500	4.17400000	1.84824400
H	0.16029000	4.38136200	1.92658600
H	1.43560400	3.22996400	2.37086000
H	1.77528700	4.97717500	2.37311900
C	1.48797300	5.47024300	-0.27553100
H	0.43893000	5.79269400	-0.21541200
H	2.10527900	6.23234700	0.22222900
H	1.76422000	5.42420200	-1.33452700

TS2′

SCF Energy (a.u.) = -2269.726318

Thermal correction to Gibbs free Energy (a.u.) = 0.619125

Charge = 0, Multiplicity = 1

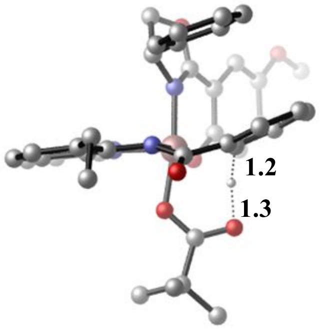

TS2′

Co	-0.05839200	-0.57371100	0.06302400
O	3.03447500	0.85009600	-1.88052200
O	-1.62884400	-1.64210900	0.14885500
O	-2.53457700	1.95067600	2.09117000
N	0.65382900	-1.19784000	1.75235700
N	1.64118400	0.32084700	-0.08441700
N	-0.91593200	0.89308200	0.98066800
C	0.02491600	-1.97738900	2.60649000
H	-1.00874000	-2.21453800	2.35578500
C	0.67018800	-2.48542300	3.74675200
H	0.12375100	-3.12746000	4.43262100
C	1.98723900	-2.15775200	3.96110300
H	2.51995100	-2.53048100	4.83630600
C	2.67403000	-1.32378700	3.05290000
C	1.96093200	-0.85841000	1.92176900
C	2.53817400	0.00000400	0.92668400
C	3.83043600	0.48013000	1.17446800
C	4.53250400	-0.02197600	2.30209200
H	5.54779200	0.34985300	2.45084100
C	4.00951900	-0.89964100	3.21427700
H	4.58735500	-1.24164400	4.07163400
C	-2.84920900	-1.20174300	0.25673700
C	-3.91478600	-2.02354600	-0.17363000
H	-3.65231900	-2.97747900	-0.62700300
C	-5.23625900	-1.64407200	-0.03475100
H	-6.01376600	-2.31703900	-0.39110300
C	-5.57200700	-0.41255900	0.55192400
C	-4.55654200	0.41765300	0.98493400
H	-4.81382800	1.37283700	1.43739000
C	-3.21018400	0.04834200	0.83157000
C	-2.17768700	0.94056100	1.28011300
C	-1.31881700	2.53568000	2.57946200

H	-1.16301300	2.17635900	3.60530400
H	-1.43750000	3.62321600	2.58449600
C	-0.23695500	2.03990500	1.60876300
H	0.64514300	1.67979700	2.15517200
C	0.19316600	3.09693300	0.62200200
C	-0.73986800	3.71191100	-0.21365400
H	-1.78183800	3.38782200	-0.19972500
C	-0.34480500	4.72211700	-1.08227000
H	-1.08056300	5.18688900	-1.73735300
C	0.98668500	5.12701000	-1.12690900
H	1.29588200	5.91355000	-1.81342900
C	1.92168000	4.51811200	-0.29542200
H	2.96757900	4.82059400	-0.32743300
C	1.52186900	3.51345500	0.57947500
H	2.25337600	3.03255000	1.23103200
C	0.67535300	-2.62441300	-1.73463600
C	1.61950000	-3.74803200	-2.14060900
O	-0.16177000	-2.17288700	-2.55179000
O	0.87679200	-2.12441700	-0.58611100
C	1.04022500	-4.54877200	-3.29965000
H	0.10110000	-5.04122200	-3.01366500
H	0.83110900	-3.90558700	-4.16191600
H	1.75399900	-5.32605500	-3.60693100
C	1.91528500	-4.65830900	-0.95254100
H	2.35296900	-4.09526000	-0.11984900
H	1.00121700	-5.14735800	-0.58839200
H	2.62288100	-5.44345000	-1.25436400
C	2.90317900	-3.02947700	-2.58137900
H	3.32676000	-2.43791800	-1.75765700
H	3.65176300	-3.76600600	-2.90626700
H	2.70469300	-2.34677700	-3.41927000
C	1.93154400	0.71355300	-1.36443600
O	-6.84778300	0.03904100	0.72826400
C	-7.89306100	-0.78431900	0.28957700

H	-7.90039400	-1.75395800	0.81304300
H	-8.82232100	-0.25378300	0.51531000
H	-7.84307100	-0.96954600	-0.79552000
C	4.56356200	1.53400700	0.38412800
H	4.97075700	2.26076800	1.10816900
H	3.88440300	2.08043300	-0.27335600
C	5.70533800	0.95565700	-0.44527400
H	5.30112500	0.26686000	-1.19434500
H	6.24885900	1.75125000	-0.97006800
H	6.42593700	0.41211400	0.18179700
C	0.66670500	1.02561400	-2.09603600
C	-0.49512700	0.30147300	-1.76176200
C	0.62791400	2.07037400	-3.00770600
C	-1.71665600	0.70611000	-2.32220000
C	-0.59304900	2.44038200	-3.56596700
H	1.54897300	2.60166500	-3.24420400
C	-1.76554400	1.77170200	-3.21479600
H	-2.62156400	0.13615200	-2.10311200
H	-0.63374800	3.26613000	-4.27600600
H	-2.71508400	2.06833900	-3.65826400
H	-0.43926600	-0.94365400	-1.95004900

D′

SCF Energy (a.u.) = -2269.767695

Thermal correction to Gibbs free Energy (a.u.) = 0.626814

Charge = 0, Multiplicity = 1

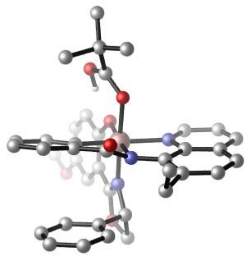

D′

Co	-0.07691400	-0.41870300	0.26702200
O	3.35504700	-0.03946500	-1.50600900
O	-1.77109500	-1.32898300	0.44919500
O	-2.41472000	2.75827800	1.22155500
N	0.47129300	-0.70412400	2.19481700
N	1.70067700	0.27780300	0.10853600
N	-0.85003900	1.27013500	0.67439700
C	-0.24719300	-1.20616700	3.17655500

H	-1.26854600	-1.49164500	2.91931300
C	0.27136700	-1.37397300	4.47310300
H	-0.35292600	-1.79734100	5.25608300
C	1.56741900	-0.98821400	4.71590300
H	2.00507900	-1.09237900	5.70927800
C	2.35442400	-0.43670100	3.68173400
C	1.76132000	-0.31905300	2.39913900
C	2.45838600	0.23470000	1.27125500
C	3.73336500	0.76545300	1.50211600
C	4.30834700	0.61502200	2.78998400
H	5.31078100	1.02266600	2.93253500
C	3.67473100	0.02814700	3.85404400
H	4.15472300	-0.05278200	4.82850200
C	-2.97054400	-0.79388800	0.46618100
C	-4.09590300	-1.61452400	0.26998600
H	-3.92409600	-2.67287700	0.07546500
C	-5.38656800	-1.11689700	0.32859200
H	-6.21893000	-1.79886300	0.16829400
C	-5.61177900	0.24101900	0.59077300
C	-4.52337000	1.07513600	0.77920800
H	-4.70000600	2.13034700	0.97256000
C	-3.21274000	0.58173900	0.70933700
C	-2.11046600	1.50070400	0.86603500
C	-1.17331800	3.43736900	1.46311300
H	-1.04948600	3.53219800	2.54967400
H	-1.23391500	4.43149500	1.01018500
C	-0.10941200	2.53625800	0.81932100
H	0.73728700	2.37887600	1.50042500
C	0.40845800	3.06254300	-0.49511500
C	-0.41756900	3.13695300	-1.61683800
H	-1.44634600	2.77819400	-1.56383100
C	0.07889100	3.62114200	-2.82121500
H	-0.56659300	3.64585400	-3.69775800
C	1.40276800	4.04258800	-2.91360200

H	1.79465900	4.40622800	-3.86206100
C	2.22554900	3.98870200	-1.79317000
H	3.26479300	4.30842300	-1.85653000
C	1.72525600	3.50471700	-0.58909000
H	2.37346600	3.44142100	0.28644100
C	0.35470500	-3.10136400	-0.80007300
C	1.29648100	-4.01981000	-1.54039800
O	-0.92005700	-3.31454800	-0.98303200
O	0.79333800	-2.16421300	-0.12191300
C	0.76545700	-5.45041900	-1.56367600
H	0.67713700	-5.86281300	-0.54918700
H	-0.21834200	-5.50900700	-2.04319200
H	1.46157300	-6.08705500	-2.12614500
C	2.68554800	-3.95935900	-0.91294200
H	3.08282000	-2.93666600	-0.91889600
H	2.67225400	-4.31361300	0.12654100
H	3.36926000	-4.60143300	-1.48437300
C	1.35626500	-3.45152300	-2.96910100
H	1.76993400	-2.43342000	-2.96992000
H	2.00890100	-4.08880800	-3.58139200
H	0.36163400	-3.42036000	-3.43353600
C	2.18485400	0.11713000	-1.17211500
O	-6.84168700	0.82160500	0.67243300
C	-7.96084500	0.00154600	0.47165000
H	-8.01929700	-0.80343400	1.22163900
H	-8.83912800	0.64474600	0.57337400
H	-7.96327100	-0.44966500	-0.53343400
C	4.55103800	1.55466000	0.51186900
H	4.83939700	2.50112600	1.00254700
H	3.95634000	1.81532100	-0.36680900
C	5.80922600	0.82078400	0.06016000
H	5.52616800	-0.09878100	-0.46159000
H	6.39586900	1.44110300	-0.62920100
H	6.45620700	0.56100300	0.90973400

C	1.05925600	0.10795300	-2.14214300
C	-0.21194300	-0.11971900	-1.61036700
C	1.25583100	0.27874600	-3.50914600
C	-1.30602300	-0.17897500	-2.46663400
C	0.15658800	0.23549100	-4.36106900
H	2.26853500	0.44152600	-3.87894800
C	-1.11647900	0.00687000	-3.83883300
H	-2.31193500	-0.36942900	-2.08530800
H	0.28750800	0.37529500	-5.43335500
H	-1.97555600	-0.03463600	-4.50921200
H	-1.41766500	-2.59261100	-0.47677100

E′

SCF Energy (a.u.) = -1922.805581

Thermal correction to Gibbs free Energy (a.u.) = 0.486347

Charge = 0, Multiplicity = 1

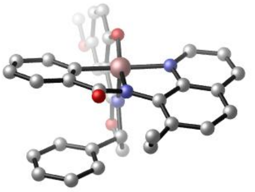

E′

Co	0.05344800	0.41077500	-0.86717200
O	-3.39596200	-1.27972800	-1.68959100
O	1.74168200	0.90157500	-1.50297500
O	2.05160000	-0.10685000	2.56055900
N	-0.51703400	2.34474400	-0.58548400
N	-1.80906500	0.11939600	-0.70830900
N	0.63019000	0.11770100	0.86855300
C	0.24774500	3.41917900	-0.57217300
H	1.29058200	3.25886600	-0.84956400
C	-0.24969800	4.68681000	-0.22692000
H	0.41188800	5.54932600	-0.23524500
C	-1.57464800	4.80002100	0.12407100
H	-1.99648100	5.76417200	0.40957500
C	-2.41063000	3.66389900	0.12910000
C	-1.83591400	2.42812700	-0.25411300
C	-2.58030100	1.20069700	-0.29062000
C	-3.89993500	1.23591100	0.17363900
C	-4.45486600	2.49211700	0.53867300

H	-5.49152300	2.48978500	0.87979500
C	-3.76833500	3.67664500	0.51504800
H	-4.23771800	4.61237500	0.81574000
C	2.88971700	0.71538300	-0.92275500
C	4.08067300	0.91089400	-1.65653900
H	3.98013200	1.18187300	-2.70569700
C	5.32658000	0.76329000	-1.07978600
H	6.20956600	0.92290600	-1.69548100
C	5.45576300	0.41037100	0.27497700
C	4.31337500	0.20892900	1.02285600
H	4.41141500	-0.06535100	2.07066100
C	3.03905500	0.34769500	0.44511900
C	1.87398600	0.12672700	1.25288100
C	0.75324500	-0.12534100	3.17364100
H	0.63421400	0.81095400	3.73327300
H	0.71397100	-0.97389500	3.86322900
C	-0.23595100	-0.24756400	2.00318000
H	-1.03901800	0.49814400	2.08814800
C	-0.85048200	-1.61561400	1.86247500
C	-0.05672800	-2.73611200	1.61176400
H	1.02264800	-2.62630100	1.49638000
C	-0.64024400	-3.98808700	1.46485600
H	-0.01484200	-4.85078700	1.24147100
C	-2.02120200	-4.13354000	1.57659500
H	-2.47816300	-5.11304700	1.44673900
C	-2.81490200	-3.02395000	1.84498100
H	-3.89610600	-3.12625400	1.92718900
C	-2.22738800	-1.77085500	1.99061300
H	-2.84833400	-0.89482700	2.18465900
C	-2.25112600	-1.03069500	-1.33840500
O	6.64604900	0.24842300	0.92111900
C	7.81958300	0.44009100	0.17936700
H	7.89252100	1.46554400	-0.21721800
H	8.65260200	0.26680100	0.86624600

H	7.89622100	-0.26993600	-0.65964200
C	-4.80929400	0.05043000	0.37032000
H	-5.22959000	0.12307200	1.38866600
H	-4.25196300	-0.88667300	0.31327400
C	-5.94805300	0.01020500	-0.64361100
H	-5.53512200	-0.10750300	-1.65060800
H	-6.61499500	-0.83820700	-0.44571300
H	-6.55264700	0.92743800	-0.61400000
C	-1.09937900	-1.93572200	-1.59146300
C	0.17387300	-1.41378200	-1.34673500
C	-1.27204000	-3.23858700	-2.04734100
C	1.29249900	-2.21429200	-1.55361000
C	-0.15250600	-4.04084200	-2.23969500
H	-2.28305100	-3.59976300	-2.23523100
C	1.12191600	-3.52783700	-1.99461300
H	2.29916300	-1.83146800	-1.37603600
H	-0.26751300	-5.06771500	-2.58426700
H	1.99713000	-4.15831000	-2.15359500

F′

SCF Energy (a.u.) = -2537.469154

Thermal correction to Gibbs free Energy (a.u.) = 0.674359

Charge = 0, Multiplicity = 1

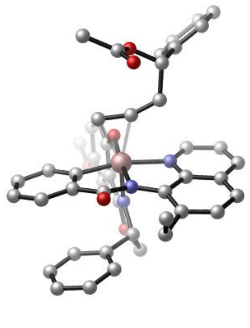

F′

Co	-0.22249700	0.06347500	0.21582200
O	-2.51005400	-2.97644700	1.30613300
O	1.02980900	1.49329200	0.47685100
O	-2.10833800	3.03160700	-1.87397600
N	0.54539200	-0.48232500	-1.55022700
N	-1.30094500	-1.50258600	-0.04587300
N	-1.41957100	1.20247100	-0.80060100
C	1.49183300	0.14250000	-2.22159100
H	1.90860400	1.02824100	-1.74029700
C	1.96241800	-0.33393300	-3.45677000
H	2.75600900	0.19833600	-3.97475100

C	1.40329600	-1.47817600	-3.97287400
H	1.73946100	-1.88332300	-4.92767500
C	0.37236000	-2.14904100	-3.27897000
C	-0.03992800	-1.61387100	-2.03148000
C	-1.08222100	-2.20074800	-1.22525300
C	-1.78188500	-3.27840400	-1.79542900
C	-1.32211500	-3.80791500	-3.02921000
H	-1.86734100	-4.66921300	-3.41942400
C	-0.28244600	-3.29903400	-3.76128400
H	0.01700900	-3.73859700	-4.71148900
C	0.87198900	2.76276200	0.24505900
C	1.84790700	3.66843500	0.71947200
H	2.67557000	3.25200900	1.29242300
C	1.77231400	5.02608400	0.47473300
H	2.55376200	5.67394500	0.86714800
C	0.70499700	5.55981300	-0.26551500
C	-0.26699100	4.70378100	-0.74524400
H	-1.09124700	5.11737700	-1.32130400
C	-0.20416400	3.32252900	-0.49529800
C	-1.22999300	2.46658900	-1.02843700
C	-2.86736600	1.96656000	-2.45930000
H	-2.48461600	1.79977300	-3.47505000
H	-3.91579600	2.27574700	-2.50616500
C	-2.62487800	0.76839100	-1.53105500
H	-2.38673700	-0.13035700	-2.11527300
C	-3.80322100	0.47158100	-0.63937800
C	-4.22114600	1.38074800	0.33320200
H	-3.65465500	2.29738800	0.50311200
C	-5.33420800	1.10279100	1.11656600
H	-5.63818800	1.80845400	1.88826400
C	-6.04129500	-0.08388900	0.93517200
H	-6.90440100	-0.30560900	1.56074600
C	-5.63531200	-0.98812200	-0.04002300
H	-6.17458200	-1.92324800	-0.18423700

C	-4.52556600	-0.70315500	-0.82911300
H	-4.20111900	-1.41221300	-1.59252700
C	-2.03763200	-1.87299100	1.06271600
O	0.55001700	6.88430300	-0.55643500
C	1.52493800	7.77039600	-0.08060300
H	2.52312900	7.54089100	-0.48757900
H	1.22794900	8.76840400	-0.41478600
H	1.58307000	7.76593900	1.01984200
C	-3.01721600	-3.95847100	-1.25729800
H	-3.70080000	-4.11001800	-2.10992500
H	-3.53888400	-3.33316300	-0.53155900
C	-2.70297600	-5.30647800	-0.61711900
H	-2.06178500	-5.15584800	0.25766200
H	-3.62221800	-5.80497700	-0.28472000
H	-2.18846300	-5.97679100	-1.32026800
C	-2.17616600	-0.71626200	1.98393400
C	-1.34559100	0.38068900	1.74819300
C	-3.08194800	-0.71792400	3.04042500
C	-1.42897800	1.49847900	2.56820300
C	-3.17725800	0.40625900	3.85420200
H	-3.70322400	-1.59993400	3.19586700
C	-2.35716100	1.50895000	3.61297400
H	-0.77783300	2.36013000	2.40732100
H	-3.88861800	0.42745600	4.67872900
H	-2.43264100	2.38850900	4.25305000
C	5.58973100	0.91094700	-0.31241100
C	6.73499000	0.36466600	-0.88941900
C	6.90354400	-1.01517400	-0.92333800
C	5.92835800	-1.84790500	-0.38058200
C	4.78206700	-1.30752300	0.19526600
C	4.61516700	0.08007500	0.22455100
H	5.45158600	1.99121500	-0.28749300
H	7.49549200	1.01726300	-1.31570700
H	7.79593500	-1.44675700	-1.37392300

H	6.05688200	-2.93070800	-0.40409400
H	3.70604100	0.50402900	0.65592400
C	3.71502300	-2.20577900	0.77256800
H	3.92613800	-3.25910100	0.53653500
O	3.73929700	-2.04257900	2.19338500
C	3.19976200	-3.05036800	2.91786300
O	2.76116200	-4.06312000	2.43487300
C	3.24086800	-2.71330600	4.37779200
H	2.66019000	-1.80241100	4.56737100
H	2.83005500	-3.54190500	4.95855800
H	4.27104400	-2.50740000	4.68988300
C	2.38057600	-1.80987800	0.19311300
H	2.29689600	-1.95414200	-0.88186500
C	1.48091300	-1.18540700	0.90970800
C	0.87888700	-0.70186200	2.00102600
H	0.26384100	-1.35635800	2.61947600
H	1.14693600	0.26933600	2.41280300

F′′

SCF Energy (a.u.) = -2537.481992

Thermal correction to Gibbs free Energy (a.u.) = 0.682206

Charge = 0, Multiplicity = 1

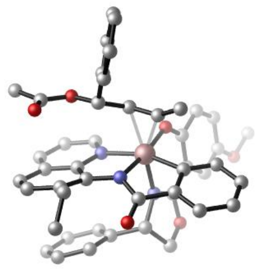

F′′

Co	-0.43864300	-0.52995900	0.30087200
O	1.34634100	1.53839200	3.17886200
O	-1.65163100	-1.64798800	-0.67217100
O	-3.93002300	1.60484500	0.78708800
N	0.10204800	0.53683400	-1.31840800
N	0.93996500	0.50111500	1.13686700
N	-1.84499900	0.83371800	0.58449200
C	-0.44750600	0.51587400	-2.51514500
H	-1.25305100	-0.20558700	-2.65725900
C	-0.01300300	1.36463000	-3.54803100
H	-0.50490100	1.32886500	-4.51684700
C	1.04386100	2.21114600	-3.30773600

H	1.42702800	2.86584200	-4.09165900
C	1.67279900	2.22112400	-2.04536800
C	1.13404500	1.37809600	-1.04371300
C	1.68181600	1.30928800	0.27643000
C	2.91297000	1.92680600	0.49707900
C	3.43464500	2.76925900	-0.51838700
H	4.38595700	3.26189100	-0.31267400
C	2.83437100	2.96215200	-1.73513500
H	3.26831900	3.62267700	-2.48549100
C	-2.93414100	-1.52816100	-0.84557400
C	-3.62159800	-2.55532900	-1.53232500
H	-3.03086000	-3.40270100	-1.87583300
C	-4.98307200	-2.50645800	-1.75777200
H	-5.45591100	-3.32895500	-2.29108500
C	-5.74446200	-1.41919500	-1.29836700
C	-5.10701000	-0.40068300	-0.61908600
H	-5.69765500	0.43685100	-0.25638100
C	-3.71917300	-0.43327700	-0.39493600
C	-3.10470200	0.65083400	0.32150200
C	-3.15932600	2.39529200	1.70112700
H	-3.37839200	2.04134300	2.71872300
H	-3.46016300	3.44142300	1.59308200
C	-1.70941500	2.11997300	1.30257600
H	-1.10256300	1.97773100	2.20300500
C	-1.08654800	3.20707500	0.46332300
C	-1.63037400	3.56795600	-0.77104900
H	-2.50456000	3.03990300	-1.15637000
C	-1.05428300	4.58860500	-1.51902900
H	-1.48057100	4.85772700	-2.48465300
C	0.06696100	5.26088700	-1.03717200
H	0.52194900	6.05533000	-1.62728100
C	0.61026800	4.90713600	0.19346600
H	1.49988700	5.41182300	0.56882500
C	0.03492800	3.88427500	0.94116100

H	0.47903300	3.57844500	1.89051700
C	0.79918700	0.67848700	2.48858200
O	-7.09241500	-1.28918200	-1.47252600
C	-7.76341600	-2.32823800	-2.12943300
H	-7.66335700	-3.28610100	-1.59367500
H	-8.82005700	-2.04757200	-2.15751800
H	-7.40383300	-2.46302900	-3.16253100
C	3.79457500	1.69022500	1.69162000
H	4.76531100	1.34130800	1.30019600
H	3.39205700	0.88529000	2.31448200
C	3.99689500	2.93536900	2.54790900
H	3.03918900	3.24616300	2.97922100
H	4.69415100	2.73676800	3.37128100
H	4.40602100	3.76937400	1.96008200
C	-0.22932700	-0.25127700	3.02334500
C	-0.94985000	-1.00330400	2.09372300
C	-0.54965500	-0.29708400	4.38014200
C	-2.00785400	-1.80114900	2.51445300
C	-1.59281400	-1.10960900	4.80644100
H	0.02523900	0.31754100	5.07263100
C	-2.32182300	-1.85084600	3.87261600
H	-2.57351800	-2.39493300	1.79547500
H	-1.85041600	-1.16268300	5.86312200
H	-3.14750500	-2.47852900	4.20817500
C	3.87134500	-5.09275600	-1.09498300
C	4.72640300	-5.17923200	0.00133300
C	4.86280200	-4.09502800	0.86290500
C	4.14262200	-2.92773800	0.63139900
C	3.27899800	-2.84058900	-0.45764100
C	3.14839800	-3.92792000	-1.32292500
H	3.76544200	-5.93850200	-1.77268500
H	5.29049200	-6.09304900	0.18134500
H	5.53683500	-4.15533800	1.71574700
H	4.25034000	-2.06703600	1.29277200

H	2.47590200	-3.85550200	-2.17849100
C	2.44478800	-1.59946500	-0.65594600
H	2.76721100	-0.83810800	0.06357000
O	2.60946900	-1.08988200	-1.98840000
C	3.76332200	-0.41853600	-2.20407000
O	4.57742600	-0.19376100	-1.34301600
C	3.89346100	-0.02316100	-3.64388700
H	4.38881300	0.95079900	-3.70360700
H	4.52823900	-0.75699600	-4.15625700
H	2.92117600	0.00588300	-4.14422100
C	0.98526300	-1.93858200	-0.47255700
H	0.42596200	-2.21217000	-1.36995300
C	0.57264200	-2.37530900	0.74200200
C	0.70575600	-3.21599200	1.74316000
H	1.30085400	-4.11373600	1.55722000
H	0.26019900	-3.09804800	2.72596200

TS3′

SCF Energy (a.u.) = -2537.464759

Thermal correction to Gibbs free Energy (a.u.) = 0.679481

Charge = 0, Multiplicity = 1

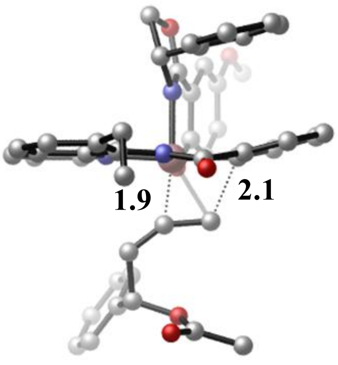

TS3′

Co	-0.09521200	0.01347600	0.02180500
O	-2.47661800	-2.80121500	1.52398000
O	1.21496400	1.42519300	0.01357000
O	-2.16470600	3.04951900	-1.89584100
N	0.38858800	-0.61613400	-1.75928800
N	-1.27893700	-1.50190400	-0.00824500
N	-1.42636100	1.21416600	-0.85744300
C	1.28130900	-0.05841200	-2.55338900
H	1.75825700	0.84173100	-2.16482600
C	1.61530800	-0.62113300	-3.79597500
H	2.36275700	-0.14032700	-4.42154700
C	0.98556800	-1.77898400	-4.18442600
H	1.21633900	-2.24717700	-5.14159500

C	0.01655800	-2.38074200	-3.35245300
C	-0.25875700	-1.76324300	-2.10630900
C	-1.22295100	-2.27080300	-1.16509300
C	-2.00839100	-3.35252900	-1.59685300
C	-1.68763900	-3.96677500	-2.83610000
H	-2.29161500	-4.83108800	-3.11782500
C	-0.71222400	-3.53649100	-3.69521200
H	-0.52071000	-4.04072500	-4.64112400
C	1.00678400	2.70871300	-0.08744500
C	2.01279500	3.59543100	0.35425500
H	2.90254000	3.15423100	0.80329600
C	1.89528000	4.96782900	0.22631300
H	2.70449300	5.59953700	0.58743700
C	0.75159900	5.53250300	-0.35708100
C	-0.25540800	4.69354700	-0.79823100
H	-1.14311400	5.13193900	-1.24840300
C	-0.15062400	3.30147200	-0.66025800
C	-1.23269600	2.46669400	-1.11893000
C	-2.99956600	1.99535300	-2.39312200
H	-2.71184400	1.80123900	-3.43509000
H	-4.04012300	2.33164500	-2.35462200
C	-2.70309100	0.80740300	-1.46546400
H	-2.55354700	-0.11430000	-2.04462400
C	-3.79255000	0.57716800	-0.44794400
C	-4.08813000	1.54630400	0.51180000
H	-3.48220300	2.45119900	0.57850200
C	-5.13031000	1.34819700	1.40829300
H	-5.34222100	2.10475600	2.16252900
C	-5.88461700	0.17813500	1.35974200
H	-6.69278800	0.01929200	2.07192800
C	-5.59413800	-0.79153200	0.40591100
H	-6.16778700	-1.71646300	0.36748200
C	-4.55835900	-0.58481000	-0.49975600
H	-4.32692600	-1.34430000	-1.24865800

C	-1.92510500	-1.76299700	1.17891300
O	0.54679100	6.87124000	-0.52587900
C	1.55031500	7.74059900	-0.07959800
H	2.50520000	7.56950300	-0.60252400
H	1.20394000	8.75445300	-0.29877600
H	1.72101500	7.64906200	1.00527900
C	-3.20305000	-3.96080300	-0.90381000
H	-3.96120600	-4.15715600	-1.68050100
H	-3.64651900	-3.27111500	-0.18453300
C	-2.85640100	-5.26592800	-0.19540700
H	-2.13894200	-5.06815400	0.60794500
H	-3.75122500	-5.71868600	0.24988200
H	-2.41533500	-5.99585500	-0.88907800
C	-1.88203300	-0.56121800	2.05273600
C	-0.90477800	0.40261100	1.78001400
C	-2.78769600	-0.38322600	3.09210100
C	-0.86049700	1.57283700	2.53395500
C	-2.74412700	0.78697500	3.84326600
H	-3.52461000	-1.16347300	3.28018100
C	-1.79407700	1.76633200	3.55119500
H	-0.09279700	2.32539700	2.34430000
H	-3.45453300	0.94286300	4.65401800
H	-1.76640300	2.68617700	4.13500100
C	5.64811200	0.76359100	-0.38044800
C	6.86595600	0.16536900	-0.69719200
C	7.01914800	-1.20995000	-0.55655400
C	5.95661400	-1.98323500	-0.09785000
C	4.73662900	-1.39080700	0.21941300
C	4.58582300	-0.00910800	0.07225500
H	5.52103800	1.83974500	-0.49408500
H	7.69522900	0.77227000	-1.05799200
H	7.96761400	-1.68344000	-0.80571900
H	6.07291900	-3.06182400	0.01630200
H	3.62322100	0.45624200	0.29564000

C	3.59345400	-2.23113200	0.73643400
H	3.79583400	-3.29845900	0.56447200
O	3.52543300	-2.01354100	2.15521000
C	2.95872200	-3.00060500	2.88379200
O	2.54360000	-4.03096800	2.41664400
C	2.94546500	-2.62615400	4.33658500
H	2.47455900	-1.64596100	4.47615000
H	2.40805700	-3.38645500	4.90754500
H	3.97380900	-2.54221800	4.70767500
C	2.30755500	-1.83390300	0.06348200
H	2.25976000	-2.07669800	-0.99944000
C	1.39035700	-1.10349800	0.65988500
C	0.96702000	-0.52057500	1.83731800
H	0.54223000	-1.15834600	2.61343400
H	1.46868700	0.38150900	2.19004800

TS3″

SCF Energy (a.u.) = -2537.479562

Thermal correction to Gibbs free Energy (a.u.) = 0.683725

Charge = 0, Multiplicity = 1

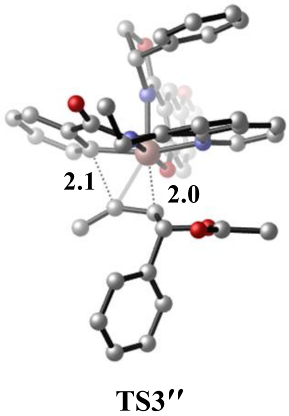

TS3″

Co	-0.39092600	-0.51216900	0.21807200
O	1.37322000	1.45060900	3.17562200
O	-1.63251600	-1.58060400	-0.78299700
O	-3.91967200	1.58248700	0.89122600
N	0.11250700	0.59792200	-1.33271000
N	0.97981600	0.48822200	1.09548300
N	-1.83049000	0.84296500	0.60684100
C	-0.44915300	0.60305800	-2.52439000
H	-1.25993700	-0.11044500	-2.67092800
C	-0.01632200	1.46921100	-3.54336600
H	-0.51776000	1.45549000	-4.50775100
C	1.04866900	2.30393000	-3.29776500
H	1.42723000	2.97186800	-4.07249000
C	1.69000200	2.28460900	-2.04181400

C	1.15643100	1.42480000	-1.05309700
C	1.71648600	1.32338400	0.25737900
C	2.94740500	1.93524000	0.48658400
C	3.46332000	2.79935400	-0.51399000
H	4.41519700	3.28882400	-0.30335200
C	2.85586200	3.01613800	-1.72332700
H	3.28646200	3.69074700	-2.46285600
C	-2.92343600	-1.46677900	-0.90355300
C	-3.61869100	-2.46850900	-1.61909500
H	-3.02611100	-3.28563500	-2.02692900
C	-4.98829800	-2.43486700	-1.79523700
H	-5.46501200	-3.23798000	-2.35404600
C	-5.75039700	-1.38721500	-1.25442700
C	-5.10503500	-0.39218800	-0.54773600
H	-5.69614900	0.41592500	-0.12416900
C	-3.71041600	-0.40974200	-0.37180900
C	-3.09241900	0.65622500	0.37214400
C	-3.13166800	2.36880100	1.79307000
H	-3.32360900	2.00544900	2.81270300
H	-3.44111200	3.41435600	1.70250200
C	-1.68798700	2.10610700	1.35897200
H	-1.06429600	1.94248800	2.24480600
C	-1.07489200	3.21209500	0.53712000
C	-1.63272500	3.59841100	-0.68322500
H	-2.51444600	3.08138600	-1.06644600
C	-1.06058900	4.62900700	-1.42045900
H	-1.49776200	4.91784100	-2.37546800
C	0.07113300	5.28592000	-0.94169200
H	0.52311400	6.08816300	-1.52352200
C	0.62851600	4.90708600	0.27507300
H	1.52568100	5.40054600	0.64740800
C	0.05720900	3.87374100	1.01154500
H	0.51254100	3.54681200	1.94856300
C	0.83743000	0.61286900	2.44954800

O	-7.10603400	-1.27289000	-1.37327900
C	-7.78249700	-2.28799600	-2.06093600
H	-7.64779900	-3.27075700	-1.58064600
H	-8.84403500	-2.02580000	-2.03795800
H	-7.45719500	-2.36191000	-3.11144100
C	3.82838500	1.67563700	1.67623000
H	4.80304200	1.34494000	1.27961100
H	3.43082000	0.85094800	2.27640100
C	4.01580300	2.89998300	2.56533700
H	3.05438000	3.18875200	3.00383900
H	4.71418900	2.68728000	3.38416900
H	4.41613500	3.75376100	2.00031700
C	-0.19050800	-0.34154800	2.94605000
C	-0.84674100	-1.13736100	2.00185300
C	-0.60657100	-0.33707700	4.27656500
C	-1.93051200	-1.92418400	2.38102100
C	-1.67667700	-1.13527400	4.66217000
H	-0.08553000	0.31202600	4.97989800
C	-2.34135300	-1.91572100	3.71174700
H	-2.43875100	-2.54801600	1.64578500
H	-2.00645500	-1.14536200	5.69982300
H	-3.19013000	-2.52871600	4.01388100
C	3.73433600	-5.15497100	-1.07159900
C	4.62659200	-5.24966400	-0.00579000
C	4.82402500	-4.15736100	0.83341200
C	4.12589200	-2.97499900	0.61119200
C	3.22218000	-2.87974800	-0.44463900
C	3.03372600	-3.97459300	-1.28975600
H	3.58174300	-6.00670100	-1.73278300
H	5.17221300	-6.17601400	0.16708900
H	5.52776900	-4.22376900	1.66149700
H	4.28050900	-2.10819200	1.25521800
H	2.33162600	-3.89642300	-2.12062100
C	2.41239900	-1.62059700	-0.62500200

H	2.76892400	-0.86987000	0.08888400
O	2.57396500	-1.10994300	-1.96059400
C	3.72772000	-0.44564300	-2.18661700
O	4.55579800	-0.22854200	-1.33615500
C	3.84054900	-0.04030000	-3.62571300
H	4.34974400	0.92648000	-3.68593900
H	4.45454500	-0.77959100	-4.15529400
H	2.86056100	0.00784500	-4.10965200
C	0.94495900	-1.90928000	-0.42687900
H	0.41834800	-2.28666000	-1.30781400
C	0.51859500	-2.31509000	0.82470100
C	0.75886600	-3.22046600	1.75530700
H	1.42172700	-4.04541800	1.48174500
H	0.34604800	-3.21537800	2.75948800

G′

SCF Energy (a.u.) = -2537.507082

Thermal correction to Gibbs free Energy (a.u.) = 0.683033

Charge = 0, Multiplicity = 1

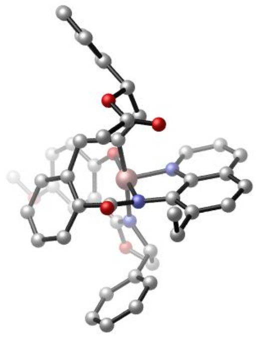

G′

Co	-0.21428400	-0.00622500	-0.48006000
O	1.99986100	-1.54720600	2.29637400
O	-1.25095100	1.46984800	-1.11142500
O	-3.91841500	-1.70944300	-1.42393900
N	0.59882900	-0.38083600	-2.11014700
N	0.95334100	-1.35641400	0.21164700
N	-1.81381800	-1.25550400	-0.80311100
C	0.31544100	0.22055900	-3.25202100
H	-0.47110000	0.97139600	-3.20123000
C	1.01935900	-0.08651400	-4.42821400
H	0.76428200	0.42889500	-5.35037600
C	2.02140400	-1.02700200	-4.38423200
H	2.58613600	-1.28291800	-5.28076700
C	2.33766200	-1.67503900	-3.17118700
C	1.59377400	-1.31299100	-2.02354700

C	1.82965900	-1.87379600	-0.73167200
C	2.78430300	-2.88613900	-0.62862700
C	3.52219700	-3.23311600	-1.78948100
H	4.27412700	-4.01594700	-1.67755200
C	3.33332300	-2.66353600	-3.02204500
H	3.92262200	-2.97005700	-3.88484900
C	-2.52266400	1.61250300	-0.83846100
C	-3.03511600	2.90961000	-0.63234600
H	-2.32353500	3.73440800	-0.67770500
C	-4.37267800	3.14185800	-0.36028500
H	-4.70518900	4.16441200	-0.19246100
C	-5.28098200	2.07559900	-0.30286300
C	-4.81861100	0.78992700	-0.52840900
H	-5.52678800	-0.03568600	-0.49512000
C	-3.46247300	0.54617400	-0.77622000
C	-3.01218400	-0.80964600	-0.98949700
C	-3.17235900	-2.87891100	-1.79235500
H	-3.08436100	-2.88834200	-2.88684300
H	-3.72740600	-3.76310600	-1.46444200
C	-1.81600800	-2.70003700	-1.09493200
H	-0.98447300	-2.92406100	-1.77896400
C	-1.66491300	-3.54246200	0.14964500
C	-2.70536100	-3.62551300	1.07742700
H	-3.62206000	-3.05506000	0.91517300
C	-2.57890000	-4.41787800	2.21235900
H	-3.40010100	-4.47669500	2.92569000
C	-1.40119700	-5.12568600	2.44167300
H	-1.29830000	-5.74211000	3.33313400
C	-0.35694100	-5.04244700	1.52543900
H	0.56924300	-5.58901500	1.69731100
C	-0.49401000	-4.26050000	0.38208900
H	0.32520500	-4.19670000	-0.33679500
C	1.09024300	-1.19868000	1.55188200
O	-6.61443300	2.20433500	-0.04000500

C	-7.10727500	3.49670500	0.18038200
H	-6.95749000	4.14852600	-0.69542100
H	-8.18015400	3.39372500	0.36583300
H	-6.63800700	3.97179800	1.05720100
C	3.10572800	-3.65102100	0.62489500
H	3.15440900	-4.72312300	0.36814400
H	2.31387900	-3.52754200	1.36791900
C	4.43594600	-3.20573700	1.22570100
H	4.41478300	-2.12635100	1.41421700
H	4.63359500	-3.72560800	2.17126100
H	5.27215800	-3.40669500	0.54180700
C	-0.15638400	-0.52105200	2.07091800
C	-0.43979500	0.83888700	1.82949300
C	-1.07976500	-1.31790000	2.75536500
C	-1.68458900	1.34218000	2.24440400
C	-2.29901600	-0.79669800	3.15520600
H	-0.83014700	-2.36156300	2.94838000
C	-2.61108800	0.53725300	2.88351600
H	-1.91251300	2.39105500	2.05290400
H	-3.01673400	-1.43388500	3.67094000
H	-3.57672600	0.94788700	3.17417100
C	1.33752800	5.77560200	-0.42628900
C	2.06812900	6.63075800	0.39100700
C	3.18253300	6.14468100	1.06984300
C	3.56085700	4.81521000	0.92975400
C	2.82935300	3.94759400	0.11476700
C	1.71450700	4.44257900	-0.56358500
H	0.46118100	6.14321100	-0.95905100
H	1.77029300	7.67246300	0.50172000
H	3.76072700	6.80571000	1.71416700
H	4.42752400	4.43495100	1.46819600
H	1.12748100	3.76865800	-1.18844400
C	3.28336300	2.51406200	-0.05380600
H	4.18592100	2.48734700	-0.68385100

O	3.69019500	2.05824700	1.26221900
C	4.35329600	0.88449600	1.30667700
O	4.71161700	0.27650300	0.32490800
C	4.54444700	0.43678700	2.72256500
H	3.69421000	-0.21191300	2.98258200
H	5.46061100	-0.15608700	2.80030800
H	4.56822700	1.28175200	3.41701900
C	2.26376700	1.57786200	-0.64773000
H	2.55104200	1.11706900	-1.59382800
C	1.12888900	1.25034400	-0.03697600
C	0.60730600	1.77922600	1.26097700
H	1.41359100	1.94107000	1.99536600
H	0.13380300	2.75829800	1.08489900

G″

SCF Energy (a.u.) = -2537.516503

Thermal correction to Gibbs free Energy (a.u.) = 0.685516

Charge = 0, Multiplicity = 1

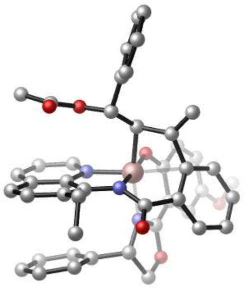

G″

Co	-0.35723500	-0.32612500	0.02912800
O	1.06024900	1.19791000	3.33513200
O	-1.59170000	-1.25737900	-1.09842700
O	-3.90174400	1.83499000	0.72164600
N	0.40588700	0.69388800	-1.34189200
N	0.97446400	0.46719200	1.12614200
N	-1.80955000	1.08951600	0.43274100
C	-0.01933100	0.74761100	-2.59022500
H	-0.85047200	0.08784600	-2.83210600
C	0.58359300	1.60052700	-3.53035400
H	0.19523000	1.63248300	-4.54505300
C	1.66130200	2.36549100	-3.14896600
H	2.16229600	3.02144900	-3.86168800
C	2.15319100	2.28880600	-1.82916600
C	1.46184100	1.44997900	-0.92644400
C	1.84935600	1.29972400	0.43753900

C	3.05235400	1.86991000	0.84300100
C	3.73286600	2.71321300	-0.07356300
H	4.66371900	3.16736400	0.26947100
C	3.30295500	2.95760200	-1.35239900
H	3.85688900	3.61613000	-2.02045500
C	-2.89416700	-1.22656300	-1.06694800
C	-3.59929600	-2.27134500	-1.70749600
H	-3.00141800	-3.04334800	-2.18937000
C	-4.97930600	-2.33776800	-1.72393600
H	-5.45896800	-3.17420700	-2.22886900
C	-5.74449400	-1.34555200	-1.09273600
C	-5.09209400	-0.30333200	-0.46348100
H	-5.68665200	0.46574200	0.02355000
C	-3.68944300	-0.22415300	-0.44214300
C	-3.07276600	0.89086100	0.23385900
C	-3.10115300	2.75784400	1.46616900
H	-3.34180800	2.63618300	2.53056700
H	-3.36110400	3.77395800	1.14970800
C	-1.64826900	2.36855400	1.13850900
H	-1.08396600	2.20080600	2.06664300
C	-0.90662600	3.39942900	0.32674900
C	-1.33601400	3.74257100	-0.95613100
H	-2.20022600	3.23509600	-1.39001300
C	-0.65758200	4.71142500	-1.68647900
H	-0.99424800	4.96745400	-2.69032600
C	0.45382300	5.34871600	-1.13802400
H	0.98788400	6.10420200	-1.71290100
C	0.88658200	5.00997800	0.14006900
H	1.76733800	5.48698100	0.56843500
C	0.20847200	4.03701400	0.86828200
H	0.55881400	3.74385000	1.86002400
C	0.64818900	0.46415100	2.43866600
O	-7.11021700	-1.32809600	-1.04930200
C	-7.78896100	-2.37383900	-1.68630100

H	-7.54016200	-3.35414100	-1.24801300
H	-8.85689200	-2.18452500	-1.54489500
H	-7.57342800	-2.40723500	-2.76669500
C	3.73319500	1.59660000	2.15345000
H	4.77270200	1.31229900	1.92188300
H	3.27393600	0.73878600	2.65458000
C	3.71744700	2.79946500	3.09090400
H	2.68792700	3.01187100	3.39934400
H	4.30884000	2.60344700	3.99371600
H	4.13274200	3.69427600	2.60503900
C	-0.51393300	-0.47226600	2.64826800
C	-0.72434100	-1.65285900	1.88805400
C	-1.51210200	-0.02359700	3.51681400
C	-1.98975700	-2.26934800	1.96451100
C	-2.74224400	-0.65942500	3.58079700
H	-1.29518200	0.85691900	4.12076900
C	-2.99574900	-1.77021300	2.77374900
H	-2.15705700	-3.16457200	1.36556300
H	-3.51662900	-0.28063300	4.24654000
H	-3.97158300	-2.25210500	2.78880500
C	3.35206600	-5.38287200	-0.99724300
C	4.30966600	-5.58799100	-0.00619400
C	4.64369300	-4.55059300	0.85950800
C	4.01335600	-3.31629300	0.74172500
C	3.03603600	-3.11317700	-0.22980100
C	2.72007100	-4.15021800	-1.10937500
H	3.09415700	-6.18964800	-1.68198400
H	4.80049900	-6.55576100	0.08610200
H	5.40118800	-4.70173300	1.62705400
H	4.27386600	-2.49355900	1.40851700
H	1.96480800	-3.98772400	-1.87901700
C	2.31451800	-1.79038000	-0.31749700
H	2.75836800	-1.10449700	0.41582500
O	2.54318500	-1.25094900	-1.64199300

C	3.76001400	-0.71277300	-1.84924800
O	4.59687600	-0.57089000	-0.98978800
C	3.94283300	-0.34299300	-3.29215700
H	4.63305700	0.50291300	-3.36407700
H	4.38799800	-1.19581700	-3.82033100
H	2.98775400	-0.10532900	-3.77132900
C	0.81479100	-1.89310100	-0.12297700
H	0.35484700	-2.43975100	-0.95933400
C	0.37593800	-2.43630000	1.20315900
C	0.76366600	-3.56259400	1.81155600
H	1.50362300	-4.23893600	1.39019900
H	0.33275400	-3.83959100	2.77426700

TS4′

SCF Energy (a.u.) = -2537.473529

Thermal correction to Gibbs free Energy (a.u.) = 0.686714

Charge = 0, Multiplicity = 1

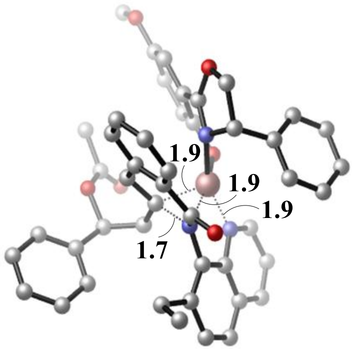

TS4′

Co	-0.50092800	0.29662600	-1.20618600
O	-3.58792400	0.28932600	1.17405000
O	1.01220400	0.56210900	-2.32796400
O	0.78729200	4.03371900	-0.20968900
N	-1.09355800	-1.17485000	-2.27829600
N	-1.69541800	-0.52160800	0.17253500
N	-0.32484200	2.15432400	-0.68609000
C	-0.70065200	-1.41269100	-3.51730400
H	0.11742300	-0.78567700	-3.87025900
C	-1.28037700	-2.42596300	-4.30160400
H	-0.93550600	-2.57636900	-5.32141100
C	-2.25368400	-3.22126700	-3.74818900
H	-2.70868900	-4.03182700	-4.31766000
C	-2.66310400	-3.00957000	-2.41361600
C	-2.06996800	-1.93710400	-1.70634100
C	-2.39432100	-1.63676200	-0.35752000
C	-3.23625600	-2.51380900	0.33111700

C	-3.84782800	-3.56117200	-0.40593000
H	-4.53782600	-4.21031500	0.13281100
C	-3.60303300	-3.80679500	-1.73176700
H	-4.09393200	-4.62783300	-2.25282500
C	2.09001500	1.06953700	-1.79397800
C	3.36195500	0.62918000	-2.21799500
H	3.39447000	-0.15206200	-2.97545400
C	4.53088200	1.13334500	-1.68100700
H	5.47912700	0.72966500	-2.02975700
C	4.50376700	2.12115700	-0.68283400
C	3.28264900	2.61849200	-0.27393600
H	3.24982100	3.41429400	0.46879300
C	2.09167100	2.10852200	-0.81152500
C	0.82960700	2.72538600	-0.53081200
C	-0.60940500	4.33511300	-0.01364100
H	-0.78054100	5.36596600	-0.33455100
H	-0.84321900	4.22178400	1.05730000
C	-1.27880100	3.28057700	-0.87103100
H	-1.15162900	3.58705300	-1.92826400
C	-2.73681500	2.98506200	-0.69159800
C	-3.54879100	3.70484900	0.18148000
H	-3.11783500	4.47553300	0.82156100
C	-4.91484200	3.44225400	0.25155800
H	-5.53840000	4.01118100	0.93969300
C	-5.47827600	2.45348700	-0.54549000
H	-6.54313400	2.23809900	-0.47744100
C	-4.67612700	1.73853900	-1.43144900
H	-5.10761400	0.95947700	-2.05780100
C	-3.32033300	2.01741900	-1.51502300
H	-2.69115600	1.46886900	-2.22118400
C	-2.37004900	0.29559400	1.08995600
O	5.61370600	2.63601700	-0.07372300
C	6.86345900	2.21955900	-0.54797200
H	6.99646300	2.45325000	-1.61679300

H	7.61442800	2.76523100	0.03072900
H	7.01947300	1.13770500	-0.40334700
C	-3.51834000	-2.47304300	1.80803800
H	-4.17370000	-3.32143500	2.05077200
H	-4.07583700	-1.56111200	2.05432300
C	-2.26548800	-2.57140600	2.67205500
H	-1.57634200	-1.73250900	2.50160100
H	-2.53335400	-2.57096400	3.73626400
H	-1.71696200	-3.49568000	2.45042800
C	-1.56414500	1.18659000	1.96756100
C	-0.17460700	1.11673500	2.09327200
C	-2.28018500	2.14050900	2.70137100
C	0.47480900	2.04577600	2.91222000
C	-1.62830800	3.04897300	3.51925500
H	-3.36226000	2.15187200	2.59118600
C	-0.23664300	3.00694400	3.61526500
H	1.56296400	2.00213000	2.98887000
H	-2.19799000	3.78881600	4.07933100
H	0.29284000	3.72186000	4.24358000
C	5.02842100	-2.30170600	-1.00091500
C	5.74615800	-1.49019100	-0.12641200
C	5.13778300	-1.02127300	1.03260500
C	3.82832200	-1.38517600	1.33565500
C	3.10894000	-2.21426800	0.47526000
C	3.71930900	-2.65395700	-0.70176100
H	5.49444500	-2.67172100	-1.91349200
H	6.78124700	-1.22813900	-0.34772600
H	5.68743900	-0.37157100	1.71351500
H	3.37489800	-1.05100800	2.26701900
H	3.15854000	-3.30111500	-1.37861400
C	1.71266300	-2.71476500	0.77249100
H	1.66206200	-3.76290900	0.44799700
O	1.55356100	-2.72875300	2.21352400
C	0.86927500	-3.75900400	2.73961900

O	0.42816500	-4.68350800	2.09982900
C	0.73870000	-3.58677200	4.22448800
H	0.25961100	-2.62530900	4.44809300
H	0.14459800	-4.40431700	4.63992000
H	1.73036100	-3.57165900	4.69171400
C	0.58686900	-2.00530900	0.06414400
H	0.18375500	-2.57904200	-0.76596900
C	0.10854800	-0.75788700	0.32108500
C	0.66499300	0.06092700	1.44911500
H	0.98465200	-0.62577300	2.24873100
H	1.59252800	0.53249200	1.08231800

H′

SCF Energy (a.u.) = -2537.494788

Thermal correction to Gibbs free Energy (a.u.) = 0.684021

Charge = 0, Multiplicity = 1

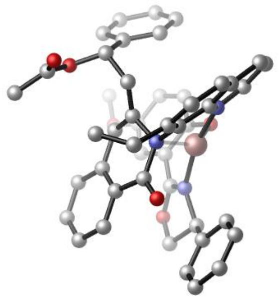

H′

Co	0.50070600	-0.37932400	-1.41470200
O	3.31431500	-0.21776000	0.92946600
O	-1.03911500	-0.77739900	-2.48354100
O	-0.34315200	-4.21189500	-0.34430400
N	0.71003800	1.31355100	-2.18804000
N	1.33777300	0.79128900	0.34286300
N	0.57017800	-2.25453800	-0.94192700
C	0.20134400	1.63138000	-3.37677400
H	-0.53500900	0.92357500	-3.75487200
C	0.58465900	2.77846700	-4.08793100
H	0.13394600	2.97572100	-5.05776700
C	1.53438900	3.61655700	-3.55613000
H	1.87373200	4.50499100	-4.08785200
C	2.07185300	3.32709100	-2.28219800
C	1.61714600	2.16805600	-1.60865100
C	2.05422600	1.88097300	-0.28828800
C	2.96076700	2.71029600	0.35988200
C	3.45516500	3.82334600	-0.37198500

H	4.18489000	4.46610800	0.11950900
C	3.02590100	4.13964500	-1.63235200
H	3.40369100	5.02484400	-2.14363400
C	-2.01875500	-1.38363600	-1.87791300
C	-3.35776400	-1.03430500	-2.16278400
H	-3.52078600	-0.25392000	-2.90397700
C	-4.42927100	-1.60682200	-1.50284000
H	-5.43295200	-1.25891000	-1.74249800
C	-4.23181200	-2.60158800	-0.53232400
C	-2.94229400	-3.02309800	-0.26716900
H	-2.77806500	-3.81643000	0.46079500
C	-1.84963100	-2.42968600	-0.91460900
C	-0.51625300	-2.92289000	-0.70945500
C	1.07557700	-4.36691200	-0.16859400
H	1.34571700	-5.38723400	-0.45408200
H	1.31479000	-4.19066600	0.89309400
C	1.63365400	-3.27906700	-1.06529000
H	1.56981600	-3.63497400	-2.11231100
C	3.04293200	-2.81464000	-0.85206700
C	3.91781500	-3.45929900	0.01949700
H	3.57082800	-4.29549700	0.62842100
C	5.24552300	-3.04887400	0.11699200
H	5.92038500	-3.56249400	0.80043700
C	5.70721900	-1.99052200	-0.65613400
H	6.74395100	-1.66734800	-0.57585500
C	4.83955300	-1.34560100	-1.53401500
H	5.19058400	-0.50960000	-2.13716100
C	3.52039400	-1.76175300	-1.63784200
H	2.83038100	-1.25946600	-2.32337100
C	2.10962400	-0.16410100	1.06608800
O	-5.23547400	-3.20173100	0.17901400
C	-6.53737400	-2.73694500	-0.03353500
H	-6.87284500	-2.90336700	-1.07031700
H	-7.18639600	-3.30257000	0.64157100

H	-6.63222500	-1.66210800	0.19599600
C	3.46611700	2.54874500	1.77306500
H	4.15599200	3.38194100	1.96757400
H	4.06677800	1.63145700	1.82530700
C	2.41945200	2.53749900	2.88465600
H	1.77103000	1.65093300	2.85752400
H	2.92230100	2.53570100	3.85944200
H	1.76203900	3.41457500	2.84623000
C	1.36194600	-1.08456600	1.94887000
C	-0.03068100	-1.04263000	2.05367300
C	2.09749600	-2.04891900	2.64391900
C	-0.67852100	-1.99986000	2.83429200
C	1.44498500	-2.98902900	3.42664200
H	3.18024200	-2.04728400	2.53119000
C	0.05136000	-2.96940000	3.51108500
H	-1.76717400	-1.97905700	2.90046000
H	2.01609900	-3.74159400	3.96767100
H	-0.46808100	-3.71220300	4.11451300
C	-3.61218600	2.45857500	-1.95167800
C	-4.86523200	2.02565400	-1.52545300
C	-5.09776900	1.82797600	-0.16974300
C	-4.09327300	2.07908300	0.76184100
C	-2.84158000	2.52242700	0.34082000
C	-2.60681200	2.69999400	-1.02588100
H	-3.40831900	2.59682400	-3.01314300
H	-5.65293300	1.82828000	-2.25189600
H	-6.07060400	1.47568000	0.17166100
H	-4.28112400	1.93142200	1.82295200
H	-1.62215500	3.02345800	-1.36812300
C	-1.74309800	2.89398300	1.30993900
H	-1.69949600	3.98947300	1.39893900
O	-2.08558900	2.38756600	2.61536800
C	-1.35078300	2.87212800	3.63845800
O	-0.61627000	3.82453000	3.54680300

C	-1.54174700	2.02545600	4.85878600
H	-0.98414700	1.08832300	4.71418600
H	-1.15008400	2.54044000	5.73878400
H	-2.59445300	1.76005600	5.00035900
C	-0.37953800	2.42604100	0.87797000
H	0.28721500	3.21205500	0.53146200
C	0.02171700	1.14845700	0.84790600
C	-0.81281500	-0.00478300	1.31440000
H	-1.62648900	0.36435600	1.94798500
H	-1.30556200	-0.46975200	0.44218100

I′

SCF Energy (a.u.) = -1493.423253

Thermal correction to Gibbs free Energy (a.u.) = 0.431005

Charge = 0, Multiplicity = 1

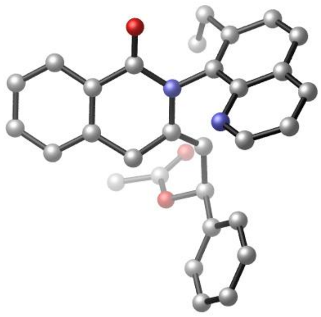

I′

O	0.49278800	-3.40033500	-1.19111600
N	2.22999300	0.28974600	-1.43085300
N	0.38012100	-1.31758700	-0.26530800
C	3.09717200	1.11028700	-1.97689100
H	2.72335900	1.73241500	-2.79410900
C	4.44429600	1.23221800	-1.57065400
H	5.10260600	1.94119800	-2.06751300
C	4.88844000	0.43215200	-0.55065500
H	5.92004000	0.47881100	-0.20058100
C	3.99618300	-0.47431900	0.06215600
C	2.65699400	-0.50312300	-0.41274900
C	1.72503100	-1.38802800	0.20423800
C	2.10564100	-2.24190300	1.22173100
C	3.45612400	-2.20093400	1.65540300
H	3.75518200	-2.87444000	2.45833500
C	4.37329100	-1.34415700	1.10969600
H	5.40179200	-1.32406600	1.47031900
C	-0.14713300	-2.38312800	-0.98496300
C	1.15464100	-3.20450900	1.88198100

H	1.74010800	-3.86253600	2.53982200
H	0.72119600	-3.85310300	1.10820500
C	0.04121400	-2.54897500	2.69696400
H	-0.64023600	-1.95852700	2.07276600
H	-0.55669200	-3.31312200	3.20766000
H	0.44388100	-1.87387100	3.46388700
C	-1.55554300	-2.22216400	-1.42906100
C	-2.14691800	-0.95764600	-1.48203700
C	-2.27261500	-3.35653000	-1.80346500
C	-3.47125800	-0.84394600	-1.89369500
C	-3.59396700	-3.23599800	-2.21062000
H	-1.77024300	-4.32098100	-1.76376500
C	-4.19277100	-1.97859600	-2.25168800
H	-3.93512400	0.14228200	-1.93367200
H	-4.16027800	-4.12034800	-2.49622800
H	-5.23035000	-1.88089600	-2.56743500
C	1.03877700	4.25995600	-0.82567400
C	0.07638400	5.22211300	-1.11486800
C	-1.20406500	5.08859200	-0.58939200
C	-1.52188700	4.00354300	0.22116500
C	-0.56383400	3.03117500	0.50717800
C	0.71997400	3.17093300	-0.02402800
H	2.04587000	4.35002500	-1.23132700
H	0.32273200	6.07174600	-1.74951200
H	-1.96549500	5.83483000	-0.81132800
H	-2.52560300	3.90118700	0.62762100
H	1.47313000	2.41043500	0.18134200
C	-0.86819000	1.86458200	1.42057100
H	-0.60836900	2.13454500	2.45488900
O	-2.29936500	1.65231900	1.39603400
C	-2.78325500	0.82571500	2.34682800
O	-2.13036600	0.40483400	3.26898300
C	-4.21403500	0.48825800	2.05657700
H	-4.24439000	-0.19230300	1.19459100

H	-4.66299300	-0.00617100	2.92063800
H	-4.78372200	1.38327100	1.78582000
C	-0.13781600	0.59636100	1.08399900
H	0.59460400	0.25012700	1.81120700
C	-0.34931400	-0.12413100	-0.02378800
C	-1.30418000	0.22802800	-1.11895600
H	-1.92782200	1.08344000	-0.84383500
H	-0.69272900	0.53144100	-1.98772000
